# Comparative efficacy of serotonin (5-HT_3_) receptor antagonists in patients undergoing surgery: a systematic review and network meta-analysis

**DOI:** 10.1186/s12916-015-0371-y

**Published:** 2015-06-18

**Authors:** Andrea C. Tricco, Charlene Soobiah, Erik Blondal, Areti A. Veroniki, Paul A. Khan, Afshin Vafaei, John Ivory, Lisa Strifler, Huda Ashoor, Heather MacDonald, Emily Reynen, Reid Robson, Joanne Ho, Carmen Ng, Jesmin Antony, Kelly Mrklas, Brian Hutton, Brenda R. Hemmelgarn, David Moher, Sharon E. Straus

**Affiliations:** Li Ka Shing Knowledge Institute, St. Michael’s Hospital, 209 Victoria Street, East Building, Toronto, ON M5B 1W8 Canada; Epidemiology Division, Dalla Lana School of Public Health, University of Toronto, 6th floor, 155 College St, Toronto, ON M5T 3M7 Canada; Institute for Health Policy Management & Evaluation, University of Toronto, 4th Floor, 155 College St, Toronto, ON M5T 3M6 Canada; Departments of Community Health Sciences, Faculty of Medicine, University of Calgary, TRW Building, 3rd Floor, 3280 Hospital Drive, Calgary, AB T2N 4Z6 Canada; Clinical Epidemiology Program, Centre for Practice-Changing Research, Ottawa Hospital Research Institute, 725 Parkdale Ave., Ottawa, ON K1Y 4E9 Canada; Department of Geriatric Medicine, University of Toronto, 27 Kings College Circle, Toronto, ON M5S 1A1 Canada

**Keywords:** Network meta-analysis, Postoperative nausea, Postoperative vomiting, Serotonin receptor antagonists, Systematic review

## Abstract

**Background:**

Serotonin (5-HT_3_) receptor antagonists are commonly used to decrease nausea and vomiting for surgery patients. We conducted a systematic review on the comparative efficacy of 5-HT_3_ receptor antagonists.

**Methods:**

Searches were done in MEDLINE, Embase, and the Cochrane Central Register of Controlled Trials to identify studies comparing 5-HT_3_ receptor antagonists with each other, placebo, and/or combined with other antiemetic agents for patients undergoing surgical procedures. Screening search results, data abstraction, and risk of bias assessment were conducted by two reviewers independently. Random-effects pairwise meta-analysis and network meta-analysis (NMA) were conducted. PROSPERO registry number: CRD42013003564.

**Results:**

Overall, 450 studies and 80,410 patients were included after the screening of 7,608 citations and 1,014 full-text articles. Significantly fewer patients experienced nausea with any drug relative to placebo, except for ondansetron plus metoclopramide in a NMA including 195 RCTs and 24,230 patients. Significantly fewer patients experienced vomiting with any drug relative to placebo except for palonosetron plus dexamethasone in NMA including 238 RCTs and 12,781 patients. All agents resulted in significantly fewer patients with postoperative nausea and vomiting versus placebo in a NMA including 125 RCTs and 16,667 patients.

**Conclusions:**

Granisetron plus dexamethasone was often the most effective antiemetic, with the number needed to treat ranging from two to nine.

**Electronic supplementary material:**

The online version of this article (doi:10.1186/s12916-015-0371-y) contains supplementary material, which is available to authorized users.

## Background

Postoperative nausea and/or vomiting can be defined as nausea and/or vomiting within 24 h of surgery [1, 2]. Between 20 % and 65 % of patients undergoing surgery experience postoperative nausea and/or vomiting [2, 3] and the anesthetic agents administered during the procedure have been identified as a contributing factor. Nausea and vomiting are associated with decreased quality of life and patient satisfaction [4, 5]. Vomiting can also cause complications such as aspiration pneumonia [6] and a longer hospital stay [7].

Serotonin (5-HT_3_) receptor antagonists reduce nausea and vomiting by inhibiting vagal nerves in the central nervous system and intestinal mucosa [8]. These agents are recommended by clinical practice guidelines for patients undergoing surgery and at risk for nausea and/or vomiting [9, 10].

We were commissioned by Health Canada to conduct a systematic review and network meta-analysis to assess the comparative efficacy of 5-HT_3_ receptor antagonists.

## Methods

### Protocol

A protocol based on the Preferred Reporting Items for Systematic reviews and Meta-Analysis for Protocols (PRISMA-P) guidelines was developed [11]. We revised our protocol using feedback from the research team and the research users, including Health Canada, a department of the federal government, who posed the original query. The final protocol was registered with PROSPERO (CRD42013003564) and published in an open-access journal [12]. As described in our protocol [12], our initial objective was to include data for patients undergoing surgery and chemotherapy in the overall analysis for both safety and efficacy outcomes. However, due to the extensive number of studies that met the inclusion criteria, we subdivided the analysis and presentation of results in separate papers for chemotherapy and surgery, as well efficacy and safety outcomes [13]. This paper focuses on the efficacy of 5-HT_3_ receptor antagonist for patients undergoing surgery. Our methods are described briefly below.

### Eligibility criteria

We included studies involving patients of any age undergoing any type of surgery and who were given a 5-HT_3_ receptor antagonist for nausea and/or vomiting (Additional file 1: Appendix 1). Randomized controlled trials (RCTs), quasi-RCTs, non-RCTs, interrupted time series, controlled before–after studies, and observational (cohort) studies were eligible for inclusion. We limited our systematic review to trials published in English due to resource constraints, and excluded studies that were identified as fraudulent or were retracted [14]. The primary outcome was the number of patients who vomited, and secondary outcomes were the number of patients with nausea and the number of patients with both postoperative nausea and vomiting (PONV).

### Information sources

Information sources included electronic databases (MEDLINE, EMBASE, and the Cochrane Central Register of Controlled Trials from inception until January 11, 2013), trial protocol registries, and conference proceedings.

### Study selection and data collection

Two reviewers screened the literature search results and potentially relevant full-text articles, independently. The same process was followed for data abstraction and methodological quality/risk of bias appraisal. We contacted authors as necessary; for example, to obtain additional information.

### Appraisal of methodological quality and risk of bias

We used the Cochrane Effective Practice and Organization of Care (EPOC) risk-of-bias tool to assess risk of bias for experimental and quasi-experimental studies [15], and the Newcastle–Ottawa Scale (NOS) [16] for cohort studies.

### Synthesis of included studies

A random-effects pairwise meta-analysis on the odds ratio (OR) scale was performed to combine studies addressing the same clinical outcome and treatment comparison. We decided to apply a random-effects model, as we expected methodological and clinical heterogeneity across the included studies that compared the same pairs of interventions. For studies with dichotomous outcomes where zero events were reported in one treatment arm, we added 0.5 to all cells. Between-study heterogeneity (*τ*^*2*^) was examined using the restricted maximum likelihood (REML) [17] method, and quantified using the *I*^*2*^ statistic [18]. The R 3.1.2 [19] and *metafor* package [20] were employed to conduct all pairwise meta-analyses.

For a connected network diagram, we conducted a random effects network meta-analysis to make inferences on the comparative efficacy of the 5-HT_3_ receptor antagonists [21]. Treatment nodes were selected by the clinicians and statisticians on the research team. If a study compared different doses of a particular intervention, we included only the recommended dose in the analysis [9, 10, 22–30].

Prior to conducting a network meta-analysis, we evaluated the transitivity assumption by examining the comparability of the distributions of potential treatment-effect modifiers across comparisons [31]. These included age (children versus adults), timing of administration (all time points versus during surgery), and risk of bias (all versus removing high risk of bias for randomization, allocation concealment, and blinding of outcome assessor). We evaluated transitivity in each network, by visually comparing the mode of the categorical potential effect modifiers across treatment comparisons [32]. We also assessed statistical inconsistency between different sources of evidence in the network using a global *χ*^2^ test derived from the design-by-treatment interaction model [21]. In the presence of statistically significant inconsistency, we applied the loop-specific approach [33, 34] to locally assess the network and identify the treatment comparisons responsible for inconsistency. In the network meta-analysis and design-by-treatment interaction models, we assumed common within-network heterogeneity, whereas in loop-specific method we assumed common within-loop heterogeneity. We assumed common heterogeneity across treatment comparisons since the included treatments are of the same nature and it was clinically reasonable to share a common heterogeneity parameter. In all approaches, we estimated the magnitude of between-study heterogeneity using the REML method [17]. Important heterogeneity and/or inconsistency would have been explored using network meta-regression analyses adjusting for potential effect modifiers. For each outcome, we carried out subgroup analyses using time of administration of antiemetics (all time periods versus during surgery) and age (all ages versus children), and sensitivity analyses excluding studies with high risk of incomplete outcome data bias. Although our primary analyses were restricted to RCTs only, as a secondary analysis, we included quasi-RCTs and non-RCTs to examine the robustness of the network meta-analysis results.

We present the network meta-analysis summary of treatment effects along with their 95 % confidence interval (CI) and 95 % predictive interval (PrI). The PrI captures both the uncertainty around the summary treatment effect and between-study variance, and shows the interval within which the treatment effect is expected to lie when a future study is conducted [35, 36]. To visually assess the presence of reporting bias (including publication bias and small-study effects), we used the comparison-adjusted funnel plot [32]. We also ranked the effectiveness of the 5-HT_3_ agonist receptors using the surface under the cumulative ranking (SUCRA) curve [37]. Network meta-analyses were conducted in Stata 13.0 [38] using the *mvmeta* command [39].

## Results

### Literature search

**Fig. 1:**
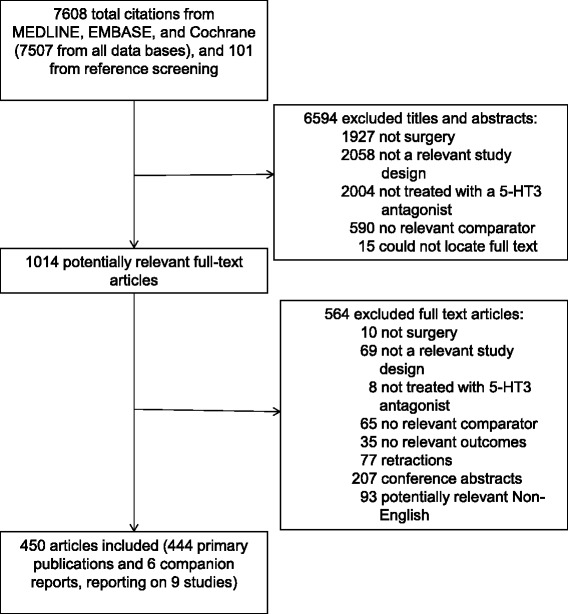
Study flow. Details the flow of information through the different phases of the review, mapping out the number of records identified, included and excluded, and the reasons for their exclusion

The literature search yielded 7,608 citations in total that met the search criteria, of which 450 full text articles met eligibility criteria for inclusion (444 primary publications and six companion reports reporting on nine studies, Fig. 1; Additional file 1: Appendix 2). Five unpublished conference abstracts were included in the review [40–44]. We excluded 77 studies because we suspected or confirmed that their results were fraudulent [14] and 535 studies from previous reviews that did not fulfill our eligibility criteria (reasons for exclusion presented in Additional file 1: Appendix 3).

### Study and patient characteristics

The majority of the included studies had an RCT design (97 %) with a short duration of follow-up of 12 to 24 h (72 %). Most studies were published between 1995 and 2013 (94 %), and were mainly conducted in Asia (39 %), North America (27 %), or Europe (24 %) (Table 1, Additional file 1: Appendix 4).

**Table 1:** Study characteristics

Characteristic	No. of studies ^*^ (n = 444)	Percentage of studies
Year of publication		
1990–1994	25	5.63
1995–1999	141	31.76
2000–2004	110	24.77
2005–2009	107	24.10
2010–2013	61	13.74
Geographic region		
Asia	171	38.51
North America	118	26.58
Europe	108	24.32
Australasia	13	2.93
Multi-continent	12	2.70
Africa	11	2.48
South America	9	2.03
Not reported	2	0.45
Study design		
Randomized clinical trial	429	96.62
Cohort study	9	2.03
Non-randomized clinical trial	5	1.13
Controlled before–after study	1	0.23
Study conduct period		
1990–1999	11	2.48
2000–2009	45	10.14
2010–2013	8	1.80
Not reported	380	85.59
Duration of follow-up ^**^		
0 to ≤6	13	2.93
>6 to ≤12	9	2.03
>12 to ≤24	319	71.85
>24 to ≤48	52	11.71
>48 to ≤72	14	3.15
>72 to ≤1 week	12	2.70
>1 week	3	0.68
Not reported	22	4.95
Interventions examined: frequency ^***^		
*Serotonin antagonists* Reported as administered alone (administered with dexamethasone)		
Ondansetron	336 (46)	75.68 (10.36)
Granisetron	57 (15)	12.84 (3.38)
Tropisetron	35 (2)	7.88 (0.45)
Dolasetron	33 (3)	7.43 (0.68)
Palonosetron	14 (3)	3.15 (0.68)
Ramosetron	10 (1)	2.25 (0.23)
*Comparator antiemetics*		
Butyrophenone	72	16.22
Benzamide	72	16.22
Dexamethasone	40	9.01
Phenothiazine	13	2.93
Antihistamine	11	2.48
NK-1	5	1.13
Anticholinergic	2	0.45
*Serotonin antagonists given with other antiemetic*		
Serotonin antagonist + dexamethasone	70	15.77
Serotonin antagonist + butyrophenone	15	3.38
Serotonin antagonist + benzamide	5	1.13
Serotonin antagonist + antihistamine	3	0.68
Serotonin antagonist + NK-1	2	0.45
Serotonin antagonist + phenothiazine	2	0.45
*Placebo or no treatment*		
	293	65.99
Outcomes examined: frequency ^****^		
Vomiting	347	78.15
Nausea	308	69.40
PONV	268	60.36
Setting		
Not reported	270	60.81
Hospital	113	25.45
Multi-center	31	6.98
Medical center	30	6.76

^*^ Includes unpublished data; ^**^ Duration is in hours unless otherwise noted; ^***^ Multiple interventions and comparators examined across the studies; ^****^ Multiple interventions and outcomes reported per study

*NK-1* neurokinin 1 receptor antagonist, *PONV* postoperative nausea and vomiting

The 5-HT_3_ receptor antagonists we examined were ondansetron (0.1 − 48 mg/day; 76 %), granisetron (0.1 − 3 mg/day; 13 %), tropisetron (0.1 − 5 mg/day; 8 %), dolasetron (12.5 − 200 mg/day; 7 %), palonosetron (0.025 − 0.25 mg/day; 3 %), and ramosetron (0.1 − 0.6 mg/day; 2 %) (Table 1, Additional file 1: Appendix 5). We also included studies comparing combinations of 5-HT_3_ drugs administered concomitantly with other antiemetics, e.g., dexamethasone (2–20 mg/day; 16 %), butyrophenone (3 %), and benzamide (1 %).

Overall, 286 studies with dichotomous outcome data were included in our analyses. Studies with continuous outcome data and studies investigating the same 5-HT_3_ treatment in different doses were not included in the analysis.

The median study size was 118 (interquartile range, 75–180) patients, whereas most patients were women (72 %), and adults (59 %), with an American Society of Anesthesiologists (ASA) physical status [45] of I or II (60 %) undergoing obstetrical and gynecological (30 %) surgery (Table 2, Additional file 1: Appendix 6). The included studies often did not report patients’ history of PONV (56 %). Similarly, a history of motion sickness was reported in only 33 % of the studies; comorbidities were rarely reported (5 %).

**Table 2:** Patient characteristics

Total no. of patients	80,410	
Mean, Median sample size	181, 118	
Mean % female	72	
	No. of studies (n = 444) ^*^	Percentage of studies
Age category		
Children only (aged <18 yr)	75	16.89
Adults only (aged ≥18 yr to ≤65 yr)	262	59.01
Children and adults (aged ≤65 yr)	17	3.83
Adults and elderly (aged ≥18 yr)	76	17.12
All ages	9	2.03
Not reported	5	1.13
American Society of Anesthesiologists (ASA) physical status		
I	15	3.38
I or II	266	59.91
I or II or III	87	19.59
II or III	4	0.90
Not reported	72	16.22
Surgery type		
Obstetric and gynecological	134	30.18
Gastrointestinal	51	11.49
Eye	35	7.88
General dentistry, oral and maxillofacial surgery, and orthodontics	35	7.88
Otolaryngological	20	4.50
Breast	18	4.05
Orthopedic	16	3.60
Neurological	15	3.38
Endocrine	9	2.03
Cardiovascular	3	0.68
Urological	1	0.23
Miscellaneous (includes multiple surgery types, abdominal surgery, and plastic surgery unspecified)	103	23.20
Not reported	4	0.90
History of motion sickness		
Yes	147	33.11
No or not reported	297	66.89
History of postoperative nausea and vomiting		
Yes	197	44.37
No or not reported	247	55.63
Comorbidities ^**^		
Not reported	415	93.47
Diabetes mellitus	9	2.03
Cardiovascular	8	1.80
Obesity	5	1.13
Cancer	4	0.90
Migraines	3	0.68
Gallbladder	2	0.45
Liver disease	2	0.45
Asthma	1	0.23
Disorder of the ear	1	0.23
Mental health	1	0.23
Osteoarthritis	1	0.23
Urological	1	0.23

^*^ Includes unpublished data; ^**^ Some studies considered more than one comorbidity

### Methodological quality and risk of bias

Most of the included RCTs and quasi-RCTs had an unclear or high risk of bias on the following items: allocation concealment (59 %), baseline outcome characteristics (89 %), incomplete outcome data (60 %), and selective outcome reporting bias (97 %). When assessing potential for funding bias, we considered a study at a high or unclear risk of bias when it was funded by a private industry or when an author on the publication was employed by the company sponsoring the study, which occurred in 92 % of the RCTs and quasi-RCTs (Additional file 1: Appendices 7 and 8). Of the nine observational studies included in the analysis, eight used a somewhat representative sample, two did not describe ascertainment of exposure, all failed to demonstrate that the outcome was not present at the start of the study, six did not control for confounders, three did not describe the assessment of outcome, and all neglected to report follow-up (Additional file 1: Appendix 9). The visual inspection of the comparison adjusted funnel plots showed that there is no evidence for small-study effects and publication bias (Additional file 1: Appendix 10).

#### Vomiting

**Fig. 2:**
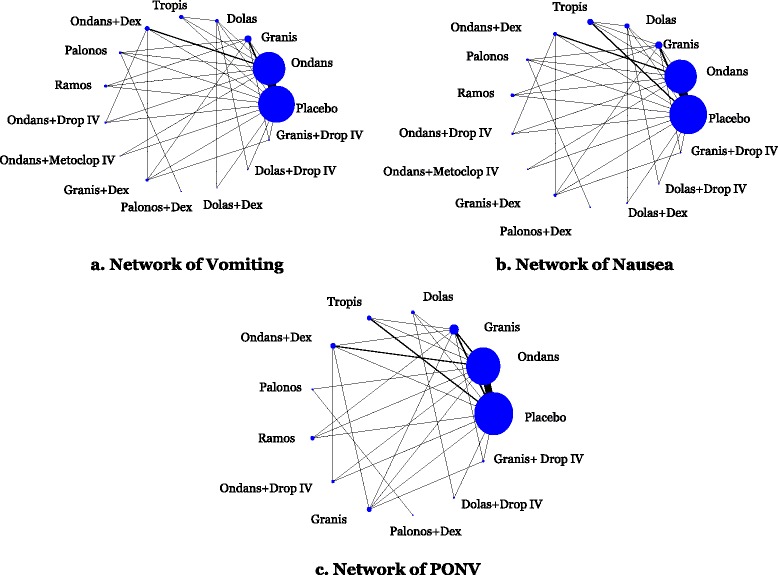
Network geometry. Network meta-analysis diagrams for vomiting, nausea, and PONV. Nodes are weighted according to the number of patients included in the corresponding treatments, and edges are weighted according to the number of studies included in the respective comparisons

**Fig. 3:**
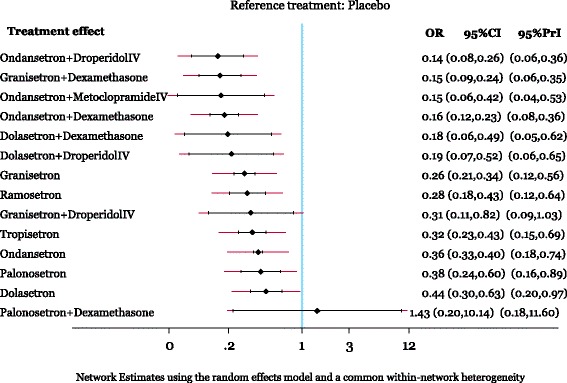
Network meta-analysis results for vomiting. All treatments are compared to placebo. The black horizontal lines represent the 95 % confidence intervals (CI) of the summary treatment effects and red horizontal lines the 95 % predictive intervals (PrI). Results are presented on the odds ratio scale

The network meta-analysis for vomiting included 238 RCTs with a total of 12,781 patients. The network geometry and included drugs can be found in Fig. 2a, whereas the statistically significant results are available in Table 3 and the overall results in Additional file 1: Appendix 11. The following treatment comparisons were statistically significant using both the CIs and PrIs: all agents (except for palonosetron plus dexamethasone and granisetron plus droperidol intravenous (IV)) versus placebo, ondansetron plus droperidol IV versus ondansetron, granisetron plus dexamethasone versus ondansetron, ondansetron plus dexamethasone versus dolasetron, ondansetron plus droperidol IV versus dolasetron, granisetron plus dexamethasone versus dolasetron, palonosetron plus dexamethasone versus ondansetron plus dexamethasone, palonosetron plus dexamethasone versus ondansetron plus droperidol IV, and palonosetron plus dexamethasone versus granisetron plus dexamethasone (Fig. 3). According to the SUCRA (Additional file 1: Appendix 12), the most effective agents for vomiting were ondansetron plus droperidol IV (85 % probability) and granisetron plus dexamethasone (84 % probability). The within-network heterogeneity in the network meta-analysis model was estimated at 0.15, and the evaluation of the network inconsistency using the design-by-treatment interaction model suggested that there was no evidence of statistical inconsistency (*χ*^*2*^ = 49.27, degrees of freedom = 44, *P* = 0.271, heterogeneity variance = 0.15).

**Table 3:** Statistically significant results of network meta-analysis for all time periods of drug administration

	All ages			Children only		
Treatment comparison	No. of studies	MA estimate: OR (95 % CI) ^*^	NMA estimate: OR (95 % CI)	No. of studies	MA estimate: OR (95 % CI) ^*^	NMA estimate: OR (95 % CI)
Vomiting	238 RCTs and 12,781 patients			46 RCTs and 1,830 patients		
Ondansetron vs. placebo	146	0.35 (0.32–0.39)	0.36 (0.33–0.40)	34	0.30 (0.24–0.38)	0.30 (0.24–0.38)
Granisetron vs. placebo	27	0.24 (0.16–0.34)	0.26 (0.21–0.34)	4	0.21 (0.08–0.56)	0.23 (0.12–0.48)
Dolasetron vs. placebo	7	0.42 (0.21–0.83)	0.44 (0.30–0.63)	3	0.41 (0.23–0.75)	0.39 (0.19–0.78)
Tropisetron vs. placebo	15	0.32 (0.22–0.48)	0.32 (0.23–0.43)	3	0.18 (0.09–0.36)	0.18 (0.08–0.41)
Ondansetron + DEX vs. placebo	12	0.16 (0.09–0.27)	0.16 (0.12–0.23)	5	0.06 (0.03–0.17)	0.07 (0.03–0.15)
Palonosetron vs. placebo	4	0.53 (0.38–0.73)	0.38 (0.24–0.60)	NA	NA	NA
Ramosetron vs. placebo	5	0.42 (0.26–0.68)	0.28 (0.18–0.43)	NA	NA	NA
Ondansetron + DROP vs. placebo	2	0.15 (0.07–0.31)	0.14 (0.08–0.26)	1	0.13 (0.05–0.33)	0.11 (0.04–0.33)
Ondansetron + METO vs. placebo	2	0.16 (0.06–0.43)	0.15 (0.06–0.42)	2	0.16 (0.06–0.43)	0.18 (0.06–0.53)
Granisetron + DEX vs. placebo	5	0.16 (0.08–0.31)	0.15 (0.09–0.24)	2	0.08 (0.03–0.27)	0.09 (0.02–0.31)
Dolasetron + DEX vs. placebo	1	0.06 (0.01–0.30)	0.18 (0.06–0.49)	NA	NA	NA
Dolasetron + DROP vs. placebo	1	0.16 (0.07–0.35)	0.19 (0.07–0.52)	NA	NA	NA
Granisetron + DROP vs. placebo	2	0.30 (0.05–1.66)	0.31 (0.11–0.82)	NA	NA	NA
Granisetron vs. ondansetron	12	0.52 (0.34–0.81)	0.73 (0.56–0.94)	NA	NA	0.78 (0.37–1.63)
Ondansetron + DEX vs. ondansetron	15	0.50 (0.33–0.75)	0.46 (0.33–0.63)	3	0.24 (0.12–0.47)	0.23 (0.11–0.49)
Ondansetron + DROP vs. ondansetron	5	0.43 (0.24–0.78)	0.39 (0.21–0.71)	1	0.31 (0.12–0.77)	0.37 (0.13–1.09)
Granisetron + DEX vs. ondansetron	NA	NA	0.41 (0.25–0.67)	NA	NA	0.28 (0.08–1.04)
Dolasetron vs. granisetron	NA	NA	1.66 (1.07–2.57)	NA	NA	1.65 (0.61–4.47)
Ondansetron + DEX vs. granisetron	NA	NA	0.63 (0.42–0.94)	NA	NA	0.30 (0.11–0.83)
Granisetron + DEX vs. granisetron	7	0.39 (0.20–0.77)	0.57 (0.35–0.92)	1	0.14 (0.02–1.23)	0.36 (0.09–1.50)
Ondansetron + DEX vs. dolasetron	NA	NA	0.38 (0.23–0.62)	NA	NA	0.18 (0.07–0.50)
Ondansetron + DROP vs. dolasetron	NA	NA	0.32 (0.16–0.65)	NA	NA	0.29 (0.08–1.04)
Granisetron + DEX vs. dolasetron	NA	NA	0.34 (0.19–0.63)	NA	NA	0.22 (0.05–0.95)
Ondansetron + DEX vs. tropisetron	NA	NA	0.52 (0.33–0.82)	NA	NA	0.40 (0.13–1.22)
Ondansetron + DROP vs. tropisetron	NA	NA	0.45 (0.23–0.88)	NA	NA	0.64 (0.16–2.48)
Granisetron + DEX vs. tropisetron	NA	NA	0.47 (0.26–0.84)	NA	NA	0.48 (0.10–2.25)
Palonosetron vs. ondansetron + DEX	NA	NA	2.32 (1.33–4.07)	NA	NA	NA
Ramosetron vs. ondansetron + DEX	NA	NA	1.71 (1.01–2.90)	NA	NA	NA
Palonosetron + DEX vs. ondansetron + DEX	NA	NA	8.68 (1.19–63.20)	NA	NA	NA
Ondansetron + DROP vs. palonosetron	NA	NA	0.37 (0.17–0.78)	NA	NA	NA
Granisetron + DEX vs. palonosetron	NA	NA	0.39 (0.20–0.75)	NA	NA	NA
Palonosetron + DEX vs. ondansetron + DROP	NA	NA	10.13 (1.31–78.58)	NA	NA	NA
Palonosetron + DEX vs. ondansetron + METO	NA	NA	9.38 (1.03–85.06)	NA	NA	NA
Palonosetron + DEX vs. granisetron + DEX	NA	NA	9.60 (1.28–72.03)	NA	NA	NA
Nausea	195 RCTs and 24,230 patients			11 RCTs and 1,326 patients		
Ondansetron vs. placebo	121	0.46 (0.40–0.52)	0.46 (0.41–0.52)	10	0.44 (0.29–0.68)	0.45 (0.30–0.66)
Granisetron vs. placebo	21	0.35 (0.23–0.52)	0.35 (0.26–0.47)	NA	NA	NA
Dolasetron vs. placebo	9	0.59 (0.48–0.73)	0.60 (0.43–0.86)	1	0.29 (0.11–0.73)	0.26 (0.09–0.74)
Tropisetron vs. placebo	15	0.51 (0.40–0.66)	0.48 (0.35–0.65)	NA	NA	NA
Ondansetron + DEX vs. placebo	8	0.21 (0.14–0.34)	0.28 (0.19–0.41)	2	0.21 (0.08–0.57)	0.22 (0.08–0.61)
Palonosetron vs. placebo	3	0.48 (0.33–0.68)	0.30 (0.17–0.53)	NA	NA	NA
Ramosetron vs. placebo	5	0.35 (0.18–0.68)	0.32 (0.22–0.47)	NA	NA	NA
Ondansetron + DROP vs. placebo	2	0.31 (0.06–1.55)	0.26 (0.14–0.49)	1	0.07 (0.00–1.26)	0.07 (0.00–1.38)
Granisetron + DEX vs. placebo	4	0.21 (0.11–0.39)	0.20 (0.12–0.34)	1	0.09 (0.02–0.49)	0.10 (0.02–0.53)
Dolasetron + DEX vs. placebo	1	0.28 (0.05–1.53)	0.21 (0.07–0.61)	NA	NA	NA
Dolasetron + DROP vs. placebo	1	0.17 (0.08–0.36)	0.19 (0.07–0.54)	NA	NA	NA
Granisetron + DROP vs. placebo	2	0.22 (0.08–0.61)	0.21 (0.07–0.62)	NA	NA	NA
Ondansetron + DEX vs. ondansetron	14	0.68 (0.48–0.96)	0.61 (0.42–0.89)	1	0.53 (0.11–2.60)	0.48 (0.16–1.43)
Granisetron + DEX vs. ondansetron	NA	NA	0.43 (0.25–0.73)	NA	NA	0.21 (0.04–1.23)
Dolasetron vs. granisetron	NA	NA	1.73 (1.10–2.72)	NA	NA	NA
Granisetron + DEX vs. granisetron	6	0.59 (0.39–0.88)	0.56 (0.33–0.95)	NA	NA	NA
Ondansetron + DEX vs. dolasetron	NA	NA	0.47 (0.28–0.78)	NA	NA	0.81 (0.19–3.50)
Palonosetron vs. dolasetron	NA	NA	0.50 (0.26–0.97)	NA	NA	NA
Ramosetron vs. dolasetron	NA	NA	0.53 (0.31–0.88)	NA	NA	NA
Ondansetron + DROP vs. dolasetron	NA	NA	0.44 (0.21–0.89)	NA	NA	0.28 (0.01–6.17)
Granisetron + DEX vs. dolasetron	NA	NA	0.33 (0.17–0.62)	NA	NA	0.36 (0.05–2.66)
Dolasetron + DEX vs. dolasetron	2	0.33 (0.15–0.72)	0.35 (0.13–0.97)	NA	NA	NA
Dolasetron + DROP vs. dolasetron	1	0.35 (0.17–0.73)	0.32 (0.11–0.89)	NA	NA	NA
Ondansetron + DEX vs. tropisetron	NA	NA	0.59 (0.36–0.95)	NA	NA	NA
Granisetron + DEX vs. tropisetron	NA	NA	0.41 (0.22–0.76)	NA	NA	NA
Postoperative nausea and vomiting	125 RCTs and 16,667 patients			14 RCTs and 2,394 patients		
Ondansetron vs. placebo	89	0.30 (0.26–0.35)	0.31 (0.27–0.36)	11	0.33 (0.18–0.60)	0.34 (0.19–0.62)
Granisetron vs. placebo	16	0.23 (0.14–0.37)	0.23 (0.16–0.32)	2	0.54 (0.26–1.12)	0.32 (0.08–1.23)
Dolasetron vs. placebo	5	0.27 (0.14–0.52)	0.25 (0.14–0.43)	NA	NA	NA
Tropisetron vs. placebo	8	0.39 (0.28–0.53)	0.36 (0.24–0.54)	NA	NA	NA
Ondansetron + DEX vs. placebo	8	0.12 (0.07–0.20)	0.15 (0.10–0.22)	1	0.20 (0.06–0.66)	0.27 (0.04–1.61)
Palonosetron vs. placebo	NA	NA	0.11 (0.03–0.40)	NA	NA	NA
Ramosetron vs. placebo	4	0.30 (0.15–0.59)	0.26 (0.16–0.41)	NA	NA	NA
Ondansetron + DROP vs. placebo	2	0.13 (0.05–0.34)	0.11 (0.05–0.24)	1	0.12 (0.04–0.34)	0.12 (0.02–0.71)
Granisetron + DEX vs. placebo	4	0.06 (0.02–0.17)	0.09 (0.05–0.16)	NA	NA	NA
Palonosetron + DEX vs. placebo	NA	NA	0.12 (0.02–0.64)	NA	NA	NA
Dolasetron + DROP vs. placebo	1	0.17 (0.08–0.36)	0.12 (0.04–0.35)	NA	NA	NA
Granisetron + DROP vs. placebo	2	0.17 (0.07–0.38)	0.16 (0.06–0.40)	NA	NA	NA
Ondansetron + DEX vs. ondansetron	11	0.48 (0.33–0.72)	0.46 (0.31–0.69)	1	1.00 (0.36–2.75)	0.78 (0.13–4.65)
Ondansetron + DROP vs. ondansetron	3	0.39 (0.20–0.75)	0.35 (0.16–0.77)	NA	NA	NA
Granisetron + DEX vs. ondansetron	1	0.22 (0.04–1.21)	0.30 (0.17–0.53)	NA	NA	NA
Granisetron + DEX vs. granisetron	8	0.48 (0.29–0.77)	0.41 (0.24–0.69)	NA	NA	NA
Granisetron + DEX vs. dolasetron	NA	NA	0.37 (0.17–0.82)	NA	NA	NA
Ondansetron + DEX vs. tropisetron	NA	NA	0.41 (0.23–0.73)	NA	NA	NA
Ondansetron + DROP vs. tropisetron	NA	NA	0.31 (0.13–0.75)	NA	NA	NA
Granisetron + DEX vs. tropisetron	NA	NA	0.26 (0.13–0.52)	NA	NA	NA
Granisetron + DEX vs. ramosetron	NA	NA	0.36 (0.18–0.75)	NA	NA	NA

^*^ Meta-analysis was not conducted for treatment comparisons where only 1 trial was included. In that situation, the direct estimate was obtained from the single trial

*CI* confidence interval, *DEX* dexamethasone, *DROP* droperidol (intravenous), *MA* meta-analysis, *METO* metoclopramide (intravenous), *NA* not applicable, *NMA* network meta-analysis, *OR* odds ratio

In order to account for the treatment effect modifier ‘age’ (Additional file 1: Appendix 13), a subgroup analysis was conducted for 46 RCTs involving a total of 1,830 children (Table 3, Additional file 1: Appendix 11). The following treatment comparisons were statistically significant for vomiting: ondansetron versus placebo, granisetron versus placebo, dolasetron versus placebo, tropisetron versus placebo, ondansetron plus dexamethasone versus placebo, ondansetron plus droperidol lV versus placebo, ondansetron plus metoclopramide IV versus placebo, granisetron plus dexamethasone versus placebo, ondansetron plus dexamethasone versus ondansetron, ondansetron plus dexamethasone versus dolasetron, ondansetron plus dexamethasone versus granisetron, and granisetron plus dexamethasone versus dolasetron (Additional file 1: Appendix 11). According to the SUCRA, the most effective agents for vomiting in children were ondansetron plus dexamethasone (83 % probability) and granisetron plus dexamethasone (82 % probability).

In order to account for the treatment effect modifier ‘timing of administration’ (Additional file 1: Appendix 14), a subgroup analysis was conducted for 220 RCTs involving 10,811 patients when the agents were administered during surgery (Additional file 1: Appendix 15). The results were the same as for the primary analysis, except that ondansetron plus droperidol IV was statistically superior to granisetron, and ondansetron plus droperidol IV and granisetron plus dexamethasone were superior to ramosetron. According to the SUCRA for this subgroup analysis, the most effective agents for vomiting were ondansetron plus droperidol IV (88 % probability) and granisetron plus dexamethasone (84 % probability).

In order to account for the treatment effect modifier ‘risk of bias’, a sensitivity analysis was conducted in which 11 RCTs were removed because of high risk of incomplete outcome data bias (Additional file 1: Appendix 15); the same results were observed, except granisetron plus dexamethasone was associated with significantly less vomiting compared with ramosetron (OR, 0.52;, 95 % CI, 0.27–0.99). In another sensitivity analysis, in which four cohort studies [46–49], two non-randomized controlled trials [50, 51], and one controlled before–after study [52] were added to the included studies, all of the results were the same, except that the differences between ondansetron plus dexamethasone and granisetron or ramosetron and between ondansetron plus metoclopramide IV and palonosetron plus dexamethasone were no longer statistically significant (Additional file 1: Appendix 15).

#### Nausea

The network meta-analysis for nausea included 195 RCTs with a total of 24,230 patients. The network geometry and included drugs can be found in Fig. 2b. We present the statistically significant treatment effect estimates derived through the network meta-analysis model in Table 3 and overall results in Additional file 1: Appendix 11. Using both the CIs and PrIs, the only treatment comparisons that were statistically significant for nausea were granisetron versus placebo (OR, 0.35; 95 % PrI, 0.13–0.91), ondansetron plus dexamethasone versus placebo (OR, 0.28; 95 % PrI, 0.10–0.76), palonosetron versus placebo (OR, 0.30; 95 % PrI, 0.10–0.89), ramosetron versus placebo (OR, 0.32; 95 % PrI, 0.12–0.86), ondansetron plus droperidol IV versus placebo (OR, 0.26; 95 % PrI, 0.09–0.80), granisetron plus dexamethasone versus placebo (OR, 0.20; 95 % PrI, 0.07–0.57), dolasetron plus droperidol IV versus placebo (OR, 0.19; 95 % PrI, 0.05–0.77), and granisetron plus droperidol IV versus placebo (OR, 0.21; 95 % PrI, 0.05–0.87) (Additional file 1: Appendices 11 and 16). According to the SUCRA (Additional file 1: Appendix 17), the most effective agents for nausea were granisetron plus dexamethasone (82 % probability) and dolasetron plus droperidol IV (78 % probability). The within-network heterogeneity in the network meta-analysis model was estimated at 0.24, and the design-by-treatment interaction model suggested that there was no statistically significant inconsistency (*χ*^2^ = 26.65, degrees of freedom = 41, *P* = 0.959, heterogeneity variance = 0.27).

In order to account for the treatment effect modifier ‘age’ (Additional file 1: Appendix 13), a subgroup analysis was conducted for 11 RCTs involving 1,326 children (Table 3, Additional file 1: Appendix 11). The following treatment comparisons were statistically significant for nausea: ondansetron versus placebo, dolasetron versus placebo, ondansetron plus dexamethasone versus placebo, and granisetron plus dexamethasone versus placebo (Additional file 1: Appendix 11). According to the SUCRA, the most effective agents for nausea in children were granisetron plus dexamethasone (84 % probability) and ondansetron plus droperidol IV (81 % probability).

In order to account for the treatment effect modifier ‘timing of administration’ (Additional file 1: Appendix 14), a subgroup analysis was conducted for 175 RCTs involving 21,844 patients when the agents were administered during surgery (Additional file 1: Appendix 18). All of the results were the same as for the primary analysis, except that the difference between tropisetron and ondansetron plus dexamethasone was no longer statistically significant. According to the SUCRA for this subgroup analysis, the most effective agents for nausea were granisetron plus dexamethasone (82 % probability) and dolasetron plus droperidol IV (77 % probability).

In order to account for the treatment effect modifier ‘risk of bias’, a sensitivity analysis was conducted in which 10 RCTs were removed because of high risk of incomplete outcome data bias [53–62], and the results were unchanged from the primary analysis (Additional file 1: Appendix 18). In another sensitivity analysis, in which two cohort studies [46, 49] and two non-randomized controlled trials [50, 51] were added to the included studies, all of the results were the same, except that the differences between palonosetron and dolasetron and between dolasetron plus dexamethasone and dolasetron were no longer statistically significant (Additional file 1: Appendix 18).

#### Postoperative nausea and vomiting

The network meta-analysis for PONV included 125 RCTs with 16,667 patients. The network geometry and included drugs can be found in Fig. 2c, statistically significant results are presented in Table 3, and the overall results in Additional file 1: Appendix 11. According to both the CIs and PrIs, the following treatment comparisons were statistically significant for PONV: all agents versus placebo except for tropisetron, granisetron plus dexamethasone versus ondansetron, and granisetron plus dexamethasone versus tropisetron (Additional file 1: Appendix 19). According to the SUCRA, the most effective agents for PONV were granisetron plus dexamethasone (86 % probability) and ondansetron plus droperidol IV (78 % probability; Additional file 1: Appendices 11 and 20). The within-network heterogeneity in the network meta-analysis model was estimated at 0.25, and the design-by-treatment interaction model suggested that there was no statistically significant inconsistency (*χ*^2^ = 26.58, degrees of freedom = 32, *P* = 0.737, heterogeneity variance = 0.26).

In order to account for the treatment effect modifier ‘age’ (Additional file 1: Appendix 13), a subgroup analysis was conducted for 14 RCTs involving a total of 2,394 children (Table 3, Additional file 1: Appendix 11). The following treatment comparisons were statistically significant: ondansetron versus placebo and ondansetron plus droperidol IV versus placebo. According to the SUCRA, the most effective agents for PONV in children were ondansetron plus droperidol IV (85 % probability) and ondansetron plus dexamethasone (59 % probability).

In order to account for the treatment effect modifier ‘timing of administration’ (Additional file 1: Appendix 14), a subgroup analysis was conducted for 116 RCTs involving 12,415 patients in which the agents were administered during surgery (Additional file 1: Appendix 21). All of the results were the same as for the primary analysis, except that ondansetron plus dexamethasone was statistically superior to ramosetron. According to the SUCRA for this subgroup analysis, the most effective agents for PONV were granisetron plus dexamethasone (84 % probability) and ondansetron plus droperidol IV (79 % probability).

In order to account for the treatment effect modifier ‘risk of bias’, a sensitivity analysis was conducted in which 10 RCTs were removed because of high risk of incomplete outcome data bias (Additional file 1: Appendix 21) [53–55, 58, 59, 61–65]; the results were unchanged from the primary analysis, except that the risk of PONV was significantly higher with tropisetron than with granisetron. In another sensitivity analysis, two cohort studies [46, 49] and three non-randomized controlled trials [50, 51, 66] were added to the included studies, and the same results were observed (Additional file 1: Appendix 11).

## Discussion

Administration of most 5-HT_3_ antagonists led to significantly fewer patients experiencing nausea, vomiting, and PONV relative to placebo. However, some of the corresponding PrIs were not statistically significant, suggesting that the statistically significant treatment effects might change should a new study become available. For all age groups and across all outcomes, the most effective agents were granisetron plus dexamethasone. For adults, the most effective agents were ondansetron plus droperidol IV; and for children, the most effective agents were ondansetron plus dexamethasone.

We also conducted a systematic review and network meta-analysis on the safety of these medications [13]. Our network meta-analysis results suggested that granisetron plus dexamethasone increases the risk of arrhythmia. However, a statistically significant increase in the risk of delirium was not observed in another network meta-analysis. In a meta-analysis including three studies for ondansetron versus placebo, no statistically significant results were observed for mortality. Only two studies reported prolongation of the QT interval; meta-analysis was not feasible because the studies compared different interventions.

Our network meta-analysis results for vomiting and PONV are similar to those of a previous network meta-analysis that examined only these two outcomes [67]. The only difference was that, unlike the current study, the earlier analysis showed that granisetron was significantly better than ondansetron and dolasetron for PONV. However, we included 378 studies involving a total of 68,167 patients that were not included in the earlier review. Although we are aware of other systematic reviews and meta-analyses of 5-HT_3_ receptor antagonists [14, 68], the previous researchers did not conduct a network meta-analysis, and therefore the results cannot be compared. Notably, because of our comprehensive literature search and broad eligibility criteria, we included 205 studies involving a total of 43,075 patients that were not included in those previous reviews (Additional file 1: Appendix 22).

The included studies were limited by having an unclear or high risk of bias on important components, including allocation concealment, selective outcome reporting bias, and potential for funding bias. Further, this systematic review process had some inherent limitations. Slight changes to the original protocol [12] were necessary, such as preparing a separate paper for patients undergoing chemotherapy, as well as one focused on the safety of these agents for patients with surgery [13]. Furthermore, it was assumed that the effects of the different doses and durations were identical across the treatments, and that they defined the same node they belong to. We are currently exploring these assumptions in another paper [69]. Although study designs above and beyond RCTs were included, the network meta-analysis was limited to the RCTs in order to increase the confidence of the results. We also were unable to present the results from the hundreds of meta-analyses conducted, as well as the raw data; these are available from the corresponding author upon request. Although the analyses were adjusted to account for the treatment effect modifiers (age, timing of administration, and risk of bias), the results might be influenced by effect modifiers that we were unaware of. However, the statistical evaluation of the transitivity assumption using the design-by-treatment interaction model suggested there was no evidence of inconsistency. Finally, 77 studies were excluded because they contained data known to be fraudulent or were retracted [14].

## Conclusions

In conclusion, granisetron plus dexamethasone was often the most effective antiemetic across the effectiveness outcomes considered here, with the number needed to treat ranging from two to nine. A study that examines the administration of these agents at different dosages would provide further clarity to this important issue and our team is currently working on such an initiative [70].

## Additional file

Additional file 1:
**Appendices 1–22.**

## Abbreviations

5-HT_3_: Serotonin
CI: Confidence interval
IV: Intravenous.
OR: Odds ratio
PONV: Postoperative nausea and vomiting
PrI: Predictive interval
RCTs: Randomized control trials
REML: Restricted maximum likelihood
SUCRA: Surface under the cumulative ranking
