# Supplementary material for: Comparative efficacy of serotonin (5-HT3) receptor antagonists in patients undergoing surgery: a systematic review and network meta-analysis
Source: BMC Med. 2015 Jun 18;13:136. doi: 10.1186/s12916-015-0371-y (PMC4472258; doi:10.1186/s12916-015-0371-y)
Supplement: Additional file 1: — Appendices 1–22. [file 12916_2015_371_MOESM1_ESM.pdf]

APPENDICES

Appendix 1. Inclusion criteria for interventions and comparator groups 2
Appendix 2. Included Studies with References for each analysis and sub-group analysis 3
Appendix 3. List of excluded studies from previous systematic reviews 24
Appendix 4. Study characteristics 36
Appendix 5. Frequencies for specific drugs and drug combinations 55
Appendix 6. Patient characteristics 56
Appendix 7. Cochrane EPOC Risk-of-bias appraisal results 70
Appendix 8. Aggregate EPOC risk-of-bias appraisal results 78
Appendix 9. Newcastle-Ottawa Scale appraisal results 79
Appendix 10. Comparison adjusted funnel plots 80
Appendix 11: Network meta-analysis results for patients of all ages and children only for all time periods 81
Appendix 12. Plot of the surface under the cumulative ranking (SUCRA) curves for the 15 treatments included in Vomiting. 90
Appendix 13. Network plots using colored edges according to the levels of the treatment effect modifier age of participants* 91
Appendix 14. Network plots using colored edges according to the levels of the treatment effect modifier timing of administration 92
Appendix 15. Vomiting sub-group analysis results 93
Appendix 16. Nausea network meta-analysis results forest plot versus placebo 99
Appendix 17. Plot of the surface under the cumulative ranking (SUCRA) curves for the 15 treatments included in Nausea. 100
Appendix 18. Nausea sub-group analysis results 101
Appendix 19. PONV network meta-analysis results forest plot versus placebo 107
Appendix 20. Plot of the surface under the cumulative ranking (SUCRA) curves for the 13 treatments included in PONV. 108
Appendix 21. PONV sub-group analysis results 109
Appendix 22. Included studies in our review versus previous systematic reviews 114

Appendix 1. Inclusion criteria for interventions and comparator groups

Intervention:	***Eligible comparators:***
5-HT3 receptor antagonist with or without dexamethasone (Ondansetron, Granisetron, Dolasetron, Tropisetron, Ramosetron, Palonosetron)	I. Different 5-HT3 antagonist with or without Dexamethasone
	II. Antiemetic comparator listed below with or without Dexamethasone
	III. Same 5-HT3 antagonist given at different dose, formula, and/or timing or same 5-ht3 with Dexamethasone
	IV. Same 5-HT3 antagonist given in combination with one of the comparators below
	V. Different 5-HT3 antagonist given in combination with one of the comparators below

Antiemetic comparators included:

**Benzamides**	**Phenothiazine**	**Butyrophenones**	**Antihistamines**	**Anticholinergics**	**NK-1**	**Steroids**	**NaSSA**
Metoclopramide	Perphenazine	Haloperidol	Dimenhydrinate	Scopolamine	Aprepitant	Dexamethasone	Mirtazapine
Azasetron	Prochlorperazine	Droperidol	Cyclizine		Casopitant		
Alizapride	Promethazine				Rolapitant		
					CP-122, 721		

Abbreviations: 5-HT3, Serotonin; NK-, Neurokinin-1 receptor antagonists; NaSSA, Noradrenergic and specific serotonergic antidepressant.

Appendix 2. Included Studies with References for each analysis and sub-group analysis

Literature Search

450 potentially relevant full-text articles (444 primary publications [1-439][440-447] and 6 companion reports (reporting on nine studies) [235, 443, 444, 448-450].

Vomiting Analyses

1. Network meta-analysis for vomiting included 238 RCTs, with a total of 12, 781 patients [1-6, 9, 12, 13, 15-18, 21, 24, 25, 27, 29, 30, 32, 34-36, 40, 42-44, 48, 53-56, 58, 59, 61, 64, 67, 68, 70, 71, 73, 74, 79-82, 84-86, 99, 100, 102, 103, 105, 107-109, 111, 112, 115-118, 123, 124, 127, 128, 130, 132, 134, 136-139, 143, 144, 149, 150, 152-154, 156-159, 163, 165, 167, 169, 174, 176, 181, 182, 188-193, 195, 196, 200, 201, 203, 210, 211, 213-218, 220, 221, 223, 224, 227-230, 233-238, 240, 241, 244, 245, 248-253, 255, 256, 258-260, 263-266, 269-272, 275-278, 285-287, 290-292, 295, 296, 298, 299, 302, 304-310, 316, 324-326, 329, 330, 332, 333, 338-341, 343, 344, 347, 348, 351, 353, 356, 358, 361-365, 367, 371-373, 377-380, 382, 384, 386, 387, 389, 390, 392, 397, 402, 404-406, 408, 411, 412, 414-417, 419, 420, 422-424, 426, 428-430, 433, 434, 438, 451-453].

2. Subgroup analysis included 220 RCTs involving 10,811 patients in which the agents were administered during surgery [1-6, 12, 13, 16-18, 21, 24, 25, 27, 29, 30, 32, 34-36, 40, 42-44, 48, 53-56, 58, 59, 61, 64, 68, 71, 73, 79-81, 84-86, 99, 102, 103, 105, 107-109, 111, 112, 115-118, 123, 124, 128, 130, 132, 134, 136-139, 143, 144, 149, 150, 153, 154, 156-158, 163, 165, 167, 174, 176, 182, 188-193, 195, 196, 200, 201, 203, 210, 211, 213-218, 221, 223, 224, 227-230, 233-235, 237, 238, 240, 241, 244, 245, 248-251, 253, 255, 256, 258-260, 263-266, 269-272, 275-278, 285-287, 290, 291, 295, 296, 298, 299, 302, 304-309, 316, 324, 326, 329-333, 338-341, 343, 344, 347, 348, 351, 353, 356, 358, 361-365, 367, 371-373, 377-380, 382, 384, 386, 387, 389, 390, 392, 397, 402, 404-406, 411, 412, 414-420, 422-424, 426, 428-430, 433, 434, 438, 451] .

3. Subgroup analysis included 46 RCTs involving a total of 1830 children [13, 29, 32, 48, 58, 59, 64, 80, 84-86, 123, 138, 139, 153, 174, 176, 191, 213, 214, 224, 249, 252, 253, 258, 285, 306, 309, 310, 324, 333, 339, 340, 348, 351, 353, 358, 361, 363, 378, 392, 397, 419, 429, 434]

Nausea Analyses

1. Network meta-analysis for nausea included 195 RCTs with a total of 24,230 patients [1-6, 8, 9, 11, 12, 15-18, 21, 24, 25, 27, 30, 34-36, 40, 44, 53-56, 61, 67, 68, 70, 71, 73, 80, 82, 93, 99-103, 105, 107-109, 111, 112, 115-118, 127, 130-132, 134, 136, 137, 143, 144, 149, 156-159, 163, 165, 167-169, 172, 173, 176, 178, 180-182, 188, 189, 192, 193, 195, 196, 198, 200, 201, 203, 210, 211, 215-217, 220, 221, 227-229, 236-238, 240, 241, 243, 245, 248-252, 255, 259, 260, 264-266, 270-272, 275-278, 284, 287-290, 292, 295, 296, 298, 299, 302, 304-308, 316, 318, 321, 324, 326, 328-330, 334, 338, 339, 341, 343, 347, 356, 360-362, 364, 365, 367, 372, 373, 377, 379, 380, 382-384, 386, 387, 389, 390, 402, 404-406, 408, 412, 414-418, 420, 422-424, 426, 428, 430-432, 451].

2. Subgroup analysis included 175 RCTs involving 21844 patients in which the agents were administered during surgery) [1-6, 11, 12, 15-18, 21, 24, 25, 27, 30, 34-36, 40, 44, 53-56, 61, 68, 71, 73, 80, 99, 102, 103, 105, 107-109, 111, 112, 115-118, 130-132, 134, 136, 137, 143, 144, 149, 156-158, 163, 165, 167, 172, 173, 176, 178, 180, 182, 188, 189, 192, 193, 195, 196, 198, 200, 201, 203, 210, 211, 215-217, 221, 227-229, 236, 238, 240, 241, 244, 245, 248-251, 255, 259, 260, 264-266, 270-272, 275-278, 284, 287-290, 295, 296, 298, 299, 302, 304-308, 316, 318, 321, 324, 326, 328-330, 334, 338, 339, 341, 343,

347, 356, 360, 362, 364, 365, 367, 372, 373, 377, 379, 380, 382-384, 386, 387, 389, 390, 402, 404-406, 412, 414-417, 420, 422-424, 426, 428, 430-432, 451].

3. Subgroup analysis included 11 RCTs involving a total of 1326 children [80, 176, 178, 249, 306, 324, 334, 339, 360].

<u>PONV Analyses</u>

1. Network meta-analysis for vomiting included 125 RCTs, with a total of 16,667 patients [3-6, 8, 9, 11, 12, 16-18, 23, 32, 33, 37, 40, 41, 44, 60, 67, 68, 71, 99-103, 109-112, 115-117, 127, 130, 132, 134, 136, 137, 139, 143, 144, 158-160, 162, 163, 165, 172, 173, 175, 176, 178-180, 182, 187-189, 192, 197, 198, 209, 211, 215, 217, 227, 228, 230, 234, 245, 250, 252, 254-256, 259, 271, 272, 275, 276, 279, 287, 290, 291, 295, 296, 298, 299, 305, 313, 317-320, 328, 334, 336, 337, 339, 343, 360-362, 367, 369, 370, 375, 379, 383, 385, 388, 390, 406, 409, 410, 412, 418, 423, 428, 430-432]

2. Subgroup analysis included 116 RCTs involving 12,415 patients in which the agents were administered during surgery) [3-6, 9, 11, 12, 15-18, 23, 32, 33, 37, 40, 41, 43, 44, 60, 68, 71, 99, 102, 103, 109-112, 115-117, 130, 132, 134, 136, 137, 139, 143, 144, 158, 160, 162, 163, 165, 172, 173, 175-177, 179, 180, 182, 187-189, 192, 197, 198, 211, 215, 217, 227, 228, 230, 234, 245, 250, 254-256, 259, 271, 272, 275, 276, 279, 287, 290, 291, 295, 296, 298, 299, 305, 313, 316, 318-320, 328, 334, 336, 337, 339, 343, 360, 362, 367, 369, 370, 375, 379, 383, 385, 388, 390, 406, 409, 410, 412, 423, 428, 430-432].

3. Subgroup analysis included 14 RCTs involving a total of 2394 children [12, 109, 110, 117, 245, 252, 259, 305, 320, 360, 361, 390, 406, 409].

References

1. Abouleish EI, Rashid S, Haque S, Giezentanner A, Joynton P, Chuang AZ. Ondansetron versus placebo for the control of nausea and vomiting during Caesarean section under spinal anaesthesia. *Anaesthesia.* 1999; 54(5):479-482.
2. Abreu MPd, Vieira JL, Silva IFd, Miziara LEPG, Fófano R. Eficácia do ondansetron, metoclopramida, droperidol e dexametasona na prevenção de náusea e vômito após laparoscopia ginecológica em regime ambulatorial. Estudo comparativo. *Rev Bras Anestesiol.* 2006; 56:08-15.
3. Adducci E, Gorgoglione M, Aceto P, Congedo E, Clemente A, Gualtieri E *et al*. The prophylaxis of postoperative nausea and vomiting after laparoscopic cholecystectomy. *Acta Medica Romana.* 2002; 40(4):331-339.
4. Agarwal A, Bose N, Gaur A, Singh U, Gupta MK, Singh D. Acupressure and ondansetron for postoperative nausea and vomiting after laparoscopic cholecystectomy. *Can J Anaesth.* 2002; 49(6):554-560.
5. Aghadavoudi OA, E. Comparison of granisetron, metoclopramide and gastric decompression for prevention of postoperative nausea and vomiting after fast track cardiac anesthesia. *Journal of Research in Medical Sciences.* 2008; 13(4):166-174.
6. Ahmed AB, Hobbs GJ, Curran JP. Randomized, placebo-controlled trial of combination antiemetic prophylaxis for day-case gynaecological laparoscopic surgery. *Br J Anaesth.* 2000; 85(5):678-682.
7. Ahmed Omran HAS, Abdelhamid Mohamed Nasr D, ezzat ali eid H. Effect of premedication with mirtazapine versus ondansetron on postoperative nausea and vomiting in breast surgery. *Egyptian Journal of Anaesthesia.* 2011; 27(3):135-139.
8. Alexander R, Fennelly M. Comparison of ondansetron, metoclopramide and placebo as premedicants to reduce nausea and vomiting after major surgery. *Anaesthesia.* 1997; 52(7):695-698.
9. Alexander R, Lovell AT, Seingry D, Jones RM. Comparison of ondansetron and droperidol in reducing postoperative nausea and vomiting associated with patient-controlled analgesia. *Anaesthesia.* 1995; 50(12):1086-1088.
10. Aleyasin A, Hanaf S, Saffarieh E, Torkamandi H, Allahyari S, Sadeghi F *et al*. Effcacy of generic granisetron vs Kytril for PONV in major gynecological operations: A randomized, double-blind clinical trial. *Iranian Journal of Pharmaceutical Research.* 2012; 11(4):1059-1064.

11. Alghanem SM, Massad IM, Rashed EM, Abu-Ali HM, Daradkeh SS. Optimization of anesthesia antiemetic measures versus combination therapy using dexamethasone or ondansetron for the prevention of postoperative nausea and vomiting. *Surg Endosc.* 2010; 24(2):353-358.
12. Alkaissi A, Gunnarsson H, Johnsson V, Evertsson K, Ofenbartl L, Kalman S. Disturbing post-operative symptoms are not reduced by prophylactic antiemetic treatment in patients at high risk of post-operative nausea and vomiting. *Acta Anaesthesiol Scand.* 2004; 48(6):761-771.
13. Allen D, Jorgensen C, Sims C. Effect of tropisetron on vomiting during patient-controlled analgesia in children. *Br J Anaesth.* 1999; 83(4):608-610.
14. Alon E, Himmelseher S. Ondansetron in the treatment of postoperative vomiting: a randomized, double-blind comparison with droperidol and metoclopramide. *Anesth Analg.* 1992; 75(4):561-565.
15. Alon E, Kocian R, Nett PC, Koechli OR, Baettig U, Grimaudo V. Tropisetron for the prevention of postoperative nausea and vomiting in women undergoing gynecologic surgery. *Anesth Analg.* 1996; 82(2):338-341.
16. Ansari MM, Siddiqui OA, Haleem S, Varshney R, Akhtar S, Khan FA. Comparison of ramosetron and ondansetron for control of post-operative nausea and vomiting following laparoscopic cholecystectomy. *Indian J Med Sci.* 2010; 64(6):272-280.
17. Aouad MT, Siddik-Sayyid SM, Taha SK, Azar MS, Nasr VG, Hakki MA *et al*. Haloperidol vs. ondansetron for the prevention of postoperative nausea and vomiting following gynaecological surgery. *Eur J Anaesthesiol.* 2007; 24(2):171-178.
18. Apfel CC, Korttila K, Abdalla M, Kerger H, Turan A, Vedder I *et al*. A factorial trial of six interventions for the prevention of postoperative nausea and vomiting. *N Engl J Med.* 2004; 350(24):2441-2451.
19. Arcioni R, della Rocca M, Romano S, Romano R, Pietropaoli P, Gasparetto A. Ondansetron inhibits the analgesic effects of tramadol: a possible 5-HT(3) spinal receptor involvement in acute pain in humans. *Anesth Analg.* 2002; 94(6):1553-1557, table of contents.
20. Argiriadou H, Papaziogas B, Pavlidis T, Parlapani A, Georgiou M, Papagiannopoulou P *et al*. Tropisetron vs ondansetron for prevention of postoperative nausea and vomiting after laparoscopic cholecystectomy: a randomized double-blind, placebo-controlled study. *Surg Endosc.* 2002; 16(7):1087-1090.
21. Ascaso FJ, Ayala I, Carbonell P, Castro FJ, Palomar A. Prophylactic intravenous ondansetron in patients undergoing cataract extraction under general anesthesia. *Ophthalmologica.* 1997; 211(5):292-295.
22. Bach-Styles T, Martin-Sheridan D, Hughes C, Kaufman S. Comparison of ondansetron, metoclopramide, and placebo in the prevention of postoperative emesis in children undergoing ophthalmic surgery. *CRNA.* 1997; 8(4):152-156.
23. Bacic A, Rumboldt Z, Gluncic I, Buklijas J. The impact of the menstrual cycle and ondansetron on postoperative nausea and vomiting. *Int J Clin Pharmacol Res.* 1998; 18(4):153-158.
24. Bajwa SS, Bajwa SK, Kaur J, Sharma V, Singh A, Singh A *et al*. Palonosetron: A novel approach to control postoperative nausea and vomiting in day care surgery. *Saudi J Anaesth.* 2011; 5(1):19-24.
25. Balki M, Kasodekar S, Dhumne S, Carvalho JC. Prophylactic [corrected] granisetron does not prevent postdelivery nausea and vomiting during elective cesarean delivery under spinal anesthesia. *Anesth Analg.* 2007; 104(3):679-683.
26. Bano F, Zafar S, Aftab S, Haider S. Dexamethasone plus ondansetron for prevention of postoperative nausea and vomiting in patients undergoing laparoscopic cholecystectomy: a comparison with dexamethasone alone. *J Coll Physicians Surg Pak.* 2008; 18(5):265-269.
27. Barbone G, Galizia M, Dambruoso P, Fiore T. Ondansetron vs Dolasetron in the prophylaxis of post-operative nausea and vomiting (PONV) following remifentanil infusion. *Acta Anaesthesiologica Italica.* 2004; 55(2):132-142.
28. Barbosa MV, Nahas FX, Ferreira LM, Farah AB, Bariani RL, Joao BB. Ondansetron for the prevention of postoperative nausea and vomiting: which is the best dosage for aesthetic plastic surgery? *Aesthetic Plast Surg.* 2004; 28(1):33-36.
29. Barst SM, Leiderman JU, Markowitz A, Rosen AM, Abramson AL, Bienkowski RS. Ondansetron with propofol reduces the incidence of emesis in children following tonsillectomy. *Can J Anaesth.* 1999; 46(4):359-362.
30. Basu A, Saha D, Hembrom BP, Roy A, Naaz A. Comparison of palanosetron, granisetron and ondansetron as anti-emetics for prevention of postoperative nausea and vomiting in patients undergoing middle ear surgery. *J Indian Med Assoc.* 2011; 109(5):327-329.
31. Bestas A, Önal SA, Bayar MK, Yildirim A, Aygen E. Effects of Ondansetron and Granisetron on Postoperative Nausea and Vomiting in Adult Patients Undergoing Laparoscopic Cholecystectomy: A Randomized, Double-Blind, Placebo-Controlled Clinical Trial. *Curr Ther Res Clin Exp.* 2007; 68(5):303-312.
32. Bhardwaj N, Bala I, Kaur C, Chari P. Comparison of ondansetron with ondansetron plus dexamethasone for antiemetic prophylaxis in children undergoing strabismus surgery. *J Pediatr Ophthalmol Strabismus.* 2004; 41(2):100-104.

33. Bharti N, Shende D. Comparison of anti-emetic effects of ondansetron and low-dose droperidol in pediatric strabismus surgery. *J Pediatr Ophthalmol Strabismus.* 2003; 40(1):23-26.
34. Bhatia N, Katyal S, Grewal A, Kaul T. Antiemetic prophylaxis with granisetron, ondansetron and metoclopramide in ambulatory gynaecological laparoscopic surgery: A comparison. *Journal of Anaesthesiology Clinical Pharmacology.* 2008; 24(3):303-306.
35. Bhatnagar S, Gupta D, Mishra S, Srikanti M, Singh M, Arora R. Preemptive antiemesis in patients undergoing modified radical mastectomy: oral granisetron versus oral ondansetron in a double-blind, randomized, controlled study. *J Clin Anesth.* 2007; 19(7):512-516.
36. Bhattacharjee DP, Dawn S, Nayak S, Roy PR, Acharya A, Dey R. A comparative study between palonosetron and granisetron to prevent postoperative nausea and vomiting after laparoscopic cholecystectomy. *J Anaesthesiol Clin Pharmacol.* 2010; 26(4):480-483.
37. Bhattarai B, Shrestha S, Singh J. Comparison of ondansetron and combination of ondansetron and dexamethasone as a prophylaxis for postoperative nausea and vomiting in adults undergoing elective laparoscopic surgery. *J Emerg Trauma Shock.* 2011; 4(2):168-172.
38. Bicer C, Aksu R, Ulgey A, Madenoglu H, Dogan H, Yildiz K *et al*. Different doses of palonosetron for the prevention of postoperative nausea and vomiting in children undergoing strabismus surgery. *Drugs R D.* 2011; 11(1):29-36.
39. Biedler A, Wermelt J, Kunitz O, Muller A, Wilhelm W, Dethling J *et al*. A risk adapted approach reduces the overall institutional incidence of postoperative nausea and vomiting. *Can J Anaesth.* 2004; 51(1):13-19.
40. Bilgin TE, Birbicer H, Ozer Z, Doruk N, Tok E, Oral U. A comparative study of the antiemetic efficacy of dexamethasone, ondansetron, and metoclopramide in patients undergoing gynecological surgery. *Med Sci Monit.* 2010; 16(7):CR336-341.
41. Binstock W, Rubin R, Bachman C, Kahana M, McDade W, Lynch JP. The effect of premedication with OTFC, with or without ondansetron, on postoperative agitation, and nausea and vomiting in pediatric ambulatory patients. *Paediatr Anaesth.* 2004; 14(9):759-767.
42. Birmingham SD, Mecklenburg BW, Lujan E, Dacanay RG, Boyle PK, Green R. Dolasetron versus ondansetron as single-agent prophylaxis for patients at increased risk for postoperative nausea and vomiting: a prospective, double-blind, randomized trial. *Mil Med.* 2006; 171(9):913-916.
43. Biswas BN, Rudra A. Comparison of granisetron and granisetron plus dexamethasone for the prevention of postoperative nausea and vomiting after laparoscopic cholecystectomy. *Acta Anaesthesiol Scand.* 2003; 47(1):79-83.
44. Biswas BN, Rudra A, Mandal SK. Comparison of ondansetron, dexamethasone, ondansetron plus dexamethasone and placebo in the prevention of nausea and vomiting after laparoscopic tubal ligation. *J Indian Med Assoc.* 2003; 101(11):638, 640, 642.
45. Bodner M, White PF. Antiemetic efficacy of ondansetron after outpatient laparoscopy. *Anesth Analg.* 1991; 73(3):250-254.
46. Bolton CM, Myles PS, Carlin JB, Nolan T. Randomized, double-blind study comparing the efficacy of moderate-dose metoclopramide and ondansetron for the prophylactic control of postoperative vomiting in children after tonsillectomy. *Br J Anaesth.* 2007; 99(5):699-703.
47. Boonmak P, Boonmak S, Bunsaengjaroen P, Srichaipanha S, Poomsawat S, Nonlhaopol D. Antiemetic effect of ondansetron 0.2 mg mL-1 in PCA morphine solution. *Eur J Anaesthesiol.* 2007; 24(8):664-667.
48. Bowhay AR, May HA, Rudnicka AR, Booker PD. A randomized controlled trial of the antiemetic effect of three doses of ondansetron after strabismus surgery in children. *Paediatr Anaesth.* 2001; 11(2):215-221.
49. Bridges JD, Nettle CB, Dugirrala VJ, Suda KJ, Garey KW. Low-dose granisetron for the prevention of postoperative nausea and vomiting. *6.* 2006; 3(223-229).
50. Broome IJ, Robb HM, Raj N, Girgis Y, Wardall GJ. The use of tramadol following day--case oral surgery. *Anaesthesia.* 1999; 54(3):289-292.
51. Browning BA, Fort CA, Kemp KD, Shimata MF, Strube MD. Ondansetron versus dolasetron: a comparison study in the prevention of postoperative nausea and vomiting in patients undergoing gynecological procedures. *AANA J.* 2004; 72(2):129-132.
52. Browning RM, Fellingham WH, O'Loughlin EJ, Brown NA, Paech MJ. Prophylactic ondansetron does not prevent shivering or decrease shivering severity during cesarean delivery under combined spinal epidural anesthesia: a randomized trial. *Reg Anesth Pain Med.* 2013; 38(1):39-43.
53. Bugedo G, Gonzalez J, Asenjo C, De la Cuadra JC, Gajardo A, Castillo L *et al*. Ondansetron and droperidol in the prevention of postoperative nausea and vomiting. *Br J Anaesth.* 1999; 83(5):813-814.
54. Burmeister MA, Standl TG, Wintruff M, Brauer P, Blanc I, Schulte am Esch J. Dolasetron prophylaxis reduces nausea and postanaesthesia recovery time after remifentanil infusion during monitored anaesthesia care for extracorporeal shock wave lithotripsy. *Br J Anaesth.* 2003; 90(2):194-198.
55. Campbell C, Miller DD. Failure of ondansetron to control postoperative nausea and vomiting in ambulatory surgical patients. *Am J Anesthesiol.* 1995; 22(2):81-86.

56. Candiotti KA, Kovac AL, Melson TI, Clerici G, Joo Gan T, Palonosetron 04-06 Study G. A randomized, double-blind study to evaluate the efficacy and safety of three different doses of palonosetron versus placebo for preventing postoperative nausea and vomiting. *Anesth Analg.* 2008; 107(2):445-451.
57. Candiotti KA, Nhuch F, Kamat A, Deepika K, Arheart KL, Birnbach DJ *et al*. Granisetron versus ondansetron treatment for breakthrough postoperative nausea and vomiting after prophylactic ondansetron failure: a pilot study. *Anesth Analg.* 2007; 104(6):1370-1373, table of contents.
58. Carnahan D, Dato K, Hartsuff J. The safety and efficacy of granisetron in postoperative vomiting in pediatric patients undergoing tonsillectomy. *AANA J.* 1997; 65(2):154-159.
59. Celiker V, Celebi N, Canbay O, Basgul E, Aypar U. Minimum effective dose of dexamethasone after tonsillectomy. *Paediatr Anaesth.* 2004; 14(8):666-669.
60. Chan MT, Choi KC, Gin T, Chui PT, Short TG, Yuen PM *et al*. The additive interactions between ondansetron and droperidol for preventing postoperative nausea and vomiting. *Anesth Analg.* 2006; 103(5):1155-1162.
61. Charuluxananan S, Kyokong O, Somboonviboon W, Narasethakamol A, Promlok P. Nalbuphine versus ondansetron for prevention of intrathecal morphine-induced pruritus after cesarean delivery. *Anesth Analg.* 2003; 96(6):1789-1793.
62. Charuluxananan S, Somboonviboon W, Kyokong O, Nimcharoendee K. Ondansetron for treatment of intrathecal morphine-induced pruritus after cesarean delivery. *Reg Anesth Pain Med.* 2000; 25(5):535-539.
63. Chen JJ, Frame DG, White TJ. Efficacy of ondansetron and prochlorperazine for the prevention of postoperative nausea and vomiting after total hip replacement or total knee replacement procedures: a randomized, double-blind, comparative trial. *Arch Intern Med.* 1998; 158(19):2124-2128.
64. Chen LK, Fan SZ, Huang CH, Chao A, Cherng YG, Chen CL *et al*. Effects of ondansetron on postoperative emesis in Chinese children. *Acta Anaesthesiol Sin.* 1998; 36(2):87-91.
65. Chen PP, Chui PT, Gin T. Comparison of ondansetron and metoclopramide for the prevention of post-operative nausea and vomiting after major gynaecological surgery. *Eur J Anaesthesiol.* 1996; 13(5):485-491.
66. Chen PT, Liaw CC. Intravenous ondansetron plus intravenous dexamethasone with different ondansetron dosing schedules during multiple cycles of cisplatin-based chemotherapy. *Chang Gung Med J.* 2008; 31(2):167-174.
67. Cherian VT, Smith I. Prophylactic ondansetron does not improve patient satisfaction in women using PCA after Caesarean section. *Br J Anaesth.* 2001; 87(3):502-504.
68. Choi DK, Chin JH, Lee EH, Lim OB, Chung CH, Ro YJ *et al*. Prophylactic control of post-operative nausea and vomiting using ondansetron and ramosetron after cardiac surgery. *Acta Anaesthesiol Scand.* 2010; 54(8):962-969.
69. Choi YS, Shim JK, Ahn SH, Kwak YL. Efficacy comparison of ramosetron with ondansetron on preventing nausea and vomiting in high-risk patients following spine surgery with a single bolus of dexamethasone as an adjunct. *Korean J Anesthesiol.* 2012; 62(6):543-547.
70. Choi YS, Shim JK, Yoon do H, Jeon DH, Lee JY, Kwak YL. Effect of ramosetron on patient-controlled analgesia related nausea and vomiting after spine surgery in highly susceptible patients: comparison with ondansetron. *Spine (Phila Pa 1976).* 2008; 33(17):E602-606.
71. Cholwill JM, Wright W, Hobbs GJ, Curran J. Comparison of ondansetron and cyclizine for prevention of nausea and vomiting after day-case gynaecological laparoscopy. *Br J Anaesth.* 1999; 83(4):611-614.
72. Cohen IT, Joffe D, Hummer K, Soluri A. Ondansetron oral disintegrating tablets: acceptability and efficacy in children undergoing adenotonsillectomy. *Anesth Analg.* 2005; 101(1):59-63, table of contents.
73. Coloma M, White PF, Markowitz SD, Whitten CW, Macaluso AR, Berrisford SB *et al*. Dexamethasone in Combination with Dolasetron for Prophylaxis in the Ambulatory Setting: Effect on Outcome after Laparoscopic Cholecystectomy. *Anesthesiology.* 2002; 96(6):1346-1350.
74. Coloma M, White PF, Ogunnaike BO, Markowitz SD, Brown PM, Lee AQ *et al*. Comparison of acustimulation and ondansetron for the treatment of established postoperative nausea and vomiting. *Anesthesiology.* 2002; 97(6):1387-1392.
75. Contreras-Dominguez V, Carbonell-Bellolio P. Prophylactic antiemetic therapy for acute abdominal surgery. A comparative study of droperidol, metoclopramide, tropisetron, granisetron and dexamethasone. *Rev Bras Anestesiol.* 2008; 58(1):35-44.
76. Cruz NI, Portilla P, Vela RE. Timing of ondansetron administration to prevent postoperative nausea and vomiting. *P R Health Sci J.* 2008; 27(1):43-47.
77. Dabbous A, Itani M, Kawas N, Karam V, Aouad M, Baraka A *et al*. Post-laparoscopic vomiting in females versus males: comparison of prophylactic antiemetic action of ondansetron versus metoclopramide. *JSLS.* 1998; 2(3):273-276.
78. Dabbous A, Khoury SJ, Chehab IR, Bartelmaos T, Khoury G. Ondansetron versus dehydrobenzoperidol and metoclopramide for management of postoperative nausea in laparoscopic surgery patients. *JSLS.* 2001; 5(2):139-142.

79. Dabbous AS, Jabbour-Khoury SI, Nasr VG, Moussa AA, Zbeidy RA, Khouzam NE *et al*. Dexamethasone with either granisetron or ondansetron for postoperative nausea and vomiting in laparoscopic surgery. *Middle East J Anesthesiol.* 2010; 20(4):565-570.
80. Daftary S, Jagtap SR, Saksena S. Intravenous Ondansetron in prevention of PONV following tonsillectomy under ether anaesthesia. *Journal of Anaesthesiology Clinical Pharmacology.* 1998; 14(1):51-54.
81. D'Angelo R, Philip B, Gan TJ, Kovac A, Hantler C, Doblar D *et al*. A randomized, double-blind, close-ranging, pilot study of intravenous granisetron in the prevention of postoperative nausea and vomiting in patients abdominal hysterectomy. *Eur J Anaesthesiol.* 2005; 22(10):774-779.
82. Davies PR, Warwick P, O'Connor M. Antiemetic efficacy of ondansetron with patient-controlled analgesia. *Anaesthesia.* 1996; 51(9):880-882.
83. Davis A, Krige S, Moyes D. A double-blind randomized prospective study comparing ondansetron with droperidol in the prevention of emesis following strabismus surgery. *Anaesth Intensive Care.* 1995; 23(4):438-443.
84. Davis PJ, Fertal KM, Boretsky KR, Fedel GM, Ingram MD, Woelfel SK *et al*. The effects of oral ondansetron disintegrating tablets for prevention of at-home emesis in pediatric patients after ear-nose-throat surgery. *Anesth Analg.* 2008; 106(4):1117-1121, table of contents.
85. Davis PJ, McGowan FX, Jr., Landsman I, Maloney K, Hoffmann P. Effect of antiemetic therapy on recovery and hospital discharge time. A double-blind assessment of ondansetron, droperidol, and placebo in pediatric patients undergoing ambulatory surgery. *Anesthesiology.* 1995; 83(5):956-960.
86. de Orange FA, Marques J, Flores M, Borges PS. Dexamethasone versus ondansetron in combination with dexamethasone for the prophylaxis of postoperative vomiting in pediatric outpatients: a double-blind, randomized, placebo-controlled clinical trial. *Paediatr Anaesth.* 2012; 22(9):890-896.
87. Demiraran Y, Ozdemir I, Kocaman B, Hayit F, Demirci F. Comparison of costs and efficacy of intravenous and orally disintegrating ondansetron tablet as a prophylactic antiemetic therapy in major gynecologic operations. *Journal of the Turkish German Gynecology Association Artemis.* 2005; 6(2):134-138.
88. Dershwitz M, Conant JA, Chang Y, Rosow CE, Connors PM. A randomized, double-blind, dose-response study of ondansetron in the prevention of postoperative nausea and vomiting. *J Clin Anesth.* 1998; 10(4):314-320.
89. Desilva PH, Darvish AH, McDonald SM, Cronin MK, Clark K. The efficacy of prophylactic ondansetron, droperidol, perphenazine, and metoclopramide in the prevention of nausea and vomiting after major gynecologic surgery. *Anesth Analg.* 1995; 81(1):139-143.
90. Diemunsch P, Conseiller C, Clyti N, Mamet JP. Ondansetron compared with metoclopramide in the treatment of established postoperative nausea and vomiting. The French Ondansetron Study Group. *Br J Anaesth.* 1997; 79(3):322-326.
91. Diemunsch P, D'Hollander A, Paxton L, Schoeffler P, Wessel P, Nave S *et al*. Intravenous dolasetron mesilate in the prevention of postoperative nausea and vomiting in females undergoing gynecological surgery. *J Clin Anesth.* 1997; 9(5):365-373.
92. Diemunsch P, Gan TJ, Philip BK, Girao MJ, Eberhart L, Irwin MG *et al*. Single-dose aprepitant vs ondansetron for the prevention of postoperative nausea and vomiting: a randomized, double-blind phase III trial in patients undergoing open abdominal surgery. *Br J Anaesth.* 2007; 99(2):202-211.
93. Diemunsch P, Korttila K, Leeser J, Helmers JH, Wilkey B, Nave S *et al*. Oral dolasetron mesylate for prevention of postoperative nausea and vomiting: a multicenter, double-blind, placebo-controlled study. The Oral Dolasetron PONV Prevention Study Group. *J Clin Anesth.* 1998; 10(2):145-152.
94. Diemunsch P, Leeser J, Feiss P, D'Hollander A, Bradburn BG, Paxton D *et al*. Intravenous dolasetron mesilate ameliorates postoperative nausea and vomiting. *Can J Anaesth.* 1997; 44(2):173-181.
95. Doe EA, Jones P, O'Hara MA. A comparison of prophylactic ondansetron hydrochloride and droperidol for strabismus repair in adults. *J Pediatr Ophthalmol Strabismus.* 1998; 35(5):264-269; 286-267.
96. Dresner M, Dean S, Lumb A, Bellamy M. High-dose ondansetron regimen vs droperidol for morphine patient-controlled analgesia. *Br J Anaesth.* 1998; 81(3):384-386.
97. D'Souza N, Swami M, Bhagwat S. Comparative study of dexamethasone and ondansetron for prophylaxis of postoperative nausea and vomiting in laparoscopic gynecologic surgery. *Int J Gynaecol Obstet.* 2011; 113(2):124-127.
98. Du Pen S, Scuderi P, Wetchler B, Sung YF, Mingus M, Clayborn L *et al*. Ondansetron in the treatment of postoperative nausea and vomiting in ambulatory outpatients: a dose-comparative, stratified, multicentre study. *Eur J Anaesthesiol Suppl.* 1992; 6:55-62.
99. Dua N, Bhatnagar S, Mishra S, Singhal AK. Granisetron and ondansetron for prevention of nausea and vomiting in patients undergoing modified radical mastectomy. *Anaesth Intensive Care.* 2004; 32(6):761-764.
100. Dupeyron JP, Conseiller C, Levarlet M, Hemmingsen C, Schoeffler P, Pedersen FM *et al*. The effect of oral ondansetron in the prevention of postoperative nausea and vomiting after major gynaecological surgery performed under general anaesthesia. *Anaesthesia.* 1993; 48(3):214-218.
101. Eberhart LH, Buning EK, Folz B, Maybauer DM, Kastner M, Kalder M *et al*. Anti-emetic prophylaxis with oral tropisetron and/or dexamethasone. *Eur J Clin Invest.* 2006; 36(8):580-587.

102. Eberhart LH, Morin AM, Hoerle S, Wulf H, Geldner G. Droperidol and dolasetron alone or in combination for prevention of postoperative nausea and vomiting after vitrectomy. *Ophthalmology.* 2004; 111(8):1569-1575.
103. Eidi M, Kolahdouzan K, Hosseinzadeh H, Tabaqi R. A comparison of preoperative ondansetron and dexamethasone in the prevention of post-tympanoplasty nausea and vomiting. *Iran J Med Sci.* 2012; 37(3):166-172.
104. Ekinci O, Malat I, Isitmangil G, Aydin N. A randomized comparison of droperidol, metoclopramide, tropisetron, and ondansetron for the prevention of postoperative nausea and vomiting. *Gynecol Obstet Invest.* 2011; 71(1):59-65.
105. El-Deeb A, Abd el motlb E. Prophylactic multimodal antiemetic in women undergoing cesarean section under spinal anesthesia. *Egyptian Journal of Anaesthesia.* 2011; 27(2):107-111.
106. El-Deeb A, Ali Y, Rashdy H. Evaluation of combination antiemetic prophylaxis in high risk emetogenic patients undergoing thyroid surgery: A randomized double-blind study. *Egyptian Journal of Anaesthesia.* 2011; 27(4):203-206.
107. El-Deeb AM, Ahmady MS. Effect of acupuncture on nausea and/or vomiting during and after cesarean section in comparison with ondansetron. *J Anesth.* 2011; 25(5):698-703.
108. Elhakim M, Ghalaab M, Soliman M. Effects of odansetron and balanced analgesia on postoperative nausea and vomiting in laparoscopic surgery. *Acta Anaesthesiologica Italica.* 1995; 46(Suppl):23-28.
109. Elhakim M, Nafie M, Mahmoud K, Atef A. Dexamethasone 8 mg in combination with ondansetron 4 mg appears to be the optimal dose for the prevention of nausea and vomiting after laparoscopic cholecystectomy. *Can J Anaesth.* 2002; 49(9):922-926.
110. Ercelen O, Celiker V, Celebioglu B, Basgul E, Aypar U. Prevention from postoperative nausea and vomiting after strabismus surgery in children. *Acta Anaesthesiologica Italica.* 1996; 47(3):211-214.
111. Erhan Y, Erhan E, Aydede H, Yumus O, Yentur A. Ondansetron, granisetron, and dexamethasone compared for the prevention of postoperative nausea and vomiting in patients undergoing laparoscopic cholecystectomy : A randomized placebo-controlled study. *Surg Endosc.* 2008; 22(6):1487-1492.
112. Eriksson H, Korttila K. Recovery profile after desflurane with or without ondansetron compared with propofol in patients undergoing outpatient gynecological laparoscopy. *Anesth Analg.* 1996; 82(3):533-538.
113. Eryilmaz T, Sencan A, Camgoz N, Ak B, Yavuzer R. A challenging problem that concerns the aesthetic surgeon: postoperative nausea and vomiting. *Ann Plast Surg.* 2008; 61(5):489-491.
114. Kim EJ, Ko JS, Kim CS, Lee SM, Choi DH. Combination of antiemetics for the prevention of postoperative nausea and vomiting in high-risk patients. *J Korean Med Sci.* 2007; 22(5):878-882.
115. van den Berg AA. The prophylactic antiemetic efficacy of prochlorperazine and ondansetron in nasal septal surgery: a randomized double-blind comparison. *Anaesth Intensive Care.* 1996; 24(5):538-545.
116. van den Berg AA. Comparison of ondansetron and prochlorperazine for the prevention of nausea and vomiting after adenotonsillectomy. *Br J Anaesth.* 1996; 76(3):449-451.
117. van den Berg AA. A comparison of ondansetron and prochlorperazine for the prevention of nausea and vomiting after tympanoplasty. *Can J Anaesth.* 1996; 43(9):939-945.
118. Fabling JM, Gan TJ, El-Moalem HE, Warner DS, Borel CO. A randomized, double-blinded comparison of ondansetron, droperidol, and placebo for prevention of postoperative nausea and vomiting after supratentorial craniotomy. *Anesth Analg.* 2000; 91(2):358-361.
119. Fabling JM, Gan TJ, El-Moalem HE, Warner DS, Borel CO. A randomized, double-blind comparison of ondansetron versus placebo for prevention of nausea and vomiting after infratentorial craniotomy. *J Neurosurg Anesthesiol.* 2002; 14(2):102-107.
120. Farouk S. Ondansetron added to lidocaine for intravenous regional anaesthesia. *Eur J Anaesthesiol.* 2009; 26(12):1032-1036.
121. Feng PH, Chu KS, Lu IC, Shieh JP, Tzeng JI, Ho ST *et al*. Haloperidol plus ondansetron prevents postoperative nausea and vomiting in patients undergoing laparoscopic cholecystectomy. *Acta Anaesthesiol Taiwan.* 2009; 47(1):3-9.
122. Flynn BC, Nemergut EC. Postoperative nausea and vomiting and pain after transsphenoidal surgery: a review of 877 patients. *Anesth Analg.* 2006; 103(1):162-167, table of contents.
123. Furst SR, Rodarte A. Prophylactic antiemetic treatment with ondansetron in children undergoing tonsillectomy. *Anesthesiology.* 1994; 81(4):799-803.
124. Furst SR, Sullivan LJ, Soriano SG, McDermott JS, Adelson PD, Rockoff MA. Effects of ondansetron on emesis in the first 24 hours after craniotomy in children. *Anesth Analg.* 1996; 83(2):325-328.
125. Gan TJ, Apfel CC, Kovac A, Philip BK, Singla N, Minkowitz H *et al*. A randomized, double-blind comparison of the NK1 antagonist, aprepitant, versus ondansetron for the prevention of postoperative nausea and vomiting. *Anesth Analg.* 2007; 104(5):1082-1089, tables of contents.
126. Gan TJ, Candiotti KA, Klein SM, Rodriguez Y, Nielsen KC, White WD *et al*. Double-blind comparison of granisetron, promethazine, or a combination of both for the prevention of postoperative nausea and vomiting in females undergoing outpatient laparoscopies. *Can J Anaesth.* 2009; 56(11):829-836.

127. Gan TJ, Collis R, Hetreed M. Double-blind comparison of ondansetron, droperidol and saline in the prevention of postoperative nausea and vomiting. *Br J Anaesth.* 1994; 72(5):544-547.
128. Gan TJ, Coop A, Philip BK. A randomized, double-blind study of granisetron plus dexamethasone versus ondansetron plus dexamethasone to prevent postoperative nausea and vomiting in patients undergoing abdominal hysterectomy. *Anesth Analg.* 2005; 101(5):1323-1329.
129. Gan TJ, Franiak R, Reeves J. Ondansetron orally disintegrating tablet versus placebo for the prevention of postdischarge nausea and vomiting after ambulatory surgery. *Anesth Analg.* 2002; 94(5):1199-1200, table of contents.
130. Gan TJ, Gu J, Singla N, Chung F, Pearman MH, Bergese SD *et al*. Rolapitant for the prevention of postoperative nausea and vomiting: a prospective, double-blinded, placebo-controlled randomized trial. *Anesth Analg.* 2011; 112(4):804-812.
131. Gan TJ, Jiao KR, Zenn M, Georgiade G. A randomized controlled comparison of electro-acupoint stimulation or ondansetron versus placebo for the prevention of postoperative nausea and vomiting. *Anesth Analg.* 2004; 99(4):1070-1075, table of contents.
132. Gandhi R, Aruna J, Ajai K. Ondansetron/dexamethasone for prophylaxis against postoperative nausea and Vomiting. *Journal of Anaesthesiology Clinical Pharmacology.* 2009; 25(3):305-310.
133. Ganem EM, Fabris P, Moro MZ, Castiglia YMM. Eficácia do ondansetron e da alizaprida na prevenção de náusea e vômito em laparoscopia ginecológica. *Rev Bras Anestesiol.* 2001; 51:401-406.
134. Gautam B, Shrestha BR, Lama P, Rai S. Antiemetic prophylaxis against postoperative nausea and vomiting with ondansetron-dexamethasone combination compared to ondansetron or dexamethasone alone for patients undergoing laparoscopic cholecystectomy. *Kathmandu Univ Med J (KUMJ).* 2008; 6(23):319-328.
135. Gesztesi Z, Scuderi PE, White PF, Wright W, Wender RH, D'Angelo R *et al*. Substance P (Neurokinin-1) antagonist prevents postoperative vomiting after abdominal hysterectomy procedures. *Anesthesiology.* 2000; 93(4):931-937.
136. Goksu S, Kocoglu H, Bayazit YA, Yuksek S, Karci Y, Kanlikama M *et al*. Antiemetic effects of granisetron, droperidol and dexamethasone in otologic surgery. *Auris Nasus Larynx.* 2002; 29(3):253-256.
137. Goll V, Akça, O., Greif R, Freitag H, Arkiliç CF, Scheck T *et al*. Ondansetron is no more effective than supplemental intraoperative oxygen for prevention of postoperative nausea and vomiting. , 92(1), 112-117. *Anesth Analg.* 2001; 92(1):112-117.
138. Gombar S, Kaur J, Kumar Gombar K, Dass A, Singh A. Superior anti-emetic efficacy of granisetron-dexamethasone combination in children undergoing middle ear surgery. *Acta Anaesthesiol Scand.* 2007; 51(5):621-624.
139. Goodarzi M. A double blind comparison of droperidol and ondansetron for prevention of emesis in children undergoing orthopaedic surgery. *Paediatr Anaesth.* 1998; 8(4):325-329.
140. Graczyk SG, McKenzie R, Kallar S, Hickok CB, Melson T, Morrill B *et al*. Intravenous dolasetron for the prevention of postoperative nausea and vomiting after outpatient laparoscopic gynecologic surgery. *Anesth Analg.* 1997; 84(2):325-330.
141. Grimsehl K, Whiteside JB, Mackenzie N. Comparison of cyclizine and ondansetron for the prevention of postoperative nausea and vomiting in laparoscopic day-case gynaecological surgery. *Anaesthesia.* 2002; 57(1):61-65.
142. Grond S, Lynch J, Diefenbach C, Altrock K, Lehmann KA. Comparison of ondansetron and droperidol in the prevention of nausea and vomiting after inpatient minor gynecologic surgery. *Anesth Analg.* 1995; 81(3):603-607.
143. Grover VK, Mathew PJ, Hegde H. Efficacy of orally disintegrating ondansetron in preventing postoperative nausea and vomiting after laparoscopic cholecystectomy: a randomised, double-blind placebo controlled study. *Anaesthesia.* 2009; 64(6):595-600.
144. Gulhas N, Erdil FA, Sagir O, Gedik E, Togal T, Begec Z *et al*. Lornoxicam and ondansetron for the prevention of intrathecal fentanyl-induced pruritus. *J Anesth.* 2007; 21(2):159-163.
145. Gunter JB, McAuliffe JJ, Beckman EC, Wittkugel EP, Spaeth JP, Varughese AM. A factorial study of ondansetron, metoclopramide, and dexamethasone for emesis prophylaxis after adenotonsillectomy in children. *Paediatr Anaesth.* 2006; 16(11):1153-1165.
146. Gupta P, Khanna J, Mitramustafi AK, Bhartia VK. Role of pre-operative dexamethasone as prophylaxis for postoperative nausea and vomiting in laparoscopic surgery. *J Minim Access Surg.* 2006; 2(1):12-15.
147. Gupta V, Wakhloo R, Mehta A, Gupta SD. Prophylactic antiemetic therapy with ondansetron, granisetron and metoclopramide in patients undergoing laparoscopic cholecystectomy under GA. *10.* 2008; 2(74-77).
148. Gurler T, Celik N, Totan S, Songur E, Sakarya M. Prophylactic use of ondansetron for emesis after craniofacial operations in children. *J Craniofac Surg.* 1999; 10(1):45-48.
149. Hahm TS, Ko JS, Choi SJ, Gwak MS. Comparison of the prophylactic anti-emetic efficacy of ramosetron and ondansetron in patients at high-risk for postoperative nausea and vomiting after total knee replacement. *Anaesthesia.* 2010; 65(5):500-504.
150. Hamid SK, Selby IR, Sikich N, Lerman J. Vomiting after adenotonsillectomy in children: a comparison of ondansetron, dimenhydrinate, and placebo. *Anesth Analg.* 1998; 86(3):496-500.

151. Han DW, Hong SW, Kwon JY, Lee JW, Kim KJ. Epidural ondansetron is more effective to prevent postoperative pruritus and nausea than intravenous ondansetron in elective cesarean delivery. *Acta Obstet Gynecol Scand.* 2007; 86(6):683-687.
152. Han SH, Lim YJ, Ro YJ, Lee SC, Park YS, Kim YC. Efficacy of prophylactic ondansetron in a patient-controlled analgesia environment. *J Int Med Res.* 2004; 32(2):160-165.
153. Hanaoka K, Toyooka H, Kugimiya T, Ohashi Y, Granisetron Study Group of J. Efficacy of prophylactic intravenous granisetron in postoperative emesis in adults. *J Anesth.* 2004; 18(3):158-165.
154. Harnett MJ, O'Rourke N, Walsh M, Carabuena JM, Segal S. Transdermal scopolamine for prevention of intrathecal morphine-induced nausea and vomiting after cesarean delivery. *Anesth Analg.* 2007; 105(3):764-769.
155. Hartsell T, Long D, Kirsch JR. The efficacy of postoperative ondansetron (Zofran) orally disintegrating tablets for preventing nausea and vomiting after acoustic neuroma surgery. *Anesth Analg.* 2005; 101(5):1492-1496.
156. Helmers JH, Briggs L, Abrahamsson J, Soni J, Moodley J, Forrler M *et al.* A single i.v. dose of ondansetron 8 mg prior to induction of anaesthesia reduces postoperative nausea and vomiting in gynaecological patients. *Can J Anaesth.* 1993; 40(12):1155-1161.
157. Helmy SA. Prophylactic anti-emetic efficacy of ondansetron in laparoscopic cholecystectomy under total intravenous anaesthesia. A randomised, double-blind comparison with droperidol, metoclopramide and placebo. *Anaesthesia.* 1999; 54(3):266-271.
158. Honkavaara P. Effect of ondansetron on nausea and vomiting after middle ear surgery during general anaesthesia. *Br J Anaesth.* 1996; 76(2):316-318.
159. Huh BK, Jung S, White W, Jeon Y. Anti-emetic effect of midazolam added to morphine patient-controlled analgesia after total abdominal hysterectomy. *Anaesth Intensive Care.* 2010; 38(3):481-485.
160. Iatrou CA, Dragoumanis CK, Vogiatzaki TD, Vretzakis GI, Simopoulos CE, Dimitriou VK. Prophylactic intravenous ondansetron and dolasetron in intrathecal morphine-induced pruritus: a randomized, double-blinded, placebo-controlled study. *Anesth Analg.* 2005; 101(5):1516-1520.
161. Isik B, Cekmen N, Arslan M, Ozsoylar O, Kordan AZ, Akcabay M. Comparison of the antiemetic effects of ondansetron and dexamethasone on middle ear surgery. *Saudi Med J.* 2006; 27(5):646-651.
162. Islam MR, Haq MF, Islam MA, Meftahuzzaman SM, Sarkar SC, Rashid H *et al*. Preoperative use of granisetron plus dexamethasone and granisetron alone in prevention of post operative nausea and vomiting in tonsillectomy. *Mymensingh Med J.* 2011; 20(3):386-390.
163. Jain V, Mitra JK, Rath GP, Prabhakar H, Bithal PK, Dash HH. A randomized, double-blinded comparison of ondansetron, granisetron, and placebo for prevention of postoperative nausea and vomiting after supratentorial craniotomy. *J Neurosurg Anesthesiol.* 2009; 21(3):226-230.
164. Janicki PK, Schuler HG, Jarzembowski TM, Rossi M, 2nd. Prevention of postoperative nausea and vomiting with granisetron and dolasetron in relation to CYP2D6 genotype. *Anesth Analg.* 2006; 102(4):1127-1133.
165. Janknegt R, Pinckaers JW, Rohof MH, Ausems ME, Arbouw ME, van der Velden RW *et al*. Double-blind comparative study of droperidol, granisetron and granisetron plus dexamethasone as prophylactic anti-emetic therapy in patients undergoing abdominal, gynaecological, breast or otolaryngological surgery. *Anaesthesia.* 1999; 54(11):1059-1068.
166. Jee YS, Yoon HJ, Jang CH. Prophylactic antiemetic effects in gynecologic patients receiving fentanyl IV-patient controlled analgesia: comparison of combined treatment with ondansetron and dexamethasone with metoclopramide and dexamethasone. *Korean J Anesthesiol.* 2010; 59(5):335-339.
167. Jellish WS, Leonetti JP, Fluder E, Thalji Z. Ondansetron versus droperidol or placebo to prevent nausea and vomiting after otologic surgery. *Otolaryngol Head Neck Surg.* 1998; 118(6):785-789.
168. Jellish WS, Leonetti JP, Sawicki K, Anderson D, Origitano TC. Morphine/ondansetron PCA for postoperative pain, nausea, and vomiting after skull base surgery. *Otolaryngol Head Neck Surg.* 2006; 135(2):175-181.
169. Jellish WS, Owen K, Fluder E, Sawicki K, Sinacore J. Patient-controlled analgesia combined with either ondansetron or ondansetron plus prochlorperazine for control of pain and nausea and vomiting in patients undergoing abdominal surgery. *J Clin Anesth.* 2008; 20(8):594-600.
170. Johns RA, Hanousek J, Montgomery JE. A comparison of cyclizine and granisetron alone and in combination for the prevention of postoperative nausea and vomiting. *Anaesthesia.* 2006; 61(11):1053-1057.
171. Jokela R, Ahonen J, Seitsonen E, Marjakangas P, Korttila K. The influence of ondansetron on the analgesic effect of acetaminophen after laparoscopic hysterectomy. *Clin Pharmacol Ther.* 2010; 87(6):672-678.
172. Jokela R, Koivuranta M, Kangas-Saarela T, Purhonen S, Alahuhta S. Oral ondansetron, tropisetron or metoclopramide to prevent postoperative nausea and vomiting: a comparison in high-risk patients undergoing thyroid or parathyroid surgery. *Acta Anaesthesiol Scand.* 2002; 46(5):519-524.
173. Jokela RM, Kangas-Saarela TA, Valanne JV, Koivuranta MK, Ranta PO, Alahuhta SM. Postoperative nausea and vomiting after sevoflurane with or without ondansetron compared with propofol in female patients undergoing breast surgery. *Anesth Analg.* 2000; 91(5):1062-1065.

174. Kabalak AA, Akcay M, Akcay F, Gogus N. Transcutaneous electrical acupoint stimulation versus ondansetron in the prevention of postoperative vomiting following pediatric tonsillectomy. *J Altern Complement Med.* 2005; 11(3):407-413.
175. Kaki AM, Abd El-Hakeem EE. Prophylaxis of postoperative nausea and vomiting with ondansetron, metoclopramide, or placebo in total intravenous anesthesia patients undergoing laparoscopic cholecystectomy. *Saudi Med J.* 2008; 29(10):1408-1413.
176. Karamanlioglu B, Turan A, Memis D, Sut N. Comparison of oral dolasetron and ondansetron in the prophylaxis of postoperative nausea and vomiting in children. *Eur J Anaesthesiol.* 2003; 20(10):831-835.
177. Kathirvel S, Dash HH, Bhatia A, Subramaniam B, Prakash A, Shenoy S. Effect of prophylactic ondansetron on postoperative nausea and vomiting after elective craniotomy. *J Neurosurg Anesthesiol.* 2001; 13(3):207-212.
178. Kathirvel S, Shende D, Madan R. Comparison of anti-emetic effects of ondansetron, metoclopromide or a combination of both in children undergoing surgery for strabismus. *Eur J Anaesthesiol.* 1999; 16(11):761-765.
179. Kaufmann MA, Rosow C, Schnieper P, Schneider M. Prophylactic antiemetic therapy with patient-controlled analgesia: a double-blind, placebo-controlled comparison of droperidol, metoclopramide, and tropisetron. *Anesth Analg.* 1994; 78(5):988-994.
180. Kaul HL, Rao U, Mandal NG, Rahman A. Comparative evaluation of single dose oral Ondansetron and Metoclopramide in a placebo controlled study for prevention of postoperative nausea and vomiting. *Journal of Anaesthesiology Clinical Pharmacology.* 1996; 12(1):27-30.
181. Kenny GN, Oates JD, Leeser J, Rowbotham DJ, Lip H, Rust M *et al*. Efficacy of orally administered ondansetron in the prevention of postoperative nausea and vomiting: a dose ranging study. *Br J Anaesth.* 1992; 68(5):466-470.
182. Khalil S, Philbrook L, Rabb M, Wells L, Aves T, Villanueva G *et al*. Ondansetron/promethazine combination or promethazine alone reduces nausea and vomiting after middle ear surgery. *J Clin Anesth.* 1999; 11(7):596-600.
183. Khalil S, Rodarte A, Weldon BC, Weinstein M, Grunwald Z, Ginsberg B *et al*. Intravenous ondansetron in established postoperative emesis in children. S3A-381 Study Group. *Anesthesiology.* 1996; 85(2):270-276.
184. Khalil SN, Kataria B, Pearson K, Conahan T, Kallar S, Zahl K *et al*. Ondansetron prevents postoperative nausea and vomiting in women outpatients. *Anesth Analg.* 1994; 79(5):845-851.
185. Khalil SN, Roth AG, Cohen IT, Simhi E, Ansermino JM, Bolos ME *et al*. A double-blind comparison of intravenous ondansetron and placebo for preventing postoperative emesis in 1- to 24-month-old pediatric patients after surgery under general anesthesia. *Anesth Analg.* 2005; 101(2):356-361, table of contents.
186. Khan A, Iqbal M, Yunus A, Ali K. Comparison of ondansetron and metoclopramide in the prevention of post-operative nausea and vomiting after laparoscopic cholecystectomy. *Pakistan Journal of Medical and Health Sciences.* 2011; 5(4):674-676.
187. Khan MP, Kohli M, Kumar GA, Singh V. Granisetron and granisetron dexamethasone combination for prevention of postoperative nausea and vomiting after laparoscopic cholecystectomy: A double blind, placebo controlled study *J Anaesthesiol Clin Pharmacol.* 2006; 22(3):261-265.
188. Khan P. Comparative study between granisetron ondansetron and propofol for the prevention of emesis after gynaecological laproscopy. *Journal of Postgraduate Medical Institute.* 2005; 19(2):135-143.
189. Kim SI, Kim SC, Baek YH, Ok SY, Kim SH. Comparison of ramosetron with ondansetron for prevention of postoperative nausea and vomiting in patients undergoing gynaecological surgery. *Br J Anaesth.* 2009; 103(4):549-553.
190. Kimya Y, Tatlikazan S, Bilgin H, Bilgin T, Cengiz C. Ondansetron: The prevention of nausea and vomiting in gynecologic operations. *Turkish Journal of Medical Sciences.* 1996; 26(4):339-342.
191. Klockgether-Radke A, Neumann S, Neumann P, Braun U, Muhlendyck H. Ondansetron, droperidol and their combination for the prevention of post-operative vomiting in children. *Eur J Anaesthesiol.* 1997; 14(4):362-367.
192. Kocamanoglu IS, Baris S, Karakaya D, Sener B, Tur A, Cetinkaya M. Effects of granisetron with droperidol or dexamethasone on prevention of postoperative nausea and vomiting after general anesthesia for cesarean section. *Methods Find Exp Clin Pharmacol.* 2005; 27(7):489-493.
193. Koh IJ, Chang CB, Jeon YT, Ryu JH, Kim TK. Does ramosetron reduce postoperative emesis and pain after TKA? *Clin Orthop Relat Res.* 2012; 470(6):1718-1727.
194. Koivuranta M, Ala-Kokko TI, Jokela R, Ranta P. Comparison of ondansetron and tropisetron combined with droperidol for the prevention of emesis in women with a history of post-operative nausea and vomiting. *Eur J Anaesthesiol.* 1999; 16(6):390-395.
195. Koivuranta M, Laara E, Ranta P, Ravaska P, Alahuhta S. Comparison of ondansetron and droperidol in the prevention of postoperative nausea and vomiting after laparoscopic surgery in women. A randomised, double-blind, placebo-controlled trial. *Acta Anaesthesiol Scand.* 1997; 41(10):1273-1279.
196. Koivuranta MK, Laara E, Ryhanen PT. Antiemetic efficacy of prophylactic ondansetron in laparoscopic cholecystectomy. A randomised, double-blind, placebo-controlled trial. *Anaesthesia.* 1996; 51(1):52-55.

197. Kolm A, Ferraz AA, Modolo NS, Ferrari F, Ganem EM, Rodrigues Junior GR *et al*. [Prevention of itching after spinal sufentanil: effects of droperidol, nalbuphine, ondansetron and the association of them.]. *Rev Bras Anestesiol.* 2006; 56(1):28-33.
198. Kontrimaviciute E, Baublys A, Ivaskevicius J. Postoperative nausea and vomiting in patients undergoing total abdominal hysterectomy under spinal anaesthesia: a randomized study of ondansetron prophylaxis. *Eur J Anaesthesiol.* 2005; 22(7):504-509.
199. Kothari SN, Boyd WC, Bottcher ML, Lambert PJ. Antiemetic efficacy of prophylactic dimenhydrinate (Dramamine) vs ondansetron (Zofran): a randomized, prospective trial inpatients undergoing laparoscopic cholecystectomy. *Surg Endosc.* 2000; 14(10):926-929.
200. Kovac A, McKenzie R, O'Connor T, Duncalf D, Angel J, Gratz I *et al*. Prophylactic intravenous ondansetron in female outpatients undergoing gynaecological surgery: a multicentre dose-comparison study. *Eur J Anaesthesiol Suppl.* 1992; 6:37-47.
201. Kovac AL, Eberhart L, Kotarski J, Clerici G, Apfel C, Palonosetron 04-07 Study G. A randomized, double-blind study to evaluate the efficacy and safety of three different doses of palonosetron versus placebo in preventing postoperative nausea and vomiting over a 72-hour period. *Anesth Analg.* 2008; 107(2):439-444.
202. Kovac AL, O'Connor TA, Pearman MH, Kekoler LJ, Edmondson D, Baughman VL *et al*. Efficacy of repeat intravenous dosing of ondansetron in controlling postoperative nausea and vomiting: a randomized, double-blind, placebo-controlled multicenter trial. *J Clin Anesth.* 1999; 11(6):453-459.
203. Kovac AL, Pearman MH, Khalil SN, Scuderi PE, Joslyn AF, Prillaman BA *et al*. Ondansetron prevents postoperative emesis in male outpatients. S3A-379 Study Group. *J Clin Anesth.* 1996; 8(8):644-651.
204. Kovac AL, Scuderi PE, Boerner TF, Chelly JE, Goldberg ME, Hantler CB *et al*. Treatment of postoperative nausea and vomiting with single intravenous doses of dolasetron mesylate: a multicenter trial. Dolasetron Mesylate PONV Treatment Study Group. *Anesth Analg.* 1997; 85(3):546-552.
205. Kreisler NS, Spiekermann BF, Ascari CM, Rhyne HA, Kloth RL, Sullivan LM *et al*. Small-dose droperidol effectively reduces nausea in a general surgical adult patient population. *Anesth Analg.* 2000; 91(5):1256-1261.
206. Krobbuaban B, Pitakpol S, Diregpoke S. Ondansetron vs. metoclopramide for the prevention of nausea and vomiting after gynecologic surgery. *J Med Assoc Thai.* 2008; 91(5):669-674.
207. Ku PK, Tong MC, Lo P, van Hasselt CA. Efficacy of ondansetron for prevention of postoperative nausea and vomiting after outpatient ear surgery under local anesthesia. *Am J Otol.* 2000; 21(1):24-27.
208. Kumar A, Agarwal M, Bhattacharya A. Effect of Ondansetron and metoclopramide on vigilance, cognition and recovery time following major gynaecological surgery. *Journal of Anaesthesiology Clinical Pharmacology.* 1996; 12(1):35-38.
209. Kushwaha BB, Chakraborty A, Agarwal J, Malick A, Bushan S, Bhattacharya P. Comparative study of granisetron and ondansetron alone and their combination with dexamethasone, for prevention of PONV in middle ear surgery. *Internet Journal of Anesthesiology.* 2007; 13(2):10.
210. Kyokong O, Somboonviboon W, Visalyaputra S, Pausawadi S, Saratan P, Vongvises P. Comparison of ondansetron and placebo for preventing postoperative nausea and emesis in gastrointestinal tract surgery: a multicenter randomized controlled trial. *J Med Assoc Thai.* 1999; 82(2):173-177.
211. Lahsaei SM, Amini A, Tabatabei SM, Mehrabani G. A comparison between cetirizine and ondansetron in preventing postoperative nausea and vomiting in adults. *J Res Med Sci.* 2012; 17(8):760-763.
212. Larijani GE, Gratz I, Afshar M, Minassian S. Treatment of postoperative nausea and vomiting with ondansetron: a randomized, double-blind comparison with placebo. *Anesth Analg.* 1991; 73(3):246-249.
213. Lawhorn CD, Bower C, Brown RE, Jr., Schmitz ML, Kymer PJ, Stoner J *et al*. Ondansetron decreases postoperative vomiting in pediatric patients undergoing tonsillectomy and adenoidectomy. *Int J Pediatr Otorhinolaryngol.* 1996; 36(2):99-108.
214. Lawhorn CD, Kymer PJ, Stewart FC, Stoner JM, Shirey R, Volpe P. Ondansetron dose response curve in high-risk pediatric patients. *J Clin Anesth.* 1997; 9(8):637-642.
215. Lee HJ, Kwon JY, Shin SW, Kim CH, Baek SH, Baik SW *et al*. Preoperatively administered ramosetron oral disintegrating tablets for preventing nausea and vomiting associated with patient-controlled analgesia in breast cancer patients. *Eur J Anaesthesiol.* 2008; 25(9):756-762.
216. Lee JW, Park HJ, Choi J, Park SJ, Kang H, Kim EG. Comparison of ramosetron's and ondansetron's preventive anti-emetic effects in highly susceptible patients undergoing abdominal hysterectomy. *Korean J Anesthesiol.* 2011; 61(6):488-492.
217. Lee SY, Lee JY, Park SY, Kim JH, Cho OG, Kim JS *et al*. Prophylactic antiemetic efficacy of granisetron or ramosetron in patients undergoing thyroidectomy. *Asian J Surg.* 2002; 25(4):309-314.
218. Lee TH, Lin CR, Lee TC, Buerkle H, Hsu TY, Mao CC *et al*. Failure of prevention against postoperative vomiting by ondansetron or prochlorperazine in patients undergoing gynecological laparoscopy. *Acta Anaesthesiol Sin.* 2000; 38(4):201-205.
219. Lee Y, Wang PK, Lai HY, Yang YL, Chu CC, Wang JJ. Haloperidol is as effective as ondansetron for preventing postoperative nausea and vomiting. *Can J Anaesth.* 2007; 54(5):349-354.

220. Leeser J, Lip H. Prevention of postoperative nausea and vomiting using ondansetron, a new, selective, 5-HT3 receptor antagonist. *Anesth Analg.* 1991; 72(6):751-755.
221. Lekprasert V, Meesangnil S, Pausawasdi S, Pongravee V. Efficacy of prophylactic ondansetron in Thai patients undergoing gastrointestinal tract surgery. *J Med Assoc Thai.* 1996; 79(6):382-387.
222. Leksowski K, Peryga P, Szyca R. Ondansetron, metoclopramid, dexamethason, and their combinations compared for the prevention of postoperative nausea and vomiting in patients undergoing laparoscopic cholecystectomy: a prospective randomized study. *Surg Endosc.* 2006; 20(6):878-882.
223. Liberman MA, Howe S, Lane M. Ondansetron versus placebo for prophylaxis of nausea and vomiting in patients undergoing ambulatory laparoscopic cholecystectomy. *Am J Surg.* 2000; 179(1):60-62.
224. Litman RS, Wu CL, Catanzaro FA. Ondansetron decreases emesis after tonsillectomy in children. *Anesth Analg.* 1994; 78(3):478-481.
225. Litman RS, Wu CL, Lee A, Griswold JD, Voisine R, Marshall C. Prevention of emesis after strabismus repair in children: a prospective, double-blinded, randomized comparison of droperidol versus ondansetron. *J Clin Anesth.* 1995; 7(1):58-62.
226. Loewen P, Lamb S, Clugston P. Randomized, double-blind trial of dolasetron versus droperidol for prophylaxis of postoperative nausea and vomiting in patients undergoing TRAM flap breast reconstruction surgery. *Ann Plast Surg.* 2003; 51(5):472-477.
227. Lopez-Olaondo L, Carrascosa F, Pueyo FJ, Monedero P, Busto N, Saez A. Combination of ondansetron and dexamethasone in the prophylaxis of postoperative nausea and vomiting. *Br J Anaesth.* 1996; 76(6):835-840.
228. Maddali MM, Mathew J, Fahr J, Zarroug AW. Postoperative nausea and vomiting in diagnostic gynaecological laparoscopic procedures: comparison of the efficacy of the combination of dexamethasone and metoclopramide with that of dexamethasone and ondansetron. *Journal of Postgraduate Medical Institute.* 2003; 49(4):302-306.
229. Madenoglu H, Akdemir C, Aksu R, Bicer C, Ulgey A, Boyaci A. The effect of palonosetron on postoperative nausea and vomiting in supratentorial craniotomy patients. *Turkish Journal of Medical Sciences.* 2012; 42:1241-1246.
230. Maestre JM, Puente J, Dierssen T. Prevention of postoperative nausea and vomiting with metoclopramide, droperidol and ondansetron: a randomized, double-blind comparison with placebo in ambulatory surgery. *Ambulatory Surgery.* 1997; 5(4):153-159.
231. Malins AF, Field JM, Nesling PM, Cooper GM. Nausea and vomiting after gynaecological laparoscopy: comparison of premedication with oral ondansetron, metoclopramide and placebo. *Br J Anaesth.* 1994; 72(2):231-233.
232. Manani G, Cantele P, Dona B, Meroni M, Tiberio I, Ceschin C *et al*. Oculo-emetic and oculo-cardiac reflex in the prevention of postoperative nausea and vomitus by diehydrobenzperidol and ondansetron after squint surgery. *Acta Anaesthesiologica Italica.* 1996; 47(3):201-209.
233. Marcus JR, Few JW, Chao JD, Fine NA, Mustoe TA. The prevention of emesis in plastic surgery: a randomized, prospective study. *Plast Reconstr Surg.* 2002; 109(7):2487-2494.
234. McCall JE, Stubbs K, Saylors S, Pohlman S, Ivers B, Smith S *et al.* The search for cost-effective prevention of postoperative nausea and vomiting in the child undergoing reconstructive burn surgery: ondansetron versus dimenhydrinate. *J Burn Care Rehabil.* 1999; 20(4):309-315.
235. McKenzie R, Kovac A, O'Connor T, Duncalf D, Angel J, Gratz I *et al*. Comparison of ondansetron versus placebo to prevent postoperative nausea and vomiting in women undergoing ambulatory gynecologic surgery. *Anesthesiology.* 1993; 78(1):21-28.
236. McKenzie R, Riley TJ, Tantisira B, Hamilton DL. Effect of propofol for induction and ondansetron with or without dexamethasone for the prevention of nausea and vomiting after major gynecologic surgery. *J Clin Anesth.* 1997; 9(1):15-20.
237. McKenzie R, Sharifi-Azad S, Dershwitz M, Miguel R, Joslyn AF, Tantisira B *et al*. A randomized, double-blind pilot study examining the use of intravenous ondansetron in the prevention of postoperative nausea and vomiting in female inpatients. *J Clin Anesth.* 1993; 5(1):30-36.
238. Metaxari M, Papaioannou A, Petrou A, Chatzimichali A, Pharmakalidou E, Askitopoulou H. Antiemetic prophylaxis in thyroid surgery: a randomized, double-blind comparison of three 5-HT3 agents. *J Anesth.* 2011; 25(3):356-362.
239. Meyer TA, Roberson CR, Rajab MH, Davis J, McLeskey CH. Dolasetron versus ondansetron for the treatment of postoperative nausea and vomiting. *Anesth Analg.* 2005; 100(2):373-377.
240. Mikawa K, Takao Y, Nishina K, Maekawa N, Obara H. The antiemetic efficacy of prophylactic granisetron in gynecologic surgery. *Anesth Analg.* 1995; 80(5):970-974.
241. Mikawa K, Takao Y, Nishina K, Shiga M, Maekawa N, Obara H. Optimal dose of granisetron for prophylaxis against postoperative emesis after gynecological surgery. *Anesth Analg.* 1997; 85(3):652-656.
242. Millo J, Siddons M, Innes R, Laurie PS. Randomised double-blind comparison of ondansetron and droperidol to prevent postoperative nausea and vomiting associated with patient-controlled analgesia. *Anaesthesia.* 2001; 56(1):60-65.

243. Mishra AR, Srivastava U, Kumar D, Saraswat N, Kumar A, Payal YS *et al*. Nausea and vomiting after ENT surgeries: A comparison between ondansetron, metoclopramide and small dose of propofol. *Indian J Otolaryngol Head Neck Surg.* 2010; 62(1):29-31.
244. Misra MN, Pullani AJ, Mohamed ZU. Prevention of PONV by acustimulation with capsicum plaster is comparable to ondansetron after middle ear surgery. *Can J Anaesth.* 2005; 52(5):485-489.
245. Moens P, Levarlet M, Hendrickx P, De Guchteneere E. Single i.v. bolus dose of ondansetron in the prevention of postoperative nausea and emesis. *Acta Anaesthesiol Belg.* 1997; 48(4):245-250.
246. Monagle J, Barnes R, Goodchild C, Hewitt M. Ondansetron is not superior to moderate dose metoclopramide in the prevention of post-operative nausea and vomiting after minor gynaecological surgery. *Eur J Anaesthesiol.* 1997; 14(6):604-609.
247. Moon YE, Joo J, Kim JE, Lee Y. Anti-emetic effect of ondansetron and palonosetron in thyroidectomy: a prospective, randomized, double-blind study. *Br J Anaesth.* 2012; 108(3):417-422.
248. Morris RW, Aune H, Feiss P, Hanson A, Hasselstrom L, Maltby JR *et al*. International, multicentre, placebo-controlled study to evaluate the effectiveness of ondansetron vs. metoclopramide in the prevention of post-operative nausea and vomiting. *Eur J Anaesthesiol.* 1998; 15(1):69-79.
249. Morton NS, Camu F, Dorman T, Knudsen KE, Kvalsvik O, Nellgard P *et al*. Ondansetron reduces nausea and vomiting after paediatric adenotonsillectomy. *Paediatr Anaesth.* 1997; 7(1):37-45.
250. Moussa AA, Oregan PJ. Prevention of postoperative nausea and vomiting in patients undergoing laparoscopic bariatric surgery--granisetron alone vs granisetron combined with dexamethasone/droperidol. *Middle East J Anesthesiol.* 2007; 19(2):357-367.
251. Mullick P, Prakash S, Naithani BK, Pawar M. The anti-emetic efficacy of a combination of ondansetron and metoclopramide. *Journal of Anaesthesiology Clinical Pharmacology.* 2002; 18(2):177-181.
252. Munro FJ, Fisher S, Dickson U, Morton N. The addition of antiemetics to the morphine solution in patient controlled analgesia syringes used by children after an appendicectomy does not reduce the incidence of postoperative nausea and vomiting. *Paediatr Anaesth.* 2002; 12(7):600-603.
253. Munro HM, D'Errico CC, Lauder GR, Wagner DS, Voepel-Lewis T, Tait AR. Oral granisetron for strabismus surgery in children. *Can J Anaesth.* 1999; 46(1):45-48.
254. Naguib M, el Bakry AK, Khoshim MH, Channa AB, el Gammal M, el Gammal K *et al*. Prophylactic antiemetic therapy with ondansetron, tropisetron, granisetron and metoclopramide in patients undergoing laparoscopic cholecystectomy: a randomized, double-blind comparison with placebo. *Can J Anaesth.* 1996; 43(3):226-231.
255. Najeeb R, Naqash I, Shah ZA, Habib M, Kant S. 'A comparative study of two antiemetics: Droperidol and Granisetron in the prevention of post anaesthesia nausea and vomiting'. *JK Practitioner.* 2000; 7(1):52-54.
256. Ng WW, Li AC, Lee DW, Leung TL, Ko CW, Leung KF *et al*. Effectiveness of Ondansetron to prevent postoperative nausea and vomiting in ambulatory two port needlescopic cholecystectomy: a randomised controlled trial. *Ambulatory surgery.* 2008; 14(2):1-14.
257. O'Brien B, Nevin B, Patterson K. A comparison of papaveretum-promethazine with morphine-ondansetron for patient-controlled analgesia. *Ir J Med Sci.* 2000; 169(1):58-59.
258. O'Brien CM, Titley G, Whitehurst P. A comparison of cyclizine, ondansetron and placebo as prophylaxis against postoperative nausea and vomiting in children. *Anaesthesia.* 2003; 58(7):707-711.
259. Oksuz H, Zencirci B, Ezberci M. Comparison of the effectiveness of metoclopramide, ondansetron, and granisetron on the prevention of nausea and vomiting after laparoscopic cholecystectomy. *J Laparoendosc Adv Surg Tech A.* 2007; 17(6):803-808.
260. Olatosi OJ, Kushimo O, Okeke C, Oriyomi O, Ajayi GO. Antiemetic prophylaxis with promethazine or ondansetron in major gynaecological surgery. *Southern African Journal of Anaesthesia and Analgesia.* 2008; 14(6):39-42.
261. Olutoye O, Jantzen EC, Alexis R, Rajchert D, Schreiner MS, Watcha MF. A comparison of the costs and efficacy of ondansetron and dolasetron in the prophylaxis of postoperative vomiting in pediatric patients undergoing ambulatory surgery. *Anesth Analg.* 2003; 97(2):390-396, table of contents.
262. Omar SH, Kamel NM, Radwan KG, Ebied RS, Ali MZ, Osman ES *et al*. Sub-antibiotic doses of erythromycin as a prokinetic in abdominal surgeries: reviving the old. *J Egypt Soc Parasitol.* 2010; 40(3):809-820.
263. Ozmen S, Yavuz L, Ceylan BG, Tarhan O, Aydin C. Comparison of granisetron with granisetron plus droperidol combination prophylaxis in post-operative nausea and vomiting after laparoscopic cholecystectomy. *J Int Med Res.* 2002; 30(5):520-524.
264. Paech MJ, Lee BH, Evans SF. The effect of anaesthetic technique on postoperative nausea and vomiting after day-case gynaecological laparoscopy. *Anaesth Intensive Care.* 2002; 30(2):153-159.
265. Paech MJ, Pavy TJ, Evans SF. Single-dose prophylaxis for postoperative nausea and vomiting after major abdominal surgery: ondansetron versus droperidol. *Anaesth Intensive Care.* 1995; 23(5):548-554.
266. Paech MJ, Rucklidge MW, Banks SL, Gurrin LC, Orlikowski CE, Pavy TJ. The efficacy and cost-effectiveness of prophylactic 5-hydroxytryptamine3 receptor antagonists: tropisetron, ondansetron and dolasetron. *Anaesth Intensive Care.* 2003; 31(1):11-17.

267. Paech MJ, Rucklidge MW, Lain J, Dodd PH, Bennett EJ, Doherty DA. Ondansetron and dexamethasone dose combinations for prophylaxis against postoperative nausea and vomiting. *Anesth Analg.* 2007; 104(4):808-814.
268. Pan PH, Lee SC, Harris LC. Antiemetic prophylaxis for postdischarge nausea and vomiting and impact on functional quality of living during recovery in patients with high emetic risks: a prospective, randomized, double-blind comparison of two prophylactic antiemetic regimens. *Anesth Analg.* 2008; 107(2):429-438.
269. Pan PH, Moore CH. Intraoperative antiemetic efficacy of prophylactic ondansetron versus droperidol for cesarean section patients under epidural anesthesia. *Anesth Analg.* 1996; 83(5):982-986.
270. Pan PH, Moore CH. Comparing the efficacy of prophylactic metoclopramide, ondansetron, and placebo in cesarean section patients given epidural anesthesia. *J Clin Anesth.* 2001; 13(6):430-435.
271. Park JW, Jun JW, Lim YH, Lee SS, Yoo BH, Kim KM *et al*. The comparative study to evaluate the effect of palonosetron monotherapy versus palonosetron with dexamethasone combination therapy for prevention of postoperative nausea and vomiting. *Korean J Anesthesiol.* 2012; 63(4):334-339.
272. Park SK, Cho EJ. A randomized, double-blind trial of palonosetron compared with ondansetron in preventing postoperative nausea and vomiting after gynaecological laparoscopic surgery. *J Int Med Res.* 2011; 39(2):399-407.
273. Patel RI, Davis PJ, Orr RJ, Ferrari LR, Rimar S, Hannallah RS *et al*. Single-dose ondansetron prevents postoperative vomiting in pediatric outpatients. *Anesth Analg.* 1997; 85(3):538-545.
274. Paventi S, Santevecchi A, Ranieri R. Efficacy of a single-dose ondansetron for preventing post-operative nausea and vomiting after laparoscopic cholecystectomy with sevoflurane and remifentanil infusion anaesthesia. *Eur Rev Med Pharmacol Sci.* 2001; 5(2):59-63.
275. Akin A, Esmaoglu A, Gunes I, Boyaci A. The effects of the prophylactic tropisetron-propofol combination on postoperative nausea and vomiting in patients undergoing thyroidectomy under desflurane anesthesia. *Mt Sinai J Med.* 2006; 73(2):560-563.
276. Blitz JD, Haile M, Kline R, Franco L, Didehvar S, Pachter HL *et al*. A randomized double blind study to evaluate efficacy of palonosetron with dexamethasone versus palonosetron alone for prevention of postoperative and postdischarge nausea and vomiting in subjects undergoing laparoscopic surgeries with high emetogenic risk. *Am J Ther.* 2012; 19(5):324-329.
277. Capouet V, De Pauw C, Vernet B, Ivens D, Derijcke V, Versichelen L *et al*. Single dose i.v. tropisetron in the prevention of postoperative nausea and vomiting after gynaecological surgery. *Br J Anaesth.* 1996; 76(1):54-60.
278. Chan MT, Chui PT, Ho WS, King WW. Single-dose tropisetron for preventing postoperative nausea and vomiting after breast surgery. *Anesth Analg.* 1998; 87(4):931-935.
279. De Witte JL, Schoenmaekers B, Sessler DI, Deloof T. The analgesic efficacy of tramadol is impaired by concurrent administration of ondansetron. *Anesth Analg.* 2001; 92(5):1319-1321.
280. Gurkan Y, Toker K. Prophylactic ondansetron reduces the incidence of intrathecal fentanyl-induced pruritus. *Anesth Analg.* 2002; 95(6):1763-1766, table of contents.
281. Habib AS, Reuveni J, Taguchi A, White WD, Gan TJ. A comparison of ondansetron with promethazine for treating postoperative nausea and vomiting in patients who received prophylaxis with ondansetron: a retrospective database analysis. *Anesth Analg.* 2007; 104(3):548-551.
282. Jokela R, Koivuranta M. Tropisetron or droperidol in the prevention of postoperative nausea and vomiting. A comparative, randomised, double-blind study in women undergoing laparoscopic cholecystectomy. *Acta Anaesthesiol Scand.* 1999; 43(6):645-650.
283. Kelsaka E, Baris S, Karakaya D, Sarihasan B. Comparison of ondansetron and meperidine for prevention of shivering in patients undergoing spinal anesthesia. *Reg Anesth Pain Med.* 2006; 31(1):40-45.
284. Korttila K, Clergue F, Leeser J, Feiss P, Olthoff D, Payeur-Michel C *et al*. Intravenous dolasetron and ondansetron in prevention of postoperative nausea and vomiting: a multicenter, double-blind, placebo-controlled study. *Acta Anaesthesiol Scand.* 1997; 41(7):914-922.
285. Paxton D, Taylor RH, Gallagher TM, Crean PM. Postoperative emesis following otoplasty in children. *Anaesthesia.* 1995; 50(12):1083-1085.
286. Paxton LD, McKay AC, Mirakhur RK. Prevention of nausea and vomiting after day case gynaecological laparoscopy. A comparison of ondansetron, droperidol, metoclopramide and placebo. *Anaesthesia.* 1995; 50(5):403-406.
287. Peixoto AJ, Celich MF, Zardo L, Peixoto Filho AJ. Ondansetron or droperidol for prophylaxis of nausea and vomiting after intrathecal morphine. *Eur J Anaesthesiol.* 2006; 23(8):670-675.
288. Peixoto AJ, Peixoto Filho AJ, Leaes LF, Celich MF, Barros MA. Efficacy of prophylactic droperidol, ondansetron or both in the prevention of postoperative nausea and vomiting in major gynaecological surgery. A prospective, randomized, double-blind clinical trial. *Eur J Anaesthesiol.* 2000; 17(10):611-615.
289. Philip BK, Pearman MH, Kovac AL, Chelly JE, Wetchler BV, McKenzie R *et al*. Dolasetron for the prevention of postoperative nausea and vomiting following outpatient surgery with general anaesthesia: a randomized, placebo-controlled study. The Dolasetron PONV Prevention Study Group. *Eur J Anaesthesiol.* 2000; 17(1):23-32.

290. Piper SN, Rohm K, Boldt J, Kranke P, Maleck W, Seifert R *et al*. Postoperative nausea and vomiting after surgery for prognathism: not only a question of patients' comfort. A placebo-controlled comparison of dolasetron and droperidol. *Journal of Cranio-Maxillofacial Surgery.* 2008; 36(3):173-179.
291. Pirat A, Tuncay SF, Torgay A, Candan S, Arslan G. Ondansetron, orally disintegrating tablets versus intravenous injection for prevention of intrathecal morphine-induced nausea, vomiting, and pruritus in young males. *Anesth Analg.* 2005; 101(5):1330-1336.
292. Pitkanen MT, Numminen MK, Tuominen MK, Rosenberg PH. Comparison of metoclopramide and ondansetron for the prevention of nausea and vomiting after intrathecal morphine. *Eur J Anaesthesiol.* 1997; 14(2):172-177.
293. Piwko C, Lasry A, Alanezi K, Coyte PC, Ungar WJ. Economic evaluation of ondansetron vs dimenhydrinate for prevention of postoperative vomiting in children undergoing strabismus surgery. *Paediatr Anaesth.* 2005; 15(9):755-761.
294. Polati E, Verlato G, Finco G, Mosaner W, Grosso S, Gottin L *et al*. Ondansetron versus metoclopramide in the treatment of postoperative nausea and vomiting. *Anesth Analg.* 1997; 85(2):395-399.
295. Principi F, Di AP, Sofra M, Salerno S, Aloe L. Intravenous ondansetron in the prophylactic treatment of postoperative nausea and vomiting in gynaecological surgery. *Acta Anaesthesiologica Italica.* 1996; 47(2):147-156.
296. Pueyo FJ, Carrascosa F, Lopez L, Iribarren MJ, Garcia-Pedrajas F, Saez A. Combination of ondansetron and droperidol in the prophylaxis of postoperative nausea and vomiting. *Anesth Analg.* 1996; 83(1):117-122.
297. Pugh SC, Jones NC, Barsoum LZ. A comparison of prophylactic ondansetron and metoclopramide administration in patients undergoing major neurosurgical procedures. *Anaesthesia.* 1996; 51(12):1162-1164.
298. Purhonen S, Koski EM, Niskanen M, Hynynen M. Efficacy and costs of 3 anesthetic regimens in the prevention of postoperative nausea and vomiting. *J Clin Anesth.* 2006; 18(1):41-45.
299. Purhonen S, Niskanen M, Wustefeld M, Hirvonen E, Hynynen M. Supplemental 80% oxygen does not attenuate post-operative nausea and vomiting after breast surgery. *Acta Anaesthesiol Scand.* 2006; 50(1):26-31.
300. Quaynor H, Raeder JC. Incidence and severity of postoperative nausea and vomiting are similar after metoclopramide 20 mg and ondansetron 8 mg given by the end of laparoscopic cholecystectomies. *Acta Anaesthesiol Scand.* 2002; 46(1):109-113.
301. Rabbani MZ, Khan MN, Qureshi R, Zubair M, Pervez MB. Does the added benefit of ondansetron over dexamethasone, to control post-operative nausea and vomiting, justify the added cost in patients undergoing tonsillectomy and adenotonsillectomy? *J Pak Med Assoc.* 2010; 60(7):559-561.
302. Rajeeva V, Bhardwaj N, Batra YK, Dhaliwal LK. Comparison of ondansetron with ondansetron and dexamethasone in prevention of PONV in diagnostic laparoscopy. *Canadian Journal of Anesthesia.* 1999; 46(1):40-44.
303. Raphael JH, Norton AC. Antiemetic efficacy of prophylactic ondansetron in laparoscopic surgery: randomized, double-blind comparison with metoclopramide. *Br J Anaesth.* 1993; 71(6):845-848.
304. Rauers NI, Stuber F, Lee EH, Musshoff F, Fimmers R, Barann M *et al*. Antagonistic effects of ondansetron and tramadol? A randomized placebo and active drug controlled study. *J Pain.* 2010; 11(12):1274-1281.
305. Reihner E, Grunditz R, Giesecke K, Gustafsson LL. Postoperative nausea and vomiting after breast surgery: efficacy of prophylactic ondansetron and droperidol in a randomized placebo-controlled study. *Eur J Anaesthesiol.* 2000; 17(3):197-203.
306. Riad W, Marouf H. Combination therapy in the prevention of PONV after strabismus surgery in children: granisetron, ondansetron, midazolam with dexamethasone. *Middle East J Anesthesiol.* 2009; 20(3):431-436.
307. Rodrigo C, Campbell R, Chow J, Tong A. The effect of a 4-mg preoperative intravenous dose of ondansetron in preventing nausea and vomiting after maxillofacial surgery. *J Oral Maxillofac Surg.* 1996; 54(10):1171-1175.
308. Rodrigo MR, Campbell RC, Chow J, Tong CK, Hui E, Lueveswanij S. Ondansetron for prevention of postoperative nausea and vomiting following minor oral surgery: a double-blind randomized study. *Anaesth Intensive Care.* 1994; 22(5):576-579.
309. Rose JB, Brenn BR, Corddry DH, Thomas PC. Preoperative oral ondansetron for pediatric tonsillectomy. *Anesth Analg.* 1996; 82(3):558-562.
310. Rose JB, Martin TM. Posttonsillectomy vomiting. Ondansetron or metoclopramide during paediatric tonsillectomy: are two doses better than one? *Paediatr Anaesth.* 1996; 6(1):39-44.
311. Rose JB, Martin TM, Corddry DH, Zagnoev M, Kettrick RG. Ondansetron reduces the incidence and severity of poststrabismus repair vomiting in children. *Anesth Analg.* 1994; 79(3):486-489.
312. Rosow CE, Haspel KL, Smith SE, Grecu L, Bittner EA. Haloperidol versus ondansetron for prophylaxis of postoperative nausea and vomiting. *Anesth Analg.* 2008; 106(5):1407-1409, table of contents.
313. Ruiz de Adana J, Tobalina Bonis R, Garcia Galan F, Hernandez Matias A, Fernandez Luengas D, Ortega Deballon P *et al*. Antiemetic efficacy of ondansetron in laparoscopic cholecystectomy. A randomized, double-blind, placebo-controlled study. *Rev Esp Enferm Dig.* 1999; 91(9):639-643.

314. Rung GW, Claybon L, Hord A, Patel C, Kallgren M, Koppel J *et al*. Intravenous ondansetron for postsurgical opioid-induced nausea and vomiting. S3A-255 Study Group. *Anesth Analg.* 1997; 84(4):832-838.
315. Rusch D, Arndt C, Martin H, Kranke P. The addition of dexamethasone to dolasetron or haloperidol for treatment of established postoperative nausea and vomiting. *Anaesthesia.* 2007; 62(8):810-817.
316. Ryu J, So YM, Hwang J, Do SH. Ramosetron versus ondansetron for the prevention of postoperative nausea and vomiting after laparoscopic cholecystectomy. *Surg Endosc.* 2010; 24(4):812-817.
317. Ryu JH, Jeon YT, Hwang JW, Oh AY, Moon JY, Ro YJ *et al*. Intravenous, oral, and the combination of intravenous and oral ramosetron for the prevention of nausea and vomiting after laparoscopic cholecystectomy: a randomized, double-blind, controlled trial. *Clin Ther.* 2011; 33(9):1162-1172.
318. Sadhasivam S, Saxena A, Kathirvel S, Kannan TR, Trikha A, Mohan V. The safety and efficacy of prophylactic ondansetron in patients undergoing modified radical mastectomy. *Anesth Analg.* 1999; 89(6):1340-1345.
319. Sadhasivam S, Shende D, Madan R. Prophylactic ondansetron in prevention of postoperative nausea and vomiting following pediatric strabismus surgery: a dose-response study. *Anesthesiology.* 2000; 92(4):1035-1042.
320. Sagir O, Gulhas N, Toprak H, Yucel A, Begec Z, Ersoy O. Control of shivering during regional anaesthesia: prophylactic ketamine and granisetron. *Acta Anaesthesiol Scand.* 2007; 51(1):44-49.
321. Sahoo T, SenDasgupta C, Goswami A, Hazra A. Reduction in spinal-induced hypotension with ondansetron in parturients undergoing caesarean section: a double-blind randomised, placebo-controlled study. *Int J Obstet Anesth.* 2012; 21(1):24-28.
322. Said-Ahmed HAF. Does granisetron affect the analgesic efficacy of tramadol in patients undergoing abdominal hysterectomy? *Acta Anaesthesiologica Italica.* 2008; 59(3):256-266.
323. Sakellaris G, Georgogianaki P, Astyrakaki E, Michalakis M, Dede O, Alegakis A *et al*. Prevention of post-operative nausea and vomiting in children--a prospective randomized double-blind study. *Acta Paediatr.* 2008; 97(6):801-804.
324. Samarkandi AH, Riad W, Altaf R, Fatani R. Dexamethasone-ondansetron combination in prevention of nausea and vomiting after strabismus surgery in children. *Egyptian Journal of Anaesthesia.* 2004; 20(4):399-403.
325. Sanchez-Ledesma MJ, Lopez-Olaondo L, Pueyo FJ, Carrascosa F, Ortega A. A comparison of three antiemetic combinations for the prevention of postoperative nausea and vomiting. *Anesth Analg.* 2002; 95(6):1590-1595, table of contents.
326. Sandhu HS, Stockall CA, Ganapathy S, Spadafora SM, Watson JT. Comparison of ondansetron, dimenhydrinate versus placebo as PONV prophylaxis for outpatient gynecological laparoscopy. *Ambulatory Surgery.* 1999; 7(4):187-191.
327. Sandhu T, Tanvatcharaphan P, Cheunjongkolkul V. Ondansetron versus metoclopramide in prophylaxis of nausea and vomiting for laparoscopic cholecystectomy: a prospective double-blind randomized study. *Asian J Surg.* 2008; 31(2):50-54.
328. Sarvela PJ, Halonen PM, Soikkeli AI, Kainu JP, Korttila KT. Ondansetron and tropisetron do not prevent intraspinal morphine- and fentanyl-induced pruritus in elective cesarean delivery. *Acta Anaesthesiol Scand.* 2006; 50(2):239-244.
329. Schnaider TB, Vieira AM, Brandao AC. Comparative study of anti-emetics and their association, in the prevention of postoperative nausea and vomiting in patients undergoing gynecologic surgeries. *Rev Bras Anestesiol.* 2008; 58(6):614-622.
330. Scholz J, Hennes HJ, Steinfath M, Farber L, Schweiger C, Dick W *et al*. Tropisetron or ondansetron compared with placebo for prevention of postoperative nausea and vomiting. *Eur J Anaesthesiol.* 1998; 15(6):676-685.
331. Scuderi PE, James RL, Harris L, Mims GR, 3rd. Antiemetic prophylaxis does not improve outcomes after outpatient surgery when compared to symptomatic treatment. *Anesthesiology.* 1999; 90(2):360-371.
332. Scuderi PE, James RL, Harris L, Mims GR, 3rd. Multimodal antiemetic management prevents early postoperative vomiting after outpatient laparoscopy. *Anesth Analg.* 2000; 91(6):1408-1414.
333. Scuderi PE, Weaver RG, Jr., James RL, Mims G, Elliott WG, Weeks DB. A randomized, double-blind, placebo controlled comparison of droperidol, ondansetron, and metoclopramide for the prevention of vomiting following outpatient strabismus surgery in children. *J Clin Anesth.* 1997; 9(7):551-558.
334. Sennaraj B, Shende D, Sadhasivam S, Ilavajady S, Jagan D. Management of post-strabismus nausea and vomiting in children using ondansetron: a value-based comparison of outcomes. *Br J Anaesth.* 2002; 89(3):473-478.
335. Shakeel M, Trinidade A, Al-Adhami A, Khan I, Supriya M, Kubba H. Retrospective review of three antiemetic regimens versus no therapy in the prevention of postoperative and vomiting following adenotonsillar surgery. *J Otolaryngol Head Neck Surg.* 2012; 41(1):35-40.
336. Shakya S, Chaturvedi A, Sah BP. Prophylactic low dose ketamine and ondansetron for prevention of shivering during spinal anaesthesia. *J Anaesthesiol Clin Pharmacol.* 2010; 26(4):465-469.

337. Sharma S, Abdullah N. A comparison of commonly used anti-emetics for the prevention of emetic sequelae after a major gynaecological surgery. *Singapore Med J.* 2000; 41(4):147-150.
338. Sharma S, Goswami U. Evaluation of acupuncture for anti-emetic prophylaxis. *Journal of Anaesthesiology Clinical Pharmacology.* 2007; 23(4):401-404.
339. Shende D, Bharti N, Kathirvel S, Madan R. Combination of droperidol and ondansetron reduces PONV after pediatric strabismus surgery more than single drug therapy. *Acta Anaesthesiol Scand.* 2001; 45(6):756-760.
340. Shende D, Mandal NG. Efficacy of ondansetron and metoclopramide for preventing postoperative emesis following strabismus surgery in children. *Anaesthesia.* 1997; 52(5):496-500.
341. Singh DK, Jindal P, Singh G. Comparative study of attenuation of the pain caused by propofol intravenous injection, by granisetron, magnesium sulfate and nitroglycerine. *Saudi J Anaesth.* 2011; 5(1):50-54.
342. Singla NK, Singla SK, Chung F, Kutsogiannis DJ, Blackburn L, Lane SR *et al*. Phase II study to evaluate the safety and efficacy of the oral neurokinin-1 receptor antagonist casopitant (GW679769) administered with ondansetron for the prevention of postoperative and postdischarge nausea and vomiting in high-risk patients. *Anesthesiology.* 2010; 113(1):74-82.
343. Sinha PK, Ambesh SP. Ondansetron in prophylaxis of postoperative nausea and vomiting in patients undergoing breast surgery: a placebo-controlled double blind study. *J Indian Med Assoc.* 2004; 102(2):73-74, 76, 78-79.
344. Sinha PK, Tripathi M, Ambesh SP. Efficacy of ondansetron in prophylaxis of postoperative nausea and vomiting in patients following infratentorial surgery: a placebo-controlled prospective double-blind study. *J Neurosurg Anesthesiol.* 1999; 11(1):6-10.
345. Sniadach MS, Alberts MS. A comparison of the prophylactic antiemetic effect of ondansetron and droperidol on patients undergoing gynecologic laparoscopy. *Anesth Analg.* 1997; 85(4):797-800.
346. So JB, Cheong KF, Sng C, Cheah WK, Goh P. Ondansetron in the prevention of postoperative nausea and vomiting after laparoscopic cholecystectomy: a prospective randomized study. *Surg Endosc.* 2002; 16(2):286-288.
347. Sodhi K, B.K. M, Sodhi GS, Kumar M. A comparative Study of Granisetron, Dexamethasone, and Granisetron Plus Dexamethasone as Prophylactic Antiemetic Therapy in Female Patients Undergoing Breast Surgery. *Journal of Anaesthesiology Clinical Pharmacology.* 2007; 23(4):373-378.
348. Somri M, Vaida SJ, Sabo E, Yassain G, Gankin I, Gaitini LA. Acupuncture versus ondansetron in the prevention of postoperative vomiting. A study of children undergoing dental surgery. *Anaesthesia.* 2001; 56(10):927-932.
349. Song F, Altman DG, Glenny A-M, Deeks JJ. Validity of indirect comparison for estimating efficacy of competing interventions: empirical evidence from published meta-analyses. 2003; 326(7387):472.
350. Splinter WM. Prevention of vomiting after strabismus surgery in children: dexamethasone alone versus dexamethasone plus low-dose ondansetron. *Paediatr Anaesth.* 2001; 11(5):591-595.
351. Splinter WM, Baxter MR, Gould HM, Hall LE, MacNeill HB, Roberts DJ *et al*. Oral ondansetron decreases vomiting after tonsillectomy in children. *Can J Anaesth.* 1995; 42(4):277-280.
352. Splinter WM, Rhine EJ. Prophylactic antiemetics in children undergoing tonsillectomy: high-dose vs low-dose ondansetron. *Paediatr Anaesth.* 1997; 7(2):125-129; discussion 130.
353. Splinter WM, Rhine EJ. Low-dose ondansetron with dexamethasone more effectively decreases vomiting after strabismus surgery in children than does high-dose ondansetron. *Anesthesiology.* 1998; 88(1):72-75.
354. Splinter WM, Rhine EJ. Prophylaxis for vomiting by children after tonsillectomy: ondansetron compared with perphenazine. *Br J Anaesth.* 1998; 80(2):155-158.
355. Splinter WM, Rhine EJ, Roberts DW, Baxter MR, Gould HM, Hall LE *et al*. Ondansetron is a better prophylactic antiemetic than droperidol for tonsillectomy in children. *Can J Anaesth.* 1995; 42(10):848-851.
356. Sriraman R, Indu S, Chari P. Is granisetron-dexamethasone combination better than ondansetron-dexamethasone in the prevention of postoperative nausea and vomiting in outpatient gynaecological laparoscopy. *Journal of Anaesthesiology Clinical Pharmacology.* 2007; 23(4):365-372.
357. Stamer UM, Lee EH, Rauers NI, Zhang L, Kleine-Brueggeney M, Fimmers R *et al*. CYP2D6- and CYP3A-dependent enantioselective plasma concentrations of ondansetron in postanesthesia care. *Anesth Analg.* 2011; 113(1):48-54.
358. Stene FN, Seay RE, Young LA, Bohnsack LE, Bostrom BC. Prospective, randomized, double-blind, placebo-controlled comparison of metoclopramide and ondansetron for prevention of posttonsillectomy or adenotonsillectomy emesis. *J Clin Anesth.* 1996; 8(7):540-544.
359. Stienstra R, Samhan YM, el-Mofty M, de Bont LE, Bovill JG. Double-blind comparison of alizapride, droperidol and ondansetron in the treatment of post-operative nausea. *Eur J Anaesthesiol.* 1997; 14(3):290-294.
360. Subramaniam B, Madan R, Sadhasivam S, Sennaraj B, Tamilselvan P, Rajeshwari S *et al*. Dexamethasone is a cost-effective alternative to ondansetron in preventing PONV after paediatric strabismus repair. *Br J Anaesth.* 2001; 86(1):84-89.

361. Subramaniam K, Pandia MP, Dash M, Dash HH, Bithal PK, Bhatia A *et al*. Scheduled prophylactic ondansetron administration did not improve its antiemetic efficacy after intracranial tumour resection surgery in children. *Eur J Anaesthesiol.* 2007; 24(7):615-619.
362. Suen TK, Gin TA, Chen PP, Rowbottom YM, Critchley LA, Ray AK. Ondansetron 4 mg for the prevention of nausea and vomiting after minor laparoscopic gynaecological surgery. *Anaesth Intensive Care.* 1994; 22(2):142-146.
363. Sukhani R, Pappas AL, Lurie J, Hotaling AJ, Park A, Fluder E. Ondansetron and dolasetron provide equivalent postoperative vomiting control after ambulatory tonsillectomy in dexamethasone-pretreated children. *Anesth Analg.* 2002; 95(5):1230-1235, table of contents.
364. Sun R, Klein KW, White PF. The effect of timing of ondansetron administration in outpatients undergoing otolaryngologic surgery. *Anesth Analg.* 1997; 84(2):331-336.
365. Sung YF, Wetchler BV, Duncalf D, Joslyn AF. A double-blind, placebo-controlled pilot study examining the effectiveness of intravenous ondansetron in the prevention of postoperative nausea and emesis. *J Clin Anesth.* 1993; 5(1):22-29.
366. Swiatkowski J, Goral A, Dzieciuch JA, Przesmycki K. Assessment of ondansetron and droperidol for the prevention of post-operative nausea and vomiting after cholecystectomy and minor gynaecological surgery performed by laparoscopy. *Eur J Anaesthesiol.* 1999; 16(11):766-772.
367. Szarvas S, Chellapuri RS, Harmon DC, Owens J, Murphy D, Shorten GD. A comparison of dexamethasone, ondansetron, and dexamethasone plus ondansetron as prophylactic antiemetic and antipruritic therapy in patients receiving intrathecal morphine for major orthopedic surgery. *Anesth Analg.* 2003; 97(1):259-263, table of contents.
368. Talesh KT, Motamedi MH, Kahnamouii S. Comparison of ondansetron and metoclopramide antiemetic prophylaxis in maxillofacial surgery patients. *Oral Surg Oral Med Oral Pathol Oral Radiol Endod.* 2011; 111(3):275-277.
369. Tan T, Ojo R, Immani S, Choroszczak P, Carey M. Reduction of severity of pruritus after elective caesarean section under spinal anaesthesia with subarachnoid morphine: a randomised comparison of prophylactic granisetron and ondansetron. *Int J Obstet Anesth.* 2010; 19(1):56-60.
370. Tang J, Chen X, White PF, Wender RH, Ma H, Sloninsky A *et al*. Antiemetic prophylaxis for office-based surgery: are the 5-HT3 receptor antagonists beneficial? *Anesthesiology.* 2003; 98(2):293-298.
371. Tang J, D'Angelo R, White PF, Scuderi PE. The efficacy of RS-25259, a long-acting selective 5-HT3 receptor antagonist, for preventing postoperative nausea and vomiting after hysterectomy procedures. *Anesth Analg.* 1998; 87(2):462-467.
372. Tang J, Wang B, White PF, Watcha MF, Qi J, Wender RH. The effect of timing of ondansetron administration on its efficacy, cost-effectiveness, and cost-benefit as a prophylactic antiemetic in the ambulatory setting. *Anesth Analg.* 1998; 86(2):274-282.
373. Tang J, Watcha MF, White PF. A comparison of costs and efficacy of ondansetron and droperidol as prophylactic antiemetic therapy for elective outpatient gynecologic procedures. *Anesth Analg.* 1996; 83(2):304-313.
374. Taylor AM, Rosen M, Diemunsch PA, Thorin D, Houweling PL. A double-blind, parallel-group, placebo-controlled, dose-ranging, multicenter study of intravenous granisetron in the treatment of postoperative nausea and vomiting in patients undergoing surgery with general anesthesia. *J Clin Anesth.* 1997; 9(8):658-663.
375. Teran L, Hawkins JK. The effectiveness of inhalation isopropyl alcohol vs. granisetron for the prevention of postoperative nausea and vomiting. *AANA J.* 2007; 75(6):417-422.
376. Thagaard KS, Steine S, Raeder J. Ondansetron disintegrating tablets of 8 mg twice a day for 3 days did not reduce the incidence of nausea or vomiting after laparoscopic surgery. *Eur J Anaesthesiol.* 2003; 20(2):153-157.
377. Thomas R, Jones N. Prospective randomized, double-blind comparative study of dexamethasone, ondansetron, and ondansetron plus dexamethasone as prophylactic antiemetic therapy in patients undergoing day-case gynaecological surgery. *Br J Anaesth.* 2001; 87(4):588-592.
378. Tramer MR, Sansonetti A, Fuchs-Buder T, Rifat K. Oculocardiac reflex and postoperative vomiting in paediatric strabismus surgery. A randomised controlled trial comparing four anaesthetic techniques. *Acta Anaesthesiol Scand.* 1998; 42(1):117-123.
379. Treschan TA, Zimmer C, Nass C, Stegen B, Esser J, Peters J. Inspired oxygen fraction of 0.8 does not attenuate postoperative nausea and vomiting after strabismus surgery. *Anesthesiology.* 2005; 103(1):6-10.
380. Tseng LH, Liou SC, Chang TC, Tsai SC, Soong YK, Wong SY. A randomized blinded study of the incidence of postoperative nausea and vomiting in women after major gynecologic laparoscopic surgery. *J Minim Invasive Gynecol.* 2006; 13(5):413-417.
381. Tsui SL, Ng KF, Wong LC, Tang GW, Pun TC, Yang JC. Prevention of postoperative nausea and vomiting in gynaecological laparotomies: a comparison of tropisetron and ondansetron. *Anaesth Intensive Care.* 1999; 27(5):471-476.

382. Turhanoglu S, Ozyilmaz MA, Tok D, Olmez G, Cinar FS, Bayhan N. A comparison of the effects of ondansetron with or without dimenhydrinate in the prevention of nausea and vomiting after major gynaecological surgery. *Acta Anaesthesiologica Italica.* 1999; 50(3):193-199.
383. Tzeng JI, Chu KS, Ho ST, Cheng KI, Liu KS, Wang JJ. Prophylactic iv ondansetron reduces nausea, vomiting and pruritus following epidural morphine for postoperative pain control. *Can J Anaesth.* 2003; 50(10):1023-1026.
384. Ulusoy HO, Akturk G, Luleci N, Kalac N, Albayrak D. Prophylactic administration of ondansetron in emergency intraabdominal operations. *Middle East J Anesthesiol.* 1997; 14(1):45-58.
385. Ummenhofer W, Frei FJ, Urwyler A, Kern C, Drewe J. Effects of ondansetron in the prevention of postoperative nausea and vomiting in children. *Anesthesiology.* 1994; 81(4):804-810.
386. Usha RP, Rama RG, Naidu MU, Jagga RP, Ramesh KT, Shobha JC *et al*. Randomised, double blind, placebo controlled study of Ondansetron in female patients undergoing day case surgery. *Journal of Anaesthesiology Clinical Pharmacology.* 1996; 12(1):31-34.
387. Usmani H, Quadir A, Siddiqui RA, Sharma SC. Ondansetron and dexamethasone in middle ear procedures. *Indian J Otolaryngol Head Neck Surg.* 2003; 55(2):97-99.
388. Vale C, Oliveira F, Assuncao J, Fontes-Ribeiro C, Pereira F. Co-administration of ondansetron decreases the analgesic efficacy of tramadol in humans. *Pharmacology.* 2011; 88(3-4):182-187.
389. Veneziani A, Dominaci M, Labardi M, Luca Noto GA, Marini F. Prevention of postoperative nausea and vomiting (PONV) in major gynecological surgery: Comparison of therapeutic regimens. *Acta Anaesthesiologica Italica.* 2006; 57(3-Feb):185-197.
390. Volpe N, Gesini A, Collini S, Grassano MT, Guzzon D, Marino MR *et al*. Single dose ondansetron for prevention of postoperative nausea and vomiting. Results from the Italian Multicentre Ondansetron Study. *DRUG INVEST.* 1994; 8(2):67-72.
391. Wagley C, Hackett C, Haug RH. Single dose ondansetron for prevention of postoperative nausea and vomiting. Results from the Italian Multicentre Ondansetron Study. *J Oral Maxillofac Surg.* 1999; 57(10):1195-1200.
392. Wagner D, Pandit U, Voepel-Lewis T, Weber M. Dolasetron for the prevention of postoperative vomiting in children undergoing strabismus surgery. *Paediatr Anaesth.* 2003; 13(6):522-526.
393. Wagner DS, Gauger V, Chiravuri D, Faust K. Ondansetron oral disintegrating tablets for the prevention of postoperative vomiting in children undergoing strabismus surgery. *Ther Clin Risk Manag.* 2007; 3(4):691-694.
394. Walker JB. Efficacy of single-dose intravenous dolasetron versus ondansetron in the prevention of postoperative nausea and vomiting. *Clin Ther.* 2001; 23(6):932-938.
395. Wang PK, Tsay PJ, Huang CC, Lai HY, Lin PC, Huang SJ *et al*. Comparison of dexamethasone with ondansetron or haloperidol for prevention of patient-controlled analgesia-related postoperative nausea and vomiting: a randomized clinical trial. *World J Surg.* 2012; 36(4):775-781.
396. Warriner CB, Knox D, Belo S, Cole C, Finegan BA, Perreault L. Prophylactic oral dolasetron mesylate reduces nausea and vomiting after abdominal hysterectomy. The Canadian Dolasetron Study Group. *Can J Anaesth.* 1997; 44(11):1167-1173.
397. Watcha MF, Bras PJ, Cieslak GD, Pennant JH. The dose-response relationship of ondansetron in preventing postoperative emesis in pediatric patients undergoing ambulatory surgery. *Anesthesiology.* 1995; 82(1):47-52.
398. Watts SA. A randomized double-blinded comparison of metoclopramide, ondansetron and cyclizine in day-case laparoscopy. *Anaesth Intensive Care.* 1996; 24(5):546-551.
399. Wattwil M, Thorn SE, Lovqvist A, Wattwil L, Gupta A, Liljegren G. Dexamethasone is as effective as ondansetron for the prevention of postoperative nausea and vomiting following breast surgery. *Acta Anaesthesiol Scand.* 2003; 47(7):823-827.
400. White LA, Vanarase M, Brockbank K, Barrett RF. Patient-controlled analgesia and postoperative nausea and vomiting: efficacy of a continuous infusion of ondansetron. *Anaesthesia.* 2001; 56(4):365-369.
401. White PF, Issioui T, Hu J, Jones SB, Coleman JE, Waddle JP *et al*. Comparative efficacy of acustimulation (ReliefBand) versus ondansetron (Zofran) in combination with droperidol for preventing nausea and vomiting. *Anesthesiology.* 2002; 97(5):1075-1081.
402. White PF, Tang J, Hamza MA, Ogunnaike B, Lo M, Wender RH *et al*. The use of oral granisetron versus intravenous ondansetron for antiemetic prophylaxis in patients undergoing laparoscopic surgery: the effect on emetic symptoms and quality of recovery. *Anesth Analg.* 2006; 102(5):1387-1393.
403. White PF, Tang J, Song D, Coleman JE, Wender RH, Ogunnaike B *et al*. Transdermal scopolamine: an alternative to ondansetron and droperidol for the prevention of postoperative and postdischarge emetic symptoms. *Anesth Analg.* 2007; 104(1):92-96.
404. Wig J, Chandrashekharappa KN, Yaddanapudi LN, Nakra D, Mukherjee KK. Effect of prophylactic ondansetron on postoperative nausea and vomiting in patients on preoperative steroids undergoing craniotomy for supratentorial tumors. *J Neurosurg Anesthesiol.* 2007; 19(4):239-242.
405. Wilson AJ, Diemunsch P, Lindeque BG, Scheinin H, Helbo-Hansen HS, Kroeks MV *et al*. Single-dose i.v. granisetron in the prevention of postoperative nausea and vomiting. *Br J Anaesth.* 1996; 76(4):515-518.

406. Wilson EB, Bass CS, Abrameit W, Roberson R, Smith RW. Metoclopramide versus ondansetron in prophylaxis of nausea and vomiting for laparoscopic cholecystectomy. *Am J Surg.* 2001; 181(2):138-141.
407. Woodward DK, Sherry KM, Harrison D. Antiemetic prophylaxis in cardiac surgery: comparison of metoclopramide and ondansetron. *Br J Anaesth.* 1999; 83(6):933-935.
408. Wrench IJ, Ward JE, Walder AD, Hobbs GJ. The prevention of postoperative nausea and vomiting using a combination of ondansetron and droperidol. *Anaesthesia.* 1996; 51(8):776-778.
409. Wu O, Belo SE, Koutsoukos G. Additive anti-emetic efficacy of prophylactic ondansetron with droperidol in out-patient gynecological laparoscopy. *Can J Anaesth.* 2000; 47(6):529-536.
410. Yazigi A, Chalhoub V, Madi-Jebara S, Haddad F, Hayek G. Prophylactic ondansetron is effective in the treatment of nausea and vomiting but not on pruritus after cesarean delivery with intrathecal sufentanil-morphine. *J Clin Anesth.* 2002; 14(3):183-186.
411. Yilmazlar A, Tokat O, Kutlay O, Yilmazlar T, Turker G. Comparison of the efficacy of 2 mg versus 5 mg tropisetron in the management of post-operative nausea and vomiting. *J Int Med Res.* 2001; 29(5):385-388.
412. Yuksek MS, Alici HA, Erdem AF, Cesur M. Comparison of prophylactic anti-emetic effects of ondansetron and dexamethasone in women undergoing day-case gynaecological laparoscopic surgery. *J Int Med Res.* 2003; 31(6):481-488.
413. Yun MJ, Kim YH, Kim AR. Comparison of azasetron and ondansetron for preventing postoperative nausea and vomiting in patients undergoing gynecological laparoscopic surgery. *Yonsei Med J.* 2010; 51(1):88-92.
414. Zarate E, Watcha MF, White PF, Klein KW, Sa Rego M, Stewart DG. A comparison of the costs and efficacy of ondansetron versus dolasetron for antiemetic prophylaxis. *Anesth Analg.* 2000; 90(6):1352-1358.
415. Zomers PJW, Langenberg CJM, Bruijn KMD. Tropisetron for Postoperative Nausea and Vomiting in Patients after Gynaecological Surgery. *Br J Anaesth.* 1993; 71(5):677-680.
416. Akcabay M, Gunaydin B, Mahli A, Karadenizli Y. Ondansetron versus placebo to prevent postoperative nausea and vomiting in patients undergoing thyroidectomy. *Gazi Medical Journal.* 1997; 8:54-57.
417. Ali-Melkkila T, Kanto J, Katevuo R. Tropisetron and metoclopramide in the prevention of postoperative nausea and vomiting. A comparative, placebo controlled study in patients undergoing ophthalmic surgery. *Anaesthesia.* 1996; 51(3):232-235.
418. Alon E, Buchser E, Herrera E, Christiaens F, De Pauw C, Ritter L *et al*. Tropisetron for treating established postoperative nausea and vomiting: a randomized, double-blind, placebo-controlled study. *Anesth Analg.* 1998; 86(3):617-623.
419. Ang C, Habre W, Sims C. Tropisetron reduces vomiting after tonsillectomy in children. *Br J Anaesth.* 1998; 80(6):761-763.
420. Asl ME, Isazadefar K, Mohammadian A, Khoshbaten M. Ondansetron and meperidine prevent postoperative shivering after general anesthesia. *Middle East J Anesthesiol.* 2011; 21(1):67-70.
421. Jensen AB, Christiansen DB, Coulthard K, Wilkins A, Roberts G, Walt JH *et al*. Tropisetron reduces postoperative vomiting in children undergoing tonsillectomy. *Paediatr Anaesth.* 2000; 10(1):69-75.
422. Madenoglu H, Yildiz K, Dogru K, Kurtsoy A, Guler G, Boyaci A. Randomized, double-blinded comparison of tropisetron and placebo for prevention of postoperative nausea and vomiting after supratentorial craniotomy. *J Neurosurg Anesthesiol.* 2003; 15(2):82-86.
423. Mitra D, Ray M, Dutta S, Gupta P, Sarkar A. Efficacy of Ondansetron and Dexamethasone in the Prevention of Postoperative Nausea and Vomiting after Caesarean Section. *Journal of Anaesthesiology Clinical Pharmacology.* 14(4):359-362.
424. Pan AK, Rudra A. Prophylactic single dose interavenous administration of ondansetron in the prevention of postoperative emetic symptoms during spinal anaesthesia for caesarean deliveryIY. *Indian Journal of Anaesthesia.* 2003; 2003(47):3.
425. Purhonen S, Kauko M, Koski EM, Nuutinen L. Comparison of tropisetron, droperidol, and saline in the prevention of postoperative nausea and vomiting after gynecologic surgery. *Anesth Analg.* 1997; 84(3):662-667.
426. Sajedi P, Yaraghi A, Moseli HA. Efficacy of granisetron in preventing postanesthetic shivering. *Acta Anaesthesiol Taiwan.* 2008; 46(4):166-170.
427. Sarkar M, Sarkar A, Dewoolkar L, Charan S. Comparative Study of single dose intravenous ondansetron and metoclopramide as a premedication for prevention of postoperative nausea and vomiting in obstetrical laparoscopic surgery under general anaesthesia. *The Internet Journal of Anesthesiology.* 2007; 13(2).
428. Siddik-Sayyid SM, Aouad MT, Taha SK, Azar MS, Hakki MA, Kaddoum RN *et al*. Does ondansetron or granisetron prevent subarachnoid morphine-induced pruritus after cesarean delivery? *Anesth Analg.* 2007; 104(2):421-424.
429. Tosun Z, Akin A, Dogan H, Boyaci A. A randomized, placebo-controlled trial of a single dose of tropisetron for the prevention of vomiting after strabismus surgery in children. *Mt Sinai J Med.* 2006; 73(8):1106-1111.
430. Wang JJ, Ho ST, Uen YH, Lin MT, Chen KT, Huang JC *et al*. Small-dose dexamethasone reduces nausea and vomiting after laparoscopic cholecystectomy: a comparison of tropisetron with saline. *Anesth Analg.* 2002; 95(1):229-232, table of contents.

431. Wang JJ, Tzeng JI, Ho ST, Chen JY, Chu CC, So EC. The prophylactic effect of tropisetron on epidural morphine-related nausea and vomiting: a comparison of dexamethasone with saline. *Anesth Analg.* 2002; 94(3):749-753; table of contents.
432. Wang JJ, Wang PC, Liu YH, Chien CC. Low-dose dexamethasone reduces nausea and vomiting after tympanomastoid surgery: a comparison of tropisetron with saline. *Am J Otolaryngol.* 2002; 23(5):267-271.
433. Yilmazlar A, Yilmazlar T, Gurpinar E, Korun N, Kutlay O. Anti-emetic efficacy of tropisetron and metoclopramide. *J Int Med Res.* 1996; 24(3):266-270.
434. Cieslak GD, Watcha MF, Phillips MB, Pennant JH. The dose-response relation and cost-effectiveness of granisetron for the prophylaxis of pediatric postoperative emesis. *Anesthesiology.* 1996; 85(5):1076-1085.
435. Holt R, Rask P, Coulthard KP, Sinclair M, Roberts G, Van Der Walt J *et al*. Tropisetron plus dexamethasone is more effective than tropisetron alone for the prevention of postoperative nausea and vomiting in children undergoing tonsillectomy. *Paediatr Anaesth.* 2000; 10(2):181-188.
436. Panda NB, Bharadwaj N, Kapoor P, Chari P, Panda NK. Prevention of nausea and vomiting after middle ear surgery: combination of ondansetron and dexamethasone is the right choice. *J Otolaryngol.* 2004; 33(2):88-92.
437. Wang S, Greco J, Joseph RA, Feuerman M, Malone B. Evaluation of Three 5-HT3 Receptor Antagonists in the Prevention of Postoperative Nausea and Vomiting in Adults. *P&T.* 2005; 30(6):341-353.
438. Srivastava B, Gaur S, Sinha AK, Sayana A, Barshaliya B, Dutt HK. Antiemetic prophylaxis with Granisetron, Ondansetron and Metoclopramide in laparoscopic cholecystectomy: A Comparison. *JARBS.* 2012; 4(4):279-287.
439. Safae M, Safi S. Evaluation of Effect of Ondansetron on Nausea and Vomiting after Elective Cholecystectomy Surgery in Women Older than 40 Years. *Journal of Isfahan Medical School.* 2011; 29(149).
440. Apfel CC, Candiotti K, Gan TJ, Ahmed R, Cox D. Palonosetron or ondansetron for rescue treatment of PONV after failed prophylaxis with ondansetron. *Anesth Analg.* 2011; 112(5S):S16.
441. Samra SK, Williams, B., Ciarralo, C., Turner, C., & Smythe, P. . Lack of effect of droperidol, ondansetron, and dolasetron administration on PONV in a placebo controlled trial. Anesthesiology, 99, A305. 2003.
442. White PF, & Scuderi, P. E. Prevention of postoperative nausea and vomiting (PONV): A dose-ranging study involving palonosetron, a potent 5-HT3 receptor antagonist. Anesthesiology, 103, A703. 2005.
443. Scuderi P, Wetchler B, Sung YF, Mingus M, DuPen S, Claybon L *et al*. Treatment of postoperative nausea and vomiting after outpatient surgery with the 5-HT3 antagonist ondansetron. *Anesthesiology.* 1993; 78(1):15-20.
444. Ebrahim Soltani AR, Mohammadinasab H, Goudarzi M, Arbabi S, Mohammadinasab A, Mohammadinasab F *et al.* Comparing the efficacy of prophylactic p6 acupressure, ondansetron, metoclopramide and placebo in the prevention of vomiting and nausea after strabismus surgery. *Acta Med Iran.* 2011; 49(4):208-212.
445. Oztekin S, Ozzeybek D, Tasdogen A, Kilercik H, Kara HC. Comparison of the antiemetic efficacy of tropisetron and droperidol with patient-given tramadol. *J Int Med Res.* 2003; 31(4):267-271.
446. Gaynes B, Fiscella R, Larson B, Kiesling D. Intravenous ondansetron versus placebo for post-operative nausea and vomiting following cataract surgery. *American Academy of Optometry.* 1996; 170.
447. Gurha P, R.D. K, Sanjay RR. Prophylactic intravenous palonosetron, granisetron and ondansetron in the prevention of post operative nausea and vomiting in laparoscopic surgeries. In. Unpublished; 2012.
448. Dershwitz M, Rosow CE, Di Biase PM, Joslyn AF, Sanderson PE. Ondansetron is effective in decreasing postoperative nausea and vomiting. *Clin Pharmacol Ther.* 1992; 52(1):96-101.
449. Jokela RM, Cakmakkaya OS, Danzeisen O, Korttila KT, Kranke P, Malhotra A *et al*. Ondansetron has similar clinical efficacy against both nausea and vomiting. *Anaesthesia.* 2009; 64(2):147-151.
450. Pearman MH. Single dose intravenous ondansetron in the prevention of postoperative nausea and vomiting. *Anaesthesia.* 1994; 49 Suppl:11-15.

Appendix 3. List of excluded studies from previous systematic reviews

Study	Tang, 2012 (NMA)	Carlisle, 2006 (Cochrane)	Rawlinson, 2012	Reason for exclusion
Levy, 2011	NO	NO	YES	No serotonin receptor antagonist
Feo, 2006	NO	NO	YES	No serotonin receptor antagonist
Zutshi, 2005	NO	NO	YES	No serotonin receptor antagonist
Fujii, 2004	YES	NO	NO	Retracted article
Altunkaya, 2003	NO	YES	NO	No serotonin receptor antagonist
Batra, 2003	NO	YES	NO	No serotonin receptor antagonist
Bisgaard, 2003	NO	YES	NO	No serotonin receptor antagonist
Bouderka, 2003	NO	YES	NO	No serotonin receptor antagonist
Caron, 2003	NO	YES	NO	No relevant comparator
Culebras, 2003	NO	YES	NO	No serotonin receptor antagonist
Eberhart, 2003	NO	YES	NO	No serotonin receptor antagonist
El Shobaki, 2003	NO	YES	NO	Could not obtain full text
Elhakim, 2003	NO	YES	NO	No serotonin receptor antagonist
Fujii, 2003	NO	YES	NO	Retracted article
Grottke, 2003	NO	YES	NO	No serotonin receptor antagonist
Gulhas, 2003	NO	YES	NO	No serotonin receptor antagonist
Günes, 2003	NO	YES	NO	Non-English [Turkish]
Gurses, 2003	NO	YES	NO	No serotonin receptor antagonist
Halvorsen, 2003	NO	YES	NO	No serotonin receptor antagonist
Hanna, 2003	NO	NO	YES	No serotonin receptor antagonist
Ismail, 2003	NO	YES	NO	No serotonin receptor antagonist
Jellish, 2003	NO	YES	NO	No serotonin receptor antagonist
Joris, 2003	NO	YES	NO	No serotonin receptor antagonist
Karabayirh, 2003	NO	YES	NO	Non-English [Turkish]
Kvalsvik, 2003	NO	NO	YES	No serotonin receptor antagonist
Latasch, 2003	NO	YES	NO	No serotonin receptor antagonist
Lee, 2003	NO	YES	NO	No serotonin receptor antagonist
McCartney, 2003	NO	YES	NO	No serotonin receptor antagonist
Memis, 2003	NO	YES	NO	No serotonin receptor antagonist
Nortcliffe, 2003	NO	YES	NO	No serotonin receptor antagonist
Ozcan, 2003	NO	YES	NO	No serotonin receptor antagonist
Piper, 2003	NO	YES	NO	Non-English [German]
Pongrojpaw, 2003	NO	YES	NO	No serotonin receptor antagonist
Purhonen, 2003	NO	YES	NO	No serotonin receptor antagonist
Quiroga, 2003	NO	YES	NO	No serotonin receptor antagonist
Rimaitis, 2003	NO	NO	YES	No serotonin receptor antagonist
Sahjpaul, 2003	NO	YES	NO	No serotonin receptor antagonist
Schultz, 2003	NO	YES	NO	No serotonin receptor antagonist
Shah, 2003	NO	YES	NO	No serotonin receptor antagonist
Sites, 2003	NO	YES	NO	No serotonin receptor antagonist
Turan, 2003	NO	YES	NO	No serotonin receptor antagonist
Yegin, 2003	NO	YES	NO	No serotonin receptor antagonist
Yelken, 2003	NO	YES	NO	Non-English [Turkish]
Purhonen, 2003b	NO	YES	NO	No serotonin receptor antagonist

Abdulatif, 2002	NO	YES	NO	No serotonin receptor antagonist
Abou Zeid, 2002	NO	YES	NO	Retracted article
Alexander, 2002	NO	NO	YES	No serotonin receptor antagonist
Apfel, 2002	NO	YES	NO	No serotonin receptor antagonist
Awad, 2002	NO	YES	NO	No relevant comparator
Barros-de, 2002	NO	YES	NO	No serotonin receptor antagonist
Bock, 2002	NO	YES	NO	No serotonin receptor antagonist
Bonhomme, 2002	NO	YES	NO	No serotonin receptor antagonist
Carli, 2002	NO	NO	YES	No serotonin receptor antagonist
Cook-Sather, 2002	NO	YES	NO	No serotonin receptor antagonist
Cote, 2002	NO	YES	NO	No serotonin receptor antagonist
Fillinger, 2002	NO	YES	NO	No serotonin receptor antagonist
Fournier, 2002	NO	YES	NO	No serotonin receptor antagonist
Fujii, 2002	NO	YES	NO	Retracted article
Fujii, 2002b	NO	YES	NO	Retracted article
Fujii, 2002c	NO	YES	NO	Retracted article
Fujii, 2002e	NO	YES	NO	No serotonin receptor antagonist
Fujii, 2002f	NO	YES	NO	Retracted article
Ganem, 2002	NO	YES	NO	No serotonin receptor antagonist
Giannoni, 2002	NO	YES	NO	No serotonin receptor antagonist
Hammas, 2002	NO	YES	NO	No serotonin receptor antagonist
Jeffs, 2002	NO	YES	NO	No serotonin receptor antagonist
Kaya, 2002	NO	YES	NO	No serotonin receptor antagonist
Lee, 2002	NO	YES	NO	No serotonin receptor antagonist
Nakata, 2002	NO	YES	NO	No serotonin receptor antagonist
Oddby-Muhrbeck, 2002	NO	YES	NO	No serotonin receptor antagonist
Omais, 2002	NO	YES	NO	No serotonin receptor antagonist
Oshima, 2002	NO	YES	NO	No serotonin receptor antagonist
Pang, 2002	NO	YES	NO	No serotonin receptor antagonist
Piper, 2002	YES	YES	NO	Retracted article
Rusch, 2002	NO	YES	NO	No serotonin receptor antagonist
Sanansilp, 2002	NO	YES	NO	No serotonin receptor antagonist
Song, 2002	NO	YES	NO	No serotonin receptor antagonist
Steinberg, 2002	NO	NO	YES	No serotonin receptor antagonist
Tosun, 2002	NO	YES	NO	Non-English [Turkish]
Tuncer, 2002	NO	YES	NO	No serotonin receptor antagonist
Tzeng, 2002	NO	YES	NO	No serotonin receptor antagonist
Uerpairojkit, 2002	NO	YES	NO	No serotonin receptor antagonist
Unlugenc, 2002	NO	YES	NO	No serotonin receptor antagonist
Vimlati, 2002	NO	YES	NO	Non-English [Hungarian]
Wang, 2002b	NO	YES	NO	No serotonin receptor antagonist
Wang, 2002d	NO	YES	NO	Non-English [Mandarin]
Wang, 2002f	NO	YES	NO	Non-English [Mandarin]
Wu, 2002	NO	YES	NO	No serotonin receptor antagonist
Yin, 2002	NO	YES	NO	No serotonin receptor antagonist
Akkaya, 2001	NO	YES	NO	Non-English [Turkish]
Aouad, 2001	NO	YES	NO	No serotonin receptor antagonist
Carli, 2001	NO	NO	YES	No serotonin receptor antagonist
Celik, 2001	NO	YES	NO	Non-English [Turkish]

Chen, 2001	NO	YES	NO	No relevant comparator
Chow, 2001	NO	NO	YES	No serotonin receptor antagonist
Coloma, 2001	NO	YES	NO	No serotonin receptor antagonist
Danner, 2001	NO	YES	NO	Non-English [German]
Eberhart, 2001	NO	YES	NO	No serotonin receptor antagonist
Fazi, 2001	NO	YES	NO	No serotonin receptor antagonist
Fonseca, 2001	NO	YES	NO	No serotonin receptor antagonist
Fujii, 2001	NO	YES	NO	No serotonin receptor antagonist
Fujii, 2001b	NO	YES	NO	Retracted article
Fujii, 2001c	NO	YES	NO	Retracted article
Fujii, 2001d	NO	YES	NO	Retracted article
Fujii, 2001e	YES	YES	NO	Retracted article
Fujii, 2001f	YES	YES	NO	Retracted article
Fujii, 2001g	NO	YES	NO	Retracted article
Gentili, 2001	NO	YES	NO	No serotonin receptor antagonist
Handa, 2001	NO	YES	NO	No serotonin receptor antagonist
Hechler, 2001	NO	YES	NO	No serotonin receptor antagonist
Hildyard, 2001	NO	YES	NO	No serotonin receptor antagonist
Ho, 2001	NO	YES	NO	No serotonin receptor antagonist
Huang, 2001	NO	YES	NO	No serotonin receptor antagonist
Lee, 2001	NO	YES	NO	No serotonin receptor antagonist
Liu, 2001	NO	YES	NO	No serotonin receptor antagonist
Lovstad, 2001	NO	YES	NO	No serotonin receptor antagonist
Mimoz, 2001	NO	NO	YES	No serotonin receptor antagonist
Papadimitriou, 2001	NO	YES	NO	No relevant comparator
Paulsen, 2001	NO	NO	YES	No serotonin receptor antagonist
Piper, 2001	YES	YES	NO	Retracted article
Ramirez, 2001	NO	YES	NO	No serotonin receptor antagonist
Schulz-Stubner, 2001	NO	YES	NO	No serotonin receptor antagonist
Siddik, 2001	NO	NO	YES	No serotonin receptor antagonist
Tan, 2001	NO	YES	NO	No serotonin receptor antagonist
Ting, 2001	NO	YES	NO	No serotonin receptor antagonist
Visser, 2001	NO	NO	YES	No serotonin receptor antagonist
Wang, 2001	NO	YES	NO	No serotonin receptor antagonist
Zatman, 2001	NO	YES	NO	No serotonin receptor antagonist
Aubrun, 2000	NO	NO	YES	No serotonin receptor antagonist
Busoni, 2000	NO	YES	NO	No serotonin receptor antagonist
Dillier, 2000	NO	YES	NO	Non-English [German]
Eberhart, 2000	NO	YES	NO	No serotonin receptor antagonist
Eberhart, 2000b	NO	YES	NO	No serotonin receptor antagonist
Frank, 2000	NO	YES	NO	No serotonin receptor antagonist
Fujii, 2000	NO	YES	NO	Retracted article
Fujii, 2000b	NO	YES	NO	Retracted article
Fujii, 2000c	NO	YES	NO	Retracted article
Hagemann, 2000	NO	YES	NO	No serotonin receptor antagonist
Kotake, 2000	NO	YES	NO	No serotonin receptor antagonist
Madan, 2000	NO	YES	NO	No relevant comparator
Mann, 2000	NO	NO	YES	No serotonin receptor antagonist
Mayson, 2000	NO	YES	NO	No serotonin receptor antagonist
Nawasreh, 2000	NO	YES	NO	No serotonin receptor antagonist
Palme, 2000	NO	YES	NO	No serotonin receptor antagonist

Pérez, 2000	NO	YES	NO	Non-English [Spanish]
Philip, 2000	YES	NO	NO	Not a relevant study design
Rao, 2000	NO	NO	YES	No serotonin receptor antagonist
Schlager, 2000	NO	YES	NO	No serotonin receptor antagonist
Tan, 2000	NO	YES	NO	No serotonin receptor antagonist
Tzeng, 2000	NO	YES	NO	No serotonin receptor antagonist
Tzeng, 2000b	NO	YES	NO	No serotonin receptor antagonist
Wang, 2000	NO	YES	YES	No serotonin receptor antagonist
Wang, 2000b	NO	YES	NO	No serotonin receptor antagonist
Wang, 2000c	NO	YES	NO	No serotonin receptor antagonist
Welters, 2000	NO	YES	NO	No serotonin receptor antagonist
Welters, 2000b	NO	YES	NO	No serotonin receptor antagonist
Ascari, 1999	NO	YES	NO	CA, >10 years
Badaoui, 1999	NO	YES	NO	Non-English [French]
Eberhart, 1999	NO	YES	NO	No serotonin receptor antagonist
Eberhart, 1999b	NO	YES	NO	No serotonin receptor antagonist
Eberhart, 1999c	NO	YES	NO	No serotonin receptor antagonist
Eberhart, 1999d	NO	YES	NO	Non-English [German]
Eberhart, 1999e	NO	YES	NO	No serotonin receptor antagonist
Fujii, 1999	NO	YES	NO	Retracted article
Fujii, 1999b	NO	YES	NO	Retracted article
Fujii, 1999c	NO	YES	NO	Retracted article
Fujii, 1999d	NO	YES	NO	Retracted article
Fujii, 1999e	NO	YES	NO	Retracted article
Fujii, 1999f	NO	YES	NO	Retracted article
Fujii, 1999g	NO	YES	NO	Retracted article
Fujii, 1999h	NO	YES	NO	Retracted article
Fujii, 1999i	NO	YES	NO	Retracted article
Fujii, 1999j	NO	YES	NO	Retracted article
Fujii, 1999k	NO	YES	NO	Retracted article
Fujii, 1999l	NO	YES	NO	Retracted article
Fujii, 1999m	NO	YES	NO	Retracted article
Fujii, 1999n	NO	YES	NO	Retracted article
Goyagi, 1999	NO	YES	NO	No serotonin receptor antagonist
Greif, 1999	NO	YES	NO	No serotonin receptor antagonist
Honkavaara, 1999	NO	YES	NO	No serotonin receptor antagonist
Ilbeigi, 1999	NO	YES	NO	CA, >10 years
Jakobsson, 1999	NO	YES	NO	Not a relevant study design
Kokinsky, 1999	NO	YES	NO	No serotonin receptor antagonist
Kranke, 1999	NO	YES	NO	No serotonin receptor antagonist
Lepouse, 1999	NO	YES	NO	CA, >10 years
Liu, 1999	NO	YES	NO	No serotonin receptor antagonist
Lim, 1999	NO	YES	NO	No serotonin receptor antagonist
Morin, 1999	NO	YES	NO	No serotonin receptor antagonist
Movinsky, 1999	NO	YES	NO	No serotonin receptor antagonist
Parks, 1999	NO	YES	NO	CA, >10 years
Rusch, 1999	NO	YES	NO	Non-English [Italian]
Triem, 1999	NO	YES	NO	Non-English [German]
Vosdoganis, 1999	NO	YES	NO	No serotonin receptor antagonist
Wang, 1999	NO	YES	NO	No serotonin receptor antagonist
Wang, 1999b	NO	YES	NO	No serotonin receptor antagonist
Wang, 1999c	NO	YES	NO	No serotonin receptor antagonist

Williams, 1999	NO	YES	NO	No serotonin receptor antagonist
Kranke, 1999b	NO	YES	NO	CA, >10 years
Apfel, 1998	NO	YES	NO	No serotonin receptor antagonist
Badaoui, 1998	NO	YES	NO	Non-English [French]
Biedler, 1998	NO	YES	NO	Non-English [German]
Bilgin, 1998	NO	YES	NO	Non-English [Turkish]
Chung, 1998	NO	YES	NO	No serotonin receptor antagonist
Fortney, 1998	NO	YES	NO	Not a relevant study design
Fujii, 1998	NO	YES	NO	Retracted article
Fujii, 1998b	NO	YES	NO	Retracted article
Fujii, 1998c	NO	YES	NO	Retracted article
Fujii, 1998d	NO	YES	NO	Retracted article
Fujii, 1998e	NO	YES	NO	Retracted article
Fujii, 1998f	NO	YES	NO	Retracted article
Fujii, 1998g	NO	YES	NO	Retracted article
Fujii, 1998h	NO	YES	NO	Retracted article
Fujii, 1998i	NO	YES	NO	Retracted article
Fujii, 1998j	NO	YES	NO	Retracted article
Fujii, 1998k	NO	YES	NO	Retracted article
Fujii, 1998l	NO	YES	NO	Retracted article
Fujii, 1998m	NO	YES	NO	Retracted article
Fujii, 1998n	YES	YES	NO	Retracted article
Fujii, 1998o	YES	YES	NO	Retracted article
Fujii, 1998q	NO	YES	NO	Retracted article
Fujii, 1998r	NO	YES	NO	Retracted article
Fujii, 1998s	NO	YES	NO	Retracted article
Fujii, 1998t	YES	YES	NO	Retracted article
Fujii, 1998u	NO	YES	NO	Retracted article
Gulec, 1998	NO	YES	NO	No serotonin receptor antagonist
Kathirvel, 1998	NO	YES	NO	No serotonin receptor antagonist
Lamond, 1998	NO	YES	NO	No serotonin receptor antagonist
Lauretti, 1998	NO	YES	NO	No serotonin receptor antagonist
López Herrera, 1998	NO	YES	NO	Non-English [Spanish]
Nelskyla, 1998	NO	YES	NO	No serotonin receptor antagonist
Pan, 1998	NO	YES	NO	No serotonin receptor antagonist
Pappas, 1998	NO	YES	NO	No serotonin receptor antagonist
Rothenberg, 1998	NO	YES	NO	No serotonin receptor antagonist
Sanansilp, 1998	NO	YES	NO	No serotonin receptor antagonist
Serrano, 1998	NO	YES	NO	Non-English [Spanish]
Shende, 1998	NO	YES	NO	No serotonin receptor antagonist
Steinbrook, 1998	NO	YES	NO	No relevant comparator
Tan, 1998	NO	YES	NO	No serotonin receptor antagonist
Visalyaputra, 1998	NO	YES	NO	No serotonin receptor antagonist
Altintas, 1997	NO	YES	NO	No serotonin receptor antagonist
Cosar, 1997	NO	YES	NO	Non-English [Turkish]
D’Angelo, 1997	NO	YES	NO	CA, >10 years
Flores, 1997	NO	YES	NO	Non-English [Spanish]
Fujii, 1997	YES	YES	NO	Retracted article
Fujii, 1997b	NO	YES	NO	Retracted article
Fujii, 1997c	YES	YES	NO	Retracted article

Fujii, 1997d	YES	YES	NO	Retracted article
Fujii, 1997e	NO	YES	NO	Retracted article
Fujii, 1997f	YES	YES	NO	Retracted article
Fujii, 1997g	YES	YES	NO	Retracted article
Fujii, 1997h	YES	NO	NO	Retracted article
Fujii, 1997i	NO	YES	NO	Retracted article
Gan, 1997	NO	YES	NO	No serotonin receptor antagonist
Goode, 1997	NO	YES	NO	CA, >10 years
Hovorka, 1997	NO	YES	NO	No serotonin receptor antagonist
Hunting, 1997	NO	YES	NO	CA, >10 years
Jellish, 1997	NO	YES	NO	Duplicate of Jellish, 1998
Khalil, 1997	NO	YES	NO	No serotonin receptor antagonist
Koivuranta, 1997	NO	YES	NO	No relevant comparator
Lauretti, 1997	NO	YES	NO	No serotonin receptor antagonist
Lauretti, 1997b	NO	YES	NO	No serotonin receptor antagonist
Lauretti, 1997c	NO	YES	NO	No serotonin receptor antagonist
Lawrence, 1997	NO	YES	NO	No serotonin receptor antagonist
Lessard, 1997	NO	YES	NO	No serotonin receptor antagonist
Loo, 1997	NO	YES	NO	No serotonin receptor antagonist
Mansfield, 1997	NO	YES	NO	Not a relevant study design
Mikawa, 1997	NO	YES	NO	No serotonin receptor antagonist
Najnigier, 1997	NO	YES	NO	Non-English [Polish]
Ng, 1997	NO	YES	NO	No serotonin receptor antagonist
Ozalp, 1997	NO	YES	NO	Non-English [Turkish]
Paech, 1997	NO	YES	NO	No serotonin receptor antagonist
Ploner, 1997	NO	YES	NO	Non-English [German]
Richardson, 1997	NO	YES	NO	No serotonin receptor antagonist
Splinter, 1997	NO	YES	NO	No serotonin receptor antagonist
Splinter, 1997b	NO	YES	NO	No serotonin receptor antagonist
Sun, 1997	NO	YES	NO	CA, >10 years
Sun, 1997b	NO	YES	NO	No serotonin receptor antagonist
TerRiet, 1997	NO	YES	NO	CA, >10 years
April, 1996	NO	YES	NO	No serotonin receptor antagonist
Butrón, 1996	NO	YES	NO	No serotonin receptor antagonist
Chelly, 1996	NO	YES	NO	CA, >10 years
Cozanitis, 1996	NO	YES	NO	No serotonin receptor antagonist
Eberhart, 1996	NO	YES	NO	No serotonin receptor antagonist
Foster, 1996	NO	YES	NO	No serotonin receptor antagonist
Fujii, 1996	NO	YES	NO	Retracted article
Fujii, 1996	NO	YES	NO	Retracted article
Fujii, 1996	YES	YES	NO	Retracted article
Fujii, 1996	NO	YES	NO	Retracted article
Fujii, 1996	NO	YES	NO	Retracted article
Gilliland, 1996	NO	YES	NO	No serotonin receptor antagonist
Goyagi, 1996	NO	YES	NO	No serotonin receptor antagonist
Grebenik, 1996	NO	YES	NO	No serotonin receptor antagonist
Guard, 1996	NO	YES	NO	No serotonin receptor antagonist
Honkavaara, 1996	NO	YES	NO	No serotonin receptor antagonist
Huston, 1996	NO	YES	NO	CA, >10 years
Kaufman, 1996	NO	YES	NO	CA, >10 years
Khalil, 1996	NO	YES	NO	No serotonin receptor antagonist

Klahsen, 1996	NO	YES	NO	No serotonin receptor antagonist
Laraki, 1996	NO	YES	NO	No serotonin receptor antagonist
Lerman, 1996	NO	YES	NO	No relevant outcome
Maltepe, 1996	NO	YES	NO	Non-English [Turkish]
Mayzner-Zawadzka, 1996	NO	YES	NO	CA, >10 years
McAllister, 1996	NO	YES	NO	CA, >10 years
McKenzie	NO	YES	NO	No relevant comparator
Miles, 1996	NO	YES	NO	Not a relevant study design
Mjahed, 1996	NO	YES	NO	Non-English [French]
Park, 1996	NO	YES	NO	No serotonin receptor antagonist
Pascucci, 1996	NO	YES	NO	Non-English [Italian]
Pertusa, 1996	NO	YES	NO	Non-English [Spanish]
Russell, 1996	NO	YES	NO	No serotonin receptor antagonist
Saur, 1996	NO	YES	NO	Non-English [German]
Splinter, 1996	NO	YES	NO	No serotonin receptor antagonist
Steinbrook, 1996	NO	YES	NO	No relevant comparator
Tom, 1996	NO	YES	NO	No serotonin receptor antagonist
Top, 1996	NO	YES	NO	Non-English [Turkish]
Trakya, 1996	NO	YES	NO	Non-English [Turkish]
Unseld, 1996	NO	YES	NO	No serotonin receptor antagonist
Valentine, 1996	NO	YES	NO	No serotonin receptor antagonist
Vener, 1996	NO	YES	NO	No serotonin receptor antagonist
Wagner, 1996	NO	YES	NO	No serotonin receptor antagonist
Arfeen, 1995	NO	YES	NO	No serotonin receptor antagonist
Borgeat, 1995	NO	YES	NO	CA, >10 years
Chisakuta, 1995	NO	YES	NO	No serotonin receptor antagonist
Freedman, 1995	NO	YES	NO	No serotonin receptor antagonist
Fujii, 1995	NO	YES	NO	Retracted article
Gan, 1995	NO	YES	NO	No serotonin receptor antagonist
Godsiff, 1995	NO	YES	NO	No serotonin receptor antagonist
Grace, 1995	NO	YES	NO	No serotonin receptor antagonist
Honkavaara, 1995	NO	YES	NO	No serotonin receptor antagonist
Jellish, 1995	NO	YES	NO	No serotonin receptor antagonist
Katayama, 1995	NO	YES	NO	No serotonin receptor antagonist
Kymer, 1995	NO	YES	NO	No serotonin receptor antagonist
Le Roy, 1995	NO	YES	NO	CA, >10 years
Lunn, 1995	NO	YES	NO	No serotonin receptor antagonist
Martínez, 1995	NO	YES	NO	No serotonin receptor antagonist
Martins, 1995	NO	YES	NO	Non-English [Spanish]
McKenzie, 1995	NO	YES	NO	No serotonin receptor antagonist
Mikawa, 1995	NO	YES	NO	No serotonin receptor antagonist
Moerman, 1995	NO	YES	NO	No serotonin receptor antagonist
Moscovici, 1995	NO	YES	NO	No serotonin receptor antagonist
Polati, 1995	NO	YES	NO	Non-English [Italian]
Roberts, 1995	NO	YES	NO	No serotonin receptor antagonist
Rodola, 1995	NO	YES	NO	No serotonin receptor antagonist
Rohling, 1995	NO	YES	NO	CA, >10 years
Splinter, 1995	NO	YES	NO	No serotonin receptor antagonist
Sun, 1995	NO	YES	NO	CA, >10 years

Tarkkila, 1995	NO	YES	NO	No serotonin receptor antagonist
Tur, 1995	NO	YES	NO	Non-English [Turkish]
Turkoglu, 1995	NO	YES	NO	No serotonin receptor antagonist
van den Berg, 1995	NO	YES	NO	CA, >10 years
Walder, 1995	NO	YES	NO	No serotonin receptor antagonist
Watcha, 1995	NO	YES	NO	No serotonin receptor antagonist
Fujii, 1995b	NO	YES	NO	Retracted article
Alon, 1994	NO	YES	NO	Non-English [German]
Barrow, 1994	NO	YES	NO	No serotonin receptor antagonist
Benhamou, 1994	NO	YES	NO	No serotonin receptor antagonist
Boeke, 1994	NO	YES	NO	No serotonin receptor antagonist
Brady, 1994	NO	YES	NO	No serotonin receptor antagonist
Calamandrei, 1994	NO	YES	NO	Non-English [French]
Carr, 1994	NO	YES	NO	CA, >10 years
Ding, 1994	NO	YES	NO	No serotonin receptor antagonist
Doenicke, 1994	NO	YES	NO	No serotonin receptor antagonist
Doyle, 1994	NO	YES	NO	No serotonin receptor antagonist
Elliott, 1994	NO	YES	NO	No serotonin receptor antagonist
Foss, 1994	NO	YES	NO	No serotonin receptor antagonist
Fujii, 1994	NO	YES	NO	Retracted article
Guldogus, 1994	NO	YES	NO	Non-English [Turkish]
Harti, 1994	NO	YES	NO	No serotonin receptor antagonist
Honkavaara, 1994	NO	YES	NO	No serotonin receptor antagonist
Iannuzzi, 1994	NO	YES	NO	Non-English [Italian]
Kafali, 1994	NO	YES	NO	Non-English [Turkish]
Knudsen, 1994	NO	YES	NO	No serotonin receptor antagonist
Lewis, 1994	NO	YES	NO	No serotonin receptor antagonist
Mecklem, 1994	NO	YES	NO	No serotonin receptor antagonist
Reinhart, 1994	NO	YES	NO	No serotonin receptor antagonist
Rust, 1994	NO	YES	NO	Not a relevant study design
Sandhya, 1994	NO	YES	NO	No serotonin receptor antagonist
Sohi, 1994	NO	YES	NO	No serotonin receptor antagonist
Spadafora, 1994	NO	YES	NO	CA, >10 years
Splinter, 1994	NO	YES	NO	No serotonin receptor antagonist
Stead, 1994	NO	YES	NO	No serotonin receptor antagonist
Tokat, 1994	NO	YES	NO	Non-English [Turkish]
Tur, 1994	NO	YES	NO	Non-English [Turkish]
Walder, 1994	NO	YES	NO	No serotonin receptor antagonist
Wildersmith, 1994	NO	YES	NO	No serotonin receptor antagonist
Fujii, 1994b	YES	YES	NO	Retracted article
Baxendale, 1993	NO	YES	NO	No serotonin receptor antagonist
Beattie, 1993	NO	YES	NO	No serotonin receptor antagonist
Bouly, 1993	NO	YES	NO	Non-English [French]
Cade, 1993	NO	YES	NO	No serotonin receptor antagonist
Conroy, 1993	NO	YES	NO	CA, >10 years
Fogarty, 1993	NO	YES	NO	No serotonin receptor antagonist
Geiger, 1993	NO	YES	NO	CA, >10 years
Horta, 1993	NO	YES	NO	No serotonin receptor antagonist

Hyrkas, 1993	NO	YES	NO	No serotonin receptor antagonist
Joshi, 1993	NO	YES	NO	No serotonin receptor antagonist
Kandler, 1993	NO	YES	NO	No serotonin receptor antagonist
Karlsson, 1993	NO	YES	NO	No serotonin receptor antagonist
Klockgether, 1993	NO	YES	NO	No serotonin receptor antagonist
Lawhorn, 1993	NO	YES	NO	CA, >10 years
Patterson, 1993	NO	YES	NO	No serotonin receptor antagonist
Pendeville, 1993	NO	YES	NO	No serotonin receptor antagonist
Phillips, 1993	NO	YES	NO	No serotonin receptor antagonist
Pitkanen, 1993	NO	YES	NO	Not a relevant study design
Sharma, 1993	NO	YES	NO	No serotonin receptor antagonist
Tezcan, 1993	NO	YES	NO	No serotonin receptor antagonist
Alon, 1993b	NO	YES	NO	CA, >10 years
Lawhorn, 1993b	NO	YES	NO	CA, >10 years
Bouly, 1992	NO	YES	NO	Non-English [French]
Carabine, 1992	NO	YES	NO	No serotonin receptor antagonist
Friesen, 1992	NO	YES	NO	No serotonin receptor antagonist
Khalil, 1992	NO	YES	NO	No serotonin receptor antagonist
Klockgether, 1992	NO	YES	NO	No serotonin receptor antagonist
Kumar, 1992	NO	YES	NO	No serotonin receptor antagonist
Lin, 1992	NO	YES	NO	No serotonin receptor antagonist
Migliavacca, 1992	NO	YES	NO	No serotonin receptor antagonist
Muñoz, 1992	NO	YES	NO	No serotonin receptor antagonist
Parnis, 1992	NO	YES	NO	No serotonin receptor antagonist
Salmenpera, 1992	NO	YES	NO	No serotonin receptor antagonist
Semple, 1992	NO	YES	NO	No serotonin receptor antagonist
Blanc, 1991	NO	YES	NO	No serotonin receptor antagonist
Brown, 1991	NO	YES	NO	No serotonin receptor antagonist
Campbell, 1991	NO	YES	NO	No serotonin receptor antagonist
Harris, 1991	NO	YES	NO	No serotonin receptor antagonist
Horimoto, 1991	NO	YES	NO	No serotonin receptor antagonist
Kraus, 1991	NO	YES	NO	No serotonin receptor antagonist
Lim, 1991	NO	YES	NO	No serotonin receptor antagonist
Patterson, 1991	NO	YES	NO	No serotonin receptor antagonist
Rothenberg, 1991	NO	YES	NO	No serotonin receptor antagonist
Scholtes, 1991	NO	YES	NO	No serotonin receptor antagonist
Stromberg, 1991	NO	YES	NO	No serotonin receptor antagonist
Watcha, 1991	NO	YES	NO	No serotonin receptor antagonist
Whalley, 1991	NO	YES	NO	No serotonin receptor antagonist
Anderson, 1990	NO	YES	NO	No serotonin receptor antagonist
Bone, 1990	NO	YES	NO	No serotonin receptor antagonist
Broadman, 1990	NO	YES	NO	No serotonin receptor antagonist
Campbell, 1990	NO	YES	NO	No serotonin receptor antagonist
Jorgensen, 1990	NO	YES	NO	No serotonin receptor antagonist
Koski, 1990	NO	YES	NO	No serotonin receptor antagonist
Larsson, 1990	NO	YES	NO	No serotonin receptor antagonist

Ostman, 1990	NO	YES	NO	No serotonin receptor antagonist
van der Walt, 1990	NO	YES	NO	No serotonin receptor antagonist
Christensen, 1989	NO	YES	NO	No serotonin receptor antagonist
Cramb, 1989	NO	YES	NO	No serotonin receptor antagonist
Loper, 1989	NO	YES	NO	No serotonin receptor antagonist
Schettini, 1989	NO	YES	NO	No serotonin receptor antagonist
Tripple, 1989	NO	YES	NO	No serotonin receptor antagonist
Wilkinson, 1989	NO	YES	NO	No serotonin receptor antagonist
Wilkinson, 1989b	NO	YES	NO	No serotonin receptor antagonist
Booij, 1988	NO	YES	NO	No serotonin receptor antagonist
Diamond, 1988	NO	YES	NO	No serotonin receptor antagonist
Gougeon, 1988	NO	YES	NO	No serotonin receptor antagonist
King, 1988	NO	YES	NO	No serotonin receptor antagonist
Larsson, 1988	NO	YES	NO	No serotonin receptor antagonist
Mathia, 1988	NO	YES	NO	No serotonin receptor antagonist
Miller, 1988	NO	YES	NO	No serotonin receptor antagonist
Nicolson, 1988	NO	YES	NO	No serotonin receptor antagonist
Simpson, 1988	NO	YES	NO	No serotonin receptor antagonist
Tigerstedt, 1988	NO	YES	NO	No serotonin receptor antagonist
Vollmer, 1988	NO	YES	NO	No serotonin receptor antagonist
Alon, 1987	NO	YES	NO	No serotonin receptor antagonist
Chapa, 1987	NO	YES	NO	No serotonin receptor antagonist
Eustis, 1987	NO	YES	NO	No serotonin receptor antagonist
Imbeloni, 1987	NO	YES	NO	No serotonin receptor antagonist
Martin, 1987	NO	YES	NO	No serotonin receptor antagonist
Millar, 1987	NO	YES	NO	No serotonin receptor antagonist
Schuh, 1987	NO	YES	NO	No serotonin receptor antagonist
Tolksdorf, 1987	NO	YES	NO	No serotonin receptor antagonist
van den Berg, 1987	NO	YES	NO	No serotonin receptor antagonist
van der Walt, 1987	NO	YES	NO	No serotonin receptor antagonist
Chestnutt, 1986	NO	YES	NO	No serotonin receptor antagonist
Clyburn, 1986	NO	YES	NO	No serotonin receptor antagonist
Hardy, 1986	NO	YES	NO	No serotonin receptor antagonist
Kauste, 1986	NO	YES	NO	No serotonin receptor antagonist
Madej, 1986	NO	YES	NO	No serotonin receptor antagonist
Madej, 1986b	NO	YES	NO	No serotonin receptor antagonist
Mogensen, 1986	NO	YES	NO	No serotonin receptor antagonist
Pandit, 1986	NO	YES	NO	No serotonin receptor antagonist
Ponnudurai, 1986	NO	YES	NO	No serotonin receptor antagonist
Tolksdorf, 1986	NO	YES	NO	No serotonin receptor antagonist
Uppington, 1986	NO	YES	NO	No serotonin receptor antagonist
Briggs, 1985	NO	YES	NO	No serotonin receptor antagonist
Duarte, 1985	NO	YES	NO	No serotonin receptor antagonist
Hammond, 1985	NO	YES	NO	No serotonin receptor antagonist
Korttila, 1985	NO	YES	NO	No serotonin receptor antagonist
Paul, 1985	NO	YES	NO	No serotonin receptor antagonist

Tolksdorf, 1985	NO	YES	NO	No serotonin receptor antagonist
Valanne, 1985	NO	YES	NO	No serotonin receptor antagonist
Waldmann, 1985	NO	YES	NO	No serotonin receptor antagonist
Layfield, 1984	NO	YES	NO	No serotonin receptor antagonist
Lazar, 1984	NO	YES	NO	No serotonin receptor antagonist
O'Donovan, 1984	NO	YES	NO	No serotonin receptor antagonist
Abramowitz, 1983	NO	YES	NO	No serotonin receptor antagonist
Chan, 1983	NO	YES	NO	No serotonin receptor antagonist
Davies, 1983	NO	YES	NO	No serotonin receptor antagonist
Guldager, 1983	NO	YES	NO	No serotonin receptor antagonist
Alon, 1982	NO	YES	NO	No serotonin receptor antagonist
McKenzie, 1982	NO	YES	NO	No serotonin receptor antagonist
Mortensen, 1982	NO	YES	NO	No serotonin receptor antagonist
Peters, 1982	NO	YES	NO	No serotonin receptor antagonist
Karhunen, 1981	NO	YES	NO	No serotonin receptor antagonist
Mattila, 1981	NO	YES	NO	No serotonin receptor antagonist
Rita, 1981	NO	YES	NO	No serotonin receptor antagonist
Mattila, 1981b	NO	YES	NO	No serotonin receptor antagonist
Diamond, 1980	NO	YES	NO	No serotonin receptor antagonist
Hovi-Viander, 1980	NO	YES	NO	No serotonin receptor antagonist
Boulanger, 1979	NO	YES	NO	No serotonin receptor antagonist
Cooke, 1979	NO	YES	NO	No serotonin receptor antagonist
Hernández-Palazón, 1979	NO	NO	YES	No serotonin receptor antagonist
Korttila, 1979a	NO	YES	NO	No serotonin receptor antagonist
Mattila, 1979b	NO	YES	NO	No serotonin receptor antagonist
Mattila, 1979c	NO	YES	NO	No serotonin receptor antagonist
Richardson, 1979	NO	YES	NO	No serotonin receptor antagonist
Wilson, 1979	NO	YES	NO	No serotonin receptor antagonist
Dodson, 1978	NO	YES	NO	No serotonin receptor antagonist
Iwamoto, 1978	NO	YES	NO	No serotonin receptor antagonist
Fassolt, 1977	NO	YES	NO	No serotonin receptor antagonist
Fozard, 1977	NO	YES	NO	No serotonin receptor antagonist
Galloon, 1977	NO	YES	NO	No serotonin receptor antagonist
Winning, 1977	NO	YES	NO	No serotonin receptor antagonist
Alsner, 1976	NO	YES	NO	No serotonin receptor antagonist
Prescott, 1976	NO	YES	NO	No serotonin receptor antagonist
Dundee, 1975	NO	YES	NO	No serotonin receptor antagonist
Loach, 1975	NO	YES	NO	No serotonin receptor antagonist
Geens, 1974	NO	YES	NO	No serotonin receptor antagonist
Mattila, 1974	NO	YES	NO	No serotonin receptor antagonist
Zsigmond, 1974	NO	YES	NO	No serotonin receptor antagonist
Boyd, 1973	NO	YES	NO	No serotonin receptor antagonist
Wilson, 1973	NO	YES	NO	No serotonin receptor antagonist
Shah, 1972	NO	YES	NO	No serotonin receptor antagonist
Breivik, 1971	NO	YES	NO	No serotonin receptor antagonist
Davies, 1971	NO	YES	NO	No serotonin receptor antagonist
Ellis, 1970	NO	YES	NO	No serotonin receptor antagonist

Lind, 1970	NO	YES	NO	No serotonin receptor antagonist
Gordon, 1969	NO	YES	NO	No serotonin receptor antagonist
Dobkin, 1968	NO	YES	NO	No serotonin receptor antagonist
Ratra, 1968	NO	YES	NO	No serotonin receptor antagonist
Handley, 1967	NO	YES	NO	No serotonin receptor antagonist
Snow, 1967	NO	YES	NO	No serotonin receptor antagonist

Abbreviations: CA, conference abstract; NMA, network meta-analysis.
0, study wasn't included in particular review. 1, study was included in particular review.

Appendix 4. Study characteristics

Author, year	Country of conduct	Study conduct period	Study duration (hours)	Setting	Interventions examined	Outcomes Reported
Randomized clinical trials (n=429)						
Browning, 2013	Australia	NR	NR	Multi-centre	Ondansetron, Placebo	Nausea, Vomiting
Aleyasin, 2012	Iran	February 2010 - January 2011	18	Hospital	Granisetron	Nausea, Vomiting
Blitz, 2012	NR	NR	72	NR	Palonosetron, Palonosetron + Dexamethasone	Nausea, Vomiting, PONV
Choi, 2012	South Korea	NR	48	NR	Ondansetron + Dexamethasone, Ramosetron + Dexamethasone	Nausea, Vomiting, PONV,
de Orange, 2012	Brazil	March 2011 - July 2011	24	Hospital	Dexamethasone, Ondansetron + Dexamethasone, Placebo	Vomiting
Eidi, 2012	Iran	NR	24	Hospital	Dexamethasone, Ondansetron, Placebo	Nausea, Vomiting, PONV
Gurha, 2012†	India	NR	24	Hospital	Granisetron, Ondansetron, Palonosetron	PONV
Koh, 2012	Korea	September 2009 - February 2010	48	NR	Placebo, Ramosetron	Nausea, Vomiting, PONV
Lahsaei, 2012	Iran	Fall 2010	24	Hospital	Ondansetron, Placebo	Nausea, Vomiting, PONV
Madenoglu, 2012	Turkey	NR	72	NR	Dexamethasone, Palonosetron + Dexamethasone	Nausea, Vomiting, PONV
Moon, 2012	South Korea	January 2010 - November 2010	24	Hospital	Ondansetron, Palonosetron	Nausea, Vomiting
Park JW, 2012	Korea	NR	24	NR	Palonosetron + Dexamethasone, Palonosetron	Nausea, Vomiting, PONV
Wang, 2012	China (Taiwan)	NR	24	NR	Dexamethasone, Haloperidol + Dexamethasone, Ondansetron + Dexamethasone	Nausea, PONV
Ahmed, 2011	Egypt	NR	24	NR	Mirtazapine, Ondansetron	Nausea, Vomiting, PONV
Apfel, 2011†	USA	NR	72	Multi-centre	Ondansetron, Palonosetron	PONV
Asl, 2011	Iran	2008	NR	Medical center	Ondansetron, Placebo	Nausea, Vomiting
Bajwa, 2011	India	January 2010 - May 2011	72	NR	Ondansetron, Palonosetron	Nausea, Vomiting
Basu, 2011	India	Setember 2007 - February 2009	24	Hospital	Granisetron, Ondansetron, Palonosetron	Nausea, Vomiting
Bhattarai, 2011	Nepal	NR	24	NR	Ondansetron, Ondansetron + Dexamethasone	PONV
Bicer, 2011	Turkey	March 2009 - December 2009	48	Medical center	Palonosetron, Palonosetron	Nausea, Vomiting, PONV

D'Souza, 2011	India	NR	24	NR	Dexamethasone, Ondansetron	Vomiting, PONV
Ekinci, 2011	Turkey	February 2007 - March 2008	48	NR	Droperidol, Metoclopramide, Ondansetron, Placebo, Tropisetron	PONV
El-deeb, 2011a	Egypt	NR	24	NR	Ondansetron + Dexamethasone, Placebo	Nausea, Vomiting, PONV
El-deeb, 2011b	Egypt	NR	24	NR	Granisetron + Dexamethasone, Placebo	Nausea, Vomiting
El-deeb, 2011c	Egypt	NR	24	Hospital	Ondansetron, Placebo	Nausea, Vomiting
Gan, 2011	USA, Canada	NR	119	NR	Ondansetron, Placebo, Rolapitant	Nausea, Vomiting, PONV
Islam, 2011	Bangladesh	July 2008 - June 2009	24	Hospital	Granisetron, Granisetron + Dexamethasone	PONV
Khan, 2011	Pakistan	2007 - 2008	12	Hospital	Metoclopramide, Ondansetron	Nausea, Vomiting
Lee, 2011	South Korea	NR	48	NR	Ondansetron, Ramosetron	Nausea, Vomiting, PONV
Metaxari, 2011	Greece	March 2006 - March 2009	24	NR	Granisetron, Ondansetron, Placebo, Tropisetron	Nausea, Vomiting
Park, 2011	Republic of Korea	June 2010 - September 2010	24	Hospital	Ondansetron, Palonosetron	Nausea, Vomiting, PONV
Ryu, 2011	South Korea	NR	48	NR	Ramosetron, Tropisetron	Nausea, Vomiting, PONV
Safae, 2011	Iran	2010	24	Hospital	Dexamethasone, Ondansetron	Nausea, Vomiting
Sahoo, 2011	India	September 2008 - December 2008	1	NR	Ondansetron, Placebo	Nausea
Singh, 2011	India	NR	24	NR	Granisetron, Placebo	Nausea, Vomiting
Song, 2011	South Korea	NR	48	NR	Ondansetron, Ondansetron + Dexamethasone	PONV
Srivastava, 2011	India	NR	24	NR	Granisetron, Metoclopramide, Ondansetron	Vomiting, PONV
Talesh, 2011	Iran	NR	24	NR	Metoclopramide, Ondansetron	Vomiting
Vale, 2011	Portugal	NR	24	Hospital	Ondansetron, Placebo	Nausea, Vomiting, PONV
Akhtar, 2010	India	January 2010 - March 2011	48	Hospital	Ondansetron, Ramosetron	Nausea, Vomiting, PONV
Alghanem, 2010	Jordan	November 2007 - March 2008	24	Hospital	Dexamethasone, Ondansetron, Placebo	Nausea, PONV
Bhattachajee, 2010	India	NR	48	Medical center	Granisetron, Palonosetron	Nausea, Vomiting, PONV
Bilgin, 2010	Turkey	NR	24	NR	Dexamethasone, Metoclopramide, Ondansetron, Placebo	Nausea, Vomiting, PONV
Choi, 2010	Korea	September 2008 - February 2009	48	NR	Ondansetron, Placebo, Ramosetron	Nausea, Vomiting, PONV

Dabbous, 2010	Lebanon	NR	24	NR	Granisetron + Dexamethasone, Ondansetron + Dexamethasone	Vomiting, PONV
Ebrahim Soltani, 2010 (CR: Ebrahim Soltani, 2011)	Iran	2007 - 2008	24	Hospital	Metoclopramide, Ondansetron, Placebo	Nausea, Vomiting
Hahm, 2010	South Korea	November 2008 - September 2009	48	NR	Ondansetron, Tropisetron	Nausea, Vomiting, PONV
Huh, 2010	Korea	NR	24	Hospital	Ondansetron, Placebo	Nausea, Vomiting, PONV
Jee, 2010	Korea	NR	36	NR	Metoclopramide + Dexamethasone, Ondansetron + Dexamethasone	Nausea, Vomiting, PONV
Jokela, 2010	Finland	December 2007 - January 2009	24	Hospital	Ondansetron, Placebo	Vomiting, PONV
Mishra, 2010	India	NR	24	NR	Metoclopramide, Ondansetron	PONV
Omar, 2010	Egypt	NR	NR	NR	Metoclopramide, Ondansetron	Nausea, Vomiting
Rauers, 2010	Germany, Switerland	NR	48	NR	Metoclopramide, Ondansetron, Placebo	Nausea, Vomiting, PONV
Ryu, 2010	South Korea	NR	48	NR	Ondansetron, Ramosetron	Nausea, Vomiting, PONV
Shakya, 2010	India	NR	2	NR	Ondansetron, Placebo	PONV
Singla, 2010	Belgium, Canada, Germany, Hungary, Spain, USA.	NR	119	Multi-centre	Casopitant, Ondansetron, Ondansetron + Casopitant	Nausea, Vomiting, PONV
Tan, 2010	Ireland	NR	24	NR	Granisetron, Ondansetron	PONV
Yun, 2010	Korea	NR	48	NR	Azasetron, Ondansetron	Nausea, Vomiting, PONV
Farouk, 2009	Egypt	NR	24	NR	Ondansetron, Placebo	PONV
Feng, 2009	Taiwan	NR	24	NR	Haloperidol, Ondansetron, Ondansetron + Haloperidol	Nausea, Vomiting, PONV
Gan, 2009	USA	NR	72	Hospital	Granisetron, Granisetron + Promethazine, Promethazine	Nausea, Vomiting, PONV
Gandhi, 2009	India	NR	24	NR	Dexamethasone, Ondansetron, Placebo	Nausea, Vomiting, PONV
Grover, 2009	India	NR	24	NR	Ondansetron, Placebo	Nausea, Vomiting, PONV
Jain, 2009	India	NR	24	NR	Granisetron, Ondansetron, Placebo	Nausea, Vomiting, PONV
Jellish, 2009	USA	NR	48	NR	Ondansetron, Ondansetron + Prochlorperazine	Nausea, Vomiting
Kim, 2009	Korea	NR	24	NR	Ondansetron, Placebo, Ramosetron	Nausea, Vomiting, PONV
Riad, 2009	Saudi Arabia	NR	24	NR	Granisetron + Dexamethasone, Ondansetron +	Nausea, Vomiting

					Dexamethasone, Placebo	
Rosow, 2009	USA	NR	NR	Hospital	Haloperidol, Ondansetron	Nausea, Vomiting, PONV
Aghadavoudi, 2008	Iran	NR	24	NR	Granisetron, Metoclopramide, Placebo	Nausea, Vomiting, PONV
Bano, 2008	Pakiston	March 2007 - September 2007	24	Hospital	Dexamethasone, Ondansetron + Dexamethasone	Nausea, Vomiting, PONV
Bhatia, 2008	India	NR	NR	NR	Granisetron, Metoclopramide, Ondansetron, Placebo	Nausea, Vomiting, PONV
Candiotti, 2008	USA, Romainia	NR	72	NR	Palonosetron, Placebo	Nausea, Vomiting, PONV
Choi, 2008	South Korea	September 2006 - June 2007	48	NR	Ondansetron, Ramosetron	Nausea, Vomiting
Contreras-Dominguez, 2008	Chile	December 2003 - February 2006	48	Hospital	Dexamethasone, Droperidol, Granisetron, Metoclopramide, Placebo, Tropisetron	PONV
Cruz, 2008	Puerto Rico	NR	24	Hospital	Ondansetron + Dexamethasone	Nausea, Vomiting, PONV
Davis, 2008	USA	NR	60	NR	Ondansetron + Dexamethasone	Vomiting
Erhan, 2008	Turkey	NR	24	NR	Dexamethasone, Granisetron, Ondansetron, Placebo	Nausea, Vomiting, PONV
Gautam, 2008	Nepal	NR	24	Hospital	Dexamethasone, Ondansetron, Ondansetron + Dexamethasone	Nausea, Vomiting, PONV
Gupta, 2008	India	NR	24	NR	Granisetron, Metoclopramide, Ondansetron	Vomiting
Kaki, 2008	Saudi Arabia	NR	24	Hospital	Metoclopramide, Ondansetron, Placebo	PONV
Kovac, 2008	Germany, Poland, Czech Republic	NR	72	Multi-centre	Palonosetron, Placebo	Nausea, Vomiting, PONV
Krobbuaban, 2008	Thailand	NR	24	Hospital	Metoclopramide, Ondansetron	PONV
Lee, 2008	Republic of Korea	NR	24	NR	Placebo, Ramosetron	Nausea, Vomiting, PONV
Ng, 2008	China	August 2003 - May 2004	3	NR	Ondansetron, Placebo	Vomiting, PONV
Olatosi, 2008	Nigeria	NR	24	NR	Ondansetron, Placebo, Promethazine	Nausea, Vomiting
Pan, 2008	USA	NR	119	Medical center	Ondansetron, Ondansetron + Dexamethasone	Nausea, Vomiting, PONV
Piper, 2008	Germany, Switzerland	1999 - 2004	24	NR	Dolsetron, Droperidol, Placebo	Nausea, Vomiting, PONV
Said-Ahmed, 2008	Egypt	NR	24	NR	Granisetron, Placebo	Vomiting
Sajedi, 2008	Iran	NR	NR	Hospital	Granisetron, Placebo	Nausea, Vomiting
Sakellaris, 2008	Greece	NR	24	NR	Ondansetron, Placebo	PONV
Schnaider, 2008	Brazil	NR	35	NR	Dexamethasone, Droperidol, Metoclopramide, Ondansetron,	Nausea, Vomiting

					Ondansetron + Dexamethasone, Ondansetron + Droperidol	
Aouad, 2007	Lebanon	NR	24	Medical center	Haloperidol, Ondansetron, Placebo	Nausea, Vomiting, PONV
Balki, 2007	Canada	May 2005 - November 2005	24	Hospital	Granisetron, Placebo	Nausea, Vomiting
Bestas, 2007	Turkey	NR	24	Medical center	Granisetron, Ondansetron, Placebo	PONV
Bhatagnar, 2007	India	NR	24	Hospital	Granisetron, Ondansetron, Placebo	Nausea, Vomiting, PONV
Bolton, 2007	Australia	October 1999 - March 2004	24	Hospital	Metoclopramide, Ondansetron	Vomiting
Boonmak, 2007	Thailand	NR	24	Hospital	Ondansetron	Nausea, Vomiting
Candiotti, 2007	USA	NR	24	NR	Granisetron, Ondansetron	Nausea, PONV
Diemunsch, 2007	North America (including USA), South America, Europe, Australia, Asia	May 2004 - April 2005	48	NR	Aprepitant, Ondansetron	Nausea, Vomiting, PONV
Gan, 2007	Multicentred	September 2003 - November 2004	48	NR	Aprepitant, Ondansetron	Nausea, Vomiting, PONV
Gombar, 2007	India	NR	24	NR	Granisetron, Granisetron + Dexamethasone, Placebo	Vomiting, PONV
Gulhas, 2007	Turkey	NR	24	NR	Ondansetron, Placebo	Nausea, Vomiting, PONV
Han, 2007	Korea	NR	48	NR	Ondansetron	Nausea, Vomiting
Harnett, 2007	USA	NR	24	Hospital	Ondansetron, Placebo, Scopolamine	Nausea, Vomiting
Kushwaha, 2007	India	NR	24	NR	Granisetron, Granisetron + Dexamethasone, Ondansetron, Ondansetron + Dexamethasone, Placebo	PONV
Lee, 2007	Republic of China (Taiwan)	NR	24	NR	Haloperidol, Ondansetron	Nausea, Vomiting, PONV
Moussa, 2007	Saudi Arabia	NR	24	NR	Granisetron, Granisetron + Dexamethasone, Granisetron + Droperidol, Placebo	Nausea, Vomiting, PONV
Oksuz, 2007	Turkey	NR	24	NR	Granisetron, Metoclopramide, Ondansetron	Nausea, Vomiting, PONV
Paech, 2007	Australia	March 2001 - December 2003	24	Hospital	Ondansetron + Dexamethasone	Nausea, Vomiting, PONV
Rusch, 2007	Germany	NR	24	Hospital	Dolsetron, Dolsetron + Dexamethasone, Haloperidol, Haloperidol + Dexamethasone	Vomiting, PONV
Sagir, 2007	Turkey	NR	0.25	NR	Granisetron, Placebo	PONV

Sandhu, 2007	Thailand	NR	24	NR	Metoclopramide, Ondansetron	Nausea, Vomiting
Sarkar, 2007	India	January 2007 - June 2007	24	Hospital	Metoclopramide, Ondansetron	Nausea, Vomiting
Sharma, 2007	India	NR	7	Hospital	Ondansetron, Placebo	Nausea, Vomiting
Siddik-Sayyid, 2007	Lebanon	NR	24	Medical center	Granisetron, Ondansetron, Placebo	Nausea, Vomiting, PONV
Sodhi, 2007	India	NR	NR	NR	Dexamethasone, Granisetron, Granisetron + Dexamethasone	Nausea, Vomiting, PONV
Sriraman, 2007	India	NR	NR	NR	Granisetron + Dexamethasone, Ondansetron + Dexamethasone	Nausea, Vomiting, PONV
Subramaniam, 2007	India	NR	24	Medical center	Ondansetron, Placebo	Nausea, Vomiting, PONV
Teran, 2007	USA	NR	24	Medical center	Granisetron, Placebo	PONV
White, 2007	USA	NR	72	Multi-centre	Ondansetron, Scopolamine	Nausea, PONV
Wig, 2007	India	NR	24	NR	Ondansetron, Placebo	Nausea, Vomiting
Akin, 2006	Turkey	NR	24	Hospital	Placebo, Tropisetron	Nausea, Vomiting, PONV
Birmingham, 2006	USA	NR	24	Medical center	Dolsetron, Ondansetron	Vomiting, PONV
Bridge, 2006	USA	NR	24	Hospital	Dolsetron, Granisetron, Ondansetron	PONV
Chan, 2006	USA, Hong Kong, New Zeland	September 2002 - June 2004	48	NR	Droperidol, Ondansetron, Ondansetron + Droperidol, Placebo	PONV
Eberhart, 2006	Germany	NR	24	NR	Dexamethasone, Placebo, Tropisetron, Tropisetron + Dexamethasone	Nausea, PONV
Gunter, 2006	USA	NR	24	Medical center	Metoclopramide + Dexamethasone, Ondansetron + Dexamethasone, Ondansetron + Metoclopramide	Vomiting
Gupta, 2006	NR	October 2004 - January 2005	NR	NR	Dexamethasone, Ondansetron	Nausea, Vomiting
Isik, 2006	Turkey	January 2004 - December 2004	24	Hospital	Dexamethasone, Ondansetron	Nausea, Vomiting, PONV
Janicki, 2006	USA	NR	24	NR	Dolsetron, Granisetron	Nausea, Vomiting, PONV
Jellish, 2006	USA	NR	48	NR	Ondansetron	Nausea, Vomiting
Johns, 2006	UK	NR	24	NR	Cyclizine, Granisetron, Granisetron + Cyclizine	Nausea, Vomiting, PONV
Khan, 2006	India	NR	24	NR	Granisetron, Granisetron + Dexamethasone, Placebo	PONV
Kolm, 2006	Brazil	NR	3	Hospital	Droperidol, Ondansetron, Placebo	PONV
Leksowski, 2006	Poland	NR	12	NR	Dexamethasone,	Nausea,

					Metoclopramide, Metoclopramide + Dexamethasone, Ondansetron, Ondansetron + Dexamethasone, Ondansetron + Metoclopramide, Placeb	Vomiting
Paranhos de Abreu, 2006	Spain	NR	NR	NR	Dexamethasone, Droperidol, Metoclopramide, Ondansetron, Placebo	Nausea, Vomiting
Peixoto, 2006	Brazil, USA	April 2001 - August 2003	24	NR	Droperidol, Ondansetron, Placebo	Nausea, Vomiting, PONV
Purhonen, 2006a	Finland	Sept 2003 - June 2004	24	NR	Ondansetron, Placebo	Nausea, Vomiting, PONV
Purhonen, 2000b	Finland	NR	24	Hospital	Ondansetron, Placebo	Nausea, Vomiting, PONV
Sarvela, 2006	Finland	January 2003 - November 2003	24	NR	Ondansetron, Placebo, Tropisetron	Nausea, PONV
Tosun, 2006	Turkey	NR	24	NR	Placebo, Tropisetron	Vomiting
Tseng, 2006	China (Taiwan)	July 2005 - Sept 2005	24	NR	Granisetron, Placebo	Nausea, Vomiting, PONV
Veneziani, 2006	Italy	NR	24	NR	Dolsetron, Dolsetron + Dexamethasone, Droperidol, Placebo	Nausea, Vomiting
White, 2006	USA	NR	48	Multi-centre	Granisetron, Ondansetron	Nausea, Vomiting, PONV
Cohen, 2005	USA	NR	24	NR	Dexamethasone, Ondansetron + Dexamethasone	Nausea, Vomiting
D'Angelo, 2005	USA	March 2002 - August 2002	24	Multi-centre	Granisetron, Placebo	Nausea, Vomiting, PONV
Demiraran, 2005	Turkey	January 2003-December 2004	60	NR	Ondansetron, Placebo	Nausea, Vomiting
Gan, 2005	USA	NR	24	NR	Granisetron + Dexamethasone, Ondansetron + Dexamethasone	Nausea, Vomiting, PONV
Hartsell, 2005	USA	October 2000 - December 2002	72	Hospital	Ondansetron, Placebo	Nausea, Vomiting
Iatrou, 2005	Greece	December 2003 - March 2004	24	NR	Dolsetron, Ondansetron, Placebo	PONV
Kabalak, 2005	Turkey	NR	24	NR	Ondansetron, Placebo	Vomiting
Khan, 2005	Saudi Arabia	January 2004 - June 2004	12	Hospital	Granisetron, Ondansetron, Placebo	Nausea, Vomiting, PONV
Kocamanoglu, 2005	Turkey	NR	24	NR	Droperidol, Granisetron, Granisetron + Dexamethasone, Granisetron + Droperidol, Placebo	Nausea, Vomiting, PONV
Kontrimaviciute, 2005	Lithuania	NR	24	Hospital	Ondansetron, Placebo	Nausea, PONV
Misra, 2005	India	NR	24	NR	Ondansetron, Placebo	Nausea,

						Vomiting
Pirat, 2005	Turkey	NR	24	NR	Ondansetron, Placebo	Nausea, Vomiting, PONV
Treschan, 2005	Germany	NR	24	NR	Ondansetron, Placebo	Nausea, Vomiting, PONV
White, 2005†	USA	NR	24	NR	Palonosetron, Placebo	Vomiting, PONV
Alkaissi, 2004	Sweden	May 2000 - January 2001	24	Multi-centre	Droperidol, Granisetron, Placebo	Nausea, Vomiting, PONV
Apfel, 2004 (CR: Jokela, 2009)	Germany, USA, Finland, UK, Turkey, Slovakia	February 2000 - July 2002	24	NR	Dexamethasone, Droperidol, Droperidol + Dexamethasone, Ondansetron, Ondansetron + Dexamethasone, Ondansetron + Droperidol	Nausea, Vomiting, PONV
Barbone, 2004	Italy	NR	24	Hospital	Dolsetron, Ondansetron	Nausea, Vomiting
Barbosa, 2004	Brazil	April 2001 - June 2002	24	Hospital	Ondansetron	PONV
Bhardwaj, 2004	India	NR	24	NR	Ondansetron, Ondansetron + Dexamethasone, Placebo	Vomiting, PONV
Binstock, 2004	USA	NR	24	NR	Ondansetron, Placebo	PONV
Browning, 2004	USA	NR	24	Medical center	Dolsetron, Ondansetron	Nausea, Vomiting
Celiker, 2004	Turkey	NR	24	NR	Ondansetron + Dexamethasone, Placebo	Vomiting
Dua, 2004	India	NR	24	NR	Granisetron, Ondansetron, Placebo	Nausea, Vomiting, PONV
Eberhart, 2004	Germany	NR	24	NR	Dolsetron, Dolsetron + Droperidol, Droperidol, Placebo	Nausea, Vomiting, PONV
Gan, 2004	USA	NR	24	NR	Ondansetron, Placebo	Nausea, Vomiting, PONV
Han, 2004	Korea	NR	48	NR	Ondansetron, Placebo	Nausea
Hanaoka, 2004	Japan	NR	24	Multi-centre	Granisetron, Placebo	Nausea, Vomiting
Meyer, 2004	USA	September 1998 - February 1999	NR	Hospital	Dolsetron, Ondansetron	PONV
Panda, 2004	India	NR	48	Hospital	Ondansetron, Ondansetron + Dexamethasone	PONV
Samakarndi, 2004	Saudi Arabia	NR	24	NR	Dexamethasone, Ondansetron, Ondansetron + Dexamethasone, Placebo	Nausea, Vomiting
Sinha, 2004	India	NR	48	Medical center	Ondansetron, Placebo	Nausea, Vomiting, PONV
Bharti, 2003	India	NR	24	Medical center	Droperidol, Ondansetron, Placebo	PONV
Biswas, 2003a	India	NR	24	NR	Dexamethasone, Ondansetron, Ondansetron + Dexamethasone, Placebo	Nausea, Vomiting, PONV

Biswas, 2003b	India	NR	24	Hospital	Granisetron, Granisetron + Dexamethasone	Nausea, Vomiting, PONV
Burmeister, 2003	Germany	NR	24	NR	Dolsetron, Placebo	Nausea, Vomiting, PONV
Charuluxananan, 2003	Thailand	NR	24	Hospital	Ondansetron, Placebo	Nausea, Vomiting, PONV
Karamanlioglu, 2003	Turkey	NR	24	NR	Dolsetron, Ondansetron, Placebo	Nausea, Vomiting, PONV
Loewen, 2003	Canada	May 2000 - December 2001	24	NR	Dolsetron, Droperidol	Nausea, Vomiting, PONV
Maddali, 2003	Oman	NR	24	NR	Metoclopramide + Dexamethasone, Ondansetron + Dexamethasone, Placebo	Nausea, Vomiting, PONV
Madenoglu, 2003	Turkey	NR	24	Hospital	Placebo, Tropisetron	Nausea, Vomiting
O'Brien, 2003	UK	NR	24	Hospital	Cyclizine, Ondansetron, Placebo	Vomiting
Olutoye, 2003	USA	NR	119	NR	Dolsetron, Ondansetron	Vomiting, PONV
Oztekin, 2003	Turkey	NR	48	NR	Droperidol, Tropisetron	Vomiting
Paech, 2003	Australia	NR	24	NR	Dolsetron, Ondansetron, Tropisetron	Nausea, Vomiting, PONV
Pan, 2003	India	NR	24	NR	Ondansetron, Placebo	Nausea, Vomiting
Samra, 2003[†]	USA	NR	24	NR	Dolsetron, Droperidol, Ondansetron, Placebo	PONV
Szarvas, 2003	Ireland	NR	24	NR	Dexamethasone, Ondansetron, Ondansetron + Dexamethasone	Nausea, Vomiting, PONV
Tang, 2003	USA	NR	24	Medical center	Dolsetron, Ondansetron, Placebo	Nausea, PONV
Thagaard, 2003	Norway	NR	72	Hospital	Ondansetron, Placebo	Nausea, Vomiting
Tzeng, 2003	Taiwan	NR	24	NR	Ondansetron, Placebo	Nausea, PONV
Usmani, 2003	India	NR	24	Hospital	Dexamethasone, Ondansetron, Ondansetron + Dexamethasone	Nausea, Vomiting, PONV
Wagner, 2003	USA	NR	24	Medical center	Dolsetron, Placebo	Vomiting, PONV
Wattwil, 2003	Sweden	NR	24	Hospital	Dexamethasone, Ondansetron	Nausea, Vomiting, PONV
Yuksek, 2003	Turkey	NR	24	NR	Dexamethasone, Ondansetron, Placebo	Nausea, Vomiting, PONV
Adducci, 2002	Italy	NR	24	Hospital	Metoclopramide, Metoclopramide + Dexamethasone, Ondansetron, Ondansetron + Dexamethasone, Placebo	Nausea, Vomiting, PONV
Agarwal, 2002	India	NR	24	NR	Ondansetron, Placebo	Nausea, Vomiting, PONV
Arcioni, 2002	Italy	NR	24	Hospital	Ondansetron, Placebo	Vomiting
Argiriadou, 2002	Greece	NR	12	Hospital	Ondansetron, Placebo,	Nausea,

					Tropisetron	Vomiting
Coloma, 2002	USA	NR	72	NR	Ondansetron, Placebo	Nausea, Vomiting, PONV
Coloma, 2002	USA	NR	24	NR	Dolsetron, Dolsetron + Dexamethasone	Nausea, Vomiting, PONV
Elhakim, 2002	Egypt	NR	24	NR	Ondansetron, Ondansetron + Dexamethasone, Placebo	Nausea, Vomiting, PONV
Fabling, 2002	Australia	NR	48	NR	Dexamethasone, Ondansetron + Dexamethasone	Nausea, Vomiting
Gan, 2002	USA	NR	24	NR	Ondansetron, Placebo	Nausea, Vomiting
Goksu, 2002	Turkey	NR	24	NR	Droperidol, Granisetron, Granisetron + Dexamethasone	Nausea, Vomiting, PONV
Grimsehl, 2002	UK	NR	48	NR	Cyclizine, Ondansetron	Nausea, Vomiting, PONV
Jokela, 2002	Finland	NR	24	Multi-centre	Metoclopramide, Ondansetron, Tropisetron	Nausea, PONV
Lee, 2002	South Korea	NR	24	NR	Granisetron, Placebo, Ramosetron	Nausea, Vomiting, PONV
Marcus, 2002	USA	NR	NR	NR	Ondansetron, Placebo	Nausea, Vomiting
Mullick, 2002	India	NR	24	NR	Metoclopramide, Ondansetron, Ondansetron + Metoclopramide	Nausea, Vomiting
Munro, 2002	UK	NR	24	NR	Droperidol, Ondansetron, Placebo	Nausea, Vomiting, PONV
Ozmen, 2002	Turkey	NR	24	NR	Granisetron, Granisetron + Droperidol	Vomiting, PONV
Paech, 2002	Australia	NR	24	NR	Dolsetron, Placebo	Nausea, Vomiting, PONV
Quaynor, 2002	Norway	NR	24	NR	Metoclopramide, Ondansetron	Vomiting, PONV
Sanchez-Ledesma, 2002	Spain	NR	48	NR	Droperidol + Dexamethasone, Ondansetron + Dexamethasone, Ondansetron + Droperidol	Nausea, PONV
Sennaraj, 2002	India, USA, UK	NR	24	NR	Ondansetron, Placebo	Nausea, PONV
So, 2002	Singapore	NR	24	NR	Ondansetron, Placebo	Nausea, Vomiting
Sukhani, 2002	USA	NR	48	NR	Dolsetron, Ondansetron, Placebo	Vomiting, PONV
Wang, 2002a	Taiwan	NR	24	NR	Dexamethasone, Placebo, Tropisetron	Nausea, Vomiting, PONV
Wang, 2002b	Taiwan	NR	24	Hospital	Dexamethasone, Placebo, Tropisetron	Nausea, PONV
Wang, 2002	Taiwan	NR	24	NR	Dexamethasone, Placebo, Tropisetron	Nausea, PONV
White, 2002	USA	NR	72	NR	Ondansetron, Placebo	Nausea, Vomiting
Yazigi, 2002	Lebanon	NR	24	Hospital	Ondansetron, Placebo	PONV
Bowhay, 2001	UK	NR	24	NR	Ondansetron, Placebo	Vomiting

Cherian, 2001	UK	NR	24	Hospital	Ondansetron, Placebo	PONV
Dabbous, 2001	Lebanon	NR	24	NR	Droperidol, Metoclopramide, Ondansetron	Nausea
De Witte, 2001	Belgium	NR	24	NR	Ondansetron, Placebo	PONV
Ganem, 2001	Barzil	NR	12	NR	Alizapride, Ondansetron	Nausea, Vomiting, PONV
Goll, 2001	Austria	NR	24	NR	Ondansetron, Placebo	Nausea, Vomiting, PONV
Kathirvel, 2001	India	NR	24	NR	Ondansetron, Placebo	Nausea, Vomiting, PONV
Millo, 2001	UK	NR	24	NR	Droperidol, Ondansetron	Nausea, Vomiting, PONV
Pan, 2001	USA	NR	24	NR	Metoclopramide, Ondansetron, Placebo	Nausea, Vomiting
Paventi, 2001	Italy	NR	24	NR	Ondansetron	Nausea, PONV
Shende, 2001	India	NR	24	NR	Droperidol, Ondansetron, Ondansetron + Droperidol, Placebo	Nausea, Vomiting, PONV
Somri, 2001	Israel	NR	24	Hospital	Ondansetron, Placebo	Vomiting
Splinter, 2001	Canada	NR	24	NR	Dexamethasone, Ondansetron + Dexamethasone	Vomiting
Subramaniam, 2001	India	NR	24	NR	Dexamethasone, Ondansetron, Placebo	Nausea, PONV
Thomas, 2001	UK	NR	24	Hospital	Dexamethasone, Ondansetron, Ondansetron + Dexamethasone	Nausea, Vomiting, PONV
White, 2001	UK	NR	24	Hospital	Ondansetron, Placebo	Nausea, Vomiting
Wilson, 2001	USA	NR	24	Hospital	Metoclopramide, Ondansetron, Placebo	Nausea, Vomiting, PONV
Yilmazlar, 2001	Turkey	NR	3	NR	Placebo, Tropisetron	Vomiting
Ahmed, 2000	UK	NR	24	NR	Ondansetron, Ondansetron + Cyclizine, Placebo	Nausea, Vomiting, PONV
Charuluxananan, 2000	Thailand	NR	4	Hospital	Ondansetron, Placebo	PONV
Fabling, 2000	USA	NR	48	NR	Droperidol, Ondansetron, Placebo	Nausea, Vomiting
Gesztesi, 2000	USA	NR	119	NR	721, CP-122, Ondansetron, Ondansetron + CP-122	Nausea, Vomiting
Holt, 2000	Australia	NR	24	Hospital	Tropisetron, Tropisetron + Dexamethasone	Vomiting
Jensen, 2000	australia	NR	24	Hospital	Placebo, Tropisetron	Vomiting
Jokela, 2000	Finland	NR	24	NR	Ondansetron, Placebo	Nausea, PONV
Kothari, 2000	USA	November 1998 - June 1999	24	NR	Dimenhydrinate, Ondansetron	Nausea, Vomiting
Kreisler, 2000	USA	NR	24	NR	Droperidol, Ondansetron, Promethazine	PONV
Ku, 2000	China	January 1995 - June 1997	24	NR	Ondansetron, Placebo	Nausea, Vomiting
Lee, 2000	Republic of China (Taiwan), Germany	NR	24	NR	Ondansetron, Placebo, Prochlorperazine	Vomiting

Liberman, 2000	USA	June 1996 - February 1998	336	NR	Ondansetron, Placebo	Nausea, Vomiting
Najeeb, 2000	India	NR	24	NR	Droperidol, Granisetron, Placebo	Nausea, Vomiting, PONV
Peixoto, 2000	Brazil, USA	NR	24	NR	Droperidol, Ondansetron, Ondansetron + Droperidol	Nausea, PONV
Philip, 2000	USA	NR	24	Multi-centre	Dolsetron, Placebo	Nausea, PONV
Reihner, 2000	Sweden	NR	24	Hospital	Droperidol, Ondansetron, Placebo	Nausea, Vomiting, PONV
Sadhasivam, 2000	USA	NR	24	NR	Ondansetron, Placebo	PONV
Scuderi, 2000	USA	NR	119	Medical center	Ondansetron, Placebo	Nausea, Vomiting, PONV
Sharma, 2000	Malaysia	NR	24	NR	Droperidol, Metoclopramide, Ondansetron, Placebo	PONV
Wu, 2000	Canada	NR	24	Hospital	Droperidol, Ondansetron, Ondansetron + Droperidol, Placebo	PONV
Zarate, 2000	USA	NR	24	NR	Dolsetron, Ondansetron	Nausea, Vomiting, PONV
Abouleish, 1999	USA	NR	2	NR	Ondansetron, Placebo	Nausea, Vomiting
Allen, 1999	Australia	NR	24	Hospital	Placebo, Tropisetron	Vomiting
Barst, 1999	USA	NR	24	Multi-centre	Ondansetron, Placebo	Vomiting
Broome, 1999	UK	NR	24	NR	Metoclopramide, Ondansetron	Nausea, Vomiting
Bugedo, 1999	Chile	NR	24	NR	Droperidol, Ondansetron, Ondansetron + Droperidol, Placebo	Nausea, Vomiting
Cholwill, 1999	UK	NR	24	NR	Cyclizine, Ondansetron, Placebo	Nausea, Vomiting, PONV
de Adana, 1999	Spain	NR	48	Hospital	Ondansetron, Placebo	PONV
Gurler, 1999	Turkey	NR	48	NR	Dexamethasone, Ondansetron + Dexamethasone	Vomiting
Helmy, 1999	Egypt	NR	24	NR	Droperidol, Metoclopramide, Ondansetron, Placebo	Nausea, Vomiting
Janknegt, 1999	The Netherlands	NR	119	NR	Droperidol, Granisetron, Granisetron + Dexamethasone	Nausea, Vomiting, PONV
Jokela, 1999	finland	October 1997 - May 1998	24	Hospital	Droperidol, Tropisetron	Nausea, Vomiting, PONV
Kathirvel, 1999	India	NR	24	NR	Metoclopramide, Ondansetron, Placebo	Nausea, Vomiting, PONV
Khalil, 1999	USA	NR	24	NR	Ondansetron, Placebo, Promethazine	Nausea, Vomiting, PONV
Koivuranta, 1999	Finland	NR	24	Hospital	Ondansetron, Tropisetron	Nausea, Vomiting
Kovac, 1999	USA	NR	24	Multi-centre	Ondansetron, Placebo	Nausea, PONV
Kyokong, 1999	Thiland	NR	24	Hospital	Ondansetron, Placebo	Nausea, Vomiting

McCall, 1999	USA	NR	8	NR	Dimenhydrinate, Ondansetron, Placebo	Vomiting, PONV
Munro, 1999	USA	NR	24	NR	Granisetron, Placebo	Vomiting
Rajeeva, 1999	India	NR	24	NR	Ondansetron, Ondansetron + Dexamethasone	Nausea, Vomiting
Sadhasivam, 1999	India	NR	24	NR	Ondansetron, Placebo	Nausea, PONV
Sandhu, 1999	Canada	NR	24	NR	Dimenhydrinate, Ondansetron, Placebo	Nausea, Vomiting
Scuderi, 1999a	USA	NR	NR	Hospital	Ondansetron, Placebo	Nausea, Vomiting, PONV
Scuderi, 1999b	USA	NR	NR	Hospital	Ondansetron, Placebo	Nausea, Vomiting
Sinha, 1999	India	NR	48	Hospital	Ondansetron, Placebo	Nausea, Vomiting, PONV
Swiatkowski, 1999	Poland	NR	24	NR	Droperidol, Ondansetron	Nausea, Vomiting
Tsui, 1999	China	NR	48	Hospital	Ondansetron, Placebo, Tropisetron	Nausea, Vomiting
Turhanoglu, 1999	Turkey	NR	24	NR	Dimenhydrinate, Ondansetron, Ondansetron + Dimenhydrinate, Placebo	Nausea, Vomiting
Wagley, 1999	USA	NR	29	Medical center	Ondansetron, Placebo	Nausea, Vomiting
Woodward, 1999	UK	NR	24	Hospital	Metoclopramide, Ondansetron	Nausea, Vomiting
Alon, 1998	Switzerland	NR	24	NR	Placebo, Tropisetron	Nausea
Ang, 1998	Australia	NR	24	Hospital	Placebo, Tropisetron	Vomiting
Bacic, 1998	Croatia	NR	24	NR	Ondansetron, Placebo	PONV
Chan, 1998	Hong Kong	NR	48	Hospital	Placebo, Tropisetron	Nausea, Vomiting
Chen, 1998a	USA	NR	48	NR	Ondansetron, Prochlorperazine	Nausea, Vomiting
Chen, 1998b	China	NR	24	Hospital	Ondansetron, Placebo	Vomiting
Dabbous, 1998	Lebanon	NR	24	NR	Metoclopramide, Ondansetron	Vomiting
Daftary, 1998	India	NR	8	NR	Metoclopramide, Ondansetron, Placebo	Nausea, Vomiting
Dershwitz, 1998	USA	NR	24	NR	Ondansetron, Placebo	Nausea
Diemunsch, 1998	France	NR	24	NR	Dolsetron, Placebo	Nausea, PONV
Doe, 1998	USA	NR	24	NR	Droperidol, Ondansetron	Nausea, Vomiting
Dresner, 1998	England	NR	24	NR	Droperidol, Ondansetron	Nausea, Vomiting
Goodarzi, 1998	USA	NR	48	NR	Droperidol, Ondansetron, Placebo	Vomiting, PONV
Hamid, 1998	Canada	NR	24	NR	Dimenhydrinate, Ondansetron, Placebo	Vomiting
Jellish, 1998	USA	NR	24	Medical center	Droperidol, Ondansetron, Placebo	Nausea, Vomiting, PONV
Mitra, 1998	India	NR	24	NR	Dexamethasone, Ondansetron, Ondansetron + Dexamethasone, Placebo	Nausea, Vomiting, PONV
Morris, 1998	Australia, Canada, Denmark, France, Germany, Iceland, Israel, Netherlands, Norway, South Africa, Sweden,	NR	24	Multi-centre	Metoclopramide, Ondansetron, Placebo	Nausea, Vomiting

	UK					
Scholz, 1998	Germany	NR	24	NR	Ondansetron, Placebo, Tropisetron	Nausea, Vomiting
Splinter, 1998a	Canada	NR	24	NR	Ondansetron, Ondansetron + Dexamethasone	Vomiting
Splinter, 1998b	Canada	NR	24	Hospital	Ondansetron, Perphenazine	Vomiting
Tang, 1998a	USA	NR	24	Medical center	Palonosetron, Placebo	Vomiting, PONV
Tang, 1998b	USA	NR	24	Medical center	Ondansetron, Placebo	Nausea, Vomiting, PONV
Tramer, 1998	Switzerland	NR	48	Hospital	Ondansetron, Placebo	Vomiting
Akcabay, 1997	Turkey	NR	24	NR	Ondansetron, Placebo	Nausea, Vomiting
Alexander, 1997	UK	NR	24	NR	Metoclopramide, Ondansetron, Placebo	Nausea, PONV
Ascaso, 1997	Spain	NR	24	Hospital	Metoclopramide, Ondansetron, Placebo	Nausea, Vomiting
Bach-Styles, 1997	USA	NR	24	Medical center	Metoclopramide, Ondansetron, Placebo	Vomiting
Carnahan, 1997	USA	NR	24	NR	Granisetron, Placebo	Vomiting
Diemunsch, 1997a	France	NR	24	NR	Metoclopramide, Ondansetron	Nausea
Diemunsch, 1997b	France	NR	24	NR	Dolsetron, Placebo	Nausea, PONV
Diemunsch, 1997c	Europe	NR	24	NR	Dolsetron, Placebo	Nausea, PONV
Graczyk, 1997	USA	NR	24	NR	Dolsetron, Placebo	PONV,
Klockgether-Radke, 1997	Germany	NR	24	NR	Droperidol, Ondansetron, Placebo	Vomiting, PONV
Koivuranta, 1997	Finland	NR	24	Hospital	Droperidol, Ondansetron, Placebo	Nausea, Vomiting
Korttila, 1997	finland	NR	24	Multi-centre	Dolsetron, Ondansetron, Placebo	Nausea, PONV
Kovac, 1997	USA	NR	24	NR	Dolsetron, Placebo	Nausea, PONV
Lawhorn, 1997	USA	NR	24	Hospital	Ondansetron, Ondansetron + Metoclopramide, Placebo	Vomiting
Maestre, 1997	Spain	NR	24	NR	Droperidol, Metoclopramide, Ondansetron, Placebo	Nausea, Vomiting, PONV
McKenzie, 1997	USA	NR	24	Hospital	Ondansetron, Ondansetron + Dexamethasone	Nausea, Vomiting, PONV
Mikawa, 1997	Japan	NR	24	NR	Granisetron, Placebo	Nausea, Vomiting
Moens, 1997	Belgium	NR	24	Multi-centre	Ondansetron, Placebo	Nausea, Vomiting, PONV
Monagle, 1997	Australia	NR	24	NR	Metoclopramide, Ondansetron	Nausea, Vomiting
Morton, 1997	Denmark, Sweden, UK, France, Belgium, Germany, Norway.	NR	24	Multi-centre	Ondansetron, Placebo	Nausea, Vomiting
Patel, 1997	USA	NR	24	Multi-centre	Ondansetron, Placebo	PONV
Pitkanen, 1997	Finland	NR	24	NR	Metoclopramide, Ondansetron, Placebo	Nausea, Vomiting
Polati, 1997	Italy	NR	NR	NR	Metoclopramide, Ondansetron, Placebo	PONV
Purhonen, 1997	Finland	NR	48	Hospital	Droperidol, Placebo, Tropisetron	Nausea, Vomiting, PONV

Rung, 1997	USA	NR	24	Multi-centre	Ondansetron, Placebo	Nausea, PONV
Scuderi, 1997	USA	NR	24	NR	Droperidol, Metoclopramide, Ondansetron, Placebo	Vomiting
Shende, 1997	India	NR	24	NR	Metoclopramide, Ondansetron, Placebo	Vomiting
Sniadach, 1997	USA	NR	24	NR	Droperidol, Ondansetron	Nausea, Vomiting
Splinter, 1997	Canada	NR	NR	NR	Ondansetron	Vomiting
Stienstra, 1997	Netherlands	NR	3	NR	Alizapride, Droperidol, Ondansetron	Nausea, Vomiting
Sun, 1997	USA	NR	24	NR	Ondansetron, Placebo	Nausea, Vomiting
Taylor, 1997	UK	NR	144	Multi-centre	Granisetron, Placebo	Nausea, Vomiting,
Ulusoy, 1997	Turkey	nr	24	Hospital	Ondansetron, Placebo	Nausea, Vomiting
Warriner, 1997	Canada	NR	24	Multi-centre	Dolsetron, Placebo	PONV
Ali-melkkila, 1996	Finland	NR	24	Hospital	Metoclopramide, Placebo, Tropisetron	Nausea, Vomiting
Alon, 1996	Switzerland	NR	24	Hospital	Placebo, Tropisetron	Nausea, Vomiting, PONV
Capouet, 1996	Belgium	NR	24	Multi-centre	Placebo, Tropisetron	Nausea, Vomiting
Chen, 1996	Hong Kong	NR	24	NR	Metoclopramide, Ondansetron	Nausea, Vomiting, PONV
Cieslak, 1996	US	NR	24	NR	Granisetron, Placebo	Vomiting
Davies, 1996	UK	NR	119	NR	Ondansetron, Placebo	Nausea, Vomiting, PONV
Ercelen, 1996	Turkey	NR	24	NR	Droperidol, Metoclopramide, Ondansetron, Placebo	PONV
Eriksson, 1996	Finland	NR	24	NR	Ondansetron, Placebo	Nausea, Vomiting, PONV
Furst, 1996	USA	NR	24	NR	Ondansetron, Placebo	Vomiting
Gaynes, 1996†	USA	NR	8	NR	Ondansetron, Placebo	Nausea, Vomiting
Honkavaara, 1996	Finland	NR	24	Hospital	Ondansetron, Placebo	Nausea, Vomiting, PONV
Kaul, 1996	India	NR	24	NR	Metoclopramide, Ondansetron, Placebo	Nausea, PONV
Khalil, 1996	USA	NR	24	Multi-centre	Ondansetron, Placebo	PONV
Kimya, 1996	Turkey	NR	24	Medical center	Ondansetron, Placebo	Nausea, Vomiting
Koivuranta, 1996	Finland	NR	24	NR	Ondansetron, Placebo	Nausea, Vomiting
Kovac, 1996 (CR: Pearman, 1994c)	USA	NR	24	NR	Ondansetron, Placebo	Nausea, Vomiting, PONV
Kumar, 1996	India	NR	24	NR	Metoclopramide, Ondansetron	Nausea, Vomiting
Lawhorn, 1996	USA	NR	24	NR	Droperidol, Metoclopramide, Ondansetron, Ondansetron + Metoclopramide	Vomiting
Lekprasert, 1996	Thailand	NR	24	NR	Ondansetron, Placebo	Nausea, Vomiting, PONV
Lopez-Orlaondo, 1996	Spain	NR	48	NR	Dexamethasone, Ondansetron,	Nausea, Vomiting,

					Ondansetron + Dexamethasone, Placebo	PONV
Naguib, 1996	Saudi Arabia	NR	24	NR	Granisetron, Metoclopramide, Ondansetron, Placebo, Tropisetron	PONV
Pan, 1996	USA	NR	NR	NR	Droperidol, Ondansetron, Placebo	Nausea, Vomiting
Principi, 1996	Italy	NR	24	NR	Ondansetron, Placebo	Nausea, Vomiting, PONV
Pueyo, 1996	Spain	NR	NR	NR	Droperidol, Ondansetron, Ondansetron + Droperidol, Placebo	Nausea, Vomiting, PONV
Pugh, 1996	UK	NR	48	NR	Metoclopramide, Ondansetron	Nausea, Vomiting, PONV
Rodrigo, 1996	Hong Kong	NR	24	Hospital	Ondansetron, Placebo	Nausea, Vomiting
Rose, 1996a	USA	NR	24	NR	Ondansetron, Placebo	Vomiting
Rose, 1996b	USA	NR	NR	NR	Metoclopramide, Ondansetron, Placebo	Vomiting
Stene, 1996	USA	NR	5	Hospital	Metoclopramide, Ondansetron, Placebo	Vomiting
Tang, 1996	USA	NR	24	Medical center	Droperidol, Ondansetron, Placebo	Nausea, Vomiting
Usha, 1996	India	NR	24	Medical center	Ondansetron, Placebo	Nausea, Vomiting
Van Den Berg, 1996a	Saudi Arabia	January 1994 - August 1995	24	Hospital	Ondansetron, Placebo, Prochlorperazine	Nausea, Vomiting, PONV
Van Den Berg, 1996b	Saudi arabia	NR	24	Hospital	Ondansetron, Placebo, Prochlorperazine	Nausea, Vomiting, PONV
Van Den Berg, 1996c	Saudi Arabia	January 1994 - July 1995	24	Hospital	Ondansetron, Placebo, Prochlorperazine	Nausea, Vomiting, PONV
Watts, 1996	New Zealand	NR	24	NR	Cyclizine, Metoclopramide, Ondansetron	PONV
Wilson, 1996	Denmark, Finland, France, Italy, Netherlands, South Africa, Switzerland, UK	NR	24	Multi-centre	Granisetron, Placebo	Nausea, Vomiting, PONV
Wrench, 1996	UK	NR	18	Medical center	Droperidol, Ondansetron, Ondansetron + Droperidol	Nausea, Vomiting
Yilmazlar, 1996	Turkey	NR	3	NR	Metoclopramide, Placebo, Tropisetron	Vomiting
Alexander, 1995	UK	NR	24	NR	Droperidol, Ondansetron, Placebo	Nausea, Vomiting, PONV
Campbell, 1995	USA	NR	24	Medical center	Ondansetron, Placebo	Nausea, Vomiting, PONV
Davis, 1995a	Saudi Arabia	NR	24	NR	Droperidol, Ondansetron	Vomiting
Davis, 1995b	USA	NR	24	Hospital	Droperidol, Ondansetron, Placebo	Vomiting
Desilva, 1995	USA	NR	4	NR	Droperidol, Metoclopramide, Ondansetron, Perphenazine, Placebo	PONV
Elhakim, 1995	Egypt	NR	24	NR	Ondansetron, Placebo	Nausea, Vomiting,

						PONV
Grond, 1995	Germany	NR	24	NR	Droperidol, Ondansetron	Nausea, Vomiting, PONV
Litman, 1995	USA	NR	24	Hospital	Droperidol, Ondansetron	Vomiting
Mikawa, 1995	Japan	NR	24	NR	Granisetron, Placebo	Nausea, Vomiting
Paech, 1995	Australia	NR	24	Hospital	Droperidol, Ondansetron, Placebo	Nausea, Vomiting
Paxton, 1995a	Ireland	NR	24	NR	Droperidol, Ondansetron, Placebo	Vomiting
Paxton, 1995b	Ireland	NR	48	NR	Droperidol, Metoclopramide, Ondansetron, Placebo	Nausea, Vomiting
Splinter, 1995a	Canada	NR	48	NR	Ondansetron, Placebo	Vomiting
Splinter, 1995b	Canada	NR	24	NR	Droperidol, Ondansetron	Vomiting
Watcha, 1995	USA	NR	24	Medical center	Ondansetron, Placebo	Vomiting
Furst, 1994	USA	NR	24	NR	Droperidol, Metoclopramide, Ondansetron, Placebo	Vomiting
Gan, 1994	UK	NR	24	NR	Droperidol, Ondansetron, Placebo	Nausea, Vomiting, PONV
Kaufmann, 1994	Switzerland, USA	NR	35	NR	Droperidol, Metoclopramide, Placebo, Tropisetron	PONV
Khalil, 1994 (CR: Pearman, 1994b)	USA	NR	24	Multi-centre	Ondansetron, Placebo	PONV
Litman, 1994	USA	NR	48	NR	Ondansetron, Placebo	Vomiting
Malins, 1994	UK	NR	48	NR	Metoclopramide, Ondansetron, Placebo	Nausea, Vomiting, PONV
McKenzie, 1994	USA	NR	24	NR	Ondansetron, Ondansetron + Dexamethasone	Vomiting, PONV
Rodrigo, 1994	Hong Kong	NR	24	NR	Ondansetron, Placebo	Nausea, Vomiting
Rose, 1994	USA	NR	24	NR	Metoclopramide, Ondansetron, Placebo	Vomiting
Suen, 1994	Hong Kong	NR	24	Hospital	Ondansetron, Placebo	Nausea, Vomiting, PONV
Ummenhofer, 1994	Switzerland	NR	24	Hospital	Ondansetron, Placebo	PONV
Volpe, 1994	Italy	NR	24	NR	Ondansetron, Placebo	Nausea, Vomiting, PONV
Dupeyron, 1993	Belgium, Denmark, France	NR	24	NR	Ondansetron, Placebo	Nausea, Vomiting, PONV
Helmers, 1993	Netherlands, Ireland, Sweden, UK, South Africa, France,	NR	24	NR	Ondansetron, Placebo	Nausea, Vomiting
McKenzie, 1993b (CR: Dershwitz, 1992)	USA	NR	24	Multi-centre	Ondansetron, Placebo	Nausea, PONV
Raphael, 1993	UK	NR	24	NR	Metoclopramide, Ondansetron	Nausea, Vomiting, PONV
Sung, 1993	USA	NR	24	Multi-centre	Ondansetron, Placebo	Nausea, Vomiting, PONV
Zomers, 1993	Netherlands	NR	24	NR	Placebo, Tropisetron	Nausea, Vomiting
Alon, 1992	Switzerland	NR	3	Hospital	Droperidol,	Nausea,

					Metoclopramide, Ondansetron	Vomiting, PONV
Du Pen, 1992 (CR: Scuderi, 1993)	USA	NR	24	Multi-centre	Ondansetron, Placebo	Nausea, PONV
Kenny, 1992	UK, Netherlands, Germany, Norway	NR	168	Multi-centre	Ondansetron, Placebo	Nausea
Kovac, 1992 (CRs: McKenzie, 1993a; Pearman, 1994a)	USA	NR	24	Multi-centre	Ondansetron, Placebo	Nausea, Vomiting
Bodner, 1991	USA	NR	24	Hospital	Ondansetron, Placebo	Nausea, Vomiting
Larijani, 1991	USA	NR	24	NR	Ondansetron, Placebo	Nausea, PONV
Leeser, 1991	Netherlands	NR	168	Hospital	Ondansetron, Placebo	Nausea, Vomiting
Non-randomized clinical trials (n=5)						
Stamer, 2011	Germany	NR	48	NR	Ondansetron	PONV
Rabbani, 2010	Pakistan	January 2009 - June 2009	119.28	Hospital	Dexamethasone, Ondansetron	PONV
Kim, 2007	Korea	NR	24	NR	Ondansetron + Dexamethasone, Placebo	Nausea, Vomiting, PONV
Biedler, 2004	Germany	NR	24	Medical center	Ondansetron, Placebo	Nausea, Vomiting, PONV
Manani, 1996	Italy	NR	24	NR	Droperidol, Ondansetron, Placebo	PONV
Controlled before-after studies (n=1)						
Wagner, 2007	USA	NR	24	Medical center	Ondansetron, Placebo	Vomiting, PONV
Cohort studies (n=9)						
Shakeel, 2012	UK	2000 - 2009	24	Hospital	Dexamethasone, Ondansetron, Ondansetron + Dexamethasone, Placebo	Vomiting
Chen, 2011	Taiwan	2000 - 2009	24	Hospital	Ondansetron, Placebo	Nausea, Vomiting, PONV
Eryilmaz, 2008	Turkey	NR	24	NR	Metoclopramide, Ondansetron	PONV
Habib, 2007	USA	April 2001 - June 2005	NR	Medical center	Ondansetron, Promethazine	PONV
Flynn, 2006	USA	January 1995 - June 2001	56952	NR	Droperidol, Ondansetron, Placebo	Vomiting
Piwko, 2005	Canada	1996 - 2001	17808	Hospital	Ondansetron, Dimenhydrinate	Vomiting
Wang, 2005	USA	March 2003 - February 2004	NR	Hospital	Dolsetron, Granisetron, Ondansetron	PONV
Walker, 2001	USA	October 1998 - March 1999	24	Medical center	Dolsetron, Ondansetron	Nausea, Vomiting, PONV
O'Brien, 2000	Ireland	NR	NR	NR	Ondansetron, Promethazine	Vomiting

Note: †Unpublished data (Gurha, 2012; Apfel, 2011; White, 2005; Samra, 2003; Gaynes, 1996).
Abbreviations: CR, companion report; NR, not reported; PONV, Post-operative nausea and vomiting; UK, United Kingdom; USA United States of America.

Appendix 5. Frequencies for specific drugs and drug combinations

Intervention/ Comparator:	% of arms (n=1260)	% of studies (n=444)
Ondansetron	31.51	75.68
Placebo/no treatment	23.41	65.99
Metoclopramide	5.63	15.54
Droperidol	5.40	15.09
Granisetron	5.71	12.84
Ondansetron + Dexamethasone	4.44	10.36
Dexamethasone	3.25	9.01
Tropisetron	3.49	7.88
Dolasetron	5.08	7.43
Granisetron + Dexamethasone	1.19	3.38
Palonosetron	2.14	3.15
Ondansetron + Droperidol	0.79	2.25
Ramosetron	1.03	2.25
Dimenhydrinate	0.48	1.35
Promethazine	0.48	1.35
Cyclizine	0.40	1.13
Haloperidol	0.40	1.13
Metoclopramide + Dexamethasone	0.40	1.13
Ondansetron + Metoclopramide	0.56	1.13
Prochlorperazine	0.63	1.13
Dolasetron + Dexamethasone	0.24	0.68
Granisetron + Droperidol	0.24	0.68
Palonosetron + Dexamethasone	0.32	0.68
Alizapride	0.16	0.45
Aprepitant	0.32	0.45
Droperidol + Dexamethasone	0.16	0.45
Haloperidol + Dexamethasone	0.16	0.45
Perphenazine	0.16	0.45
Scopolamine	0.16	0.45
Tropisetron + Dexamethasone	0.16	0.45
Azasetron	0.08	0.23
Casopitant	0.08	0.23
CP-122, 721	0.08	0.23
Dolasetron + Droperidol	0.08	0.23
Granisetron + Cyclizine	0.08	0.23
Granisetron + Promethazine	0.08	0.23
Mirtazapine	0.08	0.23
Ondansetron + Casopitant	0.24	0.23
Ondansetron + CP-122, 721	0.08	0.23
Ondansetron + Cyclizine	0.08	0.23
Ondansetron + Dimenhydrinate	0.08	0.23
Ondansetron + Haloperidol	0.08	0.23
Ondansetron + Prochlorperazine	0.08	0.23
Ramosetron + Dexamethasone	0.08	0.23
Rolapitant	0.32	0.23

Appendix 6. Patient characteristics

Author, year	Sample size	Age Category	% female	ASA status	Surgery type	H/o motion sickness	H/o PONV	Comorbidities
Randomized clinical trials (n=429)								
Browning, 2013	118	Adults	NR	I or II	Obstetrics & Gynaecological	NR	NR	Mental health
Aleyasin, 2012	120	Adults	100	NR	Breast	NR	NR	NR
Blitz, 2012	118	Adults	84	I or II	Miscellaneous	NR	NR	NR
Choi, 2012	120	Adults	100	I or II	Orthopaedic	YES	YES	NR
de Orange, 2012	129	Children	24	I or II	Miscellaneous	NR	YES	NR
Eidi, 2012	219	Adults	72	I or II	Ear, nose and larynx	NR	NR	NR
Gurha, 2012†	30	Adults & Elderly	NR	NR	Miscellaneous	NR	NR	NR
Koh, 2012	119	Adults & Elderly	89	NR	Orthopaedic	NR	NR	Osteoarthritis
Lahsaei, 2012	300	Adults	NR	I or II	Orthopaedic	NR	NR	NR
Madenoglu, 2012	90	Adults & Elderly	48	I or II or III	Neurological	NR	NR	NR
Moon, 2012	100	Adults	100	I or II	Endocrine	YES	YES	NR
Park JW, 2012	84	Adults	100	I or II	Miscellaneous	YES	YES	NR
Wang, 2012	130	Adults	100	I or II	Miscellaneous	YES	YES	NR
Ahmed, 2011	80	Adults	100	I or II	Breast	NR	NR	NR
Apfel, 2011†	48	NR	NR	NR	Miscellaneous	NR	YES	NR
Asl, 2011	90	Adults	100	I or II	Obstetrics & Gynaecological	NR	NR	NR
Bajwa, 2011	60	Adults	100	I or II	Obstetrics & Gynaecological	NR	NR	NR
Basu, 2011	75	Adults	67	I or II	Otolaryngological	YES	YES	NR
Bhattarai, 2011	150	Children	49	I	Eye	NR	NR	NR
Bicer, 2011	100	Adults	83	I or II	Gastrointestinal	NR	NR	NR
D'Souza, 2011	92	Adults	100	I or II	Obstetrics & Gynaecological	NR	NR	NR
Ekinci, 2011	100	Adults	100	I or II or III	Obstetrics & Gynaecological	NR	NR	NR
El-deeb, 2011a	80	Adults	NR	I or II	Endocrine	NR	NR	NR
El-deeb, 2011b	160	Adults	100	I or II	Obstetrics & Gynaecological	NR	NR	NR
El-deeb, 2011c	300	Adults	100	I or II	Obstetrics & Gynaecological	NR	NR	NR
Gan, 2011	619	Adults	100	I or II or III	Miscellaneous	YES	YES	NR
Islam, 2011	100	Adults & Children	NR	I or II	Otolaryngological	NR	NR	NR
Khan, 2011	60	Adults	100	I or II	Gastrointestinal	NR	NR	NR
Lee, 2011	120	Adults	100	I or II	Obstetrics & Gynaecological	YES	YES	NR
Metaxari, 2011	203	Adults	100	I or II	Endocrine	YES	YES	NR
Park, 2011	90	Adults	100	I or II	Obstetrics & Gynaecological	YES	YES	NR
Ryu, 2011	124	Adults	54	I or II	Gastrointestinal	NR	NR	Cardiovascular, liver disease
Safae, 2011	64	Adults & Elderly	100	NR	Gastrointestinal	YES	NR	Cardiovascular, diabetes, asthma, disorders of the ear
Sahoo, 2011	52	Adults	100	I	Obstetrics & Gynaecological	NR	NR	NR
Singh, 2011	50	Adults	42	I or II	Miscellaneous	NR	NR	NR
Song, 2011	123	Adults	39	I or II	Breast	YES	YES	Cardiovascular, cancer, diabetes
Srivastava, 2011	75	Adults	86	I or II	Gastrointestinal	NR	NR	NR
Talesh, 2011	100	Adults	NR	NR	General dentistry, oral and maxillofacial surgery and	NR	NR	NR

					orthodontics			
Vale, 2011	40	Adults & Elderly	55	I or II or III	Miscellaneous	NR	NR	NR
Akhtar, 2010	130	Adults	100	I or II	Gastrointestinal	NR	NR	NR
Alghanem, 2010	180	Adults & Elderly	81	I or II	Gastrointestinal	NR	YES	Cardiovascular, diabetes
Bhattachajee, 2010	60	Adults	100	I or II	Gastrointestinal	NR	NR	NR
Bilgin, 2010	160	Adults	100	I or II	Obstetrics & Gynaecological	NR	NR	NR
Choi, 2010	279	Adults & Elderly	39	NR	Cardiovascular	YES	YES	NR
Dabbous, 2010	84	Adults	50	I or II	Miscellaneous	YES	YES	NR
Ebrahim Soltani, 2010 (CR: Ebrahim Soltani, 2011)	150	Adults & Children	48	I or II	Eye	NR	NR	NR
Hahm, 2010	84	Adults & Elderly	100	I or II or III	Orthopaedic	NR	NR	NR
Huh, 2010	60	Adults	100	I or II	Obstetrics & Gynaecological	NR	NR	NR
Jee, 2010	100	Adults	100	I or II	Obstetrics & Gynaecological	YES	YES	NR
Jokela, 2010	80	Adults	100	I or II or III	Obstetrics & Gynaecological	YES	YES	NR
Mishra, 2010	40	Adults & Children	46	I or II	Miscellaneous	NR	NR	NR
Omar, 2010	40	Adults	52	I or II	Miscellaneous	NR	NR	NR
Rauers, 2010	179	Adults & Elderly	35	I or II or III	Miscellaneous	YES	YES	Cancer
Ryu, 2010	120	Adults	50	I or II	Gastrointestinal	NR	NR	Cardiovascular, liver disease
Shakya, 2010	80	Adults	63	I or II	Miscellaneous	NR	NR	NR
Singla, 2010	702	Adults	100	NR	Miscellaneous	YES	YES	NR
Tan, 2010	80	Adults	100	I or II	Obstetrics & Gynaecological	NR	NR	NR
Yun, 2010	98	Adults	100	I or II	Obstetrics & Gynaecological	NR	NR	NR
Farouk, 2009	30	Adults	36	I or II	Orthopaedic	NR	NR	NR
Feng, 2009	192	Adults	74	I or II	Gastrointestinal	YES	YES	NR
Gan, 2009	138	Adults	100	I or II or III	Obstetrics & Gynaecological	YES	YES	NR
Gandhi, 2009	150	Adults	97	I or II	Multiple types included	NR	NR	NR
Grover, 2009	103	Adults	74	I or II	Gastrointestinal	NR	NR	NR
Jain, 2009	87	Adults	29.9	I or II	Neurological	NR	NR	NR
Jellish, 2009	100	Adults	84	I or II or III	Miscellaneous	YES	YES	NR
Kim, 2009	162	Adults & Elderly	100	NR	Obstetrics & Gynaecological	YES	YES	NR
Riad, 2009	100	Children	48	I or II	Eye	NR	NR	NR
Rosow, 2009	244	Adults & Elderly	66	I or II or III	Miscellaneous	NR	NR	NR
Aghadavoudi, 2008	120	Adults	47	II or III	Cardiac	NR	YES	NR
Bano, 2008	97	Adults	87	I or II	Gastrointestinal	NR	NR	NR
Bhatia, 2008	120	Adults	100	I or II	Obstetrics & Gynaecological	NR	NR	NR
Candiotti, 2008	546	Adults & Elderly	95	I or II or III	Miscellaneous	YES	YES	NR
Choi, 2008	94	Adults	100	NR	Orthopaedic	YES	YES	NR
Contreras-Dominguez, 2008	150	Adults	49	I or II	Gastrointestinal	NR	YES	NR
Cruz, 2008	372	Adults	98	NR	Miscellaneous	NR	NR	NR
Davis, 2008	200	Children	54	I or II	General dentistry, oral and maxillofacial surgery and	YES	YES	NR

					orthodontics			
Erhan, 2008	80	Adults & Elderly	76	I or II	Gastrointestinal	NR	NR	NR
Gautam, 2008	142	Adults	90	I or II	Gastrointestinal	YES	YES	NR
Gupta, 2008	60	Adults	100	I or II	Gastrointestinal	NR	NR	NR
Kaki, 2008	75	Adults	70	I or II	Gastrointestinal	NR	NR	NR
Kovac, 2008	544	Adults	100	I or II or III	Miscellaneous	YES	YES	NR
Krobbuaban, 2008	380	Adults	100	I or II	Obstetrics & Gynaecological	YES	YES	NR
Lee, 2008	236	Adults	100	I or II	Breast	NR	NR	NR
Ng, 2008	81	Adults & Elderly	6	I or II	Gastrointestinal	NR	NR	NR
Olatosi, 2008	75	Adults	100	I or II or III	Obstetrics & Gynaecological	NR	NR	NR
Pan, 2008	60	Adults	100	I or II	Obstetrics & Gynaecological	YES	YES	NR
Piper, 2008	83	Adults	56	I or II	General dentistry, oral and maxillofacial surgery and orthodontics	YES	YES	NR
Said-Ahmed, 2008	50	Adults	100	I or II	Obstetrics & Gynaecological	NR	NR	NR
Sajedi, 2008	66	Adults	35	I or II	Orthopaedic	NR	NR	NR
Sakellaris, 2008	164	Children	NR	I or II	Miscellaneous	NR	NR	NR
Schnaider, 2008	70	Adults	100	I or II	Obstetrics & Gynaecological	NR	NR	NR
Aouad, 2007	89	Adults	100	I or II	Obstetrics & Gynaecological	YES	YES	NR
Balki, 2007	176	Adults	100	I or II	Obstetrics & Gynaecological	NR	NR	NR
Bestas, 2007	90	Adults	77	I or II	Gastrointestinal	NR	NR	NR
Bhatagnar, 2007	90	Adults	100	I or II	Breast	YES	YES	NR
Bolton, 2007	557	Children	56	I or II or III	General dentistry, oral and maxillofacial surgery and orthodontics	NR	NR	NR
Boonmak, 2007	160	Adults	66	I or II	Miscellaneous	NR	NR	NR
Candiotti, 2007	88	Adults	100	I or II	Miscellaneous	YES	YES	NR
Diemunsch, 2007	892	Adults & Elderly	91	I or II or III	Miscellaneous	YES	YES	NR
Gan, 2007	766	Adults & Elderly	94	I or II or III	Miscellaneous	YES	YES	NR
Gombar, 2007	90	Children	43	I or II	Otolaryngological	NR	NR	NR
Gulhas, 2007	72	Adults	100	I or II	Obstetrics & Gynaecological	NR	NR	NR
Han, 2007	80	Adults	100	I	Obstetrics & Gynaecological	NR	NR	NR
Harnett, 2007	240	Adults	100	I	Obstetrics & Gynaecological	NR	NR	NR
Kushwaha, 2007	125	Adults & Children	50	I or II	Otolaryngological	NR	NR	NR
Lee, 2007	86	Adults	100	I or II	Obstetrics & Gynaecological	YES	YES	NR
Moussa, 2007	120	Adults	70	II or III	Gastrointestinal	YES	YES	NR
Oksuz, 2007	75	Adults & Elderly	65	I or II	Gastrointestinal	NR	YES	NR
Paech, 2007	614	Adults	100	NR	Obstetrics & Gynaecological	NR	YES	NR
Rusch, 2007	228	Adults & Elderly	49	I or II or III	Miscellaneous	NR	YES	NR
Sagir, 2007	80	Adults	0.7	I or II	Urological	NR	NR	NR
Sandhu, 2007	80	Adults	71	I or II	Gastrointestinal	NR	NR	NR

Sarkar, 2007	60	Adults	100	I or II	Obstetrics & Gynaecological	NR	NR	NR
Sharma, 2007	40	Adults	100	I or II	Gastrointestinal	NR	NR	NR
Siddik-Sayyid, 2007	129	Adults	100	I or II	Obstetrics & Gynaecological	NR	NR	Obesity
Sodhi, 2007	90	Adults	100	I or II	Breast	NR	NR	NR
Sriraman, 2007	100	Adults	100	I or II	Obstetrics & Gynaecological	YES	YES	NR
Subramaniam, 2007	75	Children	30	II or III	Neurological	NR	NR	NR
Teran, 2007	35	Adults	100	I or II	Miscellaneous	YES	YES	NR
White, 2007	66	Adults	85	I or II or III	Miscellaneous	YES	YES	NR
Wig, 2007	70	Adults	40	NR	Neurological	YES	YES	NR
Akin, 2006	70	Adults & Elderly	83	NR	Endocrine	NR	NR	NR
Birmingham, 2006	100	Adults	82	I or II	Miscellaneous	NR	NR	NR
Bridge, 2006	194	Adults	100	NR	Miscellaneous	NR	NR	NR
Chan, 2006	394	Adults	100	I or II	Obstetrics & Gynaecological	YES	YES	NR
Eberhart, 2006	310	Adults	72	I or II or III	Miscellaneous	NR	YES	NR
Gunter, 2006	240	Children	NR	I or II	General dentistry, oral and maxillofacial surgery and orthodontics	NR	NR	NR
Gupta, 2006	200	Adults & Elderly	67	I or II	Gastrointestinal	NR	NR	NR
Isik, 2006	60	Adults	48	I or II	General dentistry, oral and maxillofacial surgery and orthodontics	NR	NR	NR
Janicki, 2006	150	Adults	84	I or II or III	Miscellaneous	NR	NR	NR
Jellish, 2006	120	Adults & Elderly	44	I or II or III	Neurological	NR	YES	NR
Johns, 2006	960	Adults	100	NR	Obstetrics & Gynaecological	YES	YES	NR
Khan, 2006	180	Adults	NR	I or II	Gastrointestinal	NR	NR	NR
Kolm, 2006	100	All groups	NR	I or II	Miscellaneous	NR	NR	NR
Leksowski, 2006	210	Adults & Elderly	75	I or II	Gastrointestinal	YES	YES	NR
Paranhos de Abreu, 2006	100	Adults	100	I or II	Obstetrics & Gynaecological	NR	NR	NR
Peixoto, 2006	120	Adults	100	I or II	Obstetrics & Gynaecological	NR	NR	NR
Purhonen, 2006a	56	Adults & Elderly	100	I or II or III	Breast	YES	YES	NR
Purhonen, 2000b	100	Adults	100	I or II	Obstetrics & Gynaecological	YES	YES	NR
Sarvela, 2006	87	Adults	100	NR	Obstetrics & Gynaecological	NR	NR	NR
Tosun, 2006	125	Children	49	I or II	Eye	NR	NR	NR
Tseng, 2006	140	Adults	100	I or II	Obstetrics & Gynaecological	NR	YES	NR
Veneziani, 2006	100	Adults & Elderly	100	I or II	Obstetrics & Gynaecological	NR	NR	NR
White, 2006	205	Adults	88	NR	Miscellaneous	YES	YES	NR
Cohen, 2005	62	Children	56	I or II	General dentistry, oral and maxillofacial surgery and orthodontics	NR	NR	NR
D'Angelo, 2005	121	Adults	100	I or II or III	Obstetrics & Gynaecological	YES	YES	NR

Demiraran, 2005	90	Adults	100	I or II or III	Obstetrics & Gynaecological	NR	NR	NR
Gan, 2005	176	Adults	100	I or II or III	Obstetrics & Gynaecological	YES	YES	NR
Hartsell, 2005	60	Adults & Elderly	63	I or II or III	Otolaryngological	NR	NR	NR
Iatrou, 2005	105	Adults & Elderly	46	I or II or III	Miscellaneous	NR	NR	NR
Kabalak, 2005	60	Children	NR	I or II	General dentistry, oral and maxillofacial surgery and orthodontics	NR	NR	NR
Khan, 2005	60	Adults	100	I or II	Obstetrics & Gynaecological	NR	NR	NR
Kocamanoglu, 2005	150	Adults	100	I or II	Obstetrics & Gynaecological	YES	YES	NR
Kontrimaviciute, 2005	34	Adults	100	I or II	Obstetrics & Gynaecological	NR	YES	NR
Misra, 2005	79	Adults	67	I or II	Otolaryngological	YES	YES	NR
Pirat, 2005	150	Adults	0	I	Miscellaneous	NR	NR	NR
Treschan, 2005	142	All groups	56	I or II	Eye	YES	YES	NR
White, 2005†	372	NR	100	NR	Obstetrics & Gynaecological	NR	NR	NR
Alkaissi, 2004	495	Adults	100	I or II or III	Obstetrics & Gynaecological	YES	YES	NR
Apfel, 2004 (CR: Jokela, 2009)	260	Adults	81	NR	Miscellaneous	YES	YES	NR
Barbone, 2004	141	All groups	69	I or II or III	Miscellaneous	NR	NR	NR
Barbosa, 2004	60	Adults & Elderly	85	NR	Miscellaneous	NR	YES	NR
Bhardwaj, 2004	100	Children	47	I or II	Eye	NR	NR	NR
Binstock, 2004	101	Children	42	I or II	Miscellaneous	NR	NR	NR
Browning, 2004	212	Adults & Elderly	100	I or II	Obstetrics & Gynaecological	NR	NR	NR
Celiker, 2004	102	Children	33	NR	General dentistry, oral and maxillofacial surgery and orthodontics	NR	NR	NR
Dua, 2004	60	Adults & Elderly	100	I or II	Breast	NR	YES	NR
Eberhart, 2004	304	Adults & Elderly	52	I or II or III	Eye	YES	YES	NR
Gan, 2004	49	Adults	100	I or II	Breast	YES	YES	NR
Han, 2004	350	Adults & Elderly	0	NR	Miscellaneous	NR	NR	NR
Hanaoka, 2004	315	Adults	95	I or II or III	Miscellaneous	YES	NR	NR
Meyer, 2004	92	Adults & Elderly	76	NR	Miscellaneous	NR	YES	NR
Panda, 2004	100	Adults	NR	I or II	Otolaryngological	NR	NR	NR
Samakarndi, 2004	80	Children	49	I or II	Eye	NR	NR	NR
Sinha, 2004	70	Adults	100	I or II	Breast	YES	YES	NR
Bharti, 2003	120	Children	52	I or II	Eye	YES	YES	NR
Biswas, 2003a	160	Adults	100	I or II	Obstetrics & Gynaecological	NR	NR	NR
Biswas, 2003b	120	Adults	80	I or II	Gastrointestinal	NR	NR	NR
Burmeister, 2003	40	Adults & Elderly	57	I or II	Gastrointestinal	YES	YES	NR
Charuluxananan, 2003	180	Adults	100	I or II	Obstetrics & Gynaecological	NR	NR	NR
Karamanlioglu, 2003	150	Children	49	I or II	Miscellaneous	NR	NR	NR
Loewen, 2003	71	Adults	100	NR	Breast	YES	YES	NR
Maddali, 2003	120	Adults	100	I or II	Obstetrics &	NR	NR	NR

					Gynaecological			
Madenoglu, 2003	60	Adults	52	I or II or III	Neurological	NR	NR	NR
O'Brien, 2003	150	Children	NR	I or II	Miscellaneous	YES	YES	NR
Olutoye, 2003	204	Children	27	I or II	Miscellaneous	YES	YES	NR
Oztekin, 2003	40	Adults	100	I or II	Obstetrics & Gynaecological	NR	NR	NR
Paech, 2003	118	Adults	100	NR	Obstetrics & Gynaecological	YES	YES	NR
Pan, 2003	40	Adults	100	I or II	Obstetrics & Gynaecological	NR	NR	NR
Samra, 2003[†]	NR	Adults	250	NR	Neurological	NR	NR	NR
Szarvas, 2003	130	Adults & Elderly	47	I or II or III	Orthopaedic	NR	NR	NR
Tang, 2003	135	Adults & Elderly	65	I or II or III	Miscellaneous	YES	YES	NR
Thagaard, 2003	96	Adults	69	I or II	Miscellaneous	YES	YES	NR
Tzeng, 2003	65	Adults	100	I or II	Obstetrics & Gynaecological	NR	NR	NR
Usmani, 2003	90	All groups	NR	I or II	Otolaryngological	NR	NR	NR
Wagner, 2003	94	Children	53	I or II	Eye	NR	NR	NR
Wattwil, 2003	78	Adults & Elderly	100	I or II or III	Breast	NR	NR	Cancer
Yuksek, 2003	60	Adults	100	I or II	Obstetrics & Gynaecological	NR	NR	NR
Adducci, 2002	180	Adults	70	I or II	Gastrointestinal	NR	NR	NR
Agarwal, 2002	100	Adults	67	I or II	Gastrointestinal	NR	NR	NR
Arcioni, 2002	59	Adults & Elderly	12	I or II or III	General dentistry, oral and maxillofacial surgery and orthodontics	NR	NR	NR
Argiriadou, 2002	87	Adults	76	I or II	Gastrointestinal	NR	NR	NR
Coloma, 2002	60	Adults	92	I or II or III	Miscellaneous	YES	YES	NR
Coloma, 2002	140	Adults	78	I or II	Gastrointestinal	YES	YES	NR
Elhakim, 2002	180	Adults	72	NR	Gastrointestinal	YES	YES	NR
Fabling, 2002	46	Adults & Elderly	50	I or II or III	Neurological	NR	NR	NR
Gan, 2002	60	Adults	100	I or II	Obstetrics & Gynaecological	YES	YES	NR
Goksu, 2002	60	Adults	45	I or II	Otolaryngological	NR	NR	NR
Grimsehl, 2002	74	Adults	100	I or II	Obstetrics & Gynaecological	NR	YES	NR
Jokela, 2002	179	Adults	100	I or II or III	Endocrine	YES	YES	NR
Lee, 2002	113	Adults	92	I or II	Endocrine	YES	YES	NR
Marcus, 2002	120	Adults	87	NR	Miscellaneous	YES	YES	NR
Mullick, 2002	75	Adults	100	I	Obstetrics & Gynaecological	YES	YES	NR
Munro, 2002	53	Children	39	NR	Gastrointestinal	NR	NR	NR
Ozmen, 2002	60	Adults	70	I or II	Gastrointestinal	NR	NR	NR
Paech, 2002	139	Adults	100	NR	Obstetrics & Gynaecological	YES	YES	NR
Quaynor, 2002	107	Adults	93	I or II	Gastrointestinal	YES	YES	NR
Sanchez-Ledesma, 2002	90	Adults	100	I or II	Obstetrics & Gynaecological	YES	YES	NR
Sennaraj, 2002	150	Children	58	I or II	Eye	NR	YES	NR
So, 2002	68	Adults & Elderly	53	I or II	Gastrointestinal	NR	YES	Cancer
Sukhani, 2002	149	Children	48	I or II	General dentistry, oral and maxillofacial surgery and orthodontics	NR	NR	NR
Wang, 2002a	115	Adults	62	I or II	Gastrointestinal	NR	NR	Obesity

Wang, 2002b	116	Adults	56	I or II	Otolaryngological	NR	NR	NR
Wang, 2002	114	Adults	100	I or II	Obstetrics & Gynaecological	NR	NR	NR
White, 2002	120	Adults	88	I or II or III	Miscellaneous	YES	YES	NR
Yazigi, 2002	100	Adults	100	I or II	Obstetrics & Gynaecological	NR	NR	NR
Bowhay, 2001	131	Children	40	I or II	Eye	NR	NR	NR
Cherian, 2001	81	Adults	100	NR	Obstetrics & Gynaecological	NR	NR	NR
Dabbous, 2001	173	Adults	77	I or II	Gastrointestinal	YES	YES	Diabetes
De Witte, 2001	40	Adults & Elderly	52	I or II	Orthopaedic	NR	NR	NR
Ganem, 2001	52	Adults	100	I or II or III	Obstetrics & Gynaecological	NR	NR	NR
Goll, 2001	240	Adults	100	I or II or III	Obstetrics & Gynaecological	YES	YES	NR
Kathirvel, 2001	152	Adults & Children	38	I or II	Neurological	NR	NR	NR
Millo, 2001	137	Adults	100	NR	Obstetrics & Gynaecological	NR	YES	NR
Pan, 2001	156	Adults	100	I or II	Obstetrics & Gynaecological	NR	NR	NR
Paventi, 2001	60	Adults	46	I or II	Obstetrics & Gynaecological	YES	YES	NR
Shende, 2001	240	Children	50	I or II	Eye	YES	YES	NR
Somri, 2001	60	Children	41	I	General dentistry, oral and maxillofacial surgery and orthodontics	NR	NR	NR
Splinter, 2001	193	Children	NR	NR	Eye	NR	NR	NR
Subramaniam, 2001	135	Children	54	I or II	Eye	NR	YES	NR
Thomas, 2001	177	Adults	100	I or II	Obstetrics & Gynaecological	YES	YES	NR
White, 2001	80	Adults	100	I or II	Breast	YES	YES	NR
Wilson, 2001	232	Adults & Elderly	79	I or II or III	Gastrointestinal	NR	NR	NR
Yilmazlar, 2001	60	Adults	100	I or II	Miscellaneous	NR	NR	NR
Ahmed, 2000	139	Adults	100	NR	Obstetrics & Gynaecological	YES	YES	NR
Charuluxananan, 2000	80	Adults	100	I or II	Obstetrics & Gynaecological	NR	NR	NR
Fabling, 2000	60	Adults & Elderly	53	I or II or III	Neurological	NR	NR	NR
Gesztesi, 2000	157	Adults	100	I or II	Obstetrics & Gynaecological	NR	YES	NR
Holt, 2000	125	Children	54.4	I or II	Ear, nose and larynx	YES	YES	Asthma
Jensen, 2000	71	Children	41	I or II	General dentistry, oral and maxillofacial surgery and orthodontics	YES	NR	NR
Jokela, 2000	120	Adults	100	I or II or III	Breast	YES	YES	Migraines
Kothari, 2000	108	Adults	73	I or II or III	Gastrointestinal	YES	NR	Gallbladder
Kreisler, 2000	31	Adults	81	NR	Miscellaneous	NR	YES	NR
Ku, 2000	58	Adults & Elderly	57	NR	Ear, nose and larynx	YES	YES	NR
Lee, 2000	120	Adults	100	I	Obstetrics & Gynaecological	NR	YES	NR
Liberman, 2000	84	Adults & Children	78	NR	Gastrointestinal	NR	NR	NR
Najeeb, 2000	45	Adults	62	I or II	Miscellaneous	NR	NR	NR
Peixoto, 2000	90	Adults &	100	I or II	Obstetrics &	NR	NR	NR

		Elderly			Gynaecological			
Philip, 2000	1030	Adults	70	I or II or III	Miscellaneous	YES	YES	NR
Reihner, 2000	207	Adults & Elderly	100	I or II	Breast	YES	YES	NR
Sadhasivam, 2000	180	Children	53	I or II	Eye	NR	NR	NR
Scuderi, 2000	139	Adults	100	I or II or III	Obstetrics & Gynaecological	YES	YES	NR
Sharma, 2000	98	Adults	100	I or II	Obstetrics & Gynaecological	NR	NR	NR
Wu, 2000	152	Adults	100	I or II or III	Obstetrics & Gynaecological	YES	YES	NR
Zarate, 2000	200	Adults	44	I or II	Miscellaneous	YES	YES	NR
Abouleish, 1999	74	Adults	100	I or II	Obstetrics & Gynaecological	NR	NR	NR
Allen, 1999	56	Children	41	NR	Miscellaneous	NR	NR	NR
Barst, 1999	90	Children	48	I or II	General dentistry, oral and maxillofacial surgery and orthodontics	NR	NR	NR
Broome, 1999	109	Adults	76	I or II	General dentistry, oral and maxillofacial surgery and orthodontics	NR	NR	NR
Bugedo, 1999	242	Adults	79	I or II	Miscellaneous	NR	NR	NR
Cholwill, 1999	175	Adults	100	I or II	Obstetrics & Gynaecological	YES	YES	NR
de Adana, 1999	40	Children	NR	NR	Neurological	NR	NR	NR
Gurler, 1999	160	Adults	78	I or II	Gastrointestinal	NR	NR	NR
Helmy, 1999	397	Adults	85	I or II or III	Miscellaneous	NR	YES	NR
Janknegt, 1999	120	Adults	100	I or II or III	Gastrointestinal	YES	YES	Migraines
Jokela, 1999	100	Children	50	I or II	Eye	YES	YES	NR
Kathirvel, 1999	63	Adults & Elderly	47	I or II	Otolaryngological	YES	YES	Cancer
Khalil, 1999	88	Adults	100	I or II or III	Obstetrics & Gynaecological	YES	YES	NR
Koivuranta, 1999	428	Adults	82	I or II or III	Miscellaneous	YES	YES	NR
Kovac, 1999	400	Adults	60	I or II	Gastrointestinal	NR	NR	NR
Kyokong, 1999	100	Adults & Children	50	NR	Miscellaneous	NR	YES	NR
McCall, 1999	73	Children	53	I or II	Eye	NR	NR	NR
Munro, 1999	51	Adults	100	I	Obstetrics & Gynaecological	NR	NR	NR
Rajeeva, 1999	52	Adults & Elderly	25	I or II	Gastrointestinal	NR	NR	Gallbladder
Sadhasivam, 1999	54	Adults	100	I or II	Breast	YES	YES	Cardiovascular
Sandhu, 1999	87	Adults	100	I or II	Obstetrics & Gynaecological	NR	NR	NR
Scuderi, 1999a	575	Adults	63	I or II or III	Miscellaneous	NR	YES	NR
Scuderi, 1999b	NR	Adults	NR	I or II or III	NR	NR	NR	NR
Sinha, 1999	40	Adults	47	I or II or III	Neurological	NR	YES	NR
Swiatkowski, 1999	134	Adults	100	I or II	Miscellaneous	NR	NR	Obesity
Tsui, 1999	121	Adults	100	I or II or III	Obstetrics & Gynaecological	NR	NR	NR
Turhanoglu, 1999	100	Adults	100	I or II	Obstetrics & Gynaecological	NR	NR	NR
Wagley, 1999	50	Adults	50	I or II	General dentistry, oral and maxillofacial	NR	NR	NR

					surgery and orthodontics			
Woodward, 1999	216	Adults & Elderly	18	II or III	Cardiac	NR	YES	NR
Alon, 1998	314	Adults & Elderly	92	I or II	Miscellaneous	NR	NR	NR
Ang, 1998	47	Children	45	I or II	General dentistry, oral and maxillofacial surgery and orthodontics	NR	NR	NR
Bacic, 1998	50	Adults	100	NR	Endocrine	NR	NR	NR
Chan, 1998	148	Adults	100	I or II	Breast	YES	YES	NR
Chen, 1998a	78	Adults & Elderly	37	I or II or III	Orthopaedic	NR	YES	Obesity
Chen, 1998b	80	Children	34	I or II	Miscellaneous	NR	NR	NR
Dabbous, 1998	85	Adults	62	I or II	Gastrointestinal	YES	YES	Diabetes
Daftary, 1998	150	Children	46	I	General dentistry, oral and maxillofacial surgery and orthodontics	NR	NR	NR
Dershwitz, 1998	175	Adults & Elderly	100	NR	Miscellaneous	NR	NR	NR
Diemunsch, 1998	793	Adults	100	I or II or III	Obstetrics & Gynaecological	YES	YES	NR
Doe, 1998	45	Adults & Children	42	I or II or III	Eye	NR	NR	NR
Dresner, 1998	60	Adults	100	I or II	Obstetrics & Gynaecological	NR	NR	NR
Goodarzi, 1998	81	Children	43	I or II	Orthopaedic	NR	NR	NR
Hamid, 1998	71	Children	55	I or II	General dentistry, oral and maxillofacial surgery and orthodontics	NR	YES	NR
Jellish, 1998	120	Adults	51	I or II	Otolaryngological	NR	NR	NR
Mitra, 1998	80	Adults	100	NR	Obstetrics & Gynaecological	YES	YES	NR
Morris, 1998	1044	Adults	100	I or II or III	Obstetrics & Gynaecological	NR	YES	NR
Scholz, 1998	842	Adults & Elderly	74	I or II or III	Miscellaneous	YES	YES	NR
Splinter, 1998a	197	Children	NR	NR	Eye	NR	NR	NR
Splinter, 1998b	216	Children	55	I or II or III	General dentistry, oral and maxillofacial surgery and orthodontics	NR	NR	NR
Tang, 1998a	218	Adults	100	I or II	Obstetrics & Gynaecological	YES	YES	NR
Tang, 1998b	156	Adults	100	I or II	Obstetrics & Gynaecological	YES	YES	NR
Tramer, 1998	157	Children	49	NR	Eye	YES	YES	
Akcabay, 1997	60	Adults	NR	I or II	Endocrine	NR	NR	NR
Alexander, 1997	124	All groups	61	I or II	Orthopaedic	NR	YES	NR
Ascaso, 1997	251	Adults & Elderly	49	I or II	Eye	NR	NR	NR
Bach-Styles, 1997	101	Children	NR	I or II or III	Eye	NR	NR	NR
Carnahan, 1997	54	Children	48	I or II	General dentistry, oral and maxillofacial surgery and orthodontics	NR	NR	NR
Diemunsch, 1997a	746	Adults & Elderly	91	NR	Miscellaneous	YES	YES	NR

Diemunsch, 1997b	281	Adults	100	I or II	Obstetrics & Gynaecological	NR	YES	NR
Diemunsch, 1997c	337	Adults	95	I or II	Obstetrics & Gynaecological	NR	YES	NR
Graczyk, 1997	635	Adults	100	I or II	Obstetrics & Gynaecological	NR	YES	NR
Klockgether-Radke, 1997	120	Children	48	I or II	Eye	NR	NR	NR
Koivuranta, 1997	439	Adults & Elderly	100	I or II or III	Miscellaneous	YES	YES	Migraines
Korttila, 1997	517	Adults	94	I or II or III	Miscellaneous	YES	YES	NR
Kovac, 1997	620	Adults	83	I or II or III	Miscellaneous	YES	YES	NR
Lawhorn, 1997	320	Children	NR	I or II	Miscellaneous	NR	NR	NR
Maestre, 1997	80	Adults	100	I or II or III	Obstetrics & Gynaecological	YES	YES	NR
McKenzie, 1997	233	Adults & Children	54	I or II	Miscellaneous	NR	NR	NR
Mikawa, 1997	200	Adults & Elderly	100	I	Obstetrics & Gynaecological	NR	YES	NR
Moens, 1997	206	Adults	69	NR	Miscellaneous	NR	YES	NR
Monagle, 1997	91	Adults	100	NR	Obstetrics & Gynaecological	NR	YES	NR
Morton, 1997	427	Children	51	I or II	General dentistry, oral and maxillofacial surgery and orthodontics	NR	YES	NR
Patel, 1997	429	Children	37	I or II or III	Miscellaneous	YES	YES	NR
Pitkanen, 1997	73	Adults & Elderly	NR	I or II or III	Orthopaedic	NR	NR	NR
Polati, 1997	175	Adults	100	I or II	Obstetrics & Gynaecological	YES	YES	NR
Purhonen, 1997	146	Adults	100	I or II or III	Miscellaneous	YES	YES	NR
Rung, 1997	121	Adults & Elderly	69	I or II or III	Miscellaneous	YES	YES	NR
Scuderi, 1997	160	Children	52	I or II	Eye	NR	YES	NR
Shende, 1997	176	Children	49	I	Eye	NR	NR	NR
Sniadach, 1997	158	Adults	100	I or II	Obstetrics & Gynaecological	NR	NR	NR
Splinter, 1997	239	Children	NR	I or II	General dentistry, oral and maxillofacial surgery and orthodontics	NR	NR	NR
Stienstra, 1997	74	Adults	100	I or II	Obstetrics & Gynaecological	NR	NR	NR
Sun, 1997	75	Adults & Elderly	33	I or II	Otolaryngological	YES	YES	NR
Taylor, 1997	523	Adults & Elderly	89	I or II or III	Multiple types included	YES	YES	NR
Ulusoy, 1997	40	Adults	37	I or II	Miscellaneous	NR	NR	NR
Warriner, 1997	374	Adults & Elderly	100	I or II	Obstetrics & Gynaecological	YES	YES	NR
Ali-melkkila, 1996	120	Adults	42	I or II or III	Eye	NR	YES	NR
Alon, 1996	80	All groups	100	I or II	Obstetrics & Gynaecological	NR	YES	NR
Capouet, 1996	385	Adults	100	I or II	Miscellaneous	YES	YES	NR
Chen, 1996	49	Adults	100	I or II	Obstetrics & Gynaecological	YES	YES	NR
Cieslak, 1996	97	Children	48	I or II	Miscellaneous	NR	NR	NR
Davies, 1996	66	Adults & Elderly	100	I or II	Obstetrics & Gynaecological	YES	YES	NR
Ercelen, 1996	100	Children	48	I	Eye	NR	NR	NR

Eriksson, 1996	58	Adults	100	I or II	Obstetrics & Gynaecological	NR	NR	NR
Furst, 1996	60	Children	45	NR	Neurological	NR	NR	NR
Gaynes, 1996[†]	87	NR	NR	NR	Eye	NR	NR	NR
Honkavaara, 1996	241	Adults	55	I or II	Miscellaneous	NR	NR	NR
Kaul, 1996	375	Children	40	I or II or III	Miscellaneous	YES	YES	NR
Khalil, 1996	140	Adults	NR	NR	Obstetrics & Gynaecological	NR	NR	NR
Kimya, 1996	63	Adults & Elderly	78	I or II	Gastrointestinal	YES	YES	NR
Koivuranta, 1996	468	Adults	0	I or II	Miscellaneous	YES	YES	NR
Kovac, 1996 (CR: Pearman, 1994c)	30	Adults	100	I or II	Obstetrics & Gynaecological	NR	NR	NR
Kumar, 1996	165	Children	NR	I or II	General dentistry, oral and maxillofacial surgery and orthodontics	NR	NR	NR
Lawhorn, 1996	82	Adults	74	I or II	Gastrointestinal	NR	NR	NR
Lekprasert, 1996	100	Adults	100	I or II	Obstetrics & Gynaecological	YES	YES	NR
Lopez-Orlaondo, 1996	132	Adults & Elderly	82	I or II	Gastrointestinal	NR	NR	NR
Naguib, 1996	48	Adults	100	I or II	Obstetrics & Gynaecological	NR	NR	NR
Pan, 1996	60	Adults & Elderly	100	I or II	Obstetrics & Gynaecological	YES	YES	NR
Principi, 1996	100	Adults	100	I or II	Miscellaneous	YES	YES	NR
Pueyo, 1996	60	Adults	62	I or II or III	Neurological	YES	YES	NR
Pugh, 1996	92	Adults	100	NR	Miscellaneous	NR	NR	NR
Rodrigo, 1996	32	Adults & Elderly	34	NR	Miscellaneous	NR	NR	NR
Rose, 1996a	136	Children	44	I or II	General dentistry, oral and maxillofacial surgery and orthodontics	NR	NR	NR
Rose, 1996b	200	Children	48	I or II	General dentistry, oral and maxillofacial surgery and orthodontics	NR	NR	NR
Stene, 1996	120	Children	NR	I or II	General dentistry, oral and maxillofacial surgery and orthodontics	NR	NR	NR
Tang, 1996	161	Adults	100	I or II	Obstetrics & Gynaecological	YES	YES	NR
Usha, 1996	148	Adults & Children	47	I or II	Otolaryngological	YES	YES	NR
Van Den Berg, 1996a	282	Adults & Children	39	I or II	General dentistry, oral and maxillofacial surgery and orthodontics	NR	NR	NR
Van Den Berg, 1996b	220	Adults & Children	21	I or II	Otolaryngological	YES	YES	NR
Van Den Berg, 1996c	167	Adults	100	NR	Obstetrics & Gynaecological	YES	YES	NR
Watts, 1996	527	Adults & Elderly	96	I or II or III	Miscellaneous	YES	YES	NR
Wilson, 1996	60	Adults	100	I or II	Obstetrics & Gynaecological	NR	YES	NR
Wrench, 1996	60	Adults	82	I or II	Endocrine	NR	NR	NR
Yilmazlar, 1996	127	All	61	I or II	Orthopaedic	NR	YES	NR

		groups						
Alexander, 1995	32	Adults	47	I or II	Miscellaneous	YES	YES	NR
Campbell, 1995	213	Adults & Children	39	I or II	Eye	NR	NR	NR
Davis, 1995a	95	Children	NR	I or II	General dentistry, oral and maxillofacial surgery and orthodontics	NR	NR	NR
Davis, 1995b	286	Adults	100	I or II or III	Obstetrics & Gynaecological	YES	YES	Diabetes
Desilva, 1995	50	Adults	70	I or II	Gastrointestinal	NR	NR	NR
Elhakim, 1995	80	Adults	100	I or II	Obstetrics & Gynaecological	YES	YES	NR
Grond, 1995	49	Adults & Children	41	I or II	Ear, nose and larynx	YES	YES	NR
Litman, 1995	57	Children	40	I or II	Eye	YES	YES	NR
Mikawa, 1995	120	Adults	100	I	Obstetrics & Gynaecological	NR	YES	NR
Paech, 1995	259	Adults	100	NR	Obstetrics & Gynaecological	NR	YES	NR
Paxton, 1995a	60	Children	47	NR	Ear, nose and larynx	NR	NR	NR
Paxton, 1995b	118	Adults	100	NR	Obstetrics & Gynaecological	NR	YES	NR
Splinter, 1995a	233	Children	NR	NR	General dentistry, oral and maxillofacial surgery and orthodontics	NR	NR	NR
Splinter, 1995b	276	Children	NR	NR	General dentistry, oral and maxillofacial surgery and orthodontics	NR	NR	NR
Watcha, 1995	130	Children	51	I or II	Multiple types included	YES	YES	NR
Furst, 1994	239	Children	49	I or II	General dentistry, oral and maxillofacial surgery and orthodontics	NR	NR	NR
Gan, 1994	120	All groups	56	I or II	Orthopaedic	NR	NR	NR
Kaufmann, 1994	286	Adults	54	I or II	Orthopaedic	NR	NR	NR
Khalil, 1994 (CR: Pearman, 1994b)	589	Adults & Elderly	100	I or II	Miscellaneous	YES	YES	NR
Litman, 1994	60	Children	57	I or II	General dentistry, oral and maxillofacial surgery and orthodontics	NR	NR	NR
Malins, 1994	180	Adults & Elderly	100	I or II	Obstetrics & Gynaecological	YES	YES	NR
McKenzie, 1994	150	Adults	100	I or II	Obstetrics & Gynaecological	NR	NR	NR
Rodrigo, 1994	77	Adults	65	I or II	General dentistry, oral and maxillofacial surgery and orthodontics	NR	NR	NR
Rose, 1994	100	Children	51	I or II	Eye	NR	NR	NR
Suen, 1994	204	Adults	100	I or II	Obstetrics & Gynaecological	NR	NR	NR
Ummenhofer, 1994	200	Children	34	I or II	Miscellaneous	NR	NR	NR
Volpe, 1994	928	Adults & Elderly	57	I or II	Miscellaneous	NR	NR	NR
Dupeyron, 1993	243	Adults &	100	I or II	Obstetrics &	NR	NR	Cardiovascular,

		Elderly		or III	Gynaecological			urological
Helmers, 1993	923	Adults	100	I or II or III	Obstetrics & Gynaecological	NR	NR	NR
McKenzie, 1993b (CR: Dershwitz, 1992)	137	Adults	100	I or II or III	Miscellaneous	NR	YES	NR
Raphael, 1993	123	Adults	100	NR	Obstetrics & Gynaecological	YES	YES	NR
Sung, 1993	180	Adults	100	I or II	Obstetrics & Gynaecological	NR	YES	NR
Zomers, 1993	69	Adults & Elderly	100	NR	Obstetrics & Gynaecological	NR	YES	Cardiovascular, diabetes
Alon, 1992	66	Adults & Children	100	I	Obstetrics & Gynaecological	NR	NR	NR
Du Pen, 1992 (CR: Scuderi, 1993)	500	All groups	11	I or II	Miscellaneous	NR	NR	NR
Kenny, 1992	982	Adults & Elderly	100	I or II or III	Obstetrics & Gynaecological	NR	NR	NR
Kovac, 1992 (CRs: McKenzie, 1993a; Pearman, 1994a)	580	Adults	100	I or II	Obstetrics & Gynaecological	NR	YES	NR
Bodner, 1991	71	Adults	100	NR	Obstetrics & Gynaecological	NR	YES	NR
Larijani, 1991	36	Adults	94	I or II or III	NR	NR	NR	NR
Leeser, 1991	84	Adults	100	I or II or III	Obstetrics & Gynaecological	NR	NR	NR
Non-randomized clinical trials (n=5)								
Stamer, 2011	141	Adults & Elderly	45	I or II or III	Miscellaneous	NR	YES	NR
Rabbani, 2010	60	Adults & Children	30	I or II	General dentistry, oral and maxillofacial surgery and orthodontics	YES	NR	NR
Kim, 2007	2839	Adults	NR	NR	NR	NR	NR	NR
Biedler, 2004	159	Adults	57	I or II or III	Miscellaneous	YES	YES	NR
Manani, 1996	100	Children	100	NR	Eye	NR	NR	NR
Controlled before-after studies (n=1)								
Wagner, 2007	66	Children	54	I or II	Eye	NR	NR	NR
Cohort studies (n=9)								
Shakeel, 2012	432	Children	50	NR	General dentistry, oral and maxillofacial surgery and orthodontics	NR	NR	NR
Chen, 2011	90	Adults & Elderly	69	I or II or III	Orthopaedic	NR	NR	NR
Eryilmaz, 2008	212	Adults & Children	84	NR	Miscellaneous	YES	YES	NR
Habib, 2007	3814	Adults & Elderly	66	NR	Miscellaneous	NR	NR	NR
Flynn, 2006	849	NR		NR	Neurological	NR	NR	NR
Piwko, 2005	38	Children	NR	I or II	Eye	NR	NR	NR
Wang, 2005	282	Adults & Elderly	NR	NR	Miscellaneous	NR	NR	NR
Walker, 2001	59	Adults	85	NR	Miscellaneous	NR	YES	NR
O'Brien, 2000	200	NR	NR	NR	NR	NR	NR	NR

Note: †Unpublished data (Gurha, 2012; Apfel, 2011; White, 2005; Samra, 2003; Gaynes, 1996).
Abbreviations: ASA, American Society of Anaesthesiologists physical classification system; NR, not reported or No; CR, companion report; PONV, post-operative nausea and vomiting; H/o, History of.

Appendix 7. Cochrane EPOC Risk-of-bias appraisal results

Author, year	1	2	3	4	5	6	7	8	9
Randomized clinical trials (n=429)									
Browning, 2013	Low	Low	Unclear	Low	Low	Low	Low	Unclear	Low
Aleyasin, 2012	Low	Unclear	Unclear	Low	Unclear	Unclear	Low	Unclear	Unclear
Blitz, 2012	Unclear	Unclear	Unclear	Low	Unclear	Unclear	Low	Low	Low
Choi, 2012	Low	Low	Unclear	Low	Unclear	Low	Low	Unclear	Unclear
de Orange, 2012	Low	Low	Unclear	Low	Low	Low	Unclear	Low	Unclear
Eidi, 2012	Low	Low	Unclear	Low	Unclear	Low	Low	Low	Low
Gurha, 2012[†]	Unclear	Unclear	Unclear	Unclear	Unclear	Unclear	Unclear	Unclear	Unclear
Koh, 2012	Low	Unclear	Low	Low	High	Low	Unclear	Low	Unclear
Lahsaei, 2012	Unclear	High	Unclear	Unclear	Unclear	Low	Low	Unclear	Unclear
Madenoglu, 2012	Unclear	Low	Unclear	Low	Unclear	Low	Low	Unclear	Unclear
Moon, 2012	Low	Low	Unclear	Low	Low	Low	Low	Unclear	Low
Park JW, 2012	Unclear	Unclear	Unclear	Low	Unclear	Unclear	Unclear	Unclear	Unclear
Wang, 2012	Low	High	Unclear	Low	Low	Low	Unclear	Unclear	Unclear
Ahmed, 2011	Low	Low	Unclear	Low	Unclear	Unclear	Low	Unclear	Unclear
Apfel, 2011[†]	Unclear	Unclear	Unclear	Unclear	Unclear	Unclear	Unclear	Unclear	Unclear
Asl, 2011	Unclear	Unclear	Unclear	Low	Unclear	Low	Unclear	Unclear	Unclear
Bajwa, 2011	Low	Unclear	Unclear	Low	Unclear	Unclear	Low	Unclear	Unclear
Basu, 2011	Unclear	Unclear	Unclear	Low	Low	Low	Low	Unclear	High
Bhattarai, 2011	Unclear	Unclear	Unclear	Low	Unclear	Unclear	Low	Unclear	Unclear
Bicer, 2011	Low	Low	Unclear	Low	Unclear	Low	Low	Unclear	Unclear
D'Souza, 2011	Unclear	Unclear	Unclear	Low	Low	High	Unclear	Unclear	Unclear
Ekinci, 2011	Low	Unclear	Unclear	Low	Low	Low	Low	Unclear	Unclear
El-deeb, 2011a	Low	Low	Unclear	Low	Unclear	Low	Low	Unclear	Unclear
El-deeb, 2011b	Low	Low	Unclear	Low	Unclear	Low	Low	Unclear	Unclear
El-deeb, 2011c	Low	Unclear	Unclear	Low	Unclear	Low	Low	Unclear	Unclear
Gan, 2011	Low	Low	Unclear	Low	High	Low	Unclear	Low	Low
Islam, 2011	Unclear	Unclear	Unclear	Low	Unclear	Unclear	Low	Unclear	Unclear
Khan, 2011	Unclear	Unclear	Unclear	Low	Unclear	Unclear	Low	Unclear	Unclear
Lee, 2011	Low	Low	Unclear	Low	Unclear	Low	Low	Unclear	Unclear
Metaxari, 2011	Low	Unclear	Unclear	Low	High	Low	Low	Unclear	Unclear
Park, 2011	Low	Unclear	Unclear	Low	Low	Low	Low	Unclear	Low
Ryu, 2011	Low	Low	Unclear	Low	Low	Low	Low	Low	Low
Safae, 2011	Unclear	Unclear	Unclear	Low	Unclear	Unclear	Low	Unclear	Unclear
Sahoo, 2011	Low	Unclear	Unclear	Low	Unclear	Low	Low	Unclear	Unclear
Singh, 2011	Unclear	Unclear	Unclear	Low	Unclear	Low	Low	Unclear	Unclear
Song, 2011	Low	Low	Unclear	Low	Low	Unclear	Low	Low	Unclear
Srivastava, 2011	Unclear	Unclear	Unclear	Low	Unclear	Unclear	Low	Unclear	Unclear
Talesh, 2011	Unclear	Low	Unclear	Unclear	Unclear	Unclear	Unclear	Unclear	Unclear
Vale, 2011	Low	Low	Unclear	Low	Low	Low	Low	Unclear	Low
Akhtar, 2010	Low	Unclear	Unclear	Low	Unclear	Low	Low	Unclear	Unclear
Alghanem, 2010	Low	Low	Unclear	Low	Low	Low	Low	Low	Unclear
Bhattachajee, 2010	Low	Low	Unclear	Low	Unclear	Unclear	Low	Unclear	Unclear
Bilgin, 2010	Low	Low	Unclear	Low	Low	Low	Low	Unclear	Unclear
Choi, 2010	Low	Low	Low	Low	High	Unclear	Low	Unclear	Unclear
Dabbous, 2010	Low	Low	Unclear	Low	High	Low	Low	Unclear	Unclear
Ebrahim Soltani, 2010 (CR: Ebrahim Soltani, 2011)	Low	Unclear	Unclear	Low	Unclear	Low	Low	Unclear	Unclear
Hahm, 2010	Low	Unclear	Unclear	Low	Low	Low	Low	Unclear	Unclear
Huh, 2010	Low	Unclear	Unclear	Low	Unclear	Low	Unclear	Unclear	Unclear
Jee, 2010	Low	Unclear	Unclear	Low	Unclear	Unclear	Unclear	Unclear	Unclear
Jokela, 2010	Low	Unclear	Unclear	Low	High	Low	Low	Unclear	Unclear
Mishra, 2010	Unclear	Unclear	Unclear	Low	Unclear	Unclear	Unclear	Unclear	Unclear
Omar, 2010	Low	Unclear	Unclear	Low	Unclear	Unclear	Low	Unclear	Unclear
Rauers, 2010	Low	Low	Unclear	Low	Low	Low	Low	Unclear	Unclear
Ryu, 2010	Low	Low	Unclear	Low	Low	Low	Low	Unclear	Unclear
Shakya, 2010	Unclear	Unclear	Unclear	Low	Unclear	Unclear	Unclear	Unclear	Low
Singla, 2010	Unclear	Unclear	Unclear	Low	Low	Low	Unclear	Low	Unclear
Tan, 2010	Low	Unclear	Unclear	Low	Unclear	Low	Low	Unclear	Unclear
Yun, 2010	Low	Low	Unclear	Low	Low	Low	Low	Unclear	Unclear
Farouk, 2009	Unclear	Unclear	Unclear	Low	Low	Low	Low	Unclear	Unclear

Feng, 2009	Low	Low	Low	Low	Low	Low	Low	Unclear	Low
Gan, 2009	Low	Low	Unclear	Low	Low	Low	Unclear	Unclear	Unclear
Gandhi, 2009	Unclear	Low	Unclear	Low	Low	Unclear	Low	Unclear	Unclear
Grover, 2009	Low	Low	Unclear	Low	Low	Low	Unclear	Unclear	Unclear
Jain, 2009	Low	Unclear	Unclear	Low	High	Unclear	Unclear	Unclear	Unclear
Jellish, 2009	Unclear	Low	Unclear	High	High	Unclear	Low	Unclear	Unclear
Kim, 2009	Low	Low	Unclear	Low	Low	Low	Low	Unclear	Unclear
Riad, 2009	Unclear	Low	Unclear	Low	Low	Low	Low	Unclear	Low
Rosow, 2009	Unclear	High	Unclear	Low	Low	Low	Unclear	Unclear	High
Aghadavoudi, 2008	Low	Unclear	Unclear	Low	Low	Low	Low	Unclear	Unclear
Bano, 2008	Low	Unclear	Unclear	Low	Low	Low	Low	Unclear	Unclear
Bhatia, 2008	Unclear	Unclear	Unclear	Low	Unclear	Unclear	Low	Unclear	Unclear
Candiotti, 2008	Low	Low	Low	Low	Low	Unclear	Low	Unclear	Unclear
Choi, 2008	Low	Low	Unclear	Low	Unclear	Low	Low	Unclear	Unclear
Contreras-Dominguez, 2008	Unclear	Unclear	Unclear	Low	Unclear	Low	Low	Unclear	Unclear
Cruz, 2008	Unclear	Low	Unclear	Low	Unclear	Low	Low	Unclear	Unclear
Davis, 2008	Low	Low	Unclear	Low	High	Low	Low	Unclear	Unclear
Erhan, 2008	Unclear	Low	Unclear	Low	Unclear	Low	Low	Unclear	Unclear
Gautam, 2008	Low	Low	Unclear	Low	Low	Low	Low	Unclear	Unclear
Gupta, 2008	Unclear	Unclear	Unclear	Low	Unclear	Low	Low	Unclear	Unclear
Kaki, 2008	Unclear	Unclear	Unclear	Low	Low	Low	Low	Unclear	Unclear
Kovac, 2008	Low	Unclear	Unclear	Low	Unclear	Unclear	Low	Unclear	Unclear
Krobbuaban, 2008	Low	Low	Unclear	Low	Low	Low	Low	Unclear	Unclear
Lee, 2008	Low	Unclear	Unclear	Low	Low	Low	Low	Unclear	High
Ng, 2008	Low	Low	Unclear	Low	Unclear	Low	Unclear	Unclear	High
Olatosi, 2008	Low	Unclear	Unclear	Low	Unclear	Low	Unclear	Unclear	Unclear
Pan, 2008	Low	Low	Unclear	Low	Low	Unclear	Unclear	Unclear	Unclear
Piper, 2008	Unclear	Low	Unclear	Low	Unclear	Low	Low	Low	Low
Said-Ahmed, 2008	Unclear	Low	Unclear	Low	Unclear	Low	Low	Unclear	Unclear
Sajedi, 2008	Low	Unclear	Unclear	Low	Low	Low	Unclear	Unclear	Unclear
Sakellaris, 2008	Low	Low	Unclear	Low	Unclear	Unclear	Low	Unclear	Unclear
Schnaider, 2008	Unclear	Unclear	Unclear	Low	Unclear	Unclear	Unclear	Unclear	Unclear
Aouad, 2007	Low	Unclear	Unclear	Low	High	Low	Low	Unclear	Unclear
Balki, 2007	Low	Low	Unclear	Low	Low	Unclear	Low	Unclear	Unclear
Bestas, 2007	Unclear	Unclear	Unclear	Low	Low	Low	Low	Unclear	Unclear
Bhatagnar, 2007	Low	Unclear	Unclear	Low	Low	Low	Low	Unclear	Unclear
Bolton, 2007	Low	Low	Unclear	Low	Unclear	Low	Low	Unclear	Unclear
Boonmak, 2007	Low	Unclear	Unclear	Low	High	Low	Low	Low	Unclear
Candiotti, 2007	Low	Low	Low	High	Low	Unclear	Low	Unclear	Unclear
Diemunsch, 2007	Low	Low	Unclear	Low	Unclear	Low	Unclear	Low	High
Gan, 2007	Low	Low	Unclear	Low	Low	Low	Low	Unclear	Unclear
Gombar, 2007	Low	Low	Unclear	Low	Low	Low	Low	Unclear	Unclear
Gulhas, 2007	Low	Low	Unclear	Low	Low	Unclear	Low	Unclear	Unclear
Han, 2007	Unclear	Unclear	Unclear	Low	Low	Low	Unclear	Unclear	Unclear
Harnett, 2007	Low	Unclear	Unclear	Low	Unclear	Unclear	Unclear	Unclear	Unclear
Kushwaha, 2007	Unclear	Unclear	Unclear	Low	Unclear	Unclear	Low	Unclear	Unclear
Lee, 2007	Low	Low	Low	Low	Low	Low	Low	Unclear	Low
Moussa, 2007	Low	Low	Unclear	Low	Unclear	Low	Low	Unclear	Low
Oksuz, 2007	Unclear	Unclear	Unclear	Low	Low	Low	Low	Unclear	Low
Paech, 2007	Low	Low	Unclear	Low	Low	Low	Unclear	Unclear	Unclear
Rusch, 2007	Low	Unclear	Low	Low	Unclear	Low	Unclear	Unclear	Low
Sagir, 2007	Low	Low	Unclear	Low	Unclear	Low	Low	Unclear	Unclear
Sandhu, 2007	Unclear	Low	Unclear	Low	Low	Low	Low	Unclear	Unclear
Sarkar, 2007	Unclear	Unclear	Unclear	Unclear	Unclear	Unclear	Unclear	Unclear	Unclear
Sharma, 2007	Unclear	Unclear	Unclear	Low	Unclear	High	Unclear	Unclear	Unclear
Siddik-Sayyid, 2007	Low	Low	Unclear	Low	Low	Low	Low	Unclear	Low
Sodhi, 2007	Unclear	Unclear	Unclear	Low	Unclear	Unclear	Unclear	Unclear	Unclear
Sriraman, 2007	Low	Low	Unclear	Low	Unclear	Low	Unclear	Unclear	Unclear
Subramaniam, 2007	Low	Low	Unclear	Low	Low	Low	Low	Unclear	Unclear
Teran, 2007	Low	Unclear	Low	Low	Low	High	Unclear	Unclear	Unclear
White, 2007	Low	Low	Unclear	Low	Low	Low	Low	Unclear	Unclear
Wig, 2007	Low	Low	Low	Low	Unclear	Unclear	Low	Unclear	Low
Akin, 2006	Unclear	Unclear	Unclear	Low	Unclear	Unclear	Low	Unclear	Low
Birmingham, 2006	Unclear	Unclear	Unclear	Low	Unclear	Low	Low	Unclear	Unclear

Bridge, 2006	Unclear	Low	Unclear	Low	Unclear	Unclear	Low	Unclear	Unclear
Chan, 2006	Low	Low	Low	Low	Low	Low	Low	Unclear	Low
Eberhart, 2006	Unclear	Low	Low	High	Low	Low	Low	Unclear	Unclear
Gunter, 2006	Low	Low	Unclear	Low	Unclear	Low	Low	Unclear	Unclear
Gupta, 2006	High	Unclear	Unclear	Unclear	Unclear	Unclear	Unclear	Unclear	Unclear
Isik, 2006	Unclear	Unclear	Low	Low	Unclear	Unclear	Low	Unclear	Unclear
Janicki, 2006	Low	Unclear	Unclear	Low	Low	Low	Low	Unclear	Unclear
Jellish, 2006	Low	Unclear	Unclear	High	Unclear	Low	Low	Unclear	Unclear
Johns, 2006	Low	Unclear	Unclear	Low	Low	Unclear	Unclear	Low	Unclear
Khan, 2006	Low	Unclear	Unclear	Low	Unclear	Unclear	Low	Unclear	Unclear
Kolm, 2006	Unclear	Unclear	Unclear	Low	Unclear	Unclear	Unclear	Unclear	Unclear
Leksowski, 2006	Unclear	Unclear	Unclear	Unclear	Unclear	Low	Unclear	Unclear	Unclear
Paranhos de Abreu, 2006	Unclear	Unclear	Unclear	Low	Unclear	Unclear	Low	Unclear	Unclear
Peixoto, 2006	Unclear	Low	Unclear	Low	Low	Low	Low	Unclear	Unclear
Purhonen, 2006a	Low	Unclear	Unclear	Low	Low	Low	Low	Unclear	Unclear
Purhonen, 2000b	Low	Low	Low	Low	Unclear	Low	Unclear	Unclear	Unclear
Sarvela, 2006	Unclear	Low	Low	Low	High	Low	Low	Unclear	Unclear
Tosun, 2006	Unclear	Unclear	Unclear	Low	Unclear	Unclear	Unclear	Unclear	Unclear
Tseng, 2006	Low	Low	Unclear	Low	Unclear	Low	Low	Unclear	Unclear
Veneziani, 2006	Unclear	Unclear	Unclear	Low	Unclear	Low	Low	Unclear	Unclear
White, 2006	Low	Low	Unclear	Low	Low	Low	Low	Unclear	Low
Cohen, 2005	Unclear	Unclear	Unclear	Low	Low	Low	Low	Unclear	Unclear
D'Angelo, 2005	Low	Low	Unclear	High	Low	Low	Low	Unclear	Unclear
Demiraran, 2005	Unclear	Unclear	Unclear	Low	Low	Low	Unclear	Unclear	Unclear
Gan, 2005	Low	Low	Unclear	Low	Low	Low	Low	Unclear	Unclear
Hartsell, 2005	Unclear	Unclear	Unclear	Low	High	Unclear	Low	Unclear	Unclear
Iatrou, 2005	Low	Unclear	Unclear	Low	High	Low	Unclear	Unclear	Unclear
Kabalak, 2005	Low	Unclear	Unclear	Unclear	Low	Unclear	Unclear	Unclear	Unclear
Khan, 2005	Unclear	Unclear	Unclear	Low	Unclear	High	Low	Unclear	Unclear
Kocamanoglu, 2005	Unclear	Unclear	Low	Low	Unclear	Low	Low	Unclear	Unclear
Kontrimaviciute, 2005	Unclear	Unclear	Unclear	Low	Unclear	Unclear	Unclear	Unclear	Unclear
Misra, 2005	Low	Unclear	Unclear	Low	Low	Low	Low	Unclear	Unclear
Pirat, 2005	Unclear	Unclear	Unclear	Low	Unclear	Low	Low	Unclear	Unclear
Treschan, 2005	Low	Low	Unclear	Low	Unclear	Low	Low	Unclear	Unclear
White, 2005[†]	Unclear	Unclear	Unclear	Low	Unclear	Unclear	Unclear	Unclear	Unclear
Alkaissi, 2004	Unclear	Low	Unclear	Low	Low	Unclear	Low	Unclear	Unclear
Apfel, 2004 (CR: Jokela, 2009)	Low	Low	Unclear	Unclear	Low	Low	Low	Unclear	Low
Barbone, 2004	Unclear	Unclear	Unclear	Unclear	Unclear	Low	Low	Unclear	Unclear
Barbosa, 2004	Unclear	Unclear	Unclear	Low	Unclear	Unclear	Low	Unclear	Unclear
Bhardwaj, 2004	Low	Low	Unclear	Low	Unclear	Low	Low	Unclear	Unclear
Binstock, 2004	Unclear	Unclear	Unclear	High	Low	Unclear	Low	Unclear	Unclear
Browning, 2004	Low	Unclear	Unclear	Unclear	Unclear	Low	Unclear	Unclear	Unclear
Celiker, 2004	Low	Low	Unclear	High	Unclear	Unclear	Low	Unclear	Unclear
Dua, 2004	Low	Unclear	Unclear	Low	Unclear	Low	Low	Unclear	Unclear
Eberhart, 2004	Unclear	Low	Unclear	Low	Low	Low	Low	Unclear	Unclear
Gan, 2004	Unclear	Unclear	Unclear	Low	Unclear	Unclear	Unclear	Unclear	Unclear
Han, 2004	Low	Low	Unclear	Unclear	Low	Unclear	Unclear	Unclear	Unclear
Hanaoka, 2004	Unclear	Low	Unclear	Low	High	Unclear	Unclear	Unclear	Unclear
Meyer, 2004	Low	Low	Unclear	Low	High	Low	Low	Unclear	Unclear
Panda, 2004	Low	Unclear	Low	Low	Unclear	Low	Low	Unclear	Unclear
Samakarndi, 2004	Unclear	Unclear	Low	Unclear	Low	Low	Low	Unclear	Unclear
Sinha, 2004	Low	Unclear	Unclear	Low	Low	Low	Low	Unclear	Unclear
Bharti, 2003	Unclear	Unclear	Unclear	Low	Unclear	Low	Low	Unclear	Unclear
Biswas, 2003a	Low	Low	Unclear	Unclear	Unclear	Low	Low	Unclear	Unclear
Biswas, 2003b	Low	Low	Unclear	Low	Unclear	Low	Low	Unclear	Unclear
Burmeister, 2003	Unclear	Unclear	Low	Low	Unclear	Unclear	Unclear	Unclear	Unclear
Charuluxananan, 2003	Low	Low	Unclear	Low	Low	Low	Low	Unclear	Unclear
Karamanlioglu, 2003	Low	Low	Unclear	Low	Low	Low	Low	Unclear	Unclear
Loewen, 2003	Low	Low	Unclear	Low	Low	Low	Low	Unclear	Unclear
Maddali, 2003	Low	Low	Unclear	Low	Unclear	Low	Low	Unclear	Unclear
Madenoglu, 2003	Unclear	Unclear	Unclear	Low	Low	Low	Low	Unclear	Unclear
O'Brien, 2003	Unclear	Unclear	Unclear	Unclear	Low	Unclear	Low	Unclear	Unclear

Olutoye, 2003	Low	Low	Unclear	Low	Unclear	Low	Unclear	Unclear	Unclear
Oztekin, 2003	Unclear	Low	Unclear	Low	Low	Low	Low	Unclear	Unclear
Paech, 2003	Low	Low	Unclear	Low	Low	Low	Low	Unclear	Unclear
Pan, 2003	Unclear	Unclear	Unclear	Low	Unclear	Unclear	Unclear	Unclear	Unclear
Samra, 2003†	Unclear	Unclear	Unclear	Low	Unclear	Low	Low	Unclear	Unclear
Szarvas, 2003	Low	Low	Unclear	Low	Low	Unclear	Low	Unclear	Unclear
Tang, 2003	Low	Low	Unclear	Low	Low	Low	Unclear	Unclear	Unclear
Thagaard, 2003	Low	Unclear	Unclear	High	Low	Unclear	Unclear	Unclear	Unclear
Tzeng, 2003	Low	Unclear	Unclear	Low	Unclear	Low	Low	Unclear	Unclear
Usmani, 2003	Unclear	Unclear	Unclear	Low	Unclear	Unclear	Unclear	Unclear	Unclear
Wagner, 2003	Unclear	Low	Unclear	Low	High	Unclear	Unclear	Unclear	Unclear
Wattwil, 2003	Low	Unclear	Unclear	Low	Low	Unclear	Unclear	Unclear	Unclear
Yuksek, 2003	Unclear	Low	Unclear	Low	Unclear	Low	Low	Unclear	Unclear
Adducci, 2002	Low	Unclear	Unclear	Low	Unclear	Low	Low	Unclear	Unclear
Agarwal, 2002	Low	Unclear	Unclear	Low	Unclear	Low	Low	Unclear	Unclear
Arcioni, 2002	Unclear	Unclear	Unclear	Low	Low	Low	Low	Unclear	Low
Argiriadou, 2002	Low	Low	Unclear	Low	Unclear	Low	Low	Unclear	Unclear
Coloma, 2002	Low	Low	Low	Low	Low	Low	Low	Unclear	Unclear
Coloma, 2002	Low	Low	Unclear	Low	Unclear	Low	Low	Unclear	Low
Elhakim, 2002	Low	Low	Unclear	Low	Unclear	Low	Low	Unclear	Unclear
Fabling, 2002	Unclear	Low	Unclear	Low	Low	Low	Low	Unclear	Unclear
Gan, 2002	Unclear	Unclear	Unclear	Low	Low	Unclear	Unclear	Unclear	Unclear
Goksu, 2002	Unclear	Unclear	Unclear	Low	Low	Low	Low	Unclear	Unclear
Grimsehl, 2002	Low	Low	Unclear	Low	Low	Low	Low	Unclear	Unclear
Jokela, 2002	Unclear	Unclear	Unclear	Low	High	Unclear	Low	Unclear	Unclear
Lee, 2002	Unclear	Unclear	Unclear	Low	Unclear	Low	Low	Unclear	Unclear
Marcus, 2002	Unclear	Unclear	Unclear	Unclear	Unclear	Unclear	Low	Unclear	Unclear
Mullick, 2002	Unclear	Unclear	Unclear	Low	Low	Unclear	Low	Unclear	Unclear
Munro, 2002	Low	Unclear	Unclear	High	High	Low	Low	Unclear	Unclear
Ozmen, 2002	Unclear	Unclear	Unclear	Low	Unclear	Unclear	Unclear	Unclear	Unclear
Paech, 2002	Unclear	Unclear	Low	Low	Low	Low	Low	Unclear	Unclear
Quaynor, 2002	Low	Low	Unclear	Low	Low	Low	Low	Unclear	Unclear
Sanchez-Ledesma, 2002	Unclear	Low	Unclear	Low	Unclear	Low	Low	Unclear	Unclear
Sennaraj, 2002	Low	Low	Unclear	Low	Unclear	Low	Low	Unclear	Unclear
So, 2002	Low	Unclear	Unclear	Low	Low	Low	Unclear	Unclear	Unclear
Sukhani, 2002	Unclear	Unclear	Unclear	Low	Low	Low	Unclear	Unclear	Unclear
Wang, 2002a	Low	Low	Unclear	Low	Low	Low	Low	Unclear	Unclear
Wang, 2002b	Low	Low	Unclear	Low	Low	Low	Low	Unclear	Unclear
Wang, 2002	Low	Unclear	Unclear	Low	Low	Low	Low	Unclear	Low
White, 2002	Low	Unclear	Low	Low	Unclear	Unclear	Low	Unclear	Unclear
Yazigi, 2002	Low	Unclear	Unclear	Low	Unclear	Low	Unclear	Unclear	Unclear
Bowhay, 2001	Low	Low	Unclear	Low	Unclear	Low	Low	Unclear	Unclear
Cherian, 2001	Low	Unclear	Unclear	Unclear	Unclear	Low	Low	Unclear	Unclear
Dabbous, 2001	Low	Low	Unclear	Low	High	Low	Low	Unclear	Unclear
De Witte, 2001	Unclear	Unclear	Unclear	Low	Unclear	Low	Low	Unclear	Unclear
Ganem, 2001	Low	Low	Unclear	Low	Low	Low	Low	Unclear	Unclear
Goll, 2001	Low	Unclear	Unclear	Low	Unclear	Low	Low	Unclear	Unclear
Kathirvel, 2001	Low	Unclear	Unclear	Low	Unclear	Low	Low	Unclear	Unclear
Millo, 2001	Low	Low	Unclear	Low	Low	Low	Low	Unclear	Unclear
Pan, 2001	Low	Low	Unclear	Low	Low	Low	Low	Unclear	High
Paventi, 2001	Unclear	Unclear	Unclear	Low	Unclear	Low	Low	Unclear	Unclear
Shende, 2001	Low	Unclear	Unclear	Low	Unclear	Low	Unclear	Low	Unclear
Somri, 2001	Low	Unclear	Unclear	Low	Unclear	Unclear	Unclear	Unclear	Unclear
Splinter, 2001	Unclear	Unclear	Unclear	Low	Unclear	Low	Unclear	Unclear	Unclear
Subramaniam, 2001	Low	Low	Unclear	Low	Low	Low	Low	Unclear	Unclear
Thomas, 2001	Low	Low	Unclear	Low	Unclear	Low	Low	Unclear	Low
White, 2001	Low	Low	Unclear	Low	Low	Unclear	Unclear	Unclear	Low
Wilson, 2001	Unclear	Low	Unclear	Low	Low	Unclear	Unclear	Unclear	Unclear
Yilmazlar, 2001	Unclear	Unclear	Unclear	Low	Unclear	Low	Unclear	Unclear	Unclear
Ahmed, 2000	Low	Unclear	Unclear	Low	Low	Low	Low	Unclear	Unclear
Charuluxananan, 2000	Low	Low	Unclear	Low	Unclear	Unclear	Low	Unclear	Unclear
Fabling, 2000	Unclear	Low	Unclear	Low	Unclear	Unclear	Low	Unclear	Unclear
Gesztesi, 2000	Low	Low	Low	Low	Low	Low	Low	Unclear	Low
Holt, 2000	Low	Low	Unclear	Low	Low	Low	Low	Unclear	Unclear

Jensen, 2000	Low	Unclear	Unclear	Unclear	Low	Low	Low	Unclear	Low
Jokela, 2000	Unclear	Unclear	Unclear	Low	Low	Low	Low	Unclear	Unclear
Kothari, 2000	Low	Unclear	Unclear	Low	High	Low	Low	Unclear	Unclear
Kreisler, 2000	Unclear	Unclear	Unclear	Low	High	Low	Low	Unclear	High
Ku, 2000	Unclear	Unclear	Unclear	Unclear	Low	Unclear	Low	Unclear	Unclear
Lee, 2000	Low	Unclear	Unclear	Unclear	High	Low	Low	Unclear	Unclear
Liberman, 2000	Low	Low	Unclear	Low	Unclear	Low	Unclear	Unclear	Unclear
Najeeb, 2000	Unclear	Unclear	Unclear	High	Unclear	Unclear	Unclear	Unclear	Unclear
Peixoto, 2000	Unclear	Low	Unclear	Low	Unclear	Unclear	Low	Unclear	Low
Philip, 2000	Unclear	Unclear	Unclear	Low	Low	Unclear	Low	Unclear	Low
Reihner, 2000	Unclear	Low	Unclear	Low	Low	Low	Low	Unclear	Unclear
Sadhasivam, 2000	Low	Low	Unclear	Low	Low	Low	Low	Unclear	Unclear
Scuderi, 2000	Unclear	Unclear	Low	Unclear	Unclear	High	Unclear	Unclear	Unclear
Sharma, 2000	Unclear	Unclear	Unclear	Low	Unclear	Low	Unclear	Unclear	Unclear
Wu, 2000	Low	Unclear	Unclear	Low	Low	Low	Unclear	Unclear	Unclear
Zarate, 2000	Low	Unclear	Unclear	Low	Low	Low	Unclear	Unclear	Unclear
Abouleish, 1999	Low	Unclear	Low	Unclear	Low	Low	Low	Unclear	High
Allen, 1999	Low	Unclear	Unclear	Low	Low	Low	Low	Unclear	Unclear
Barst, 1999	Low	Low	Unclear	Low	Low	Low	Unclear	Unclear	Unclear
Broome, 1999	Unclear	Unclear	Unclear	Low	High	Unclear	Unclear	Unclear	Unclear
Bugedo, 1999	Low	Low	Unclear	Low	Unclear	Unclear	Low	Unclear	Low
Cholwill, 1999	Unclear	Unclear	Unclear	Low	Low	Low	Low	Unclear	Unclear
de Adana, 1999	Low	Low	Unclear	Low	High	Low	Low	Unclear	Low
Gurler, 1999	Unclear	Unclear	Unclear	Unclear	Unclear	Unclear	Unclear	Unclear	Unclear
Helmy, 1999	Unclear	Unclear	Unclear	Low	Unclear	Low	Unclear	Unclear	Unclear
Janknegt, 1999	Low	Unclear	Low	Low	Unclear	Low	Unclear	Unclear	Unclear
Jokela, 1999	Unclear	Low	Unclear	Low	Unclear	Unclear	Low	Unclear	Unclear
Kathirvel, 1999	Unclear	Unclear	Unclear	Low	Unclear	Low	Low	Unclear	Unclear
Khalil, 1999	Low	Unclear	Unclear	Low	Unclear	Low	Low	Unclear	Unclear
Koivuranta, 1999	Unclear	Low	Unclear	Low	Low	Low	Low	Unclear	Unclear
Kovac, 1999	Low	Unclear	Unclear	Low	Low	Unclear	Unclear	Unclear	Unclear
Kyokong, 1999	Unclear	Low	Unclear	Unclear	Low	Low	Low	Unclear	Unclear
McCall, 1999	Unclear	Unclear	Unclear	Low	Unclear	Low	Low	Unclear	Unclear
Munro, 1999	Low	Low	Unclear	Low	Low	Unclear	Unclear	Unclear	Unclear
Rajeeva, 1999	Low	Unclear	Unclear	Low	Unclear	Unclear	Low	Unclear	Unclear
Sadhasivam, 1999	Unclear	Unclear	Unclear	Low	Low	Unclear	Low	Unclear	High
Sandhu, 1999	Unclear	Low	Unclear	Low	Unclear	Low	Unclear	Unclear	Unclear
Scuderi, 1999a	Unclear	Unclear	Low	Low	Low	Low	Low	Unclear	Unclear
Scuderi, 1999b	Unclear	Unclear	Unclear	Low	Low	Low	Low	Unclear	Unclear
Sinha, 1999	Low	Unclear	Unclear	Low	High	Low	Low	Unclear	Unclear
Swiatkowski, 1999	Unclear	Unclear	Unclear	Low	Unclear	Low	Low	Unclear	Unclear
Tsui, 1999	Unclear	Unclear	Unclear	Low	Unclear	Low	Unclear	Unclear	Low
Turhanoglu, 1999	Unclear	Unclear	Unclear	Low	Unclear	Unclear	Unclear	Unclear	Unclear
Wagley, 1999	Low	Unclear	Unclear	Low	Unclear	Unclear	Low	Unclear	Unclear
Woodward, 1999	Unclear	Low	Unclear	Low	High	Low	Low	Unclear	Unclear
Alon, 1998	Low	Low	Unclear	Low	Low	Unclear	Low	Unclear	Unclear
Ang, 1998	Low	Low	Unclear	Low	Low	Low	Unclear	Unclear	Unclear
Bacic, 1998	Unclear	Unclear	Unclear	Unclear	Low	Unclear	Low	Unclear	Unclear
Chan, 1998	Low	Unclear	Low	Low	Low	Low	Unclear	Unclear	Unclear
Chen, 1998a	Low	Low	Unclear	Low	Low	Low	Low	Unclear	Unclear
Chen, 1998b	Unclear	Unclear	Unclear	Low	Low	Low	Low	Unclear	Unclear
Dabbous, 1998	Low	Unclear	Unclear	Unclear	Unclear	Low	Low	Unclear	Unclear
Daftary, 1998	Unclear	Low	Unclear	Low	Unclear	Unclear	Low	Unclear	Unclear
Dershwitz, 1998	Low	Low	Unclear	Low	Unclear	Low	Low	Unclear	Unclear
Diemunsch, 1998	Unclear	Unclear	Unclear	Low	Low	Unclear	Low	Unclear	Unclear
Doe, 1998	Unclear	Unclear	Unclear	Low	Unclear	Low	Low	Unclear	Unclear
Dresner, 1998	Unclear	Unclear	Unclear	Low	Unclear	Low	Low	Unclear	Unclear
Goodarzi, 1998	Unclear	Unclear	Unclear	Low	Low	Low	Low	Unclear	Unclear
Hamid, 1998	Low	Low	Unclear	Low	Low	Low	Low	Unclear	Unclear
Jellish, 1998	Unclear	Low	Unclear	High	Unclear	Unclear	Low	Unclear	Unclear
Mitra, 1998	Unclear	Unclear	Unclear	Low	Unclear	Unclear	Low	Unclear	Unclear
Morris, 1998	Unclear	Unclear	Unclear	Low	Unclear	Unclear	Unclear	Unclear	Unclear
Scholz, 1998	Unclear	Low	Unclear	Low	Low	Unclear	Unclear	Unclear	Unclear
Splinter, 1998a	Low	Unclear	Unclear	Low	Low	Unclear	Unclear	Unclear	Unclear
Splinter, 1998b	Unclear	Low	Unclear	Low	Low	Low	Low	Unclear	Unclear
Tang, 1998a	Unclear	Low	Low	Low	Unclear	Unclear	Low	Unclear	Unclear

Tang, 1998b	Low	Low	Low	Unclear	Unclear	Low	Low	Unclear	Unclear
Tramer, 1998	Low	Unclear	Unclear	Low	Unclear	Low	Low	Unclear	Unclear
Akcabay, 1997	Unclear	Unclear	Unclear	Unclear	Unclear	Low	Unclear	Unclear	Unclear
Alexander, 1997	Low	Low	Unclear	Low	High	Low	Low	Unclear	Unclear
Ascaso, 1997	Unclear	Unclear	Unclear	Low	Unclear	Low	Low	Unclear	Unclear
Bach-Styles, 1997	Unclear	Unclear	Unclear	Low	Unclear	Low	Low	Unclear	Unclear
Carnahan, 1997	Unclear	Unclear	Unclear	Low	Unclear	Unclear	Low	Unclear	Low
Diemunsch, 1997a	Unclear	Unclear	Low	Low	Low	Low	Low	Unclear	High
Diemunsch, 1997b	Unclear	Low	Low	Low	Unclear	Unclear	Low	Unclear	Unclear
Diemunsch, 1997c	Unclear	Unclear	Low	Low	Unclear	Unclear	Low	Unclear	Unclear
Graczyk, 1997	Unclear	Low	Unclear	Low	Low	Low	Low	Unclear	Unclear
Klockgether-Radke, 1997	Unclear	Unclear	Unclear	Low	Unclear	Unclear	Low	Unclear	Unclear
Koivuranta, 1997	Low	Unclear	Unclear	High	Low	Low	Low	Unclear	Unclear
Korttila, 1997	Unclear	Unclear	Unclear	Low	Low	Unclear	Unclear	Unclear	Unclear
Kovac, 1997	Low	Low	Low	High	Low	Unclear	Low	Unclear	Unclear
Lawhorn, 1997	Low	Unclear	Unclear	Unclear	Unclear	Unclear	Unclear	Unclear	Unclear
Maestre, 1997	Unclear	Unclear	Unclear	Low	Unclear	Unclear	Unclear	Unclear	Unclear
McKenzie, 1997	Low	Low	Unclear	Low	Unclear	Unclear	Low	Unclear	Unclear
Mikawa, 1997	Unclear	Unclear	Low	Low	Unclear	Low	Low	Unclear	Unclear
Moens, 1997	Unclear	Unclear	Unclear	Low	Low	Unclear	Low	Unclear	Unclear
Monagle, 1997	Unclear	Low	Low	Low	Low	Low	Unclear	Unclear	Unclear
Morton, 1997	Unclear	Unclear	Unclear	Low	Low	Unclear	Low	Unclear	Unclear
Patel, 1997	Unclear	Unclear	Unclear	Low	Low	Low	Low	Unclear	Unclear
Pitkanen, 1997	Unclear	Unclear	Unclear	Low	Unclear	Unclear	Low	Unclear	Unclear
Polati, 1997	Unclear	Low	Low	Low	Low	Low	Low	Unclear	Unclear
Purhonen, 1997	Unclear	Low	Low	Low	Low	Low	Unclear	Unclear	Unclear
Rung, 1997	Low	Low	Low	Low	Unclear	Unclear	Low	Unclear	Unclear
Scuderi, 1997	Low	Unclear	Unclear	Low	Unclear	Unclear	Low	Unclear	Unclear
Shende, 1997	Unclear	Unclear	Unclear	Low	Unclear	Low	Low	Unclear	Unclear
Sniadach, 1997	Unclear	Low	Unclear	Low	Unclear	Low	Low	Unclear	Unclear
Splinter, 1997	Low	Unclear	Unclear	Low	Low	Low	Unclear	Unclear	Unclear
Stienstra, 1997	Low	Low	Low	Low	Unclear	Unclear	Low	Unclear	Unclear
Sun, 1997	Unclear	Low	Unclear	High	Unclear	Low	Unclear	Unclear	Unclear
Taylor, 1997	Low	Unclear	Low	Low	High	Unclear	Unclear	Unclear	Unclear
Ulusoy, 1997	Unclear	Unclear	Unclear	Unclear	Unclear	Unclear	Low	Unclear	Unclear
Warriner, 1997	Unclear	Unclear	Unclear	Low	Low	Unclear	Low	Unclear	Unclear
Ali-melkkila, 1996	Unclear	Unclear	Unclear	Low	Low	Unclear	Low	Unclear	Unclear
Alon, 1996	Unclear	Unclear	Unclear	Low	Low	Unclear	Low	Unclear	Unclear
Capouet, 1996	Unclear	Low	Unclear	Low	Unclear	Unclear	Low	Unclear	Unclear
Chen, 1996	Unclear	Unclear	Unclear	Unclear	Low	Low	Low	Unclear	Unclear
Cieslak, 1996	Low	Low	Unclear	Low	Unclear	Low	Low	Unclear	Unclear
Davies, 1996	Low	Unclear	Unclear	Low	Low	Low	Low	Unclear	Unclear
Ercelen, 1996	Unclear	Low	Unclear	Low	Unclear	Low	Low	Unclear	Unclear
Eriksson, 1996	Unclear	Unclear	Unclear	Low	Low	Low	Unclear	Unclear	Unclear
Furst, 1996	Unclear	Low	Unclear	Low	Low	Low	Low	Unclear	Unclear
Gaynes, 1996[†]	Unclear	Unclear	Unclear	Unclear	Unclear	Unclear	Unclear	Unclear	Unclear
Honkavaara, 1996	Unclear	Unclear	Unclear	Low	Unclear	Low	Unclear	Unclear	Unclear
Kaul, 1996	Unclear	Low	Unclear	Low	Unclear	Unclear	Low	Unclear	Unclear
Khalil, 1996	Low	Unclear	Low	Low	Low	Unclear	Low	Unclear	Unclear
Kimya, 1996	Unclear	Unclear	Unclear	Low	Unclear	Unclear	Low	Unclear	Unclear
Koivuranta, 1996	Low	Low	Low	High	Unclear	Low	Low	Unclear	Unclear
Kovac, 1996 (CR: Pearman, 1994c)	Unclear	Unclear	Unclear	Low	Unclear	Unclear	Low	Unclear	Unclear
Kumar, 1996	Unclear	Unclear	Unclear	Low	Unclear	Low	Low	Unclear	Unclear
Lawhorn, 1996	Unclear	Unclear	Unclear	Unclear	Unclear	Unclear	Unclear	Unclear	Unclear
Lekprasert, 1996	Unclear	Unclear	Unclear	Low	Unclear	Unclear	Unclear	Unclear	Unclear
Lopez-Orlaondo, 1996	Unclear	Unclear	Unclear	Low	Unclear	Low	Unclear	Unclear	Unclear
Naguib, 1996	Unclear	Low	Unclear	Low	Unclear	Low	Low	Unclear	Unclear
Pan, 1996	Low	Unclear	Unclear	Low	Unclear	Low	Unclear	Unclear	Unclear
Principi, 1996	Unclear	Unclear	Unclear	Low	Low	Unclear	Low	Unclear	Unclear
Pueyo, 1996	Unclear	Low	Unclear	Low	Low	Low	Low	Unclear	Unclear
Pugh, 1996	Unclear	Unclear	Unclear	Low	Unclear	Unclear	Low	Unclear	Unclear
Rodrigo, 1996	Low	Unclear	Unclear	Low	Low	Low	Low	Unclear	Unclear
Rose, 1996a	Unclear	Low	Unclear	Low	Low	Low	Low	Unclear	Unclear

Rose, 1996b	Low	Low	Unclear	Low	Unclear	Low	Low	Unclear	Unclear
Stene, 1996	Low	Low	Unclear	Low	Unclear	Low	Low	Unclear	Unclear
Tang, 1996	Low	Low	Unclear	Low	High	Low	Unclear	Unclear	Unclear
Usha, 1996	Unclear	Unclear	Unclear	Unclear	Unclear	Unclear	Unclear	Unclear	Unclear
Van Den Berg, 1996a	Unclear	Unclear	Unclear	Low	Low	Low	Low	Unclear	Unclear
Van Den Berg, 1996b	Unclear	Unclear	Unclear	Low	High	Unclear	Low	Unclear	Unclear
Van Den Berg, 1996c	Unclear	Unclear	Unclear	Low	Low	Low	Low	Unclear	Unclear
Watts, 1996	Unclear	Low	Unclear	Low	Unclear	Low	Low	Unclear	Unclear
Wilson, 1996	Low	Low	Unclear	Low	Unclear	Unclear	Low	Unclear	Low
Wrench, 1996	Unclear	Unclear	Unclear	Low	Unclear	Low	Low	Unclear	Unclear
Yilmazlar, 1996	Unclear	Unclear	Unclear	Low	Unclear	Unclear	Low	Unclear	Unclear
Alexander, 1995	Unclear	Unclear	Unclear	Low	Unclear	Low	Low	Unclear	Unclear
Campbell, 1995	Unclear	Unclear	Low	Low	Unclear	Unclear	Low	Unclear	Unclear
Davis, 1995a	Unclear	Unclear	Unclear	Low	Unclear	Low	Low	Unclear	Unclear
Davis, 1995b	Low	Low	Unclear	Low	Low	Low	Low	Unclear	Unclear
Desilva, 1995	Unclear	Low	Low	Low	High	Low	Low	Unclear	Unclear
Elhakim, 1995	Unclear	Low	Unclear	Low	Unclear	Low	Low	Unclear	Unclear
Grond, 1995	Low	Low	Unclear	Low	Low	Low	Low	Unclear	Unclear
Litman, 1995	Low	Unclear	Unclear	Low	Unclear	Unclear	Unclear	Unclear	Unclear
Mikawa, 1995	Unclear	Unclear	Low	Low	Unclear	Low	Low	Unclear	Unclear
Paech, 1995	Low	Unclear	Unclear	Low	Low	Unclear	Low	Unclear	Unclear
Paxton, 1995a	Unclear	Unclear	Unclear	Low	Unclear	Unclear	Low	Unclear	Low
Paxton, 1995b	Unclear	Unclear	Unclear	Low	Unclear	Unclear	Low	Unclear	Low
Splinter, 1995a	Unclear	Unclear	Unclear	Low	Unclear	Unclear	Unclear	Unclear	Unclear
Splinter, 1995b	Unclear	Unclear	Unclear	Unclear	Unclear	Low	Low	Unclear	Unclear
Watcha, 1995	Low	Low	Unclear	Low	Unclear	Unclear	Low	Unclear	Unclear
Furst, 1994	Unclear	Low	Unclear	Low	Unclear	Low	Low	Unclear	Unclear
Gan, 1994	Unclear	Unclear	Unclear	Low	Low	Unclear	Low	Unclear	Unclear
Kaufmann, 1994	Low	Low	Unclear	High	Low	Low	Low	Unclear	Unclear
Khalil, 1994 (CR: Pearman, 1994b)	Unclear	Unclear	Unclear	Low	Unclear	Low	Unclear	Unclear	High
Litman, 1994	Low	Low	Unclear	Low	Unclear	Low	Unclear	Unclear	Unclear
Malins, 1994	Unclear	Low	Unclear	Low	Low	Low	Low	Unclear	Unclear
McKenzie, 1994	Unclear	Unclear	Unclear	Low	Unclear	Unclear	Low	Unclear	Unclear
Rodrigo, 1994	Low	Unclear	Unclear	Low	Low	Low	Low	Unclear	Unclear
Rose, 1994	Low	Unclear	Unclear	Low	Low	Low	Low	Unclear	Unclear
Suen, 1994	Unclear	Unclear	Unclear	Low	Low	Low	Low	Unclear	Unclear
Ummenhofer, 1994	Low	Low	Unclear	Low	Unclear	Low	Low	Unclear	Unclear
Volpe, 1994	Unclear	Unclear	Unclear	Low	Unclear	Low	Low	Unclear	Unclear
Dupeyron, 1993	Unclear	Unclear	Unclear	Low	Unclear	Unclear	Low	Unclear	Unclear
Helmers, 1993	Unclear	Unclear	Unclear	Low	Low	Unclear	Unclear	Unclear	Unclear
McKenzie, 1993b (CR: Dershwitz, 1992)	Unclear	Unclear	Unclear	Low	High	Unclear	Unclear	Unclear	High
Raphael, 1993	Unclear	Unclear	Low	Low	Low	Unclear	Unclear	Unclear	Unclear
Sung, 1993	Low	Low	Unclear	High	Low	Low	Unclear	Unclear	Unclear
Zomers, 1993	Unclear	Unclear	Unclear	Low	Low	Unclear	Low	Unclear	Unclear
Alon, 1992	Unclear	Unclear	Low	Low	Low	Low	Low	Unclear	Unclear
Du Pen, 1992 (CR: Scuderi, 1993)	Unclear	Unclear	Low	High	Unclear	Unclear	Low	Unclear	Unclear
Kenny, 1992	Unclear	Unclear	Unclear	Low	Low	Low	Low	Unclear	Unclear
Kovac, 1992 (CRs: McKenzie, 1993a; Pearman, 1994a)	Unclear	Unclear	Low	Low	Low	Unclear	Low	Unclear	Unclear
Bodner, 1991	Low	Unclear	Unclear	High	Unclear	Unclear	Low	Unclear	High
Larijani, 1991	Low	Unclear	Unclear	Low	Unclear	Unclear	Low	Unclear	Unclear
Leeser, 1991	Low	Unclear	Unclear	Low	Unclear	Low	Low	Unclear	Unclear
Non-randomized clinical trials (n=5)									
Stamer, 2011	High	High	Unclear	Low	Low	High	Unclear	Unclear	High
Rabbani, 2010	High	High	Unclear	Unclear	Unclear	Unclear	Unclear	Unclear	Unclear
Kim, 2007	High	High	Unclear	Low	Unclear	Unclear	Unclear	Unclear	Unclear
Biedler, 2004	High	High	Unclear	High	Low	Unclear	Low	Unclear	High
Manani,1996	High	High	Unclear	Low	Unclear	Unclear	Unclear	Unclear	Unclear

Controlled before-after studies (n=1)									
Wagner, 2007	High	High	Unclear	High	High	High	Unclear	Unclear	High

Note: †Unpublished data.
Abbreviations: CR, companion report; EPOC, Effective Practice and Organization of Care; High, high risk; Low, low risk; Unclear, unclear risk .
Items:
1. Random sequence generation
2. Allocation concealment
3. Similar baseline outcome measures
4. Similar baseline characteristics
5. Incomplete outcome data
6. Blinding of outcome assessor
7. Contamination
8. Selective outcome reporting
9. Other bias

Appendix 8. Aggregate EPOC risk-of-bias appraisal results

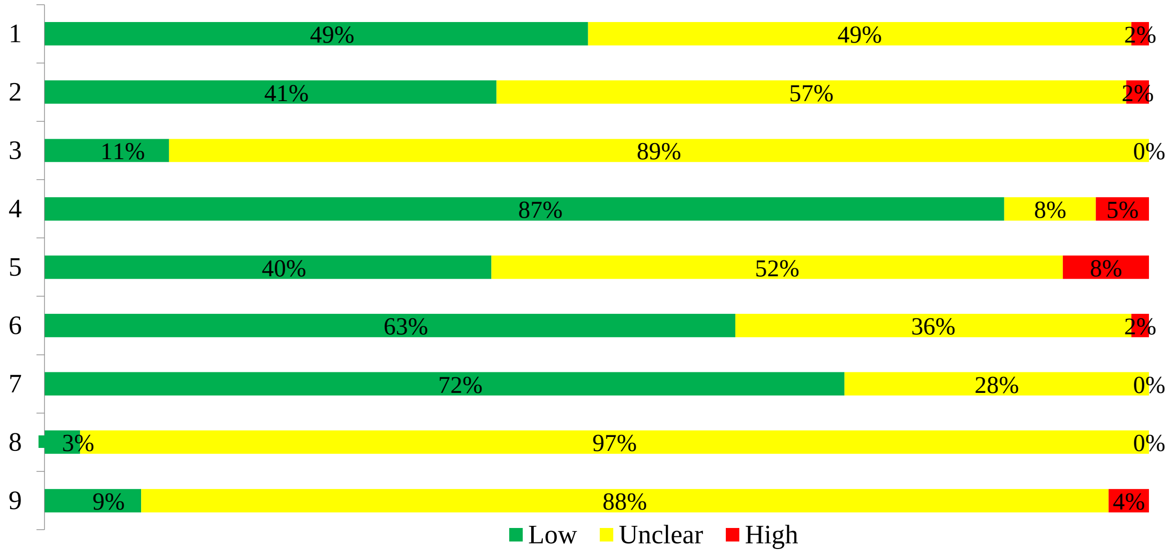

Abbreviations: EPOC, Effective Practice and Organization of Care; High, High risk of bias; Low, Low risk of bias; Unclear, Unclear risk of bias.

Items:

1. Random sequence generation
2. Allocation concealment
3. Similar baseline outcome measures
4. Similar baseline characteristics
5. Incomplete outcome data
6. Blinding
7. Contamination
8. Selective outcome reporting
9. Other bias

Appendix 9. Newcastle-Ottawa Scale appraisal results

**Author, year**	**1**	**2**	**3**	**4**	**5**	**6**	**7**
Shakeel, 2012	B	A	A	B	A	B	D
Chen, 2011	B	A	A	B	A	B	D
Eryilmaz, 2008	B	A	D	B	C	C	D
Habib, 2007	B	A	A	B	C	B	D
Flynn, 2006	B	A	A	B	C	B	D
Piwko, 2005	B	A	A	B	C	B	D
Wang, 2005	A	A	A	B	A	D	D
Walker, 2001	B	A	A	B	C	D	D
O'Brien, 2000	B	A	D	B	C	D	D

Items:

1. Representativeness of the exposed cohort: A, truly representative of the average participant undergoing surgery; B, somewhat representative of the average undergoing surgery.
2. Selection of the non-exposed cohort: A, drawn from the same community as the exposed cohort.
3. Ascertainment of exposure: A, secure records (e.g. medical records); D, no description.
4. Demonstration that outcome of interest was not present at start of study: B, no.
5. Comparability of cohorts on the basis of the design or analysis: A, study controls for most important factors (e.g., age, gender); C, nothing is controlled for.
6. Assessment of outcome: B, record linkage/questionnaire; C, self-report; D, no description.
7. Adequacy of follow up of cohorts: D, no statement.

Appendix 10. Comparison adjusted funnel plots

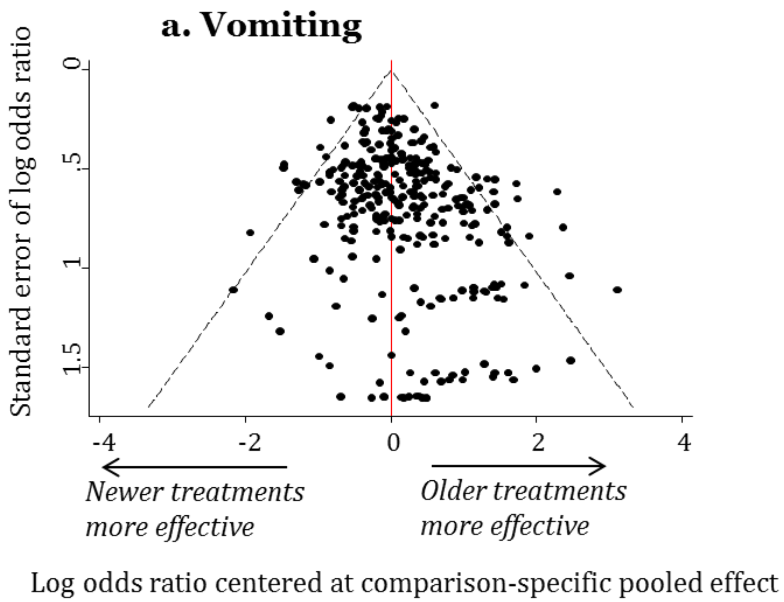

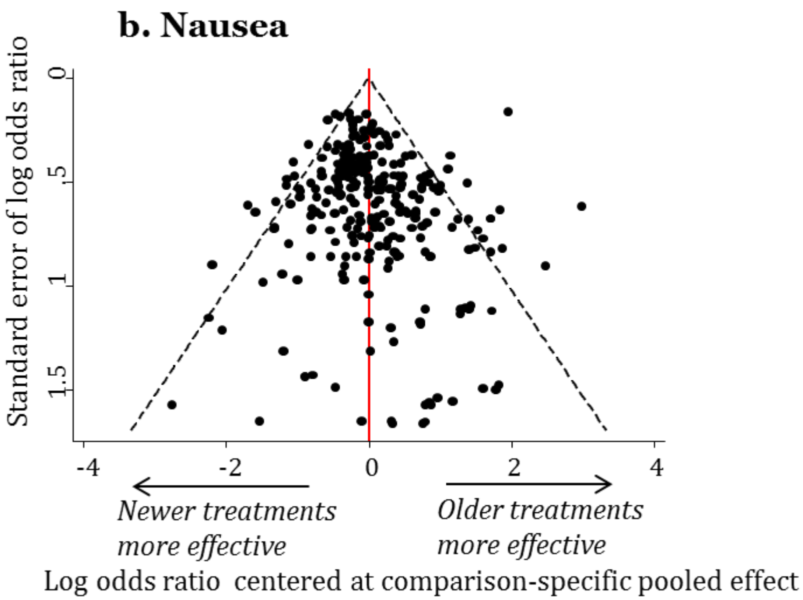

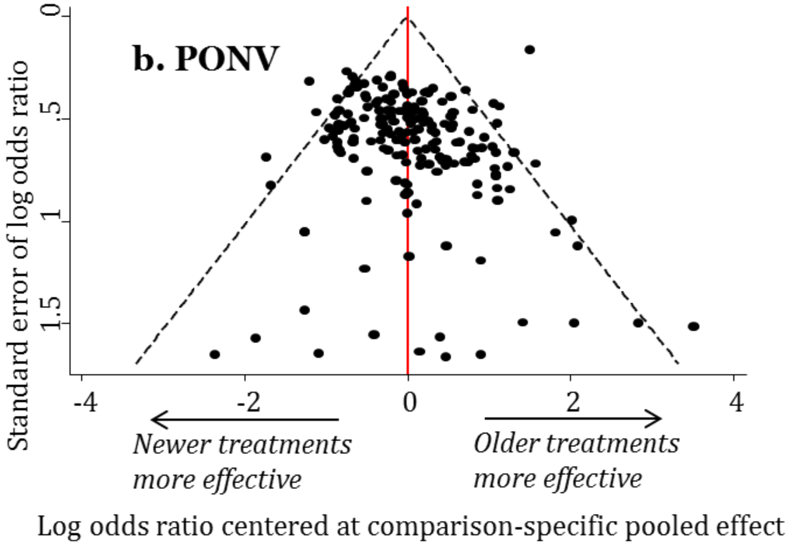

Appendix 11: Network meta-analysis results for patients of all ages and children only for all time periods

Treatment Comparison	*# of studies*	*MA estimate: OR (95% CI)*	*Heterogeneity variance*	*NMA estimate: OR (95% CI)*	*# of studies*	*MA estimate: OR (95% CI)*	*Heterogeneity variance*	*NMA estimate: OR (95% CI)*
	**Vomiting (all ages) - 238 RCTs and 12,781 patients**				**Vomiting (children) - 46 RCTs and 1,830 patients**			
Ondansetron vs Placebo	146	0.35 (0.32–0.39)	0.093	0.36 (0.33–0.40)	34	0.30 (0.24–0.38)	0.181	0.30 (0.24–0.38)
Granisetron vs Placebo	27	0.24 (0.16–0.34)	0.446	0.26 (0.21–0.34)	4	0.21 (0.08–0.56)	0.637	0.23 (0.12–0.48)
Dolasetron vs Placebo	7	0.42 (0.21–0.83)	0.581	0.44 (0.30–0.63)	3	0.41 (0.23–0.75)	0.000	0.39 (0.19–0.78)
Tropisetron vs Placebo	15	0.32 (0.22–0.48)	0.248	0.32 (0.23–0.43)	3	0.18 (0.09–0.36)	0.000	0.18 (0.08–0.41)
Ondansetron+DEX vs Placebo	12	0.16 (0.09–0.27)	0.150	0.16 (0.12–0.23)	5	0.06 (0.03–0.17)	0.000	0.07 (0.03–0.15)
Palonosetron vs Placebo	4	0.53 (0.38–0.73)	0.000	0.38 (0.24–0.60)	NA	NA	NA	NA
Ramosetron vs Placebo	5	0.42 (0.26–0.68)	0.000	0.28 (0.18–0.43)	NA	NA	NA	NA
Ondansetron+DROP vs Placebo	2	0.15 (0.07–0.31)	0.000	0.14 (0.08–0.26)	1	0.13 (0.05–0.33)	--	0.11 (0.04–0.33)
Ondansetron+METO vs Placebo	2	0.16 (0.06–0.43)	0.000	0.15 (0.06–0.42)	2	0.16 (0.06–0.43)	0.000	0.18 (0.06–0.53)
Granisetron+DEX vs Placebo	5	0.16 (0.08–0.31)	0.000	0.15 (0.09–0.24)	2	0.08 (0.03–0.27)	0.000	0.09 (0.02–0.31)
Palonosetron+DEX vs Placebo	NA	NA	NA	1.43 (0.2–10.14)	NA	NA	NA	NA
Dolasetron+DEX vs Placebo	1	0.06 (0.01–0.30)	--	0.18 (0.06–0.49)	NA	NA	NA	NA
Dolasetron+DROP vs Placebo	1	0.16 (0.07–0.35)	--	0.19 (0.07–0.52)	NA	NA	NA	NA
Granisetron+DROP vs Placebo	2	0.30 (0.05–1.66)	0.864	0.31 (0.11–0.82)	NA	NA	NA	NA
Granisetron vs Ondansetron	12	0.52 (0.34–0.81)	0.000	0.73 (0.56–0.94)	NA	NA	NA	0.78 (0.37–1.63)
Dolasetron vs Ondansetron	6	1.14 (0.72–1.80)	0.000	1.21 (0.83–1.75)	2	1.03 (0.39–2.77)	0.169	1.28 (0.63–2.62)
Tropisetron vs Ondansetron	3	0.72 (0.29–1.78)	0.348	0.87 (0.63–1.21)	NA	NA	NA	0.58 (0.24–1.39)
Ondansetron+DEX vs Ondansetron	15	0.50 (0.33–0.75)	0.170	0.46 (0.33–0.63)	3	0.24 (0.12–0.47)	0.000	0.23 (0.11–0.49)
Palonosetron vs Ondansetron	3	0.20 (0.02–2.16)	3.553	1.06 (0.67–1.68)	NA	NA	NA	NA
Ramosetron vs Ondansetron	6	0.57 (0.38–0.85)	0.000	0.78 (0.51–1.18)	NA	NA	NA	NA
Ondansetron+DROP vs Ondansetron	5	0.43 (0.24–0.78)	0.000	0.39 (0.21–0.71)	1	0.31 (0.12–0.77)	--	0.37 (0.13–1.09)
Ondansetron+METO vs Ondansetron	3	0.53 (0.18–1.58)	0.030	0.42 (0.15–1.17)	2	0.73 (0.23–2.25)	0.000	0.59 (0.19–1.78)
Granisetron+DEX vs Ondansetron	NA	NA	NA	0.41 (0.25–0.67)	NA	NA	NA	0.28 (0.08–1.04)
Palonosetron+DEX vs Ondansetron	NA	NA	NA	3.96 (0.56–28.12)	NA	NA	NA	NA
Dolasetron+DEX vs Ondansetron	NA	NA	NA	0.50 (0.18–1.37)	NA	NA	NA	NA
Dolasetron+DROP vs Ondansetron	NA	NA	NA	0.54 (0.20–1.44)	NA	NA	NA	NA
Granisetron+DROP vs Ondansetron	NA	NA	NA	0.85 (0.31–2.28)	NA	NA	NA	NA
Dolasetron vs Granisetron	NA	NA	NA	1.66 (1.07–2.57)	NA	NA	NA	1.65 (0.61–4.47)
Tropisetron vs Granisetron	NA	NA	NA	1.20 (0.80–1.79)	NA	NA	NA	0.75 (0.25–2.25)
Ondansetron+DEX vs Granisetron	NA	NA	NA	0.63 (0.42–0.94)	NA	NA	NA	0.30 (0.11–0.83)
Palonosetron vs Granisetron	2	0.20 (0.02–2.25)	2.058	1.46 (0.88–2.42)	NA	NA	NA	NA
Ramosetron vs Granisetron	1	2.20 (0.51–9.58)	--	1.07 (0.66–1.73)	NA	NA	NA	NA

Ondansetron+DROP vs Granisetron	NA	NA	NA	0.54 (0.28–1.03)	NA	NA	NA	0.48 (0.13–1.73)
Ondansetron+METO vs Granisetron	NA	NA	NA	0.58 (0.20–1.64)	NA	NA	NA	0.76 (0.21–2.81)
Granisetron+DEX vs Granisetron	7	0.39 (0.2–0.77)	0.176	0.57 (0.35–0.92)	1	0.14 (0.02, 1.23)	--	0.36 (0.09–1.50)
Palonosetron+DEX vs Granisetron	NA	NA	NA	5.44 (0.76–39.01)	NA	NA	NA	NA
Dolasetron+DEX vs Granisetron	NA	NA	NA	0.68 (0.24–1.92)	NA	NA	NA	NA
Dolasetron+DROP vs Granisetron	NA	NA	NA	0.74 (0.27–2.03)	NA	NA	NA	NA
Granisetron+DROP vs Granisetron	2	1.00 (0.32–3.17)	0.000	1.16 (0.43–3.12)	NA	NA	NA	NA
Tropisetron vs Dolasetron	1	0.51 (0.20–1.28)	--	0.72 (0.45–1.16)	NA	NA	NA	0.46 (0.15–1.35)
Ondansetron+DEX vs Dolasetron	NA	NA	NA	0.38 (0.23–0.62)	NA	NA	NA	0.18 (0.07–0.50)
Palonosetron vs Dolasetron	NA	NA	NA	0.88 (0.49–1.58)	NA	NA	NA	NA
Ramosetron vs Dolasetron	NA	NA	NA	0.65 (0.37–1.12)	NA	NA	NA	NA
Ondansetron+DROP vs Dolasetron	NA	NA	NA	0.32 (0.16–0.65)	NA	NA	NA	0.29 (0.08–1.04)
Ondansetron+METO vs Dolasetron	NA	NA	NA	0.35 (0.12–1.03)	NA	NA	NA	0.46 (0.13–1.68)
Granisetron+DEX vs Dolasetron	NA	NA	NA	0.34 (0.19–0.63)	NA	NA	NA	0.22 (0.05–0.95)
Palonosetron+DEX vs Dolasetron	NA	NA	NA	3.28 (0.45–24.07)	NA	NA	NA	NA
Dolasetron+DEX vs Dolasetron	2	0.30 (0.04–2.43)	1.795	0.41 (0.16–1.08)	NA	NA	NA	NA
Dolasetron+DROP vs Dolasetron	1	0.57 (0.24–1.35)	--	0.45 (0.17–1.20)	NA	NA	NA	NA
Granisetron+DROP vs Dolasetron	NA	NA	NA	0.70 (0.25–2.01)	NA	NA	NA	NA
Ondansetron+DEX vs Tropisetron	NA	NA	NA	0.52 (0.33–0.82)	NA	NA	NA	0.40 (0.13–1.22)
Palonosetron vs Tropisetron	NA	NA	NA	1.21 (0.70–2.10)	NA	NA	NA	NA
Ramosetron vs Tropisetron	NA	NA	NA	0.89 (0.53–1.51)	NA	NA	NA	NA
Ondansetron+DROP vs Tropisetron	NA	NA	NA	0.45 (0.23–0.88)	NA	NA	NA	0.64 (0.16–2.48)
Ondansetron+METO vs Tropisetron	NA	NA	NA	0.48 (0.17–1.40)	NA	NA	NA	1.01 (0.25–4.02)
Granisetron+DEX vs Tropisetron	NA	NA	NA	0.47 (0.26–0.84)	NA	NA	NA	0.48 (0.10–2.25)
Palonosetron+DEX vs Tropisetron	NA	NA	NA	4.53 (0.62–32.90)	NA	NA	NA	NA
Dolasetron+DEX vs Tropisetron	NA	NA	NA	0.57 (0.20–1.62)	NA	NA	NA	NA
Dolasetron+DROP vs Tropisetron	NA	NA	NA	0.61 (0.22–1.72)	NA	NA	NA	NA
Granisetron+DROP vs Tropisetron	NA	NA	NA	0.97 (0.34–2.72)	NA	NA	NA	NA
Palonosetron vs Ondansetron+DEX	NA	NA	NA	2.32 (1.33–4.07)	NA	NA	NA	NA
Ramosetron vs Ondansetron+DEX	NA	NA	NA	1.71 (1.01–2.90)	NA	NA	NA	NA
Ondansetron+DROP vs Ondansetron+DEX	3	0.55 (0.20–1.50)	0.044	0.86 (0.45–1.63)	NA	NA	NA	1.60 (0.44–5.83)
Ondansetron+METO vs Ondansetron+DEX	NA	NA	NA	0.93 (0.32–2.68)	NA	NA	NA	2.53 (0.68–9.47)
Granisetron+DEX vs Ondansetron+DEX	3	1.54 (0.72–3.27)	0.000	0.90 (0.53–1.55)	1	3.27 (0.32–33.78)	--	1.22 (0.29–5.15)
Palonosetron+DEX vs Ondansetron+DEX	NA	NA	NA	8.68 (1.19–63.20)	NA	NA	NA	NA
Dolasetron+DEX vs Ondansetron+DEX	NA	NA	NA	1.08 (0.37–3.14)	NA	NA	NA	NA
Dolasetron+DROP vs Ondansetron+DEX	NA	NA	NA	1.18 (0.42–3.32)	NA	NA	NA	NA

Granisetron+DROP vs Ondansetron+DEX	NA	NA	NA	1.85 (0.66–5.21)	NA	NA	NA	NA
Ramosetron vs Palonosetron	NA	NA	NA	0.74 (0.40–1.36)	NA	NA	NA	NA
Ondansetron+DROP vs Palonosetron	NA	NA	NA	0.37 (0.17–0.78)	NA	NA	NA	NA
Ondansetron+METO vs Palonosetron	NA	NA	NA	0.40 (0.13–1.21)	NA	NA	NA	NA
Granisetron+DEX vs Palonosetron	NA	NA	NA	0.39 (0.20–0.75)	NA	NA	NA	NA
Palonosetron+DEX vs Palonosetron	2	3.75 (0.6–23.42)	0.000	3.73 (0.56–25.08)	NA	NA	NA	NA
Dolasetron+DEX vs Palonosetron	NA	NA	NA	0.47 (0.15–1.41)	NA	NA	NA	NA
Dolasetron+DROP vs Palonosetron	NA	NA	NA	0.51 (0.17–1.50)	NA	NA	NA	NA
Granisetron+DROP vs Palonosetron	NA	NA	NA	0.80 (0.27–2.36)	NA	NA	NA	NA
Ondansetron+DROP vs Ramosetron	NA	NA	NA	0.50 (0.24–1.04)	NA	NA	NA	NA
Ondansetron+METO vs Ramosetron	NA	NA	NA	0.54 (0.18–1.62)	NA	NA	NA	NA
Granisetron+DEX vs Ramosetron	NA	NA	NA	0.53 (0.28–1.00)	NA	NA	NA	NA
Palonosetron+DEX vs Ramosetron	NA	NA	NA	5.08 (0.69–37.64)	NA	NA	NA	NA
Dolasetron+DEX vs Ramosetron	NA	NA	NA	0.63 (0.21–1.90)	NA	NA	NA	NA
Dolasetron+DROP vs Ramosetron	NA	NA	NA	0.69 (0.24–2.00)	NA	NA	NA	NA
Granisetron+DROP vs Ramosetron	NA	NA	NA	1.09 (0.37–3.16)	NA	NA	NA	NA
Ondansetron+METO vs Ondansetron+DROP	NA	NA	NA	1.08 (0.33–3.51)	NA	NA	NA	1.58 (0.34–7.29)
Granisetron+DEX vs Ondansetron+DROP	NA	NA	NA	1.06 (0.49–2.27)	NA	NA	NA	0.76 (0.14–4.04)
Palonosetron+DEX vs Ondansetron+DROP	NA	NA	NA	10.13 (1.31–78.58)	NA	NA	NA	NA
Dolasetron+DEX vs Ondansetron+DROP	NA	NA	NA	1.27 (0.39–4.11)	NA	NA	NA	NA
Dolasetron+DROP vs Ondansetron+DROP	NA	NA	NA	1.37 (0.43–4.34)	NA	NA	NA	NA
Granisetron+DROP vs Ondansetron+DROP	NA	NA	NA	2.16 (0.68–6.86)	NA	NA	NA	NA
Granisetron+DEX vs Ondansetron+METO	NA	NA	NA	0.98 (0.32–3.01)	NA	NA	NA	0.48 (0.09–2.61)
Palonosetron+DEX vs Ondansetron+METO	NA	NA	NA	9.38 (1.03–85.06)	NA	NA	NA	NA
Dolasetron+DEX vs Ondansetron+METO	NA	NA	NA	1.17 (0.28–4.91)	NA	NA	NA	NA
Dolasetron+DROP vs Ondansetron+METO	NA	NA	NA	1.27 (0.31–5.22)	NA	NA	NA	NA
Granisetron+DROP vs Ondansetron+METO	NA	NA	NA	2.00 (0.49–8.24)	NA	NA	NA	NA
Palonosetron+DEX vs Granisetron+DEX	NA	NA	NA	9.6 (1.28–72.03)	NA	NA	NA	NA
Dolasetron+DEX vs Granisetron+DEX	NA	NA	NA	1.20 (0.39–3.68)	NA	NA	NA	NA
Dolasetron+DROP vs Granisetron+DEX	NA	NA	NA	1.30 (0.44–3.90)	NA	NA	NA	NA
Granisetron+DROP vs Granisetron+DEX	2	2.38 (0.61–9.23)	0.000	2.05 (0.72–5.87)	NA	NA	NA	NA
Dolasetron+DEX vs Palonosetron+DEX	NA	NA	NA	0.12 (0.01–1.13)	NA	NA	NA	NA
Dolasetron+DROP vs Palonosetron+DEX	NA	NA	NA	0.14 (0.02–1.21)	NA	NA	NA	NA
Granisetron+DROP vs Palonosetron+DEX	NA	NA	NA	0.21 (0.02–1.91)	NA	NA	NA	NA
Dolasetron+DROP vs Dolasetron+DEX	NA	NA	NA	1.09 (0.27–4.29)	NA	NA	NA	NA

Granisetron+DROP vs Dolasetron+DEX	NA	NA	NA	1.71 (0.42–7.02)	NA	NA	NA	NA
Granisetron+DROP vs Dolasetron+DROP	NA	NA	NA	1.57 (0.39–6.33)	NA	NA	NA	NA
**Between-study heterogeneity**	**0.129**				**0.154**			
**Design-by-treatment interaction model for inconsistency (χ^2, d.f., P-value, heterogeneity)**	**50.77 (44, 0.224, 0.124)**				**2.98 (10, 0.982, 0.229)**			
	**Nausea (all ages) - 195 RCTs and 24,230 patients**				**Nausea (children) - 11 RCTs and 1,326 patients**			
Ondansetron vs Placebo	121	0.46 (0.41–0.52)	0.294	0.46 (0.41–0.52)	10	0.44 (0.29–0.68)	0.143	0.45 (0.30–0.66)
Granisetron vs Placebo	21	0.35 (0.23–0.52)	0.393	0.35 (0.26–0.47)	NA	NA	NA	NA
Dolasetron vs Placebo	9	0.59 (0.48–0.73)	0.000	0.60 (0.43–0.86)	1	0.29 (0.11–0.73)	--	0.26 (0.09–0.74)
Tropisetron vs Placebo	15	0.51 (0.40–0.66)	0.056	0.48 (0.35–0.65)	NA	NA	NA	NA
Ondansetron+DEX vs Placebo	8	0.21 (0.14–0.34)	<0.0001	0.28 (0.19–0.41)	2	0.21 (0.08–0.57)	0.00	0.22 (0.08–0.61)
Palonosetron vs Placebo	3	0.48 (0.33–0.68)	0.000	0.30 (0.17–0.53)	NA	NA	NA	NA
Ramosetron vs Placebo	5	0.35 (0.18–0.68)	0.393	0.32 (0.22–0 .47)	NA	NA	NA	NA
Ondansetron+DROP vs Placebo	2	0.31 (0.06–1.55)	0.730	0.26 (0.14–0.49)	1	0.07 (0.00–1.26)	--	0.07 (0.00–1.38)
Ondansetron+METO vs Placebo	1	1.00 (0.13–7.72)	--	0.30 (0.07–1.37)	1	1.00 (0.13–7.72)	--	1.18 (0.19–7.52)
Granisetron+DEX vs Placebo	4	0.21 (0.11–0.39)	0.000	0.20 (0.12–0.34)	1	0.09 (0.02–0.49)	--	0.10 (0.02–0.53)
Palonosetron+DEX vs Placebo	NA	NA	NA	0.37 (0.12–1.10)	NA	NA	NA	NA
Dolasetron+DEX vs Placebo	1	0.28 (0.05–1.53)	--	0.21 (0.07–0.61)	NA	NA	NA	NA
Dolasetron+DROP vs Placebo	1	0.17 (0.08–0.36)	--	0.19 (0.07–0.54)	NA	NA	NA	NA
Granisetron+DROP vs Placebo	2	0.22 (0.08–0.61)	0.000	0.21 (0.07–0.62)	NA	NA	NA	NA
Granisetron vs Ondansetron	10	0.67 (0.46–0.99)	0.000	0.76 (0.56–1.03)	NA	NA	NA	NA
Dolasetron vs Ondansetron	5	1.27 (0.89–1.80)	<0.0001	1.31 (0.92–1.87)	1	0.54 (0.20–1.45)	--	0.59 (0.21–1.67)
Tropisetron vs Ondansetron	5	1.00 (0.75–1.33)	<0.0001	1.04 (0.75–1.42)	NA	NA	NA	NA
Ondansetron+DEX vs Ondansetron	14	0.68 (0.48–0.96)	0.000	0.61 (0.42–0.89)	1	0.53 (0.11–2.60)	--	0.48 (0.16–1.43)
Palonosetron vs Ondansetron	3	0.29 (0.13–0.65)	0.000	0.66 (0.37–1.16)	NA	NA	NA	NA
Ramosetron vs Ondansetron	7	0.64 (0.39–1.03)	0.223	0.69 (0.47–1.01)	NA	NA	NA	NA
Ondansetron+DROP vs Ondansetron	6	0.63 (0.38–1.05)	0.000	0.57 (0.31–1.06)	1	0.19 (0.01–4.12)	--	0.17 (0.01–3.12)
Ondansetron+METO vs Ondansetron	2	0.59 (0.01–33.22)	6.615	0.66 (0.15–2.95)	1	5.43 (0.25–119.05)	--	2.65 (0.41–17.28)
Granisetron+DEX vs Ondansetron	NA	NA	NA	0.43 (0.25–0.73)	NA	NA	NA	0.21 (0.04–1.23)
Palonosetron+DEX vs Ondansetron	NA	NA	NA	0.79 (0.26–2.38)	NA	NA	NA	NA
Dolasetron+DEX vs Ondansetron	NA	NA	NA	0.46 (0.16–1.33)	NA	NA	NA	NA
Dolasetron+DROP vs Ondansetron	NA	NA	NA	0.41 (0.15–1.17)	NA	NA	NA	NA
Granisetron+DROP vs Ondansetron	NA	NA	NA	0.45 (0.15–1.35)	NA	NA	NA	NA
Dolasetron vs Granisetron	NA	NA	NA	1.73 (1.10–2.72)	NA	NA	NA	NA
Tropisetron vs Granisetron	1	3.00 (0.30–29.85)	--	1.37 (0.90–2.08)	NA	NA	NA	NA
Ondansetron+DEX vs Granisetron	NA	NA	NA	0.81 (0.50–1.29)	NA	NA	NA	NA

Palonosetron vs Granisetron	2	0.33 (0.07–1.54)	0.000	0.87 (0.47–1.62)	NA	NA	NA	NA
Ramosetron vs Granisetron	1	1.75 (0.61–4.99)	--	0.91 (0.57–1.46)	NA	NA	NA	NA
Ondansetron+DROP vs Granisetron	NA	NA	NA	0.76 (0.38–1.50)	NA	NA	NA	NA
Ondansetron+METO vs Granisetron	NA	NA	NA	0.87 (0.19–4.01)	NA	NA	NA	NA
Granisetron+DEX vs Granisetron	6	0.59 (0.39–0.88)	<0.0001	0.56 (0.33–0.95)	NA	NA	NA	NA
Palonosetron+DEX vs Granisetron	NA	NA	NA	1.04 (0.34–3.23)	NA	NA	NA	NA
Dolasetron+DEX vs Granisetron	NA	NA	NA	0.60 (0.20–1.82)	NA	NA	NA	NA
Dolasetron+DROP vs Granisetron	NA	NA	NA	0.55 (0.19–1.61)	NA	NA	NA	NA
Granisetron+DROP vs Granisetron	2	0.84 (0.26–2.68)	0.000	0.60 (0.20–1.79)	NA	NA	NA	NA
Tropisetron vs Dolasetron	1	0.83 (0.31–2.22)	--	0.79 (0.50–1.24)	NA	NA	NA	NA
Ondansetron+DEX vs Dolasetron	NA	NA	NA	0.47 (0.28–0.78)	NA	NA	NA	0.81 (0.19–3.50)
Palonosetron vs Dolasetron	NA	NA	NA	0.50 (0.26–0.97)	NA	NA	NA	NA
Ramosetron vs Dolasetron	NA	NA	NA	0.53 (0.31–0.88)	NA	NA	NA	NA
Ondansetron+DROP vs Dolasetron	NA	NA	NA	0.44 (0.21–0.89)	NA	NA	NA	0.28 (0.01–6.17)
Ondansetron+METO vs Dolasetron	NA	NA	NA	0.50 (0.11–2.35)	NA	NA	NA	4.47 (0.54–36.96)
Granisetron+DEX vs Dolasetron	NA	NA	NA	0.33 (0.17–0.62)	NA	NA	NA	0.36 (0.05–2.66)
Palonosetron+DEX vs Dolasetron	NA	NA	NA	0.60 (0.19–1.91)	NA	NA	NA	NA
Dolasetron+DEX vs Dolasetron	2	0.33 (0.15–0.72)	0.000	0.35 (0.13–0.97)	NA	NA	NA	NA
Dolasetron+DROP vs Dolasetron	1	0.35 (0.17–0.73)	--	0.32 (0.11–0.89)	NA	NA	NA	NA
Granisetron+DROP vs Dolasetron	NA	NA	NA	0.35 (0.11–1.08)	NA	NA	NA	NA
Ondansetron+DEX vs Tropisetron	NA	NA	NA	0.59 (0.36–0.95)	NA	NA	NA	NA
Palonosetron vs Tropisetron	NA	NA	NA	0.63 (0.34–1.20)	NA	NA	NA	NA
Ramosetron vs Tropisetron	NA	NA	NA	0.66 (0.41–1.08)	NA	NA	NA	NA
Ondansetron+DROP vs Tropisetron	NA	NA	NA	0.55 (0.28–1.10)	NA	NA	NA	NA
Ondansetron+METO vs Tropisetron	NA	NA	NA	0.64 (0.14–2.94)	NA	NA	NA	NA
Granisetron+DEX vs Tropisetron	NA	NA	NA	0.41 (0.22–0.76)	NA	NA	NA	NA
Palonosetron+DEX vs Tropisetron	NA	NA	NA	0.76 (0.24–2.38)	NA	NA	NA	NA
Dolasetron+DEX vs Tropisetron	NA	NA	NA	0.44 (0.15–1.33)	NA	NA	NA	NA
Dolasetron+DROP vs Tropisetron	NA	NA	NA	0.40 (0.14–1.17)	NA	NA	NA	NA
Granisetron+DROP vs Tropisetron	NA	NA	NA	0.44 (0.14–1.35)	NA	NA	NA	NA
Palonosetron vs Ondansetron+DEX	NA	NA	NA	1.08 (0.55–2.12)	NA	NA	NA	NA
Ramosetron vs Ondansetron+DEX	NA	NA	NA	1.13 (0.66–1.92)	NA	NA	NA	NA
Ondansetron+DROP vs Ondansetron+DEX	2	0.45 (0.19–1.09)	0.000	0.94 (0.47–1.86)	NA	NA	NA	0.35 (0.02–7.67)
Ondansetron+METO vs Ondansetron+DEX	NA	NA	NA	1.08 (0.23–5 .06)	NA	NA	NA	5.50 (0.66–45.96)
Granisetron+DEX vs Ondansetron+DEX	2	0.60 (0.19–1.94)	0.000	0.70 (0.37–1.31)	1	0.46 (0.08–2.75)	--	0.44 (0.07–2.66)
Palonosetron+DEX vs Ondansetron+DEX	NA	NA	NA	1.30 (0.41–4.13)	NA	NA	NA	NA
Dolasetron+DEX vs Ondansetron+DEX	NA	NA	NA	0.75 (0.24–2.32)	NA	NA	NA	NA

Dolasetron+DROP vs Ondansetron+DEX	NA	NA	NA	0.68 (0.22–2.05)	NA	NA	NA	NA
Granisetron+DROP vs Ondansetron+DEX	NA	NA	NA	0.74 (0.24–2.34)	NA	NA	NA	NA
Ramosetron vs Palonosetron	NA	NA	NA	1.05 (0.53–2.06)	NA	NA	NA	NA
Ondansetron+DROP vs Palonosetron	NA	NA	NA	0.87 (0.38–2.01)	NA	NA	NA	NA
Ondansetron+METO vs Palonosetron	NA	NA	NA	1.00 (0.20–4.97)	NA	NA	NA	NA
Granisetron+DEX vs Palonosetron	NA	NA	NA	0.65 (0.30–1.40)	NA	NA	NA	NA
Palonosetron+DEX vs Palonosetron	2	1.16 (0.61–2.19)	0.000	1.20 (0.47–3.09)	NA	NA	NA	NA
Dolasetron+DEX vs Palonosetron	NA	NA	NA	0.70 (0.21–2.31)	NA	NA	NA	NA
Dolasetron+DROP vs Palonosetron	NA	NA	NA	0.63 (0.19–2.05)	NA	NA	NA	NA
Granisetron+DROP vs Palonosetron	NA	NA	NA	0.69 (0.20–2.33)	NA	NA	NA	NA
Ondansetron+DROP vs Ramosetron	NA	NA	NA	0.83 (0.40–1.72)	NA	NA	NA	NA
Ondansetron+METO vs Ramosetron	NA	NA	NA	0.96 (0.20–4.50)	NA	NA	NA	NA
Granisetron+DEX vs Ramosetron	NA	NA	NA	0.62 (0.32–1.19)	NA	NA	NA	NA
Palonosetron+DEX vs Ramosetron	NA	NA	NA	1.15 (0.36–3.67)	NA	NA	NA	NA
Dolasetron+DEX vs Ramosetron	NA	NA	NA	0.67 (0.21–2.06)	NA	NA	NA	NA
Dolasetron+DROP vs Ramosetron	NA	NA	NA	0.60 (0.20–1.82)	NA	NA	NA	NA
Granisetron+DROP vs Ramosetron	NA	NA	NA	0.66 (0.21–2.08)	NA	NA	NA	NA
Ondansetron+METO vs Ondansetron+DROP	NA	NA	NA	1.15 (0.23–5.84)	NA	NA	NA	15.91 (0.50–503.02)
Granisetron+DEX vs Ondansetron+DROP	NA	NA	NA	0.75 (0.33–1.69)	NA	NA	NA	1.29 (0.04–37.91)
Palonosetron+DEX vs Ondansetron+DROP	NA	NA	NA	1.38 (0.39–4.88)	NA	NA	NA	NA
Dolasetron+DEX vs Ondansetron+DROP	NA	NA	NA	0.80 (0.23–2.74)	NA	NA	NA	NA
Dolasetron+DROP vs Ondansetron+DROP	NA	NA	NA	0.72 (0.22–2.43)	NA	NA	NA	NA
Granisetron+DROP vs Ondansetron+DROP	NA	NA	NA	0.80 (0.23–2.77)	NA	NA	NA	NA
Granisetron+DEX vs Ondansetron+METO	NA	NA	NA	0.65 (0.13–3.18)	NA	NA	NA	0.08 (0.01–1.00)
Palonosetron+DEX vs Ondansetron+METO	NA	NA	NA	1.20 (0.19–7.69)	NA	NA	NA	NA
Dolasetron+DEX vs Ondansetron+METO	NA	NA	NA	0.69 (0.11–4.36)	NA	NA	NA	NA
Dolasetron+DROP vs Ondansetron+METO	NA	NA	NA	0.63 (0.10–3.89)	NA	NA	NA	NA
Granisetron+DROP vs Ondansetron+METO	NA	NA	NA	0.69 (0.11–4.39)	NA	NA	NA	NA
Palonosetron+DEX vs Granisetron+DEX	NA	NA	NA	1.86 (0.55–6.27)	NA	NA	NA	NA
Dolasetron+DEX vs Granisetron+DEX	NA	NA	NA	1.07 (0.33–3.53)	NA	NA	NA	NA
Dolasetron+DROP vs Granisetron+DEX	NA	NA	NA	0.97 (0.30–3.12)	NA	NA	NA	NA
Granisetron+DROP vs Granisetron+DEX	2	0.72 (0.23–2.25)	0.000	1.07 (0.35–3.30)	NA	NA	NA	NA
Dolasetron+DEX vs Palonosetron+DEX	NA	NA	NA	0.58 (0.13–2.67)	NA	NA	NA	NA
Dolasetron+DROP vs Palonosetron+DEX	NA	NA	NA	0.52 (0.12–2.37)	NA	NA	NA	NA
Granisetron+DROP vs Palonosetron+DEX	NA	NA	NA	0.58 (0.12–2.68)	NA	NA	NA	NA
Dolasetron+DROP vs Dolasetron+DEX	NA	NA	NA	0.90 (0.21–3.86)	NA	NA	NA	NA
Granisetron+DROP vs Dolasetron+DEX	NA	NA	NA	0.99 (0.22–4.53)	NA	NA	NA	NA

Granisetron+DROP vs Dolasetron+DROP	NA	NA	NA	1.10 (0.25–4.94)	NA	NA	NA	NA
**Between-study heterogeneity**	**0.213**				**0.097**			
**Design-by-treatment interaction model for inconsistency (χ^2, d.f., P-value, heterogeneity)**	**23.47 (41, 0.988, 0.264)**				**0.42 (5, 0.995, 0.846)**			
	**PONV (all ages) - 125 RCTs and 16,667 patients**				**PONV (children only) - 14 RCTs and 2,394 patients**			
Ondansetron vs Placebo	89	0.30 (0.26–0.35)	0.212	0.31 (0.27–0.36)	11	0.33 (0.18–0.60)	0.804	0.34 (0.19–0.62)
Granisetron vs Placebo	16	0.23 (0.14–0.37)	0.521	0.23 (0.16–0.32)	2	0.54 (0.26–1.12)	0.088	0.32 (0.08–1.23)
Dolasetron vs Placebo	5	0.27 (0.14–0.52)	0.323	0.25 (0.14–0.43)	NA	NA	NA	NA
Tropisetron vs Placebo	8	0.39 (0.28–0.53)	< 0.0001	0.36 (0.24–0.54)	NA	NA	NA	NA
Ondansetron+DEX vs Placebo	8	0.12 (0.07–0.20)	0.124	0.15 (0.10–0.22)	1	0.20 (0.06–0.66)	--	0.27 (0.04–1.61)
Palonosetron vs Placebo	NA	NA	NA	0.11 (0.03–0.40)	NA	NA	NA	NA
Ramosetron vs Placebo	4	0.30 (0.15–0.59)	0.325	0.26 (0.16–0.41)	NA	NA	NA	NA
Ondansetron+DROP vs Placebo	2	0.13 (0.05–0.34)	0.000	0.11 (0.05–0.24)	1	0.12 (0.04–0.34)	--	0.12 (0.02–0.71)
Granisetron+DEX vs Placebo	4	0.06 (0.02–0.17)	0.756	0.09 (0.05–0.16)	NA	NA	NA	NA
Palonosetron+DEX vs Placebo	NA	NA	NA	0.12 (0.02–0.64)	NA	NA	NA	NA
Dolasetron+DROP vs Placebo	1	0.17 (0.08–0.36)	--	0.12 (0.04–0.35)	NA	NA	NA	NA
Granisetron+DROP vs Placebo	2	0.17 (0.07–0.38)	0.000	0.16 (0.06–0.40)	NA	NA	NA	NA
Granisetron vs Ondansetron	9	0.75 (0.48–1.18)	0.047	0.73 (0.52–1.03)	1	0.31 (0.03–3.16)	--	0.92 (0.22–3.88)
Dolasetron vs Ondansetron	3	0.61 (0.34–1.10)	0.000	0.79 (0.45–1.39)	NA	NA	NA	NA
Tropisetron vs Ondansetron	3	0.90 (0.30–2.69)	0.452	1.14 (0.74–1.75)	NA	NA	NA	NA
Ondansetron+DEX vs Ondansetron	11	0.48 (0.33–0.72)	0.104	0.46 (0.31–0.69)	1	1.00 (0.36–2.75)	--	0.78 (0.13–4.65)
Palonosetron vs Ondansetron	1	0.37 (0.16–0.86)	--	0.37 (0.11–1.26)	NA	NA	NA	NA
Ramosetron vs Ondansetron	4	0.74 (0.48–1.13)	0.022	0.82 (0.51–1.30)	1	0.35 (0.13–0.95)	--	0.35 (0.06–2.07)
Ondansetron+DROP vs Ondansetron	3	0.39 (0.20–0.75)	0.000	0.35 (0.16–0.77)	NA	NA	NA	NA
Granisetron+DEX vs Ondansetron	1	0.22 (0.04–1.21)	--	0.30 (0.17–0.53)	NA	NA	NA	NA
Palonosetron+DEX vs Ondansetron	NA	NA	NA	0.39 (0.07–2.01)	NA	NA	NA	NA
Dolasetron+DROP vs Ondansetron	NA	NA	NA	0.39 (0.14–1.14)	NA	NA	NA	NA
Granisetron+DROP vs Ondansetron	NA	NA	NA	0.51 (0.20–1.28)	NA	NA	NA	NA
Dolasetron vs Granisetron	NA	NA	NA	1.09 (0.57–2.08)	NA	NA	NA	NA
Tropisetron vs Granisetron	1	1.17 (0.39–3.56)	--	1.56 (0.93–2.63)	NA	NA	NA	NA
Ondansetron+DEX vs Granisetron	1	0.72 (0.14–3.59)	--	0.64 (0.38–1.06)	NA	NA	NA	NA
Palonosetron vs Granisetron	NA	NA	NA	0.50 (0.14–1.82)	NA	NA	NA	0.85 (0.09–7.93)
Ramosetron vs Granisetron	1	2.27 (0.87–5.96)	--	1.12 (0.64–1.95)	NA	NA	NA	0.39 (0.04–3.53)
Ondansetron+DROP vs Granisetron	NA	NA	NA	0.48 (0.20–1.13)	NA	NA	NA	NA
Granisetron+DEX vs Granisetron	8	0.48 (0.29–0.77)	0.094	0.41 (0.24–0.69)	NA	NA	NA	NA
Palonosetron+DEX vs Granisetron	NA	NA	NA	0.53 (0.10–2.87)	NA	NA	NA	NA
Dolasetron+DROP vs Granisetron	NA	NA	NA	0.54 (0.18–1.63)	NA	NA	NA	NA
Granisetron+DROP vs Granisetron	2	0.91 (0.38–2.17)	0.000	0.70 (0.28–1.74)	NA	NA	NA	NA

Tropisetron vs Dolasetron	NA	NA	NA	1.43 (0.72–2.85)	NA	NA	NA	NA
Ondansetron+DEX vs Dolasetron	NA	NA	NA	0.58 (0.30–1.15)	NA	NA	NA	NA
Palonosetron vs Dolasetron	NA	NA	NA	0.46 (0.12–1.79)	NA	NA	NA	NA
Ramosetron vs Dolasetron	NA	NA	NA	1.03 (0.50–2.11)	NA	NA	NA	NA
Ondansetron+DROP vs Dolasetron	NA	NA	NA	0.44 (0.17–1.16)	NA	NA	NA	NA
Granisetron+DEX vs Dolasetron	NA	NA	NA	0.37 (0.17–0.82)	NA	NA	NA	NA
Palonosetron+DEX vs Dolasetron	NA	NA	NA	0.48 (0.08–2.78)	NA	NA	NA	NA
Dolasetron+DROP vs Dolasetron	1	0.35 (0.17–0.73)	--	0.49 (0.17–1.42)	NA	NA	NA	NA
Granisetron+DROP vs Dolasetron	NA	NA	NA	0.64 (0.22–1.86)	NA	NA	NA	NA
Ondansetron+DEX vs Tropisetron	NA	NA	NA	0.41 (0.23–0.73)	NA	NA	NA	NA
Palonosetron vs Tropisetron	NA	NA	NA	0.32 (0.09–1.19)	NA	NA	NA	NA
Ramosetron vs Tropisetron	NA	NA	NA	0.72 (0.39–1.33)	NA	NA	NA	NA
Ondansetron+DROP vs Tropisetron	NA	NA	NA	0.31 (0.13–0.75)	NA	NA	NA	NA
Granisetron+DEX vs Tropisetron	NA	NA	NA	0.26 (0.13–0.52)	NA	NA	NA	NA
Palonosetron+DEX vs Tropisetron	NA	NA	NA	0.34 (0.06–1.87)	NA	NA	NA	NA
Dolasetron+DROP vs Tropisetron	NA	NA	NA	0.34 (0.11–1.07)	NA	NA	NA	NA
Granisetron+DROP vs Tropisetron	NA	NA	NA	0.44 (0.16–1.22)	NA	NA	NA	NA
Palonosetron vs Ondansetron+DEX	NA	NA	NA	0.79 (0.21–2.90)	NA	NA	NA	NA
Ramosetron vs Ondansetron+DEX	NA	NA	NA	1.76 (0.96–3.23)	NA	NA	NA	NA
Ondansetron+DROP vs Ondansetron+DEX	1	0.52 (0.21–1.29)	--	0.75 (0.33–1.75)	NA	NA	NA	0.45 (0.04–5.38)
Granisetron+DEX vs Ondansetron+DEX	1	0.64 (0.10–4.19)	--	0.64 (0.32–1.27)	NA	NA	NA	NA
Palonosetron+DEX vs Ondansetron+DEX	NA	NA	NA	0.83 (0.15–4.55)	NA	NA	NA	NA
Dolasetron+DROP vs Ondansetron+DEX	NA	NA	NA	0.85 (0.27–2.62)	NA	NA	NA	NA
Granisetron+DROP vs Ondansetron+DEX	NA	NA	NA	1.09 (0.40–2.97)	NA	NA	NA	NA
Ramosetron vs Palonosetron	NA	NA	NA	2.23 (0.59–8.40)	NA	NA	NA	NA
Ondansetron+DROP vs Palonosetron	NA	NA	NA	0.96 (0.22–4.17)	NA	NA	NA	NA
Granisetron+DEX vs Palonosetron	NA	NA	NA	0.81 (0.21–3.19)	NA	NA	NA	NA
Palonosetron+DEX vs Palonosetron	2	0.99 (0.48–2.01)	0.000	1.05 (0.35–3.14)	NA	NA	NA	NA
Dolasetron+DROP vs Palonosetron	NA	NA	NA	1.07 (0.21–5.50)	NA	NA	NA	NA
Granisetron+DROP vs Palonosetron	NA	NA	NA	1.38 (0.29–6.52)	NA	NA	NA	NA
Ondansetron+DROP vs Ramosetron	NA	NA	NA	0.43 (0.17–1.07)	NA	NA	NA	NA
Granisetron+DEX vs Ramosetron	NA	NA	NA	0.36 (0.18–0.75)	NA	NA	NA	NA
Palonosetron+DEX vs Ramosetron	NA	NA	NA	0.47 (0.08–2.63)	NA	NA	NA	NA
Dolasetron+DROP vs Ramosetron	NA	NA	NA	0.48 (0.15–1.53)	NA	NA	NA	NA
Granisetron+DROP vs Ramosetron	NA	NA	NA	0.62 (0.22–1.73)	NA	NA	NA	NA
Granisetron+DEX vs Ondansetron+DROP	NA	NA	NA	0.85 (0.32–2.24)	NA	NA	NA	NA
Palonosetron+DEX vs Ondansetron+DROP	NA	NA	NA	1.10 (0.18–6.87)	NA	NA	NA	NA
Dolasetron+DROP vs Ondansetron+DROP	NA	NA	NA	1.12 (0.30–4.20)	NA	NA	NA	NA

Granisetron+DROP vs Ondansetron+DROP	NA	NA	NA	1.44 (0.43–4.86)	NA	NA	NA	NA
Palonosetron+DEX vs Granisetron+DEX	NA	NA	NA	1.30 (0.23–7.48)	NA	NA	NA	NA
Dolasetron+DROP vs Granisetron+DEX	NA	NA	NA	1.32 (0.40–4.38)	NA	NA	NA	NA
Granisetron+DROP vs Granisetron+DEX	2	1.23 (0.50–3.07)	0.000	1.70 (0.65–4.47)	NA	NA	NA	NA
Dolasetron+DROP vs Palonosetron+DEX	NA	NA	NA	1.02 (0.14–7.27)	NA	NA	NA	NA
Granisetron+DROP vs Palonosetron+DEX	NA	NA	NA	1.31 (0.20–8.75)	NA	NA	NA	NA
Granisetron+DROP vs Dolasetron+DROP	NA	NA	NA	1.29 (0.32–5.24)	NA	NA	NA	NA
**Between-study heterogeneity**	**0.209**				**0.777**			
**Design-by-treatment interaction model for inconsistency (χ^2, d.f., P-value, heterogeneity)**	**27.98 (31, 0.622, 0.212)**				**0.84 (3, 0.841, 0.950)**			

Abbreviations: DEX dexamethasone, DROP droperidol (intravenous), METO Metoclopramide (intravenous), PONV post-operative nausea and vomiting, RCTs Randomized Controlled Trials, OR Odds Ratio, CI Confidence Interval, MA Meta-analysis, NMA Network Meta-analysis
Note: Treatment comparisons with - - for heterogeneity variance indicate that the between-study heterogeneity variance could not be estimated because a single study was included in the analysis

Appendix 12. Plot of the surface under the cumulative ranking (SUCRA) curves for the 15 treatments included in Vomiting.

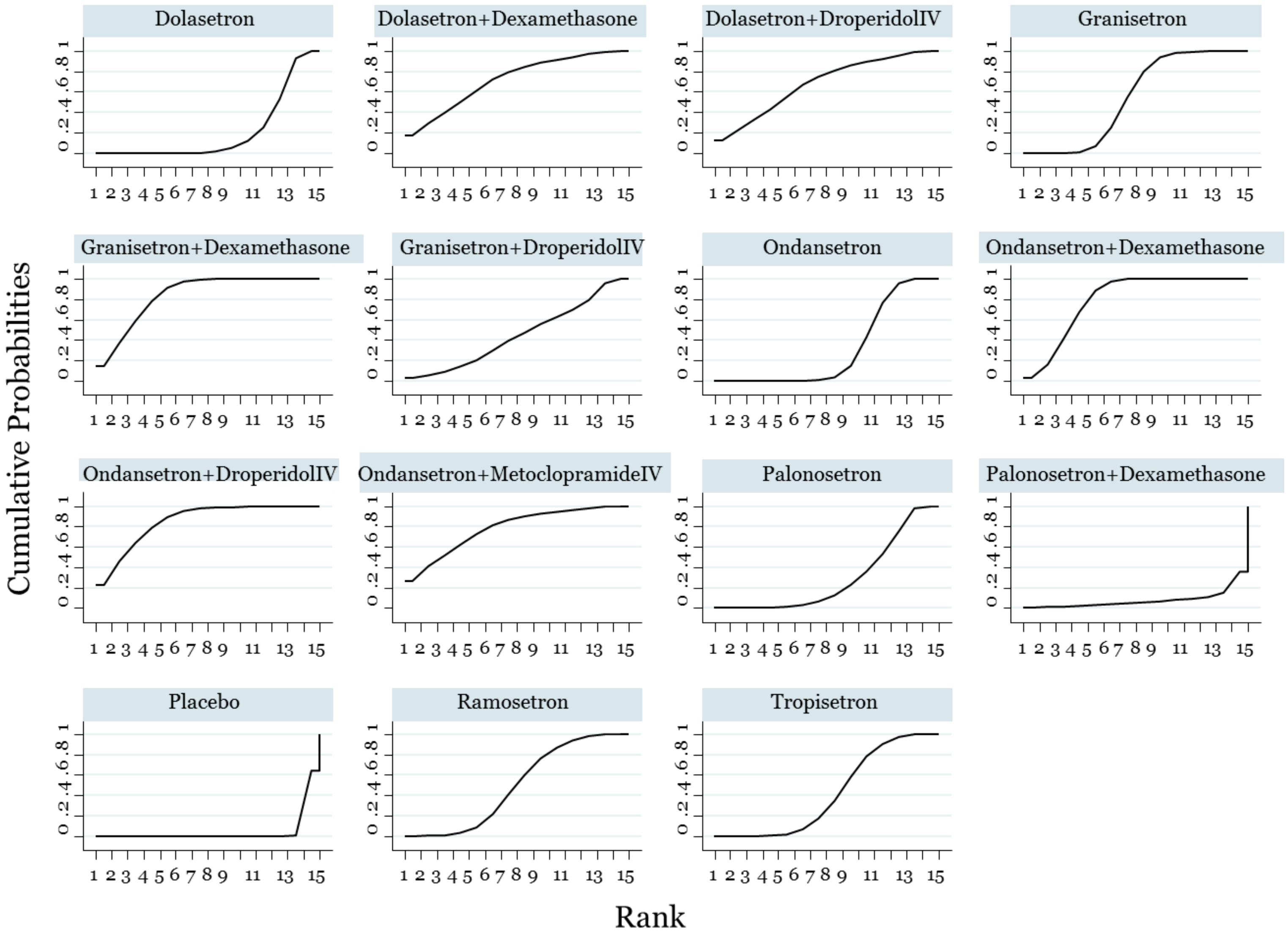

Appendix 13. Network plots using colored edges according to the levels of the treatment effect modifier age of participants*

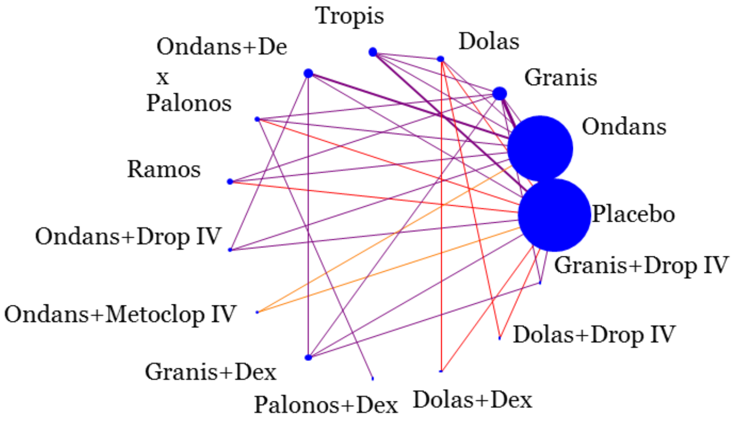

a. Network of Vomiting

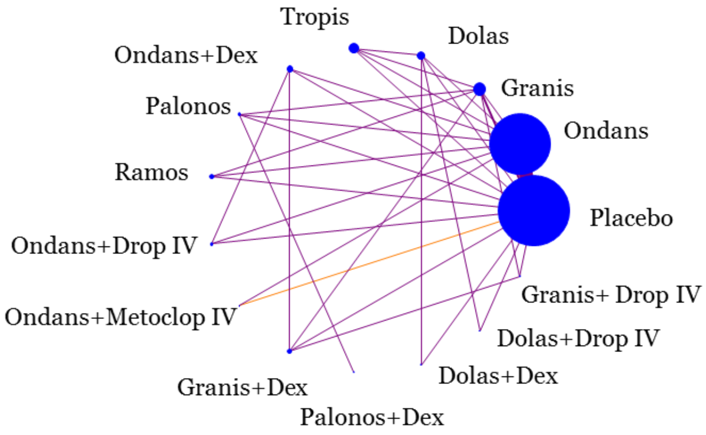

b. Network of Nausea

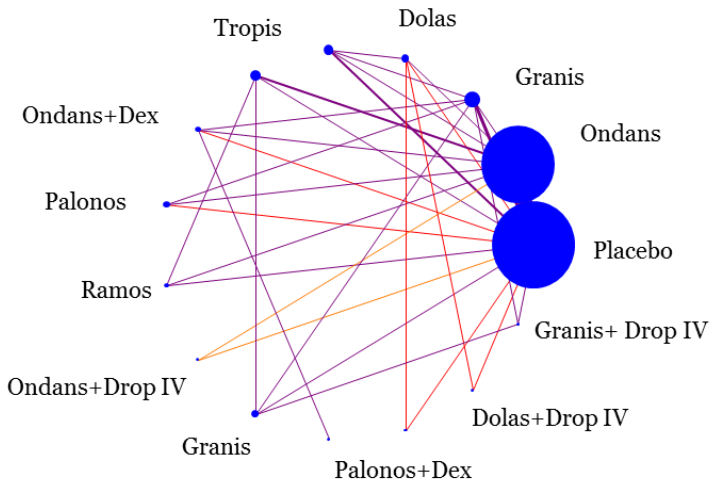

c. Network of PONV

*Note: Each edge has been colored according to the mode of the study-specific levels: **Orange** corresponds to children, **Purple** corresponds to adults, and **Green** corresponds to all ages.

Appendix 14. Network plots using colored edges according to the levels of the treatment effect modifier timing of administration

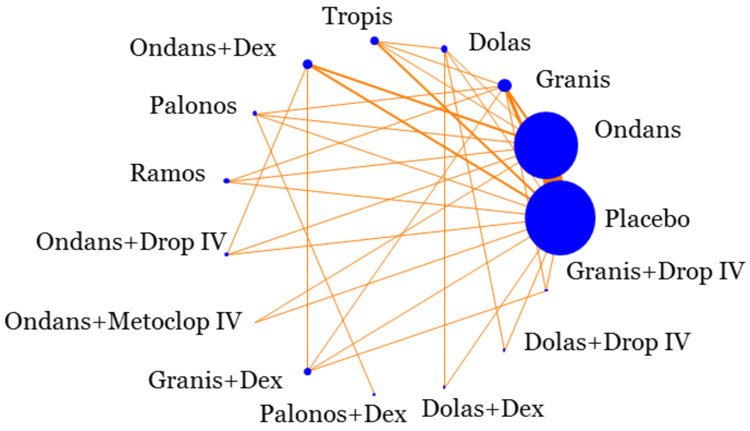

a. Network of Vomiting

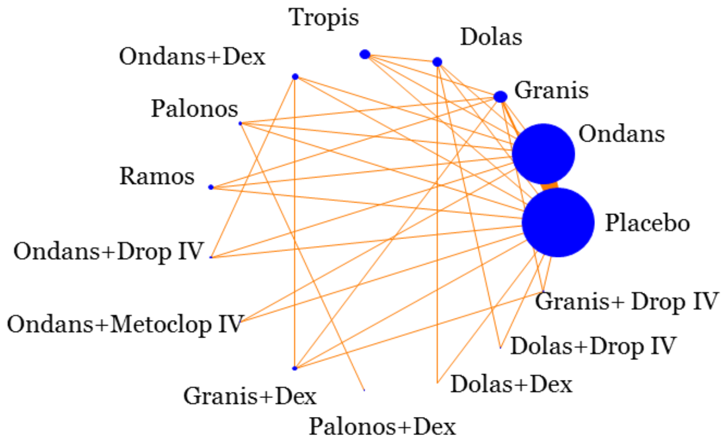

b. Network of Nausea

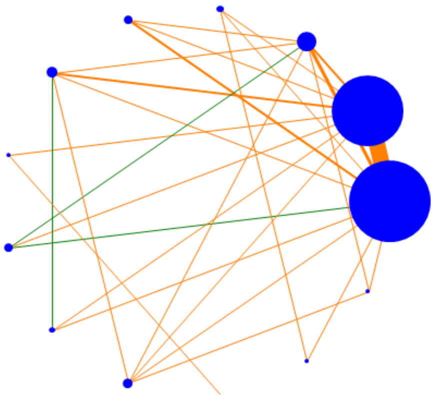

c. Network of PONV

*Note: Each edge has been colored according to the mode of the study-specific levels: **Orange** corresponds to administration during surgery, **Purple** corresponds to administration during and after surgery, **Green** corresponds to administration after surgery, and **Blue** corresponds to Pre.

Appendix 15. Vomiting sub-group analysis results

VOMITING	*All time periods - all study designs*				*All time periods - RCTs only - sensitivity analysis for Risk of Bias*				*Intra-operative - RCTs only*			
**Treatment Comparison**	**OR**	**CI_ Low**	**CI_ High**	**Hetero geneity**	**OR**	**CI_ Low**	**CI_ High**	**Hetero geneity**	**OR**	**CI_ Low**	**CI_ High**	**Hetero geneity**
Ondansetron vs Placebo	0.366	0.330	0.407	0.152	0.360	0.323	0.400	0.132	0.355	0.318	0.397	0.129
Granisetron vs Placebo	0.263	0.204	0.339	0.152	0.268	0.208	0.345	0.132	0.263	0.205	0.337	0.129
Dolasetron vs Placebo	0.423	0.292	0.613	0.152	0.433	0.295	0.634	0.132	0.433	0.300	0.624	0.129
Tropisetron vs Placebo	0.312	0.224	0.433	0.152	0.331	0.238	0.461	0.132	0.315	0.229	0.433	0.129
Ondansetron+Dexamethasone vs Placebo	0.180	0.132	0.245	0.152	0.166	0.119	0.232	0.132	0.165	0.118	0.231	0.129
Palonosetron vs Placebo	0.376	0.234	0.605	0.152	0.382	0.241	0.605	0.132	0.382	0.241	0.604	0.129
Ramosetron vs Placebo	0.285	0.185	0.438	0.152	0.281	0.184	0.429	0.132	0.357	0.229	0.557	0.129
Ondansetron+DroperidolIV vs Placebo	0.146	0.079	0.269	0.152	0.141	0.077	0.259	0.132	0.127	0.064	0.251	0.129
Ondansetron+MetoclopramideIV vs Placebo	0.153	0.054	0.428	0.152	0.152	0.055	0.421	0.132	0.151	0.055	0.418	0.129
Granisetron+Dexamethasone vs Placebo	0.150	0.091	0.248	0.152	0.147	0.089	0.241	0.132	0.149	0.091	0.243	0.129
Palonosetron+Dexamethasone vs Placebo	1.403	0.195	10.120	0.152	1.426	0.201	10.140	0.132	1.426	0.201	10.112	0.129
Dolasetron+Dexamethasone vs Placebo	0.172	0.061	0.484	0.152	0.177	0.064	0.493	0.132	0.178	0.065	0.490	0.129
Dolasetron+DroperidolIV vs Placebo	0.192	0.070	0.530	0.152	0.194	0.072	0.520	0.132	0.194	0.073	0.516	0.129
Granisetron+DroperidolIV vs Placebo	0.305	0.112	0.831	0.152	0.307	0.114	0.824	0.132	0.306	0.114	0.819	0.129
Granisetron vs Ondansetron	0.717	0.550	0.934	0.152	0.745	0.572	0.971	0.132	0.740	0.571	0.960	0.129
Dolasetron vs Ondansetron	1.155	0.796	1.677	0.152	1.203	0.818	1.770	0.132	1.218	0.841	1.765	0.129
Tropisetron vs Ondansetron	0.850	0.606	1.193	0.152	0.920	0.655	1.293	0.132	0.886	0.637	1.231	0.129
Ondansetron+Dexamethasone vs Ondansetron	0.492	0.362	0.668	0.152	0.462	0.332	0.643	0.132	0.465	0.333	0.648	0.129
Palonosetron vs Ondansetron	1.026	0.635	1.659	0.152	1.062	0.667	1.691	0.132	1.074	0.676	1.707	0.129

Ramosetron vs Ondansetron	0.777	0.505	1.194	0.152	0.782	0.513	1.190	0.132	1.005	0.643	1.570	0.129
Ondansetron+DroperidolIV vs Ondansetron	0.398	0.216	0.734	0.152	0.393	0.215	0.718	0.132	0.357	0.181	0.707	0.129
Ondansetron+MetoclopramideIV vs Ondansetron	0.417	0.148	1.170	0.152	0.423	0.153	1.170	0.132	0.426	0.154	1.176	0.129
Granisetron+Dexamethasone vs Ondansetron	0.410	0.248	0.680	0.152	0.408	0.247	0.672	0.132	0.419	0.256	0.687	0.129
Palonosetron+Dexamethasone vs Ondansetron	3.830	0.530	27.652	0.152	3.965	0.557	28.223	0.132	4.012	0.565	28.493	0.129
Dolasetron+Dexamethasone vs Ondansetron	0.468	0.166	1.323	0.152	0.493	0.177	1.371	0.132	0.500	0.181	1.381	0.129
Dolasetron+DroperidolIV vs Ondansetron	0.524	0.189	1.451	0.152	0.538	0.200	1.451	0.132	0.545	0.203	1.460	0.129
Granisetron+DroperidolIV vs Ondansetron	0.832	0.304	2.275	0.152	0.852	0.316	2.299	0.132	0.860	0.320	2.314	0.129
Dolasetron vs Granisetron	1.612	1.032	2.519	0.152	1.615	1.023	2.547	0.132	1.646	1.060	2.554	0.129
Tropisetron vs Granisetron	1.187	0.785	1.793	0.152	1.235	0.817	1.865	0.132	1.196	0.802	1.783	0.129
Ondansetron+Dexamethasone vs Granisetron	0.686	0.466	1.012	0.152	0.619	0.412	0.932	0.132	0.628	0.419	0.942	0.129
Palonosetron vs Granisetron	1.432	0.847	2.423	0.152	1.425	0.854	2.376	0.132	1.451	0.874	2.409	0.129
Ramosetron vs Granisetron	1.084	0.661	1.777	0.152	1.049	0.646	1.703	0.132	1.357	0.822	2.242	0.129
Ondansetron+DroperidolIV vs Granisetron	0.555	0.286	1.076	0.152	0.527	0.274	1.012	0.132	0.483	0.234	0.995	0.129
Ondansetron+MetoclopramideIV vs Granisetron	0.581	0.201	1.681	0.152	0.567	0.199	1.616	0.132	0.575	0.203	1.633	0.129
Granisetron+Dexamethasone vs Granisetron	0.573	0.347	0.946	0.152	0.547	0.333	0.897	0.132	0.566	0.347	0.924	0.129
Palonosetron+Dexamethasone vs Granisetron	5.345	0.732	39.039	0.152	5.319	0.739	38.298	0.132	5.420	0.755	38.902	0.129
Dolasetron+Dexamethasone vs Granisetron	0.654	0.225	1.897	0.152	0.662	0.232	1.890	0.132	0.676	0.239	1.915	0.129
Dolasetron+DroperidolIV vs Granisetron	0.731	0.257	2.082	0.152	0.722	0.261	2.001	0.132	0.736	0.267	2.024	0.129
Granisetron+DroperidolIV vs Granisetron	1.161	0.424	3.176	0.152	1.143	0.424	3.084	0.132	1.161	0.432	3.124	0.129
Tropisetron vs Dolasetron	0.736	0.456	1.188	0.152	0.765	0.470	1.245	0.132	0.727	0.455	1.162	0.129

Ondansetron+Dexamethasone vs Dolasetron	0.426	0.265	0.685	0.152	0.384	0.232	0.635	0.132	0.381	0.233	0.624	0.129
Palonosetron vs Dolasetron	0.888	0.488	1.618	0.152	0.882	0.486	1.601	0.132	0.882	0.492	1.581	0.129
Ramosetron vs Dolasetron	0.672	0.382	1.182	0.152	0.650	0.369	1.143	0.132	0.825	0.465	1.462	0.129
Ondansetron+DroperidolIV vs Dolasetron	0.344	0.169	0.703	0.152	0.326	0.160	0.665	0.132	0.293	0.136	0.634	0.129
Ondansetron+MetoclopramideIV vs Dolasetron	0.361	0.121	1.077	0.152	0.351	0.119	1.040	0.132	0.350	0.119	1.027	0.129
Granisetron+Dexamethasone vs Dolasetron	0.355	0.191	0.660	0.152	0.339	0.182	0.631	0.132	0.344	0.187	0.632	0.129
Palonosetron+Dexamethasone vs Dolasetron	3.315	0.445	24.726	0.152	3.295	0.447	24.286	0.132	3.293	0.449	24.143	0.129
Dolasetron+Dexamethasone vs Dolasetron	0.406	0.150	1.093	0.152	0.410	0.155	1.083	0.132	0.411	0.156	1.081	0.129
Dolasetron+DroperidolIV vs Dolasetron	0.453	0.163	1.261	0.152	0.447	0.165	1.210	0.132	0.447	0.166	1.202	0.129
Granisetron+DroperidolIV vs Dolasetron	0.720	0.247	2.095	0.152	0.708	0.246	2.042	0.132	0.706	0.247	2.018	0.129
Ondansetron+Dexamethasone vs Tropisetron	0.578	0.370	0.904	0.152	0.502	0.314	0.800	0.132	0.525	0.331	0.831	0.129
Palonosetron vs Tropisetron	1.207	0.681	2.140	0.152	1.154	0.659	2.020	0.132	1.213	0.699	2.104	0.129
Ramosetron vs Tropisetron	0.913	0.531	1.570	0.152	0.849	0.498	1.449	0.132	1.135	0.658	1.958	0.129
Ondansetron+DroperidolIV vs Tropisetron	0.468	0.233	0.938	0.152	0.427	0.214	0.849	0.132	0.404	0.190	0.856	0.129
Ondansetron+MetoclopramideIV vs Tropisetron	0.490	0.166	1.445	0.152	0.459	0.158	1.337	0.132	0.481	0.166	1.392	0.129
Granisetron+Dexamethasone vs Tropisetron	0.483	0.266	0.874	0.152	0.443	0.245	0.799	0.132	0.474	0.266	0.844	0.129
Palonosetron+Dexamethasone vs Tropisetron	4.504	0.609	33.324	0.152	4.308	0.591	31.427	0.132	4.531	0.624	32.906	0.129
Dolasetron+Dexamethasone vs Tropisetron	0.551	0.187	1.622	0.152	0.536	0.185	1.552	0.132	0.565	0.197	1.621	0.129
Dolasetron+DroperidolIV vs Tropisetron	0.616	0.212	1.786	0.152	0.585	0.207	1.651	0.132	0.615	0.220	1.721	0.129
Granisetron+DroperidolIV vs Tropisetron	0.978	0.341	2.807	0.152	0.926	0.327	2.622	0.132	0.971	0.345	2.732	0.129

Palonosetron vs Ondansetron+Dexamethasone	2.086	1.191	3.655	0.152	2.300	1.307	4.045	0.132	2.312	1.316	4.060	0.129
Ramosetron vs Ondansetron+Dexamethasone	1.579	0.935	2.666	0.152	1.693	0.995	2.881	0.132	2.162	1.246	3.754	0.129
Ondansetron+DroperidolIV vs Ondansetron+Dexamethasone	0.809	0.422	1.548	0.152	0.851	0.445	1.625	0.132	0.769	0.372	1.591	0.129
Ondansetron+MetoclopramideIV vs Ondansetron+Dexamethasone	0.847	0.289	2.479	0.152	0.916	0.315	2.665	0.132	0.917	0.316	2.662	0.129
Granisetron+Dexamethasone vs Ondansetron+Dexamethasone	0.834	0.485	1.436	0.152	0.883	0.509	1.531	0.132	0.902	0.525	1.551	0.129
Palonosetron+Dexamethasone vs Ondansetron+Dexamethasone	7.786	1.056	57.415	0.152	8.588	1.176	62.730	0.132	8.633	1.185	62.921	0.129
Dolasetron+Dexamethasone vs Ondansetron+Dexamethasone	0.952	0.324	2.800	0.152	1.068	0.366	3.120	0.132	1.077	0.371	3.125	0.129
Dolasetron+DroperidolIV vs Ondansetron+Dexamethasone	1.065	0.369	3.075	0.152	1.166	0.411	3.305	0.132	1.172	0.416	3.305	0.129
Granisetron+DroperidolIV vs Ondansetron+Dexamethasone	1.691	0.596	4.798	0.152	1.846	0.654	5.210	0.132	1.850	0.657	5.208	0.129
Ramosetron vs Palonosetron	0.757	0.399	1.436	0.152	0.736	0.395	1.372	0.132	0.935	0.495	1.767	0.129
Ondansetron+DroperidolIV vs Palonosetron	0.388	0.179	0.841	0.152	0.370	0.173	0.790	0.132	0.333	0.147	0.755	0.129
Ondansetron+MetoclopramideIV vs Palonosetron	0.406	0.131	1.262	0.152	0.398	0.131	1.213	0.132	0.397	0.131	1.204	0.129
Granisetron+Dexamethasone vs Palonosetron	0.400	0.203	0.790	0.152	0.384	0.197	0.747	0.132	0.390	0.202	0.755	0.129
Palonosetron+Dexamethasone vs Palonosetron	3.732	0.548	25.399	0.152	3.734	0.555	25.131	0.132	3.735	0.556	25.087	0.129
Dolasetron+Dexamethasone vs Palonosetron	0.457	0.147	1.422	0.152	0.464	0.152	1.417	0.132	0.466	0.154	1.410	0.129
Dolasetron+DroperidolIV vs Palonosetron	0.510	0.166	1.567	0.152	0.507	0.170	1.507	0.132	0.507	0.172	1.497	0.129
Granisetron+DroperidolIV vs Palonosetron	0.811	0.268	2.449	0.152	0.803	0.271	2.379	0.132	0.800	0.271	2.363	0.129
Ondansetron+DroperidolIV vs Ramosetron	0.512	0.243	1.080	0.152	0.502	0.242	1.045	0.132	0.356	0.158	0.800	0.129
Ondansetron+MetoclopramideIV	0.536	0.176	1.638	0.152	0.541	0.180	1.623	0.132	0.424	0.140	1.281	0.129

vs Ramosetron												
Granisetron+Dexamethasone vs Ramosetron	0.528	0.274	1.019	0.152	0.521	0.273	0.995	0.132	0.417	0.216	0.804	0.129
Palonosetron+Dexamethasone vs Ramosetron	4.931	0.653	37.239	0.152	5.072	0.683	37.690	0.132	3.993	0.536	29.741	0.129
Dolasetron+Dexamethasone vs Ramosetron	0.603	0.196	1.852	0.152	0.631	0.209	1.901	0.132	0.498	0.165	1.504	0.129
Dolasetron+DroperidolIV vs Ramosetron	0.674	0.224	2.032	0.152	0.688	0.235	2.013	0.132	0.542	0.185	1.591	0.129
Granisetron+DroperidolIV vs Ramosetron	1.071	0.360	3.186	0.152	1.090	0.373	3.189	0.132	0.856	0.291	2.519	0.129
Ondansetron+MetoclopramideIV vs Ondansetron+DroperidolIV	1.048	0.316	3.473	0.152	1.077	0.330	3.507	0.132	1.192	0.352	4.041	0.129
Granisetron+Dexamethasone vs Ondansetron+DroperidolIV	1.032	0.471	2.258	0.152	1.038	0.479	2.246	0.132	1.174	0.511	2.693	0.129
Palonosetron+Dexamethasone vs Ondansetron+DroperidolIV	9.629	1.217	76.188	0.152	10.095	1.297	78.573	0.132	11.228	1.411	89.308	0.129
Dolasetron+Dexamethasone vs Ondansetron+DroperidolIV	1.178	0.354	3.924	0.152	1.255	0.384	4.107	0.132	1.400	0.413	4.745	0.129
Dolasetron+DroperidolIV vs Ondansetron+DroperidolIV	1.317	0.402	4.313	0.152	1.370	0.431	4.359	0.132	1.524	0.462	5.033	0.129
Granisetron+DroperidolIV vs Ondansetron+DroperidolIV	2.091	0.646	6.767	0.152	2.170	0.682	6.903	0.132	2.406	0.727	7.967	0.129
Granisetron+Dexamethasone vs Ondansetron+MetoclopramideIV	0.985	0.314	3.094	0.152	0.964	0.312	2.982	0.132	0.984	0.320	3.029	0.129
Palonosetron+Dexamethasone vs Ondansetron+MetoclopramideIV	9.192	0.990	85.306	0.152	9.378	1.030	85.342	0.132	9.417	1.038	85.419	0.129
Dolasetron+Dexamethasone vs Ondansetron+MetoclopramideIV	1.124	0.261	4.846	0.152	1.166	0.276	4.919	0.132	1.175	0.280	4.920	0.129
Dolasetron+DroperidolIV vs Ondansetron+MetoclopramideIV	1.257	0.296	5.346	0.152	1.273	0.308	5.252	0.132	1.278	0.312	5.244	0.129
Granisetron+DroperidolIV vs Ondansetron+MetoclopramideIV	1.996	0.474	8.409	0.152	2.016	0.488	8.320	0.132	2.018	0.491	8.298	0.129

Palonosetron+Dexamethasone vs Granisetron+Dexamethasone	9.332	1.220	71.395	0.152	9.728	1.291	73.293	0.132	9.567	1.275	71.802	0.129
Dolasetron+Dexamethasone vs Granisetron+Dexamethasone	1.141	0.362	3.596	0.152	1.210	0.390	3.749	0.132	1.193	0.389	3.663	0.129
Dolasetron+DroperidolIV vs Granisetron+Dexamethasone	1.276	0.412	3.958	0.152	1.320	0.437	3.985	0.132	1.299	0.434	3.886	0.129
Granisetron+DroperidolIV vs Granisetron+Dexamethasone	2.027	0.695	5.913	0.152	2.091	0.727	6.013	0.132	2.050	0.716	5.872	0.129
Dolasetron+Dexamethasone vs Palonosetron+Dexamethasone	0.122	0.013	1.136	0.152	0.124	0.014	1.132	0.132	0.125	0.014	1.129	0.129
Dolasetron+DroperidolIV vs Palonosetron+Dexamethasone	0.137	0.015	1.261	0.152	0.136	0.015	1.220	0.132	0.136	0.015	1.214	0.129
Granisetron+DroperidolIV vs Palonosetron+Dexamethasone	0.217	0.024	1.987	0.152	0.215	0.024	1.929	0.132	0.214	0.024	1.917	0.129
Dolasetron+DroperidolIV vs Dolasetron+Dexamethasone	1.118	0.271	4.607	0.152	1.091	0.274	4.347	0.132	1.088	0.275	4.307	0.129
Granisetron+DroperidolIV vs Dolasetron+Dexamethasone	1.775	0.420	7.500	0.152	1.728	0.418	7.149	0.132	1.718	0.418	7.055	0.129
Granisetron+DroperidolIV vs Dolasetron+DroperidolIV	1.588	0.381	6.615	0.152	1.584	0.392	6.406	0.132	1.579	0.393	6.345	0.129
**Design-by-treatment interaction model for inconsistency (χ^2, d.f., P-value, heterogeneity)**	**49.27 (44, 0.271, 0.151)**				**45.67 (41, 0.2843, 0.134)**				**50.95 (42, 0.162, 0.121)**			

Appendix 16. Nausea network meta-analysis results forest plot versus placebo*

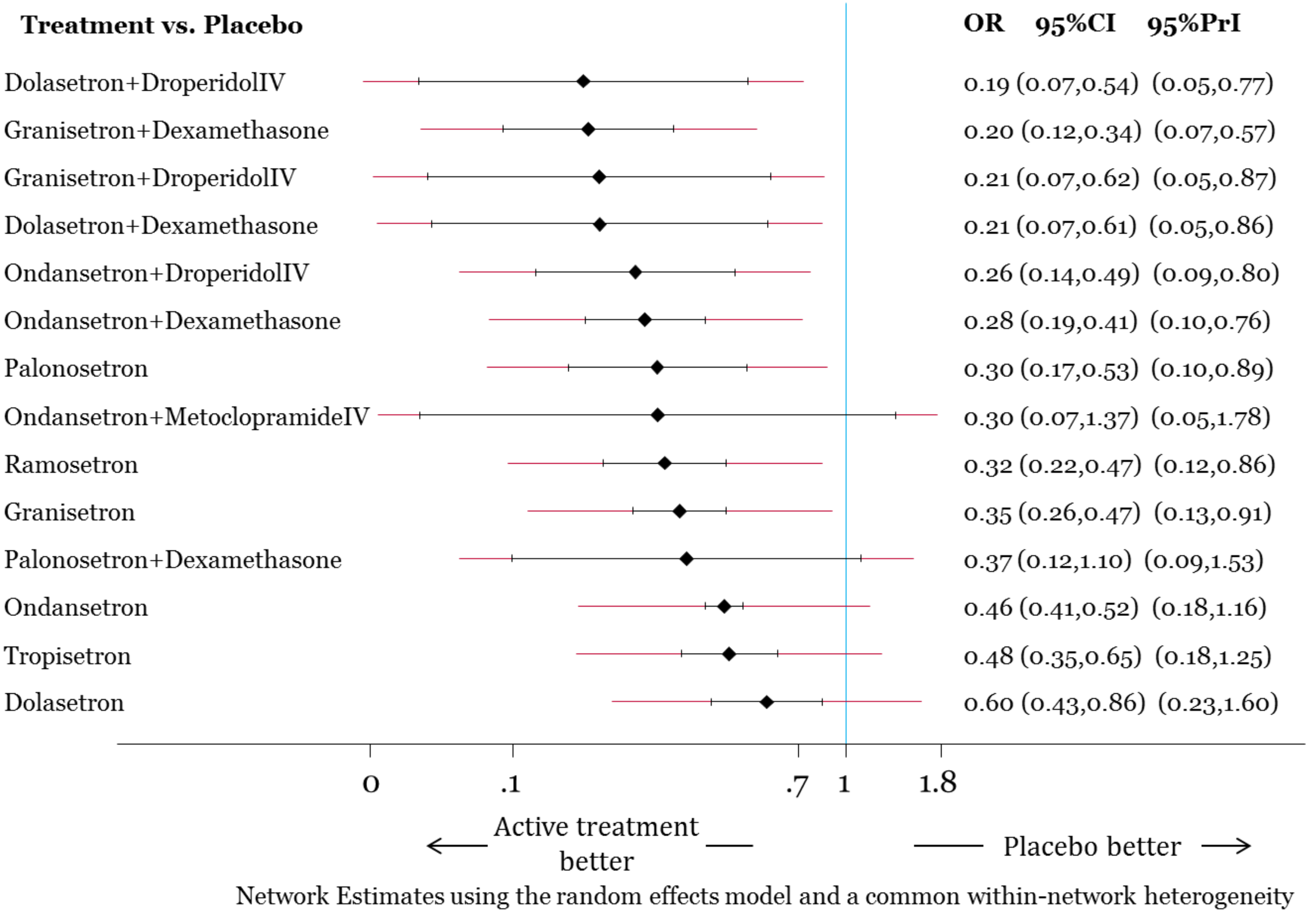

*Note: All treatments are compared to placebo. The black horizontal lines represent the 95% confidence intervals (CI) of the summary treatment effects and red horizontal lines the 95% predictive intervals (PrI). The results are presented on the odds ratio scale.

Appendix 17. Plot of the surface under the cumulative ranking (SUCRA) curves for the 15 treatments included in Nausea.

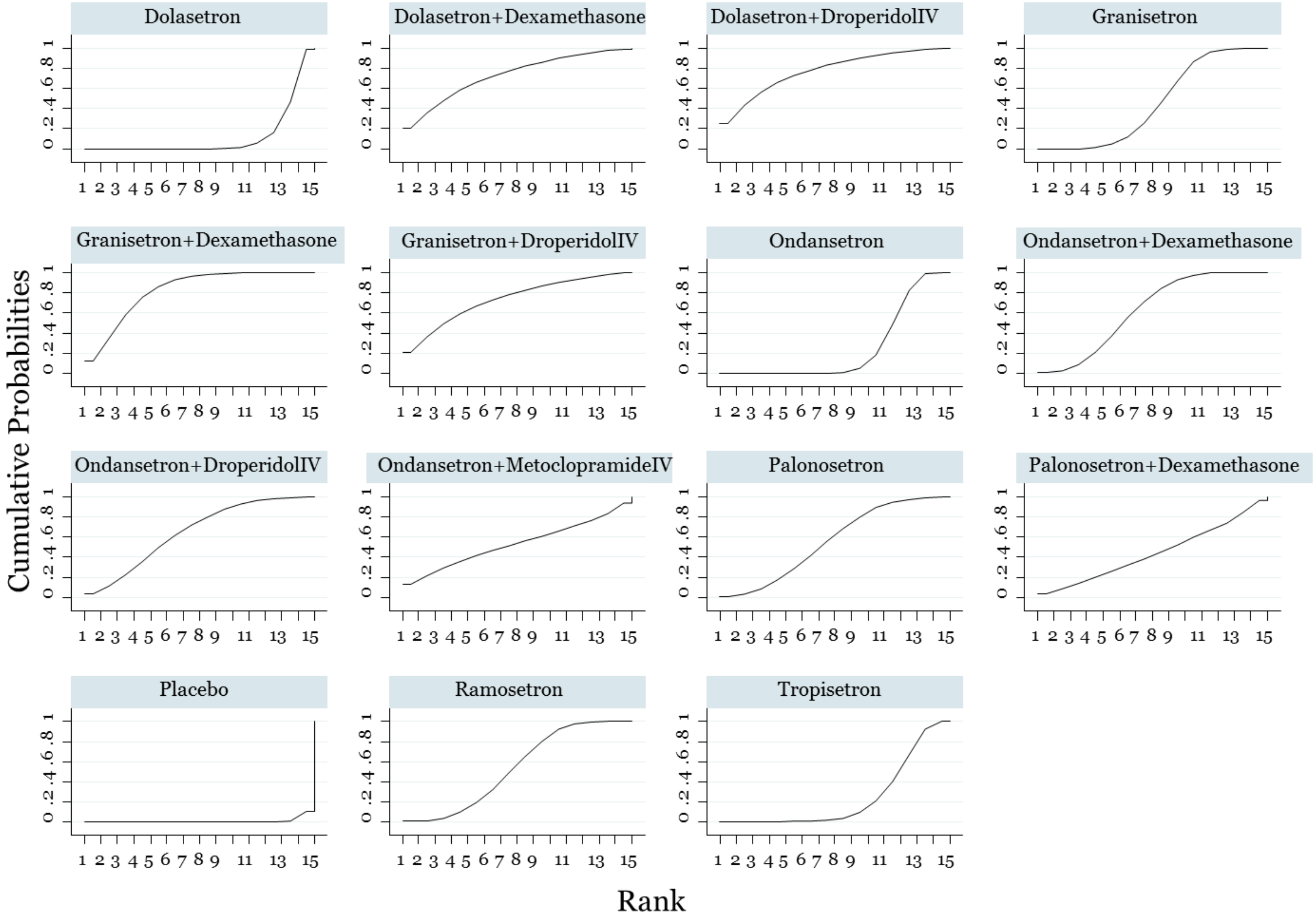

Appendix 18. Nausea sub-group analysis results

NAUSEA	*All time periods - all study designs*				*All time periods - RCTs only - sensitivity analysis for Risk of Bias*				*Intra-operative - RCTs only*			
**Treatment Comparison**	**OR**	**CI_ Low**	**CI_ High**	**Hetero geneity**	**OR**	**CI_ Low**	**CI_ High**	**Hetero geneity**	**OR**	**CI_ Low**	**CI_ High**	**Hetero geneity**
Ondansetron vs Placebo	0.455	0.402	0.514	0.240	0.451	0.398	0.511	0.214	0.444	0.391	0.506	0.218
Granisetron vs Placebo	0.345	0.254	0.467	0.240	0.353	0.260	0.479	0.214	0.347	0.257	0.467	0.218
Dolasetron vs Placebo	0.578	0.407	0.821	0.240	0.602	0.424	0.853	0.214	0.600	0.413	0.872	0.218
Tropisetron vs Placebo	0.475	0.347	0.650	0.240	0.499	0.360	0.692	0.214	0.451	0.324	0.628	0.218
Ondansetron+Dexamethasone vs Placebo	0.230	0.160	0.332	0.240	0.278	0.189	0.407	0.214	0.274	0.186	0.402	0.218
Palonosetron vs Placebo	0.298	0.166	0.534	0.240	0.302	0.172	0.531	0.214	0.300	0.170	0.529	0.218
Ramosetron vs Placebo	0.314	0.211	0.470	0.240	0.314	0.213	0.463	0.214	0.341	0.225	0.517	0.218
Ondansetron+DroperidolIV vs Placebo	0.251	0.132	0.476	0.240	0.259	0.138	0.486	0.214	0.238	0.122	0.465	0.218
Ondansetron+MetoclopramideIV vs Placebo	0.301	0.066	1.374	0.240	0.301	0.067	1.349	0.214	0.298	0.066	1.341	0.218
Granisetron+Dexamethasone vs Placebo	0.191	0.110	0.330	0.240	0.198	0.115	0.338	0.214	0.195	0.114	0.335	0.218
Palonosetron+Dexamethasone vs Placebo	0.359	0.115	1.117	0.240	0.363	0.121	1.092	0.214	0.361	0.119	1.089	0.218
Dolasetron+Dexamethasone vs Placebo	0.203	0.068	0.605	0.240	0.211	0.073	0.611	0.214	0.210	0.072	0.615	0.218
Dolasetron+DroperidolIV vs Placebo	0.187	0.064	0.549	0.240	0.190	0.067	0.540	0.214	0.190	0.067	0.543	0.218
Granisetron+DroperidolIV vs Placebo	0.208	0.069	0.624	0.240	0.211	0.071	0.624	0.214	0.209	0.071	0.620	0.218
Granisetron vs Ondansetron	0.758	0.553	1.040	0.240	0.782	0.569	1.076	0.214	0.780	0.571	1.064	0.218
Dolasetron vs Ondansetron	1.271	0.889	1.817	0.240	1.334	0.933	1.909	0.214	1.350	0.922	1.976	0.218
Tropisetron vs Ondansetron	1.045	0.753	1.449	0.240	1.107	0.786	1.561	0.214	1.015	0.720	1.431	0.218
Ondansetron+Dexamethasone vs Ondansetron	0.507	0.353	0.728	0.240	0.616	0.422	0.899	0.214	0.616	0.421	0.900	0.218
Palonosetron vs Ondansetron	0.656	0.364	1.181	0.240	0.670	0.379	1.185	0.214	0.675	0.381	1.198	0.218
Ramosetron vs Ondansetron	0.692	0.465	1.029	0.240	0.696	0.473	1.023	0.214	0.768	0.507	1.164	0.218

Ondansetron+DroperidolIV vs Ondansetron	0.551	0.291	1.043	0.240	0.575	0.309	1.071	0.214	0.535	0.275	1.042	0.218
Ondansetron+MetoclopramideIV vs Ondansetron	0.663	0.146	3.020	0.240	0.667	0.149	2.991	0.214	0.671	0.149	3.017	0.218
Granisetron+Dexamethasone vs Ondansetron	0.420	0.240	0.733	0.240	0.438	0.254	0.757	0.214	0.439	0.254	0.760	0.218
Palonosetron+Dexamethasone vs Ondansetron	0.790	0.253	2.464	0.240	0.805	0.267	2.428	0.214	0.812	0.268	2.459	0.218
Dolasetron+Dexamethasone vs Ondansetron	0.448	0.150	1.334	0.240	0.467	0.161	1.360	0.214	0.473	0.161	1.388	0.218
Dolasetron+DroperidolIV vs Ondansetron	0.411	0.139	1.213	0.240	0.422	0.148	1.204	0.214	0.428	0.149	1.229	0.218
Granisetron+DroperidolIV vs Ondansetron	0.456	0.151	1.380	0.240	0.467	0.157	1.391	0.214	0.471	0.158	1.403	0.218
Dolasetron vs Granisetron	1.676	1.057	2.659	0.240	1.705	1.074	2.706	0.214	1.731	1.077	2.783	0.218
Tropisetron vs Granisetron	1.378	0.893	2.126	0.240	1.415	0.906	2.211	0.214	1.301	0.837	2.023	0.218
Ondansetron+Dexamethasone vs Granisetron	0.668	0.420	1.064	0.240	0.787	0.488	1.270	0.214	0.789	0.491	1.269	0.218
Palonosetron vs Granisetron	0.865	0.456	1.641	0.240	0.857	0.459	1.600	0.214	0.866	0.464	1.616	0.218
Ramosetron vs Granisetron	0.912	0.559	1.488	0.240	0.889	0.549	1.438	0.214	0.985	0.599	1.618	0.218
Ondansetron+DroperidolIV vs Granisetron	0.727	0.359	1.472	0.240	0.735	0.368	1.469	0.214	0.686	0.331	1.421	0.218
Ondansetron+MetoclopramideIV vs Granisetron	0.874	0.186	4.101	0.240	0.852	0.184	3.938	0.214	0.861	0.186	3.979	0.218
Granisetron+Dexamethasone vs Granisetron	0.554	0.322	0.952	0.240	0.560	0.329	0.951	0.214	0.563	0.331	0.958	0.218
Palonosetron+Dexamethasone vs Granisetron	1.042	0.325	3.342	0.240	1.029	0.331	3.195	0.214	1.041	0.334	3.239	0.218
Dolasetron+Dexamethasone vs Granisetron	0.590	0.191	1.827	0.240	0.597	0.197	1.806	0.214	0.606	0.199	1.845	0.218
Dolasetron+DroperidolIV vs Granisetron	0.542	0.177	1.660	0.240	0.540	0.182	1.599	0.214	0.549	0.185	1.632	0.218
Granisetron+DroperidolIV vs Granisetron	0.602	0.199	1.822	0.240	0.597	0.200	1.780	0.214	0.603	0.202	1.800	0.218
Tropisetron vs Dolasetron	0.822	0.519	1.301	0.240	0.830	0.521	1.323	0.214	0.752	0.463	1.221	0.218
Ondansetron+Dexamethasone vs Dolasetron	0.399	0.242	0.657	0.240	0.462	0.276	0.771	0.214	0.456	0.268	0.775	0.218
Palonosetron vs Dolasetron	0.516	0.262	1.016	0.240	0.502	0.259	0.974	0.214	0.500	0.254	0.984	0.218

Ramosetron vs Dolasetron	0.544	0.321	0.922	0.240	0.521	0.311	0.875	0.214	0.569	0.327	0.990	0.218
Ondansetron+DroperidolIV vs Dolasetron	0.434	0.210	0.897	0.240	0.431	0.211	0.880	0.214	0.397	0.185	0.850	0.218
Ondansetron+MetoclopramideIV vs Dolasetron	0.522	0.110	2.470	0.240	0.500	0.107	2.330	0.214	0.497	0.106	2.336	0.218
Granisetron+Dexamethasone vs Dolasetron	0.330	0.172	0.633	0.240	0.328	0.173	0.623	0.214	0.325	0.169	0.626	0.218
Palonosetron+Dexamethasone vs Dolasetron	0.621	0.190	2.036	0.240	0.604	0.190	1.913	0.214	0.601	0.187	1.928	0.218
Dolasetron+Dexamethasone vs Dolasetron	0.352	0.123	1.007	0.240	0.350	0.126	0.976	0.214	0.350	0.125	0.980	0.218
Dolasetron+DroperidolIV vs Dolasetron	0.323	0.110	0.950	0.240	0.317	0.112	0.898	0.214	0.317	0.111	0.906	0.218
Granisetron+DroperidolIV vs Dolasetron	0.359	0.113	1.140	0.240	0.350	0.112	1.095	0.214	0.349	0.111	1.099	0.218
Ondansetron+Dexamethasone vs Tropisetron	0.485	0.301	0.782	0.240	0.556	0.337	0.917	0.214	0.607	0.367	1.002	0.218
Palonosetron vs Tropisetron	0.628	0.324	1.214	0.240	0.605	0.316	1.160	0.214	0.665	0.346	1.279	0.218
Ramosetron vs Tropisetron	0.662	0.399	1.098	0.240	0.628	0.379	1.041	0.214	0.757	0.447	1.282	0.218
Ondansetron+DroperidolIV vs Tropisetron	0.528	0.259	1.075	0.240	0.519	0.257	1.051	0.214	0.527	0.251	1.109	0.218
Ondansetron+MetoclopramideIV vs Tropisetron	0.635	0.135	2.983	0.240	0.602	0.130	2.796	0.214	0.661	0.142	3.078	0.218
Granisetron+Dexamethasone vs Tropisetron	0.402	0.214	0.755	0.240	0.395	0.211	0.741	0.214	0.433	0.231	0.813	0.218
Palonosetron+Dexamethasone vs Tropisetron	0.756	0.233	2.451	0.240	0.727	0.231	2.290	0.214	0.800	0.253	2.531	0.218
Dolasetron+Dexamethasone vs Tropisetron	0.428	0.138	1.325	0.240	0.422	0.139	1.279	0.214	0.466	0.152	1.425	0.218
Dolasetron+DroperidolIV vs Tropisetron	0.393	0.128	1.206	0.240	0.381	0.128	1.134	0.214	0.422	0.141	1.263	0.218
Granisetron+DroperidolIV vs Tropisetron	0.437	0.139	1.372	0.240	0.422	0.136	1.310	0.214	0.464	0.149	1.443	0.218
Palonosetron vs Ondansetron+Dexamethasone	1.294	0.652	2.567	0.240	1.088	0.553	2.140	0.214	1.097	0.556	2.163	0.218
Ramosetron vs Ondansetron+Dexamethasone	1.365	0.801	2.326	0.240	1.129	0.661	1.929	0.214	1.247	0.715	2.177	0.218
Ondansetron+DroperidolIV vs Ondansetron+Dexamethasone	1.088	0.541	2.186	0.240	0.934	0.469	1.859	0.214	0.869	0.422	1.793	0.218

Ondansetron+MetoclopramideIV vs Ondansetron+Dexamethasone	1.308	0.276	6.208	0.240	1.082	0.231	5.080	0.214	1.090	0.232	5.128	0.218
Granisetron+Dexamethasone vs Ondansetron+Dexamethasone	0.828	0.440	1.558	0.240	0.711	0.379	1.335	0.214	0.714	0.380	1.341	0.218
Palonosetron+Dexamethasone vs Ondansetron+Dexamethasone	1.559	0.474	5.125	0.240	1.307	0.409	4.179	0.214	1.318	0.410	4.234	0.218
Dolasetron+Dexamethasone vs Ondansetron+Dexamethasone	0.883	0.281	2.779	0.240	0.758	0.245	2.346	0.214	0.768	0.246	2.396	0.218
Dolasetron+DroperidolIV vs Ondansetron+Dexamethasone	0.811	0.260	2.526	0.240	0.686	0.226	2.080	0.214	0.695	0.228	2.122	0.218
Granisetron+DroperidolIV vs Ondansetron+Dexamethasone	0.901	0.284	2.857	0.240	0.759	0.242	2.383	0.214	0.764	0.243	2.406	0.218
Ramosetron vs Palonosetron	1.055	0.522	2.131	0.240	1.038	0.525	2.050	0.214	1.137	0.566	2.285	0.218
Ondansetron+DroperidolIV vs Palonosetron	0.841	0.355	1.993	0.240	0.858	0.371	1.985	0.214	0.793	0.331	1.897	0.218
Ondansetron+MetoclopramideIV vs Palonosetron	1.011	0.199	5.126	0.240	0.995	0.200	4.937	0.214	0.994	0.200	4.947	0.218
Granisetron+Dexamethasone vs Palonosetron	0.640	0.290	1.415	0.240	0.653	0.302	1.414	0.214	0.651	0.300	1.411	0.218
Palonosetron+Dexamethasone vs Palonosetron	1.204	0.455	3.191	0.240	1.201	0.467	3.092	0.214	1.202	0.465	3.105	0.218
Dolasetron+Dexamethasone vs Palonosetron	0.683	0.199	2.345	0.240	0.697	0.209	2.323	0.214	0.700	0.208	2.355	0.218
Dolasetron+DroperidolIV vs Palonosetron	0.626	0.184	2.132	0.240	0.630	0.193	2.061	0.214	0.634	0.192	2.087	0.218
Granisetron+DroperidolIV vs Palonosetron	0.696	0.201	2.411	0.240	0.697	0.206	2.360	0.214	0.697	0.205	2.365	0.218
Ondansetron+DroperidolIV vs Ramosetron	0.797	0.377	1.685	0.240	0.827	0.399	1.714	0.214	0.697	0.319	1.522	0.218
Ondansetron+MetoclopramideIV vs Ramosetron	0.958	0.200	4.587	0.240	0.958	0.204	4.506	0.214	0.874	0.184	4.146	0.218
Granisetron+Dexamethasone vs Ramosetron	0.607	0.309	1.190	0.240	0.630	0.326	1.215	0.214	0.572	0.292	1.122	0.218
Palonosetron+Dexamethasone vs Ramosetron	1.142	0.344	3.795	0.240	1.157	0.361	3.710	0.214	1.057	0.325	3.432	0.218
Dolasetron+Dexamethasone vs Ramosetron	0.647	0.203	2.060	0.240	0.672	0.217	2.082	0.214	0.616	0.195	1.942	0.218

Dolasetron+DroperidolIV vs Ramosetron	0.594	0.188	1.873	0.240	0.607	0.200	1.846	0.214	0.557	0.181	1.720	0.218
Granisetron+DroperidolIV vs Ramosetron	0.660	0.205	2.124	0.240	0.672	0.213	2.122	0.214	0.613	0.192	1.956	0.218
Ondansetron+MetoclopramideIV vs Ondansetron+DroperidolIV	1.203	0.232	6.222	0.240	1.159	0.229	5.879	0.214	1.254	0.243	6.479	0.218
Granisetron+Dexamethasone vs Ondansetron+DroperidolIV	0.761	0.329	1.761	0.240	0.762	0.336	1.729	0.214	0.821	0.350	1.925	0.218
Palonosetron+Dexamethasone vs Ondansetron+DroperidolIV	1.433	0.390	5.263	0.240	1.400	0.396	4.955	0.214	1.516	0.418	5.503	0.218
Dolasetron+Dexamethasone vs Ondansetron+DroperidolIV	0.812	0.230	2.867	0.240	0.812	0.237	2.789	0.214	0.883	0.250	3.124	0.218
Dolasetron+DroperidolIV vs Ondansetron+DroperidolIV	0.745	0.213	2.609	0.240	0.735	0.218	2.477	0.214	0.800	0.231	2.773	0.218
Granisetron+DroperidolIV vs Ondansetron+DroperidolIV	0.828	0.232	2.956	0.240	0.813	0.233	2.841	0.214	0.879	0.246	3.145	0.218
Granisetron+Dexamethasone vs Ondansetron+MetoclopramideIV	0.633	0.126	3.174	0.240	0.657	0.133	3.234	0.214	0.655	0.133	3.229	0.218
Palonosetron+Dexamethasone vs Ondansetron+MetoclopramideIV	1.191	0.179	7.909	0.240	1.208	0.188	7.761	0.214	1.209	0.187	7.803	0.218
Dolasetron+Dexamethasone vs Ondansetron+MetoclopramideIV	0.675	0.104	4.362	0.240	0.701	0.111	4.410	0.214	0.705	0.111	4.463	0.218
Dolasetron+DroperidolIV vs Ondansetron+MetoclopramideIV	0.620	0.096	3.980	0.240	0.634	0.102	3.939	0.214	0.638	0.102	3.986	0.218
Granisetron+DroperidolIV vs Ondansetron+MetoclopramideIV	0.688	0.106	4.484	0.240	0.701	0.110	4.468	0.214	0.701	0.110	4.479	0.218
Palonosetron+Dexamethasone vs Granisetron+Dexamethasone	1.882	0.536	6.608	0.240	1.838	0.543	6.228	0.214	1.847	0.543	6.284	0.218
Dolasetron+Dexamethasone vs Granisetron+Dexamethasone	1.066	0.315	3.608	0.240	1.067	0.324	3.514	0.214	1.076	0.324	3.573	0.218
Dolasetron+DroperidolIV vs Granisetron+Dexamethasone	0.979	0.292	3.280	0.240	0.965	0.299	3.116	0.214	0.974	0.300	3.166	0.218
Granisetron+DroperidolIV vs Granisetron+Dexamethasone	1.088	0.346	3.422	0.240	1.067	0.345	3.300	0.214	1.071	0.346	3.319	0.218
Dolasetron+Dexamethasone vs Palonosetron+Dexamethasone	0.567	0.118	2.729	0.240	0.580	0.126	2.681	0.214	0.583	0.125	2.717	0.218
Dolasetron+DroperidolIV vs Palonosetron+Dexamethasone	0.520	0.109	2.487	0.240	0.525	0.115	2.388	0.214	0.527	0.115	2.420	0.218

Granisetron+DroperidolIV vs Palonosetron+Dexamethasone	0.578	0.119	2.802	0.240	0.581	0.124	2.715	0.214	0.580	0.123	2.724	0.218
Dolasetron+DroperidolIV vs Dolasetron+Dexamethasone	0.918	0.205	4.108	0.240	0.904	0.211	3.877	0.214	0.905	0.210	3.906	0.218
Granisetron+DroperidolIV vs Dolasetron+Dexamethasone	1.020	0.217	4.796	0.240	1.001	0.219	4.574	0.214	0.995	0.216	4.583	0.218
Granisetron+DroperidolIV vs Dolasetron+DroperidolIV	1.111	0.238	5.188	0.240	1.107	0.246	4.981	0.214	1.099	0.243	4.978	0.218
**Design-by-treatment interaction model for inconsistency (χ^2, d.f., P-value, heterogeneity)**	**26.65 (41, 0.959, 0.271)**				**22.61 (37, 0.970, 0.260)**				**24.38 (41, 0.982, 0.271)**			

Appendix 19. PONV network meta-analysis results forest plot versus placebo*

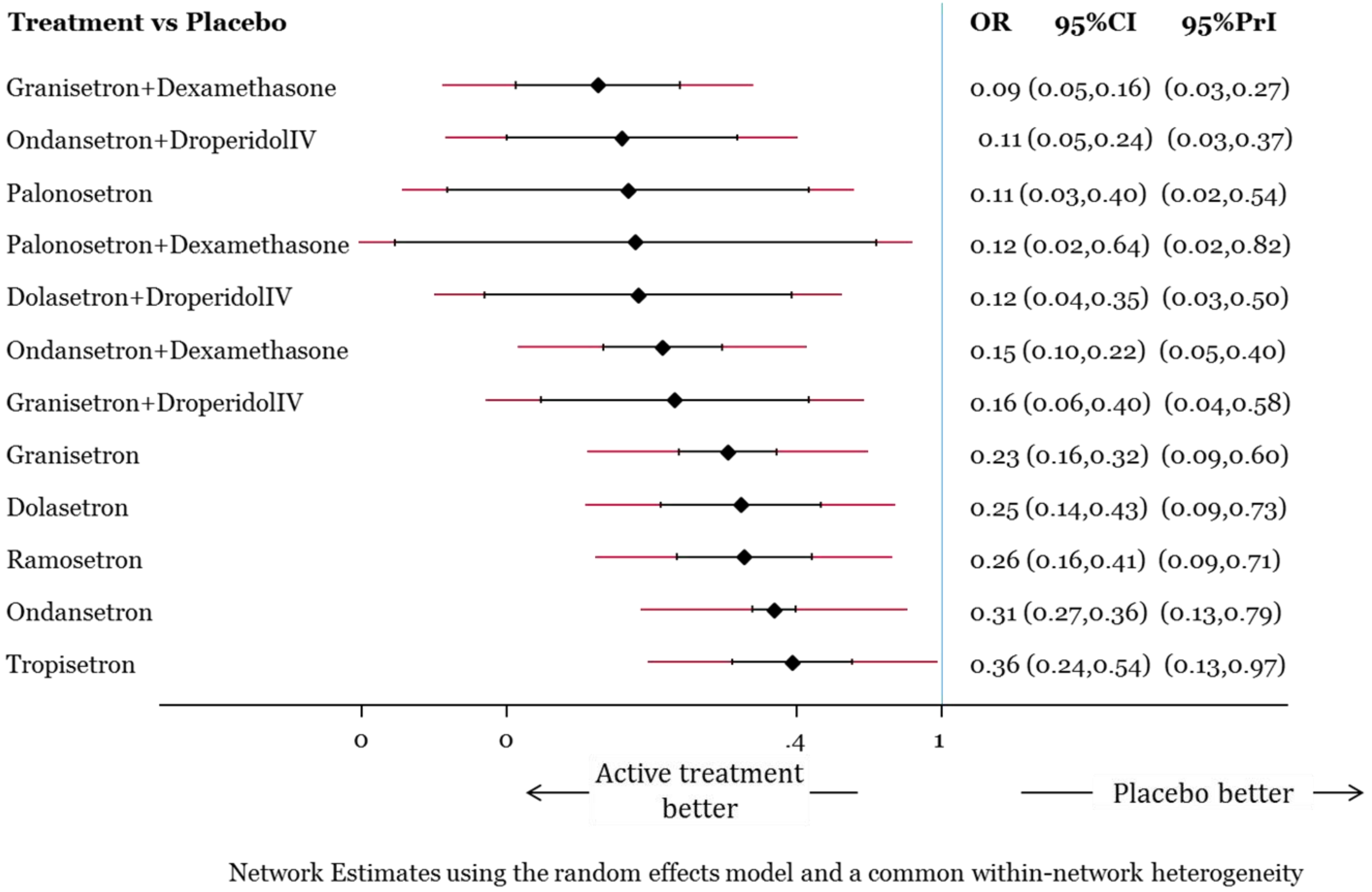

*Note: All treatments are compared to placebo. The black horizontal lines represent the 95% confidence intervals (CI) of the summary treatment effects and red horizontal lines the 95% predictive intervals (PrI). The results are presented on the odds ratio scale.

Appendix 20. Plot of the surface under the cumulative ranking (SUCRA) curves for the 13 treatments included in PONV.

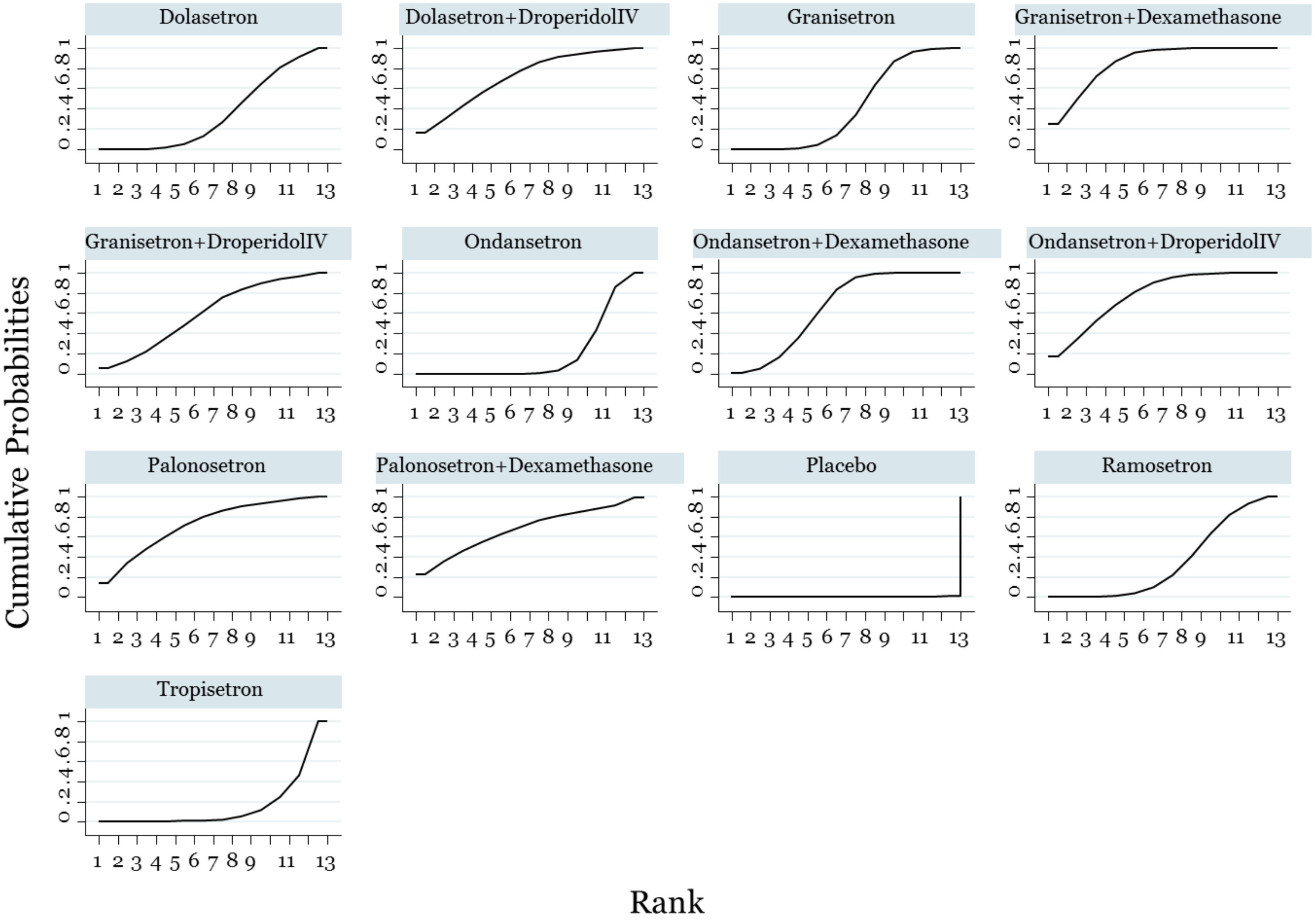

Appendix 21. PONV sub-group analysis results

PONV	*All time periods - all study designs*				*All time periods - RCTs only - sensitivity analysis for Risk of Bias*				*Intra-operative - RCTs only*			
**Treatment Comparison**	**OR**	**CI_ Low**	**CI_ High**	**Hetero geneity**	**OR**	**CI_ Low**	**CI_ High**	**Hetero geneity**	**OR**	**CI_ Low**	**CI_ High**	**Hetero geneity**
Ondansetron vs Placebo	0.321	0.277	0.372	0.250	0.311	0.266	0.363	0.254	0.310	0.265	0.362	0.226
Granisetron vs Placebo	0.229	0.162	0.324	0.250	0.222	0.156	0.317	0.254	0.240	0.169	0.340	0.226
Dolasetron vs Placebo	0.246	0.144	0.421	0.250	0.242	0.134	0.436	0.254	0.248	0.141	0.436	0.226
Tropisetron vs Placebo	0.359	0.233	0.554	0.250	0.398	0.249	0.633	0.254	0.349	0.222	0.550	0.226
Ondansetron+Dexamethasone vs Placebo	0.160	0.108	0.236	0.250	0.157	0.106	0.232	0.254	0.148	0.097	0.228	0.226
Palonosetron vs Placebo	0.117	0.032	0.435	0.250	0.114	0.030	0.424	0.254	0.113	0.032	0.405	0.226
Ramosetron vs Placebo	0.259	0.158	0.423	0.250	0.254	0.155	0.417	0.254	0.300	0.184	0.488	0.226
Ondansetron+DroperidolIV vs Placebo	0.114	0.050	0.260	0.250	0.112	0.049	0.256	0.254	0.110	0.049	0.247	0.226
Granisetron+Dexamethasone vs Placebo	0.092	0.051	0.166	0.250	0.090	0.050	0.163	0.254	0.100	0.054	0.184	0.226
Palonosetron+Dexamethasone vs Placebo	0.125	0.022	0.714	0.250	0.121	0.021	0.698	0.254	0.120	0.022	0.652	0.226
Dolasetron+DroperidolIV vs Placebo	0.122	0.040	0.371	0.250	0.121	0.040	0.372	0.254	0.123	0.042	0.362	0.226
Granisetron+DroperidolIV vs Placebo	0.158	0.061	0.409	0.250	0.155	0.060	0.404	0.254	0.165	0.064	0.420	0.226
Granisetron vs Ondansetron	0.713	0.498	1.020	0.250	0.715	0.495	1.031	0.254	0.774	0.537	1.114	0.226
Dolasetron vs Ondansetron	0.767	0.446	1.318	0.250	0.778	0.430	1.410	0.254	0.801	0.452	1.419	0.226
Tropisetron vs Ondansetron	1.119	0.714	1.753	0.250	1.279	0.785	2.083	0.254	1.128	0.705	1.804	0.226
Ondansetron+Dexamethasone vs Ondansetron	0.498	0.338	0.734	0.250	0.505	0.342	0.745	0.254	0.480	0.315	0.730	0.226
Palonosetron vs Ondansetron	0.365	0.099	1.343	0.250	0.365	0.099	1.351	0.254	0.365	0.103	1.296	0.226
Ramosetron vs Ondansetron	0.806	0.493	1.318	0.250	0.818	0.499	1.341	0.254	0.969	0.593	1.583	0.226
Ondansetron+DroperidolIV vs Ondansetron	0.355	0.156	0.807	0.250	0.359	0.157	0.819	0.254	0.355	0.159	0.793	0.226

Granisetron+Dexamethasone vs Ondansetron	0.288	0.159	0.523	0.250	0.291	0.160	0.530	0.254	0.324	0.174	0.602	0.226
Palonosetron+Dexamethasone vs Ondansetron	0.389	0.069	2.210	0.250	0.390	0.068	2.229	0.254	0.387	0.072	2.092	0.226
Dolasetron+DroperidolIV vs Ondansetron	0.381	0.125	1.162	0.250	0.390	0.126	1.204	0.254	0.397	0.133	1.178	0.226
Granisetron+DroperidolIV vs Ondansetron	0.492	0.189	1.285	0.250	0.500	0.191	1.310	0.254	0.532	0.206	1.369	0.226
Dolasetron vs Granisetron	1.075	0.570	2.030	0.250	1.089	0.550	2.157	0.254	1.035	0.535	2.003	0.226
Tropisetron vs Granisetron	1.570	0.912	2.702	0.250	1.789	1.007	3.179	0.254	1.457	0.832	2.554	0.226
Ondansetron+Dexamethasone vs Granisetron	0.699	0.420	1.162	0.250	0.706	0.422	1.181	0.254	0.620	0.359	1.068	0.226
Palonosetron vs Granisetron	0.513	0.133	1.978	0.250	0.511	0.132	1.988	0.254	0.472	0.127	1.763	0.226
Ramosetron vs Granisetron	1.130	0.633	2.018	0.250	1.144	0.637	2.055	0.254	1.252	0.703	2.229	0.226
Ondansetron+DroperidolIV vs Granisetron	0.498	0.204	1.212	0.250	0.502	0.205	1.230	0.254	0.458	0.191	1.101	0.226
Granisetron+Dexamethasone vs Granisetron	0.404	0.235	0.696	0.250	0.407	0.236	0.702	0.254	0.418	0.240	0.729	0.226
Palonosetron+Dexamethasone vs Granisetron	0.546	0.093	3.216	0.250	0.545	0.092	3.241	0.254	0.500	0.089	2.812	0.226
Dolasetron+DroperidolIV vs Granisetron	0.534	0.167	1.707	0.250	0.546	0.169	1.766	0.254	0.512	0.165	1.595	0.226
Granisetron+DroperidolIV vs Granisetron	0.691	0.268	1.782	0.250	0.700	0.270	1.811	0.254	0.687	0.271	1.743	0.226
Tropisetron vs Dolasetron	1.459	0.733	2.905	0.250	1.643	0.775	3.482	0.254	1.408	0.684	2.901	0.226
Ondansetron+Dexamethasone vs Dolasetron	0.650	0.337	1.253	0.250	0.648	0.322	1.306	0.254	0.599	0.297	1.208	0.226
Palonosetron vs Dolasetron	0.477	0.116	1.953	0.250	0.470	0.112	1.974	0.254	0.456	0.114	1.831	0.226
Ramosetron vs Dolasetron	1.051	0.510	2.165	0.250	1.051	0.490	2.254	0.254	1.210	0.577	2.537	0.226
Ondansetron+DroperidolIV vs Dolasetron	0.463	0.174	1.233	0.250	0.461	0.168	1.268	0.254	0.443	0.166	1.181	0.226
Granisetron+Dexamethasone vs Dolasetron	0.376	0.170	0.830	0.250	0.374	0.163	0.858	0.254	0.404	0.177	0.925	0.226
Palonosetron+Dexamethasone vs Dolasetron	0.508	0.082	3.131	0.250	0.501	0.079	3.160	0.254	0.483	0.081	2.872	0.226

Dolasetron+DroperidolIV vs Dolasetron	0.497	0.164	1.509	0.250	0.501	0.163	1.539	0.254	0.495	0.168	1.462	0.226
Granisetron+DroperidolIV vs Dolasetron	0.642	0.216	1.913	0.250	0.643	0.209	1.971	0.254	0.664	0.223	1.979	0.226
Ondansetron+Dexamethasone vs Tropisetron	0.445	0.249	0.795	0.250	0.395	0.215	0.725	0.254	0.425	0.229	0.790	0.226
Palonosetron vs Tropisetron	0.327	0.082	1.295	0.250	0.286	0.071	1.154	0.254	0.324	0.084	1.251	0.226
Ramosetron vs Tropisetron	0.720	0.375	1.382	0.250	0.639	0.325	1.259	0.254	0.859	0.443	1.665	0.226
Ondansetron+DroperidolIV vs Tropisetron	0.317	0.125	0.804	0.250	0.281	0.109	0.726	0.254	0.314	0.125	0.792	0.226
Granisetron+Dexamethasone vs Tropisetron	0.258	0.125	0.531	0.250	0.227	0.108	0.480	0.254	0.287	0.135	0.610	0.226
Palonosetron+Dexamethasone vs Tropisetron	0.348	0.058	2.091	0.250	0.305	0.050	1.863	0.254	0.343	0.060	1.978	0.226
Dolasetron+DroperidolIV vs Tropisetron	0.340	0.104	1.120	0.250	0.305	0.091	1.027	0.254	0.352	0.109	1.135	0.226
Granisetron+DroperidolIV vs Tropisetron	0.440	0.155	1.248	0.250	0.391	0.135	1.129	0.254	0.471	0.167	1.331	0.226
Palonosetron vs Ondansetron+Dexamethasone	0.734	0.189	2.854	0.250	0.724	0.185	2.834	0.254	0.762	0.201	2.893	0.226
Ramosetron vs Ondansetron+Dexamethasone	1.618	0.872	3.001	0.250	1.620	0.871	3.014	0.254	2.020	1.067	3.824	0.226
Ondansetron+DroperidolIV vs Ondansetron+Dexamethasone	0.712	0.300	1.693	0.250	0.711	0.298	1.695	0.254	0.739	0.314	1.741	0.226
Granisetron+Dexamethasone vs Ondansetron+Dexamethasone	0.578	0.290	1.155	0.250	0.576	0.287	1.155	0.254	0.675	0.322	1.414	0.226
Palonosetron+Dexamethasone vs Ondansetron+Dexamethasone	0.782	0.132	4.633	0.250	0.772	0.129	4.613	0.254	0.807	0.142	4.596	0.226
Dolasetron+DroperidolIV vs Ondansetron+Dexamethasone	0.765	0.236	2.474	0.250	0.773	0.236	2.529	0.254	0.827	0.259	2.640	0.226
Granisetron+DroperidolIV vs Ondansetron+Dexamethasone	0.988	0.355	2.751	0.250	0.991	0.355	2.769	0.254	1.108	0.397	3.097	0.226
Ramosetron vs Palonosetron	2.205	0.548	8.867	0.250	2.238	0.553	9.055	0.254	2.651	0.682	10.305	0.226
Ondansetron+DroperidolIV vs Palonosetron	0.971	0.208	4.527	0.250	0.982	0.209	4.609	0.254	0.970	0.216	4.347	0.226

Granisetron+Dexamethasone vs Palonosetron	0.788	0.188	3.300	0.250	0.796	0.189	3.354	0.254	0.886	0.216	3.627	0.226
Palonosetron+Dexamethasone vs Palonosetron	1.065	0.338	3.361	0.250	1.067	0.336	3.381	0.254	1.059	0.347	3.233	0.226
Dolasetron+DroperidolIV vs Palonosetron	1.042	0.188	5.789	0.250	1.067	0.190	5.998	0.254	1.085	0.204	5.763	0.226
Granisetron+DroperidolIV vs Palonosetron	1.347	0.267	6.787	0.250	1.368	0.270	6.942	0.254	1.454	0.299	7.062	0.226
Ondansetron+DroperidolIV vs Ramosetron	0.440	0.170	1.143	0.250	0.439	0.168	1.143	0.254	0.366	0.143	0.934	0.226
Granisetron+Dexamethasone vs Ramosetron	0.358	0.168	0.759	0.250	0.356	0.167	0.758	0.254	0.334	0.155	0.719	0.226
Palonosetron+Dexamethasone vs Ramosetron	0.483	0.080	2.937	0.250	0.477	0.078	2.919	0.254	0.399	0.069	2.316	0.226
Dolasetron+DroperidolIV vs Ramosetron	0.473	0.141	1.588	0.250	0.477	0.140	1.621	0.254	0.409	0.125	1.337	0.226
Granisetron+DroperidolIV vs Ramosetron	0.611	0.211	1.772	0.250	0.611	0.210	1.780	0.254	0.549	0.192	1.566	0.226
Granisetron+Dexamethasone vs Ondansetron+DroperidolIV	0.812	0.296	2.225	0.250	0.810	0.294	2.231	0.254	0.913	0.333	2.506	0.226
Palonosetron+Dexamethasone vs Ondansetron+DroperidolIV	1.097	0.161	7.493	0.250	1.086	0.158	7.475	0.254	1.091	0.168	7.079	0.226
Dolasetron+DroperidolIV vs Ondansetron+DroperidolIV	1.074	0.270	4.272	0.250	1.087	0.270	4.372	0.254	1.119	0.290	4.309	0.226
Granisetron+DroperidolIV vs Ondansetron+DroperidolIV	1.388	0.395	4.880	0.250	1.393	0.394	4.923	0.254	1.499	0.436	5.160	0.226
Palonosetron+Dexamethasone vs Granisetron+Dexamethasone	1.352	0.216	8.476	0.250	1.340	0.212	8.478	0.254	1.196	0.198	7.224	0.226
Dolasetron+DroperidolIV vs Granisetron+Dexamethasone	1.322	0.377	4.634	0.250	1.341	0.378	4.757	0.254	1.225	0.354	4.236	0.226
Granisetron+DroperidolIV vs Granisetron+Dexamethasone	1.709	0.631	4.626	0.250	1.719	0.633	4.669	0.254	1.642	0.613	4.400	0.226
Dolasetron+DroperidolIV vs Palonosetron+Dexamethasone	0.978	0.124	7.704	0.250	1.001	0.125	7.981	0.254	1.025	0.138	7.638	0.226

Granisetron+DroperidolIV vs Palonosetron+Dexamethasone	1.264	0.174	9.192	0.250	1.283	0.175	9.407	0.254	1.374	0.198	9.511	0.226
Granisetron+DroperidolIV vs Dolasetron+DroperidolIV	1.292	0.300	5.573	0.250	1.282	0.294	5.587	0.254	1.340	0.321	5.602	0.226
**Design-by-treatment interaction model for inconsistency (χ^2, d.f., P-value, heterogeneity)**	**26.58 (32, 0.737, 0.263)**				**23.05 (29, 0.774, 0.269)**				**22.53 (27, 0.710, 0.241)**			

Appendix 22. Included studies in our review versus previous systematic reviews

Study	Tang, 2012 (NMA)	Carlisle, 2006 (Cochrane)	Rawlinson, 2012	# of patients
Randomized clinical trials (n=429)				
Browning, 2013	NO	NO	NO	118
Aleyasin, 2012	NO	NO	NO	120
Blitz, 2012	NO	NO	NO	118
Choi, 2012	NO	NO	NO	120
de Orange, 2012	NO	NO	NO	129
Eidi, 2012	NO	NO	NO	219
Gurha, 2012[†]	NO	NO	NO	90
Koh, 2012	NO	NO	NO	119
Lahsaei, 2012	NO	NO	NO	300
Madenoglu, 2012	NO	NO	NO	90
Moon, 2012	NO	NO	NO	100
Park JW, 2012	NO	NO	NO	84
Wang, 2012	NO	NO	NO	130
Ahmed, 2011	NO	NO	NO	80
Apfel, 2011[†]	NO	NO	NO	98
Asl, 2011	NO	NO	NO	90
Bajwa, 2011	NO	NO	NO	60
Basu, 2011	NO	NO	NO	75
Bhattarai, 2011	NO	NO	NO	100
Bicer, 2011	NO	NO	NO	150
D'Souza, 2011	NO	NO	NO	92
Ekinci, 2011	NO	NO	NO	100
El-deeb, 2011a	NO	NO	NO	80
El-deeb, 2011b	NO	NO	NO	160
El-deeb, 2011c	NO	NO	NO	300
Gan, 2011	NO	NO	NO	619
Islam, 2011	NO	NO	NO	100
Khan, 2011	NO	NO	NO	60
Lee, 2011	NO	NO	NO	120
Metaxari, 2011	NO	NO	NO	203
Park, 2011	NO	NO	NO	90
Ryu, 2011	NO	NO	NO	124
Safae, 2011	NO	NO	NO	64
Sahoo, 2011	NO	NO	NO	52
Singh, 2011	NO	NO	NO	50
Song, 2011	NO	NO	NO	123
Srivastava, 2011	NO	NO	NO	75
Talesh, 2011	NO	NO	NO	100
Vale, 2011	NO	NO	NO	40
Akhtar, 2010	NO	NO	NO	130
Alghanem, 2010	NO	NO	NO	180
Bhattachajee, 2010	NO	NO	NO	60
Bilgin, 2010	NO	NO	NO	160

Choi, 2010	NO	NO	NO	279
Dabbous, 2010	NO	NO	NO	84
Ebrahim Soltani, 2010 (CR: Ebrahim Soltani, 2011)	NO	NO	NO	150
Hahm, 2010	NO	NO	NO	84
Huh, 2010	NO	NO	NO	60
Jee, 2010	NO	NO	NO	100
Jokela, 2010	NO	NO	NO	80
Mishra, 2010	NO	NO	NO	40
Omar, 2010	NO	NO	NO	40
Rauers, 2010	NO	NO	NO	179
Ryu, 2010	NO	NO	NO	120
Shakya, 2010	NO	NO	NO	80
Singla, 2010	NO	NO	NO	702
Tan, 2010	YES	NO	NO	80
Yun, 2010	NO	NO	NO	98
Farouk, 2009	NO	NO	NO	30
Feng, 2009	NO	NO	NO	192
Gan, 2009	NO	NO	NO	138
Gandhi, 2009	NO	NO	NO	150
Grover, 2009	YES	NO	NO	103
Jain, 2009	NO	NO	NO	87
Jellish, 2009	NO	NO	NO	100
Kim, 2009	YES	NO	NO	162
Riad, 2009	NO	NO	NO	100
Rosow, 2009	NO	NO	NO	244
Aghadavoudi, 2008	YES	NO	NO	120
Bano, 2008	NO	NO	NO	97
Bhatia, 2008	NO	NO	NO	120
Candiotti, 2008	NO	NO	NO	546
Choi, 2008	NO	NO	NO	94
Contreras-Dominguez, 2008	YES	NO	NO	150
Cruz, 2008	NO	NO	NO	372
Davis, 2008	NO	NO	NO	200
Erhan, 2008	YES	NO	NO	80
Gautam, 2008	NO	NO	NO	142
Gupta, 2008	NO	NO	NO	60
Kaki, 2008	YES	NO	NO	75
Kovac, 2008	NO	NO	NO	544
Krobbuaban, 2008	NO	NO	NO	380
Lee, 2008	NO	NO	NO	236
Ng, 2008	NO	NO	NO	81
Olatosi, 2008	NO	NO	NO	75
Pan, 2008	NO	NO	NO	60
Piper, 2008	YES	NO	NO	83
Said-Ahmed, 2008	NO	NO	NO	50
Sajedi, 2008	NO	NO	NO	66
Sakellaris, 2008	NO	NO	NO	110

Schnaider, 2008	NO	NO	NO	70
Aouad, 2007	NO	NO	NO	89
Balki, 2007	NO	NO	NO	176
Bestas, 2007	YES	NO	NO	90
Bhatagnar, 2007	NO	NO	NO	90
Bolton, 2007	NO	NO	NO	557
Boonmak, 2007	NO	NO	NO	160
Candiotti, 2007	NO	NO	NO	88
Diemunsch, 2007	NO	NO	NO	892
Gan, 2007	NO	NO	NO	766
Gombar, 2007	NO	NO	NO	90
Gulhas, 2007	NO	NO	NO	72
Han, 2007	NO	NO	NO	80
Harnett, 2007	NO	NO	NO	240
Kushwaha, 2007	NO	NO	NO	125
Lee, 2007	NO	NO	NO	86
Moussa, 2007	YES	NO	NO	120
Oksuz, 2007	NO	NO	NO	75
Paech, 2007	NO	NO	NO	614
Rusch, 2007	NO	NO	NO	228
Sagir, 2007	NO	NO	NO	80
Sandhu, 2007	NO	NO	NO	80
Sarkar, 2007	NO	NO	NO	60
Sharma, 2007	NO	NO	NO	40
Siddik-Sayyid, 2007	NO	NO	NO	129
Sodhi, 2007	NO	NO	NO	90
Sriraman, 2007	NO	NO	NO	100
Subramaniam, 2007	NO	NO	NO	75
Teran, 2007	NO	NO	NO	35
White, 2007	NO	NO	NO	66
Wig, 2007	NO	NO	NO	70
Akin, 2006	YES	NO	NO	70
Birmingham, 2006	NO	NO	NO	100
Bridge, 2006	NO	NO	NO	194
Chan, 2006	NO	NO	NO	394
Eberhart, 2006	YES	NO	NO	310
Gunter, 2006	NO	NO	NO	240
Gupta, 2006	NO	NO	NO	200
Isik, 2006	NO	NO	NO	60
Janicki, 2006	NO	NO	NO	150
Jellish, 2006	NO	NO	NO	120
Johns, 2006	NO	NO	NO	960
Khan, 2006	NO	NO	NO	180
Kolm, 2006	NO	NO	NO	100
Leksowski, 2006	NO	NO	NO	210
Paranhos de Abreu, 2006	NO	NO	NO	100
Peixoto, 2006	YES	NO	NO	120
Purhonen, 2006a	YES	NO	NO	56

Purhonen, 2000b	NO	NO	NO	100
Sarvela, 2006	NO	NO	NO	87
Tosun, 2006	NO	YES	NO	125
Tseng, 2006	NO	NO	NO	140
Veneziani, 2006	NO	NO	NO	100
White, 2006	NO	NO	NO	205
Cohen, 2005	NO	NO	NO	62
D'Angelo, 2005	YES	NO	NO	121
Demiraran, 2005	NO	NO	NO	90
Gan, 2005	NO	NO	NO	176
Hartsell, 2005	NO	NO	NO	60
Iatrou, 2005	NO	NO	NO	105
Kabalak, 2005	NO	NO	NO	60
Khan, 2005	NO	NO	NO	60
Kocamanoglu, 2005	YES	NO	NO	150
Kontrimaviciute, 2005	NO	NO	NO	34
Misra, 2005	YES	NO	NO	79
Pirat, 2005	YES	NO	NO	150
Treschan, 2005	NO	NO	NO	142
White, 2005[†]	NO	NO	NO	372
Alkaissi, 2004	NO	NO	NO	495
Apfel, 2004 (CR: Jokela, 2009)	NO	YES	YES	260
Barbone, 2004	NO	NO	NO	141
Barbosa, 2004	NO	NO	NO	60
Bhardwaj, 2004	NO	NO	NO	100
Binstock, 2004	NO	NO	NO	101
Browning, 2004	NO	NO	NO	212
Celiker, 2004	NO	NO	NO	102
Dua, 2004	YES	NO	NO	60
Eberhart, 2004	NO	NO	NO	304
Gan, 2004	YES	NO	NO	49
Han, 2004	NO	NO	NO	350
Hanaoka, 2004	YES	NO	NO	315
Meyer, 2004	NO	NO	NO	92
Panda, 2004	NO	NO	NO	100
Samakarndi, 2004	NO	NO	NO	80
Sinha, 2004	YES	NO	NO	70
Bharti, 2003	NO	YES	NO	120
Biswas, 2003a	YES	NO	NO	160
Biswas, 2003b	NO	YES	NO	120
Burmeister, 2003	NO	YES	NO	40
Charuluxananan, 2003	NO	YES	NO	180
Karamanlioglu, 2003	NO	YES	NO	150
Loewen, 2003	NO	YES	NO	71
Maddali, 2003	NO	NO	NO	120
Madenoglu, 2003	YES	YES	NO	60
O'Brien, 2003	NO	YES	NO	150
Olutoye, 2003	NO	YES	NO	204

Oztekin, 2003	NO	YES	NO	40
Paech, 2003	NO	YES	NO	118
Pan, 2003	NO	NO	NO	40
Samra, 2003†	NO	NO	NO	250
Szarvas, 2003	NO	YES	NO	130
Tang, 2003	NO	YES	NO	135
Thagaard, 2003	NO	YES	NO	96
Tzeng, 2003	YES	YES	NO	65
Usmani, 2003	NO	YES	NO	90
Wagner, 2003	NO	YES	NO	94
Wattwil, 2003	NO	YES	NO	78
Yuksek, 2003	YES	NO	NO	60
Adducci, 2002	NO	YES	NO	180
Agarwal, 2002	NO	YES	NO	100
Arcioni, 2002	NO	YES	NO	59
Argiriadou, 2002	NO	YES	NO	87
Coloma, 2002	NO	NO	NO	60
Coloma, 2002	NO	YES	NO	140
Elhakim, 2002	NO	YES	NO	180
Fabling, 2002	YES	YES	NO	46
Gan, 2002	NO	YES	NO	60
Goksu, 2002	NO	YES	NO	60
Grimsehl, 2002	NO	YES	NO	74
Jokela, 2002	YES	YES	NO	179
Lee, 2002	YES	YES	NO	113
Marcus, 2002	NO	YES	NO	120
Mullick, 2002	NO	NO	NO	75
Munro, 2002	NO	YES	NO	53
Ozmen, 2002	NO	YES	NO	60
Paech, 2002	NO	YES	NO	139
Quaynor, 2002	NO	YES	NO	107
Sanchez-Ledesma, 2002	NO	YES	NO	90
Sennaraj, 2002	NO	YES	NO	150
So, 2002	NO	YES	NO	68
Sukhani, 2002	NO	YES	NO	149
Wang, 2002a	YES	YES	NO	115
Wang, 2002b	YES	YES	NO	116
Wang, 2002	YES	YES	NO	114
White, 2002	NO	NO	NO	120
Yazigi, 2002	NO	YES	NO	100
Bowhay, 2001	NO	YES	NO	131
Cherian, 2001	NO	YES	NO	81
Dabbous, 2001	NO	NO	NO	173
De Witte, 2001	NO	NO	NO	40
Ganem, 2001	NO	YES	NO	52
Goll, 2001	NO	YES	NO	240
Kathirvel, 2001	YES	YES	NO	152
Millo, 2001	NO	YES	NO	137

Pan, 2001	YES	YES	NO	156
Paventi, 2001	NO	YES	NO	60
Shende, 2001	NO	YES	NO	240
Somri, 2001	NO	YES	NO	60
Splinter, 2001	NO	YES	NO	193
Subramaniam, 2001	NO	YES	NO	135
Thomas, 2001	NO	YES	NO	177
White, 2001	NO	YES	NO	80
Wilson, 2001	NO	YES	NO	232
Yilmazlar, 2001	NO	YES	NO	60
Ahmed, 2000	NO	YES	NO	139
Charuluxananan, 2000	NO	NO	NO	80
Fabling, 2000	YES	YES	NO	60
Gesztesi, 2000	NO	YES	NO	157
Holt, 2000	NO	YES	NO	125
Jensen, 2000	NO	YES	NO	71
Jokela, 2000	NO	YES	NO	120
Kothari, 2000	NO	YES	NO	108
Kreisler, 2000	NO	YES	NO	31
Ku, 2000	YES	YES	NO	58
Lee, 2000	YES	NO	NO	120
Liberman, 2000	NO	YES	YES	84
Najeeb, 2000	NO	YES	NO	45
Peixoto, 2000	NO	YES	NO	90
Philip, 2000	YES	YES	NO	1030
Reihner, 2000	YES	YES	NO	207
Sadhasivam, 2000	NO	YES	NO	180
Scuderi, 2000	NO	YES	NO	139
Sharma, 2000	NO	YES	NO	98
Wu, 2000	YES	YES	NO	152
Zarate, 2000	NO	YES	NO	200
Abouleish, 1999	NO	NO	NO	74
Allen, 1999	NO	YES	NO	56
Barst, 1999	NO	YES	NO	90
Broome, 1999	NO	NO	NO	109
Bugedo, 1999	NO	YES	NO	242
Cholwill, 1999	NO	YES	NO	175
de Adana, 1999	NO	YES	NO	52
Gurler, 1999	NO	YES	NO	40
Helmy, 1999	YES	YES	YES	160
Janknegt, 1999	NO	YES	NO	397
Jokela, 1999	NO	YES	NO	120
Kathirvel, 1999	NO	YES	NO	100
Khalil, 1999	NO	YES	NO	63
Koivuranta, 1999	NO	YES	NO	88
Kovac, 1999	NO	NO	NO	428
Kyokong, 1999	NO	YES	NO	400
McCall, 1999	NO	YES	NO	100

Munro, 1999	NO	YES	NO	73
Rajeeva, 1999	NO	YES	NO	51
Sadhasivam, 1999	YES	YES	NO	54
Sandhu, 1999	NO	NO	NO	87
Scuderi, 1999a	NO	YES	NO	575
Scuderi, 1999b	NO	YES	NO	NR
Sinha, 1999	YES	YES	NO	40
Swiatkowski, 1999	NO	YES	NO	134
Tsui, 1999	NO	YES	NO	121
Turhanoglu, 1999	NO	YES	NO	100
Wagley, 1999	NO	YES	NO	50
Woodward, 1999	NO	YES	NO	216
Alon, 1998	NO	NO	NO	314
Ang, 1998	NO	NO	NO	47
Bacic, 1998	NO	YES	NO	50
Chan, 1998	YES	YES	NO	148
Chen, 1998a	NO	YES	NO	78
Chen, 1998b	NO	NO	NO	80
Dabbous, 1998	NO	YES	NO	85
Daftary, 1998	NO	YES	NO	150
Dershwitz, 1998	NO	YES	NO	175
Diemunsch, 1998	YES	YES	NO	793
Doe, 1998	NO	YES	NO	45
Dresner, 1998	NO	YES	NO	60
Goodarzi, 1998	NO	YES	NO	81
Hamid, 1998	NO	YES	NO	71
Jellish, 1998	NO	NO	NO	120
Mitra, 1998	NO	NO	NO	80
Morris, 1998	NO	YES	NO	1044
Scholz, 1998	YES	YES	NO	842
Splinter, 1998a	NO	YES	NO	197
Splinter, 1998b	NO	YES	NO	216
Tang, 1998a	NO	YES	NO	218
Tang, 1998b	NO	YES	NO	156
Tramer, 1998	NO	YES	NO	157
Akcabay, 1997	NO	NO	NO	60
Alexander, 1997	NO	YES	NO	124
Ascaso, 1997	NO	YES	NO	251
Bach-Styles, 1997	NO	YES	NO	101
Carnahan, 1997	NO	YES	NO	54
Diemunsch, 1997a	NO	NO	NO	746
Diemunsch, 1997b	NO	YES	NO	281
Diemunsch, 1997c	NO	NO	NO	337
Graczyk, 1997	NO	NO	NO	635
Klockgether-Radke, 1997	NO	YES	NO	120
Koivuranta, 1997	NO	YES	NO	439
Korttila, 1997	YES	YES	NO	517
Kovac, 1997	NO	NO	NO	620

Lawhorn, 1997	NO	YES	NO	320
Maestre, 1997	YES	NO	NO	233
McKenzie, 1997	NO	YES	NO	80
Mikawa, 1997	YES	YES	NO	200
Moens, 1997	YES	YES	NO	206
Monagle, 1997	NO	YES	NO	91
Morton, 1997	NO	YES	NO	427
Patel, 1997	NO	NO	NO	429
Pitkanen, 1997	NO	YES	NO	73
Polati, 1997	NO	NO	NO	175
Purhonen, 1997	YES	YES	NO	146
Rung, 1997	NO	NO	NO	121
Scuderi, 1997	NO	YES	NO	160
Shende, 1997	NO	YES	NO	176
Sniadach, 1997	NO	YES	NO	158
Splinter, 1997	NO	YES	NO	239
Stienstra, 1997	NO	NO	NO	74
Sun, 1997	NO	YES	YES	75
Taylor, 1997	NO	NO	NO	523
Ulusoy, 1997	NO	NO	NO	40
Warriner, 1997	YES	YES	NO	374
Ali-melkkila, 1996	NO	NO	NO	120
Alon, 1996	NO	YES	NO	80
Capouet, 1996	YES	YES	NO	385
Chen, 1996	NO	YES	NO	49
Cieslak, 1996	NO	YES	NO	97
Davies, 1996	NO	YES	NO	66
Ercelen, 1996	NO	YES	NO	100
Eriksson, 1996	YES	YES	NO	58
Furst, 1996	NO	YES	NO	60
Gaynes, 1996†	NO	NO	NO	87
Honkavaara, 1996	YES	YES	NO	49
Kaul, 1996	YES	YES	NO	241
Khalil, 1996	NO	NO	NO	375
Kimya, 1996	NO	YES	NO	140
Koivuranta, 1996	NO	YES	NO	63
Kovac, 1996 (CR: Pearman, 1994c)	YES	YES	NO	468
Kumar, 1996	NO	YES	NO	30
Lawhorn, 1996	NO	YES	NO	165
Lekprasert, 1996	NO	YES	NO	82
Lopez-Orlaondo, 1996	YES	YES	NO	100
Naguib, 1996	YES	YES	NO	132
Pan, 1996	NO	NO	NO	48
Principi, 1996	NO	YES	NO	60
Pueyo, 1996	YES	YES	NO	100
Pugh, 1996	NO	YES	NO	60
Rodrigo, 1996	NO	YES	NO	32

Rose, 1996a	NO	YES	NO	136
Rose, 1996b	NO	YES	NO	200
Stene, 1996	NO	YES	NO	120
Tang, 1996	NO	YES	NO	161
Usha, 1996	NO	YES	NO	92
Van Den Berg, 1996a	YES	YES	NO	220
Van Den Berg, 1996b	NO	YES	NO	282
Van Den Berg, 1996c	YES	YES	NO	148
Watts, 1996	NO	YES	NO	167
Wilson, 1996	YES	YES	NO	527
Wrench, 1996	NO	YES	NO	60
Yilmazlar, 1996	NO	YES	NO	60
Alexander, 1995	NO	YES	NO	127
Campbell, 1995	NO	YES	NO	32
Davis, 1995a	NO	YES	NO	213
Davis, 1995b	NO	YES	NO	95
Desilva, 1995	NO	NO	NO	286
Elhakim, 1995	NO	YES	NO	50
Grond, 1995	NO	YES	NO	80
Litman, 1995	NO	YES	NO	57
Mikawa, 1995	YES	YES	NO	120
Paech, 1995	NO	YES	NO	259
Paxton, 1995a	NO	YES	NO	60
Paxton, 1995b	NO	YES	NO	118
Splinter, 1995a	NO	YES	NO	233
Splinter, 1995b	NO	YES	NO	276
Watcha, 1995	NO	YES	NO	130
Furst, 1994	NO	YES	NO	239
Gan, 1994	YES	YES	NO	120
Kaufmann, 1994	YES	YES	NO	286
Khalil, 1994 (CR: Pearman, 1994b)	NO	NO	NO	589
Litman, 1994	NO	YES	NO	60
Malins, 1994	NO	YES	NO	150
McKenzie, 1994	NO	YES	NO	180
Rodrigo, 1994	NO	YES	NO	77
Rose, 1994	NO	YES	NO	100
Suen, 1994	NO	YES	NO	204
Ummenhofer, 1994	NO	YES	NO	200
Volpe, 1994	NO	YES	NO	928
Dupeyron, 1993	NO	YES	NO	243
Helmers, 1993	NO	YES	NO	923
McKenzie, 1993b (CR: Dershwitz, 1992)	NO	YES	NO	137
Raphael, 1993	NO	YES	NO	123
Sung, 1993	NO	YES	NO	180
Zomers, 1993	NO	YES	NO	69
Alon, 1992	NO	YES	NO	66

Du Pen, 1992 (CR: Scuderi, 1993)	NO	NO	NO	500
Kenny, 1992	NO	NO	NO	982
Kovac, 1992 (CRs: McKenzie, 1993a; Pearman, 1994a)	NO	NO	NO	580
Bodner, 1991	NO	NO	NO	71
Larijani, 1991	NO	NO	NO	36
Leeser, 1991	NO	YES	NO	84
Non randomized clinical trials (n=5)				
Stamer, 2011	NO	NO	NO	141
Rabbani, 2010	NO	NO	NO	60
Kim, 2007	NO	NO	NO	2839
Biedler, 2004	NO	NO	NO	159
Manani, 1996	NO	YES	NO	200
Controlled before-after study (n=1)				
Wagner, 2007	NO	NO	NO	66
Cohort studies (n=9)				
Shakeel, 2012	NO	NO	NO	432
Chen, 2011	NO	NO	NO	90
Eryilmaz, 2008	NO	NO	NO	212
Habib, 2007	NO	NO	NO	3814
Flynn, 2006	NO	NO	NO	849
Wang, 2005	NO	NO	NO	38
Piwko, 2005	NO	NO	NO	282
Walker, 2001	NO	NO	NO	59
O'Brien, 2000	NO	NO	NO	200
**Total included studies**	66	217	4	

Note: †Unpublished data (Gurha, 2012; Apfel, 2011; White, 2005; Samra, 2003; Gaynes, 1996).
Abbreviations: CR, companion report; NMA, network meta-analysis; NO, study wasn't included in review; YES, study was included in review.
